# Leave or Stay: Simulating Motility and Fitness of Microorganisms in Dynamic Aquatic Ecosystems

**DOI:** 10.3390/biology10101019

**Published:** 2021-10-09

**Authors:** Alexandra Klimenko, Yury Matushkin, Nikolay Kolchanov, Sergey Lashin

**Affiliations:** 1 Systems Biology Department, Institute of Cytology and Genetics, Siberian Branch of the Russian Academy of Science, Lavrentiev Avenue 10, 630090 Novosibirsk, Russia; mat@bionet.nsc.ru (Y.M.); kol@bionet.nsc.ru (N.K.); lashin@bionet.nsc.ru (S.L.); 2 Kurchatov Genomics Center, Institute of Cytology and Genetics, Siberian Branch of the Russian Academy of Science, Lavrentiev Avenue 10, 630090 Novosibirsk, Russia; 3 Natural Science Department, Novosibirsk State University, Pirogova St. 1, 630090 Novosibirsk, Russia

**Keywords:** motility, migratory costs, marine bacteria, agent-based modelling, ecological modelling

## Abstract

**Simple Summary:**

Motile bacteria are widespread in various water ecosystems along with nonmotile species, which posits the question: what makes motility an advantage in such habitats, and under what conditions? This simulation study addresses these problems using a computer model of competition of two microbial species: Nomad of a motile population and Settler of a sedentary one. We simulated their competition under various environmental conditions such as the nutrient availability and frequency of changes in the location of the nutrient source as well as depending on some population parameters determining how much energy it takes for a bacterium to migrate and what the effect of density-dependent mortality is on the outcome of Settler vs. Nomad competition. We showed that dynamic and nutrient-scarce environments favour motile populations, whereas nutrient-rich and stagnant environments promote sedentary microorganisms. Moreover, the energetic costs of migration determine whether or not the motile population outcompetes the sedentary one, though it also depends on such conditions as nutrient availability. There is also another way for Settler to succeed even without penalties for migration—by grasping an opportunity to occupy the nutrient source, bringing about a biotic desert around it, which cannot be overcome by Nomad constantly searching for locally optimal conditions.

**Abstract:**

Motility is a key adaptation factor in scarce marine environments inhabited by bacteria. The question of how a capacity for adaptive migrations influences the success of a microbial population in various conditions is a challenge addressed in this study. We employed the agent-based model of competition of motile and sedentary microbial populations in a confined aquatic environment supplied with a periodic batch nutrient source to assess the fitness of both. Such factors as nutrient concentration in a batch, batch period, mortality type and energetic costs of migration were considered to determine the conditions favouring different strategies: Nomad of a motile population and Settler of a sedentary one. The modelling results demonstrate that dynamic and nutrient-scarce environments favour motile populations, whereas nutrient-rich and stagnant environments promote sedentary microorganisms. Energetic costs of migration determine whether or not the Nomad strategy of the motile population is successful, though it also depends on such conditions as nutrient availability. Even without penalties for migration, under certain conditions, the sedentary Settler population dominates in the ecosystem. It is achieved by decreasing the local nutrient availability near the nutrient source, as motile populations relying on a local optimizing strategy tend to follow benign conditions and fail, enduring stress associated with crossing the valleys of suboptimal nutrient availability.

## 1. Introduction

In contrast to well-studied symbiotic gut microbiota living in abundant nutrient conditions, marine bacteria live in the world of scarce and ephemeral nutrition sources. Hence, motility is one of the key factors of adaptation in these conditions [1], providing bacteria with an advantage in resource-limited physically structured habitats, even in the case of undirected motility [2]. The capacity to sense the gradients of essential chemicals, which is known as chemotaxis, is very important for the adaptive potential of bacterial motility [3,4]. The biophysics and molecular and signalling machinery of chemotaxis in model organisms such as *Escherichia coli* are relatively well-studied at the microscale level [5,6,7,8,9,10,11], namely the results of the immediate reaction of bacterial cells to their local environment. However, there is a lack of understanding of how the ability for adaptive migrations influences the evolutionary success of a population. Overall, adaptive migrations of microorganisms act as a key factor involved in resolving the struggle for existence in microbial ecosystems [12,13].

There are two different strategies microorganisms resort to when facing environmental changes: either the adaptation to existing conditions, e.g., by adjusting their metabolism to an alternative energy source; or migration following the optimal environmental conditions and invading new biotopes, where they compete with the species of a local community [14,15,16]. Both strategies are relevant for marine bacteria who can either attach to the nutrient sources and colonize it by growing a biofilm or rapidly detect short-lived nutrient sources and constantly move towards them as fugitive species do [17]. The impact of such an adaptive behaviour at the scale of individuals on the community-level dynamics is of a fundamental scientific interest since it is important for achieving a comprehensive understanding of structure and functioning of respective ecosystems. Moreover, this interconnection is covered by the perspective on particular aspects of ecology and evolution of search behaviour [18].

The problem of interrelation between migratory activity and population fitness has been investigated in many species; among those are marine fish such as the Salmonidae family, bacteria (*Escherichia coli*) and amoeba (*Dictyostelium discoideum*) [19,20,21,22]. Motility improves foraging behaviour and raises the chances of successful mating; however, a migratory activity of organisms incurs certain energetic costs, whereas the cell’s energy reserve might be invested into the reproduction instead of migration [23,24]. Various groups of organisms manage this emerging trade-off between reproductive investment and migration differently. For instance, chinook salmon resort to developmental modification, resulting in an increased number-to-size ratio of eggs with a greater migration distance [25]. However, unicellular organisms cannot enjoy such flexibility at the tissue- and organ-level and are compelled to meet the challenge by adopting a particular life strategy on a species level or, in some cases, at the level of the population by implementing diversified phenotypes [26] and dynamic life strategies [27]. Motility and chemotaxis are widespread among bacteria inhabiting marine environments [1,28,29], though nonmotile groups such as SAR11 [30,31] and *Prochlorococcus* [32] do exist as well. The cost of motility varies for different species: marine bacteria, such as *Vibrio alginolyticus* and *Pseudoalteromonas haloplanktis*, swim ~3- to 5-fold faster than *E. coli*, whose typical velocity range is 15 to 30 μm/s, and spend a ~10- to 25-fold larger amount of energy (because the propulsive power increases with the square of the swimming speed) [28]. Consider the fact that the most expensive part of *E. coli*’s motility is the synthesis and operation of flagella, which constitutes ~2% and ~0.1% of their energy expenditure, respectively [33]. From here, one can obtain an estimate of the energy expenditure spent for motility among marine bacteria ranging from 2% to 50% of their total energy budgets. In this way, the fitness cost of motility in bacteria is associated with both direct spending energy on movement (e.g., via rotating flagella) and with the maintenance of respective molecular machinery, especially the biosynthesis of flagella [34]. Thus, migration via chemotaxis can be quite expensive for the energy budgets of motile bacteria posing a trade-off between energy investment into optimal foraging strategies and allocation of resources into direct reproduction. This growth–motility trade-off has been recently explored in [23], where the regulation of such complex and costly bacterial behaviours as motility and chemotaxis has been investigated as a function of the bacterial growth rate, both theoretically and experimentally. Studying the impact of such a trade-off on a population’s fitness in aquatic ecosystems constitutes a great challenge for modelling eco-evolutionary processes, and that is the question we would like to address in this simulation study.

## 2. Materials and Methods

Despite some recent experimental progress [23], the problem of interconnection between a migratory capacity of organisms and their fitness remains largely obscure. Moreover, high-throughput pairwise competition experiments might be challenging because they are costly, and it is difficult to assess species-specific migratory costs in vivo and to choose appropriate model species. On the other hand, computational simulation enables a researcher to investigate the space solution thoroughly and draw conclusions for a general case. To a certain extent, one can consider this problem to be a special case of the interconnection between genotype and phenotype. In this case, we deal with a complex behavioural phenotype that is influenced by a number of factors ranging from the nutrient concentration in the local environment of motile cells to ecological interactions with the representatives of other species.

**Figure 1:**
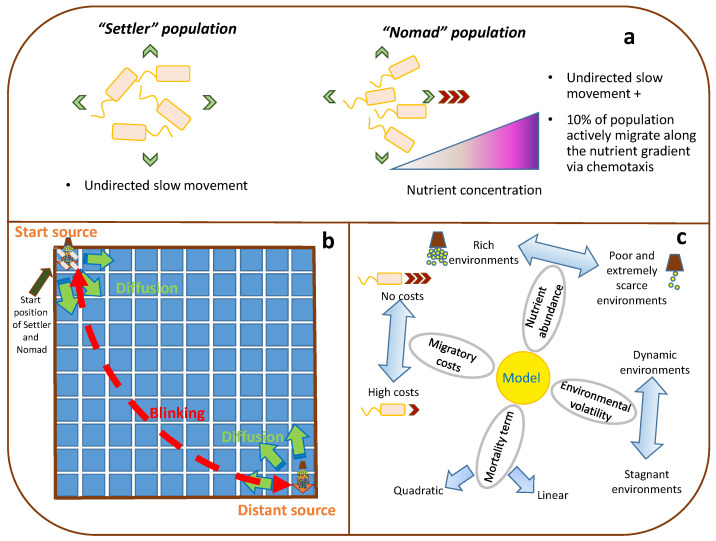
Conceptual diagram of two population strategies (**a**), the habitat structure (**b**) and simulation scenarios (**c**).

Therefore, to obtain the answers to the questions raised above, we have built a simplified model of competition of two microbial populations in confined aquatic environment supplied with a periodically blinking batch nutrient source. One of the populations possesses a chemotaxis capacity being able to migrate adaptively along the nutrient gradient, whereas another one is sedentary and distributes undirectedly by the process of passive transport (e.g., as a result of cell shoving), which, on a large scale, is similar to diffusion for substances (see Figure 1a). A comparison of final abundances of two competing populations with different strategies in various conditions sheds light on whether migration grants an advantage in these conditions or not compared to spending energy for immediate reproduction.

### 2.1. The Settler–Nomad Model

We have used the Haploid Evolutionary Constructor (HEC) [35,36,37] software to build the models of competition of motile and sedentary populations of microorganisms in a confined aquatic environment supplied with a periodic batch nutrient source. The HEC is designed for building agent-based models of microbial communities [38]. It is based on the super-individual concept [39] and allows creating multilayer ecological models of microbial populations inhabiting spatially structured environments. The basic functionality of HEC (see Supplementary Materials for a detailed description) was extended to take into account the diversity in energetic costs of migration.

To estimate which conditions allow the motile population to make use of its advantage and dominate over the sedentary one, we have investigated a model of a community consisting of two microbial populations in different model scenarios (see Section 2.2 for details). These populations inhabit a spatially structured environment supplied with a periodic source of a nutrient (also called nonspecific substrate or NS), which distributes throughout the environment via diffusion. A 10 × 10 2-dimensional (2D) square lattice with non-permeable boundaries represents the spatial structure of the habitat in the model (see Figure 1b). Such an environment can be regarded as a counterpart of a stagnant pond. The nutrient source blinks periodically between the two opposite corners of the lattice. That is, the nutrient comes into the system in batches periodically either through the top-left corner or through the bottom-right corner at a time. Let us call “start source” the NS source position that coincides with the start location occupied by both populations of microorganisms and “distant source” the NS source position that is located in the opposite corner of the lattice. Thus, both nutrient sources refresh asynchronously—the “start source” goes first and after a certain time period elapses it turns out to be depleted while the nutrient is supplied via the “distant source” for the same period until the nutrient source blinks back to the start position and the cycle runs all over again. This blinking periodic change of nutrient supply is one of the simplest cases of a dynamically changing habitat with contrasting conditions, which might be associated with external factors that result in periodic appearance of nutrient sources in different parts of the system, such as, for example, periodic organic effluents to the aquatic environment. The time step is synchronized with an average generation turnover, which is considered equal to 30 min to be definite. Initially, the nutrient is distributed spatially homogeneously, but subsequently, the heterogeneity increases due to the localized batch nutrient source and foraging activity of cells.

Henceforth, we will refer to the population of motile microorganisms as Nomad and to the population of sedentary microorganisms as Settler. We will also refer to the described above model as the Settler–Nomad model.

At the [*i*,*j*] node of the spatial lattice, the population abundances on the (*n* + 1)-th iteration of both Nomad and Settler populations obey the following balance equation:

(1) $$P_{n+1}\left[i,j\right]=growth\left(P_{n}\left[i,j\right],r\left[i,j\right]\right)-mort\left(P_{n}^{Settler}\left[i,j\right],P_{n}^{Nomad}\left[i,j\right]\right)+\;\;+immigr\left(P_{n}\left[neighbourhood\right]\right)-emigr\left(P_{n}\left[i,j\right]\right)$$

where $P_{n+1}\left[i,j\right]$ is the population abundance on the (*n* + 1)-th iteration, *r*[*i*,*j*] is the number of accumulated nutrient molecules by the cells of the population (population energy reserve), $immigr\left(P_{n}\left[neighbourhood\right]\right)$ is the population abundance increase associated with the cells of the same population immigrated into the [*i*,*j*] node from its 4-cross neighbourhood, $emigr\left(P_{n}\left[i,j\right]\right)$ is the population abundance decrease associated with the cells of the same population emigrated from the [*i*,*j*] node (both processes are described in the Supplementary Materials, Document S1) and $growth\left(P_{n}\left[i,j\right],r\left[i,j\right]\right)$ is the term that expresses the population’s growth with the nutrient availability as a limiting factor:

(2) growth(Pn[i,j], r[i,j])=Pn[i,j]⋅(1+r[i,j]K1+r[i,j](B⋅K+N))

where *N* is the genetically determined nutrient utilization efficacy (in this paper, *N* = 1), *B*, *K* are the adjustable parameters shaping the abundance curve (see Supplementary Materials, in our case, *K* = 5, *B* = 0.8), and $P_{n+1}\left[i,j\right]$ and *r*[*i*,*j*] are the same as in (1). In this study, we do not model the energy reserve explicitly and use the consumed nonspecific substrate molecules as its proxy instead. The process of the consumption of nutrients including the nutrient consumption rate as well is described in detail in the Supplementary Materials. Note that if the reserve is depleted (*r* = 0), there is no immigration and emigration—the migration flow halts.

Furthermore, $mort\left(P_{n}^{Settler}\left[i,j\right],P_{n}^{Nomad}\left[i,j\right]\right)$ is a mortality term, i.e., an equation term that describes the way of natural population decline. We consider two types of mortality: quadratic (density-dependent) and linear (proportional). In the first case, the mortality term is described as follows: $mort\left(P_{n}^{Settler}\left[i,j\right],P_{n}^{Nomad}\left[i,j\right]\right)=death_{coeff}\cdot \;(P_{n}^{Settler}\left[i,j\right]+P_{n}^{Nomad}\left[i,j\right]){}^{2}$, and in the second case: $mort\left(P_{n}^{Settler}\left[i,j\right],P_{n}^{Nomad}\left[i,j\right]\right)=death_{coeff}\cdot \;(P_{n}^{Settler}\left[i,j\right]+P_{n}^{Nomad}\left[i,j\right])$, where $death_{coeff}$ is the death coefficient. As one can see, the mortality rate of Nomad or Settler is a function of the overall density of both populations in the local node and local overpopulation casts its adverse effects on both populations. Thus, the interspecific competition between Nomad and Settler breaks into two parts—the competition for the available nutrients in the environment and spatial competition for the nodes to inhabit. Though the same node can be occupied by the cells of both populations, this situation is quite unfortunate for them because it leads to a lesser per-cell nutrient uptake and higher mortality toll.

As has been already mentioned, the population abundance in a particular node depends not only on the reproduction, which is described in Equation (2), and death of cells but also on migration fluxes (see Equation (1)) brought about by both active movement of cells and their passive advection. We take into account the adaptive migration of cells via chemotaxis—the detailed description of the used algorithm one can find in [36,37] and in the Supplementary Materials—but it is worth mentioning some of its crucial points here. A free-floating portion of actively migrating cells (we take 10% of the population in a particular node, which is in a broad agreement with observed values for coastal seawater samples [29]) is divided between favourable directions according to the attraction values of respective lattice nodes. We estimate the attraction of environmental conditions in the adjacent (neighbour) nodes based on the difference between attractants in the neighbour and the current nodes. After that, neighbour nodes are separated into two lists—unappealing nodes with lower attraction values than it is in the current node and attractive nodes possessing higher attraction values. The difference of these two lists defines the change of population size in the current node (see Supplementary Materials for a detailed description). It should be noted that the portion of actively moving cells in the current node divides between all favourable directions and the share of a direction is proportional to its attraction value. Thus, we assume motile cells to be capable of chemotaxis to spread through the environment due to both random swimming and by following nutrient gradients, whereas the cells without chemotactic capacity only spread randomly, i.e., without any specific direction.

To take into account the migration costs, we have proposed a sub-model of penalty for migration with two parameters. The first parameter (*h*) controls the degree of nonlinearity in the migratory costs function (see Equation (3)) and it is fixed in all simulation scenarios. The second parameter (*x*) corresponds to a basic genetic background determining the migration costs, and it varies throughout different simulation scenarios, bringing about Nomad populations characterized by various energetic costs of migration. Both parameters can be attributed to the costs associated with the maintenance of respective genetic regulatory networks involved in the mechanisms that control the chemotaxis machinery in any particular microorganism. In the case wherein the cell’s energy reserve is less than is necessary to pay the penalty fee, no migration is performed and no energy is expended (details of the cell’s energy balance is below, see Equation (5)).

**Figure 2:**
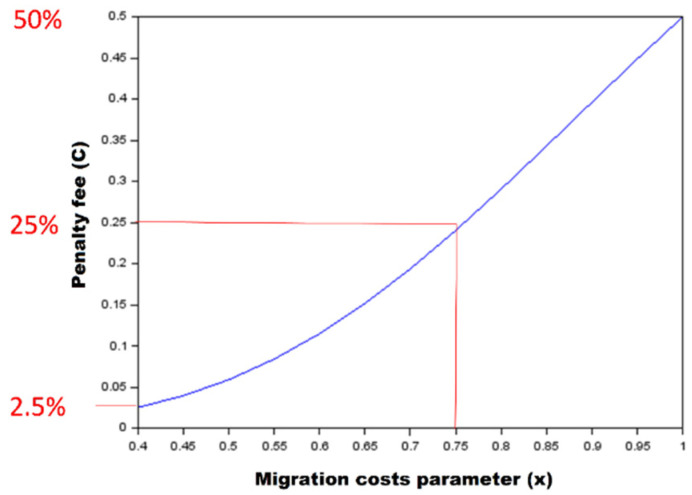
The dependence of migration penalty fee on migration costs parameter under nonlinearity parameter *h* = 4.

Thus, we regard *C*(*x*) as a *migratory costs function* (see Figure 2), and the following equation binds migration energy costs and organism-specific parameters controlling energy expenditure associated with its motility:

(3) $$C\left(x\right)=\frac{x^{h}}{1+x^{h}}$$

where *x* is the value of the parameter that controls basic migration costs; in this article we treat *x* as a variable of *C*(*x*), i.e., *C*(*x*) is a function of *x*; and h is the value of the parameter that controls the degree of nonlinearity between the migratory costs function and basic migration costs.

Henceforth, we evaluate the penalty for migration as a fraction of the cell’s maximal energy reserve value that is expended if the cell migrates (see (4–6) for a detailed description of the dynamics of consumed amount of nutrient in the cells of a particular population). In this model, we regard the consumed nonspecific substrate (NS) resources as an energy reserve of a cell (see Table S1 in the Supplementary Materials for a summary of Settler–Nomad model state variables). Thus, the penalty for migration represents the actual spending accumulated NS for motility. The equation that reflects the dynamics of energy reserves of a particular population in the [*i*,*j*]-node before the migration simulation step is applied as follows:

(4) $$r_{n+1}{}^{\prime }\left[i,j\right]=r_{n}\left[i,j\right]+NS_{consumed\;}$$

where *NS_consumed_* is the number of molecules of the nutrient consumed by the cells belonging to the population under consideration (see paragraph 2.1 in the Supplementary Materials for the detailed description of the nutrient consumption submodel).

Then, taking into account passive transport of cells and active motility via chemotaxis, we derive the following formula:

(5) $${r_{n+1}}^{\prime \prime }\left[i,j\right]=\{\begin{array}{cc} r_{n+1}{}^{\prime }\left[i,j\right]-M\cdot C\left(x\right)+r_{pass\_immigr}-r_{pass\_emigr}+r_{act\_immigr}-r_{act\_emigr}, & \frac{r_{n+1}{}^{\prime }\left[i,j\right]}{P_{n}}>M\cdot C\left(x\right)\\ r_{n+1}{}^{\prime }\left[i,j\right]+r_{pass\_immigr}-r_{pass\_emigr}, & \frac{r_{n+1}{}^{\prime }\left[i,j\right]}{P_{n}}\leq M\cdot C\left(x\right) \end{array}$$

where *r_pass_immigr_* and *r_pass_emigr_* are the energy reserve income and outcome via passive transport of cells of the population into and out of the current node, respectively; *r_act_immigr_* and *r_act_emigr_* are the energy reserve income and outcome via immigration and emigration of cells of the population into and out of the current node, respectively, mediated by chemotaxis; *P_n_* is the population abundance; *M* is the maximal amount of nutrient molecules that can be consumed per cell; and *C*(*x*) is a migratory costs function. *r_pass_immigr_*, *r_pass_emigr_*, *r_act_immigr_* and *r_act_emigr_* dynamics are described in the Supplementary Materials, paragraph 1.4.

(6) $$r_{n+1}\left[i,j\right]=(1-R-k_{degrad})\cdot r_{n+1}{}^{\prime \prime }\left[i,j\right]$$

where *R* is the costs for reproduction and *k_degrad_* is the nutrient degradation rate. *R + k_degrad_* = 0.999.

Let us clarify the biological assumptions underlying the chemotaxis simulation algorithm. Since we are interested in population dynamics and the time scale of HEC is calibrated accordingly based on the mean generation time, the effects of chemotaxis appear on the same scale and we consider its integral effect on a population level. For this reason, we do not describe movement irregularities of distinct cells on short time intervals (such as seconds or split seconds) in detail in the model since they appear to be averaged out on this simulation scale. Meanwhile, other factors step forward and influence the effect of motility on a population dispersion—namely, the adaptation to the baseline signal of attractants in the current location and rough attractant gradient on a wider spatial scale. The former is achieved by the one-off nature of a chemotaxis act—the cells have moved to the adjacent node and their propulsion does not have momentum [40], they adapt to the baseline signal as the literature on bacterial chemotaxis signalling pathways indicates [11]. The latter is attained by the algorithm evaluating the attraction values of nodes [36]. A cell swimming via chemotaxis expends energy. Those cells who run out of their energy reserves halt their flagellar motor rotation until they gain more energy again [41]. Thus, a constraint for an active movement for cells that do not have enough energy has a clear physical sense.

### 2.2. Simulation Scenarios

We have investigated two groups of simulation scenarios—those without migration penalty and those with energetic costs of migration. Both groups follow the Settler–Nomad model described in Section 2.1 and we varied such parameters as nutrient concentration in a batch, a batch period and a mortality term (see Table 1 for the details). For the model that takes into account migration energy costs, we varied migration penalty fee value as well. See Figure 1c for a conceptual diagram of examined simulation scenarios and corresponding varied parameters.

Lower batch period values correspond to the environments that are more dynamic, whereas higher batch period values correspond to the ones that are more stagnant. Similarly, different values of nutrient concentration in a batch, i.e., the amount of nutrients that are added into the node located at the nutrient source, produce the environments with different nutrient richness. We will call a habitat characterized by high nutrient concentration in a batch (1 × 10^−1^ M) a “rich” environment and that characterized by low concentration (1 × 10^−5^ M) a “poor” one. The environments bearing very low NS concentration in a batch (1 × 10^−10^ M) will be called “extremely scarce” hereafter. Comparing the quadratic mortality with a linear one allows us to conclude whether density-dependent mortality accounts for the effects observed in the model or is explained by a deeper underlying cause. Both types of mortality terms are described in Section 2.1.

All groups of simulation scenarios were run for 9000 iterations yielding simulation results on population dynamics in the system, which covers a time period of half a year.

## 3. Results

In this study, we have investigated the model of competition of motile and sedentary species and the impact of a capacity for adaptive migration on a population abundance in various conditions. There is a number of factors—both population and environmental ones—that shape the context of competition between motile and sedentary microorganisms. The nutrient concentration in a batch and a batch period are among environmental factors, while migration energetic costs and a mortality term, describing the way of natural population decline, can be regarded as intrinsic traits of the populations. It should be noted that two kinds of simulation scenarios describing mortality either as quadratic or linear term correspond to the classic Verhulst density-dependent mortality and proportional mortality caused by constant adverse factors, respectively, such as, e.g., average pressure of external amensals that are not taken into account in the examined ecosystem. Comparing the simulation results of two types of the model—one that takes into account energetic costs of migration and one that does not—allows us to answer the following questions:

- What are the critical migration penalty fee values that Nomad can bear while keeping the dominance in the system?
- How does it depend on other environmental and population factors?

### 3.1. The Impact of Motility on Fitness

In order to draw the baseline, first of all, we have calculated the simulation results for the model that do not take into account energetic costs of migration. That is one pole corresponding to the situation when the mechanism underlying cells’ motility is designed quite effectively so that the energetic costs of migration are negligible. These results are summarized in the Table 2.

The simulations show that the mortality term, the batch period and the nutrient concentration in a batch affect the dominance patterns in the ecosystem. It should be mentioned that though this fact is not included in Table 2, in the “extremely scarce” environment (1 × 10^−10^ M nutrient concentration in a batch), Nomad outcompetes Settler, making use of its motility advantage regardless of the batch period (50≤period ≤1000 iterations), and it does even under quadratic mortality. However, further examination of the model shows that the Nomad’s dominance can be challenged under certain conditions.

**Figure 3:**
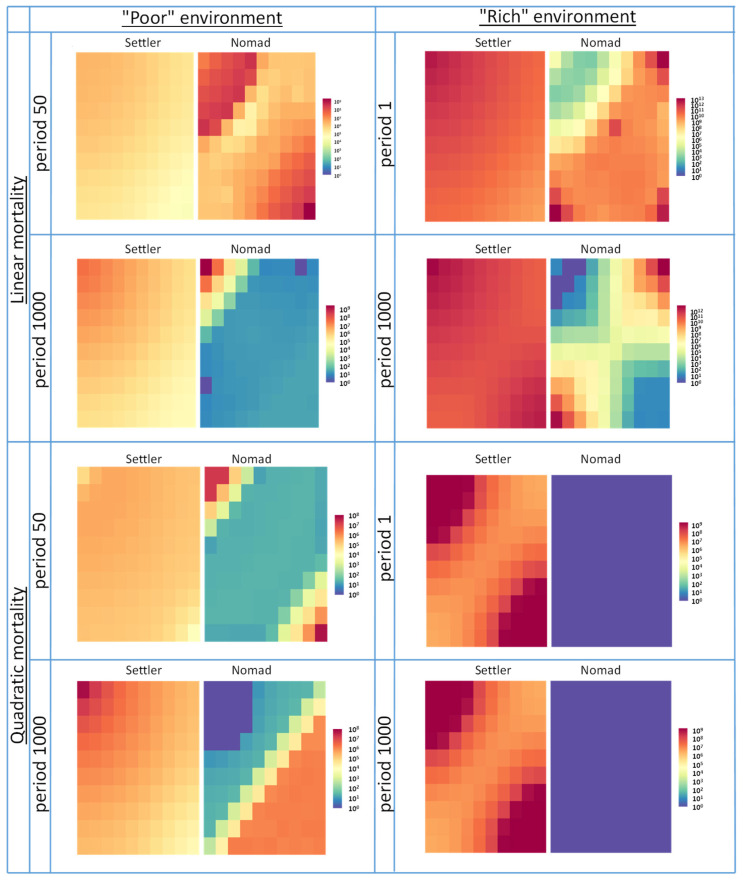
Heatmaps of spatial dispersion snapshots of Settler and Nomad cells in the habitat taken at the end of the simulation run under various simulation scenarios. The heatmap scale represents the abundance measured in the number of cells. The “start” nutrient source is located in the upper-left corner while the “distant” source is located in the opposite bottom-right corner.

Similarly to the “extremely scarce” case, under linear mortality in a “poor” environment (1 × 10^−5^ M nutrient concentration in a batch), Nomad outcompetes Settler, regardless of the batch period (see Table 2). It is achieved due to Nomad moving freely between both nutrient sources and occupying the space that is more beneficial at every time moment (see spatial population dynamics time snapshots in the Supplementary Materials). However, the situation changes under quadratic mortality in a “poor” environment where the effect of density-dependent factors results in the fact that in stagnant environments characterized by a long batch period (period 1000); though Settler dominates in the system, the populations polarize according to the nutrient sources—Nomad occupies the “distant” source, while Settler controls the “start” nutrient source. One can see it clearly looking at the spatial dispersion of cells (see Figure 3), which allow to disentangle how exactly Settler and Nomad share the common habitat.

In “rich” (1 × 10^−1^ M nutrient concentration in a batch) environments under linear mortality, Settler controls the “start” nutrient source regardless of the batch period (see Table 2). However, the batch period influences the situation around the “distant” nutrient source; in more dynamic environments, Nomad manages to occupy it, while in more stagnant ones, as the batch period steadily increases, Settler pushes Nomad to the outskirts of the habitat. In the case of quadratic mortality in “rich” environments, the Nomad population is disproportionately affected by density-dependent factors while pursuing the most favourable conditions and it goes extinct while Settler thrives regardless of the batch period value.

Settler’s domination around the “start” source can be explained by the initial location of the sedentary population; while Nomad leaves, Settler stays around the start source, establishing its dominance. Moreover, as has been discussed above, it holds not only for the “rich” environments but also for the “poor” ones, though in the latter case, Nomad has more opportunities, especially if the batch period is low. However, further investigation taking into account various initial distributions of cells is needed to conclude whether this effect is robust or not.

Thus, even without any migration penalty fees only under certain conditions Nomad is able to take an advantage from its adaptive behaviour—namely in nutrient poor and dynamic environments where spatial localization of the nutrient source changes quickly bringing about dynamic inequality in the nutrient availability.

### 3.2. The Impact of Diversity in Energetic Costs of Migration

Introducing the migration penalty fee, it is important to find out what the thresholds of its value are that determine whether Nomad dominates in the habitat or Settler does. We have scrutinized the impact of the migration penalty fee value on the outcome of the competition in the Settler–Nomad model under different mortality types and in the environments characterized by different nutrient concentration in a batch but the same fixed batch period that equals to 50 generations. The summary of the results is presented in Table 3 and Table 4.

The observed patterns do not fit into plain linear correlations. However, as the penalty fee value increases, Nomad’s abundance fraction decreases until it faces a certain limit, which varies for the environments characterized by different nutrient concentrations in a batch. This fact can be explained by either Nomad’s lacking energy reserves to move anywhere as the migration penalty fee increases and nutrient availability decreases or by spending those limited resources to establish in already occupied nodes rather than to reclaim new water areas.

In the relatively abundant conditions characterizing intermediate environments between the “rich” (1 × 10^−1^ M nutrient concentration in a batch) and the “poor” (1 × 10^−5^ M nutrient concentration in a batch) ones, introducing a migration penalty fee results in Nomad’s fraction becoming negligible up to the complete extinction of Nomad (see Table 3). Notably, the mortality type does not affect this tendency significantly, though taking into account density-dependent factors worsens the implications for Nomad, whereas linear mortality mitigates them.

**Figure 4:**
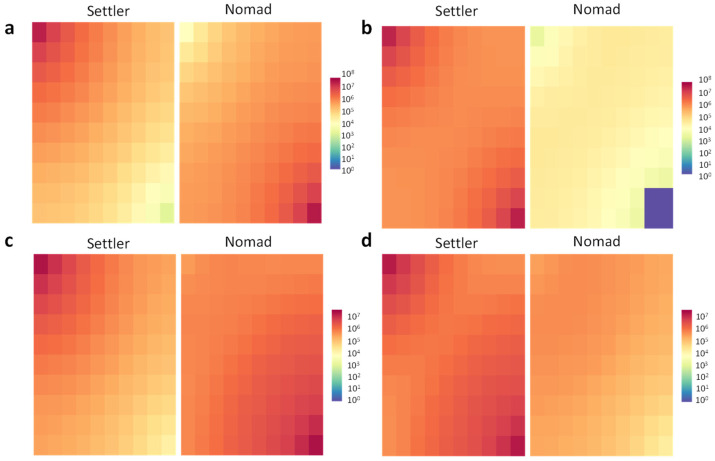
The effect of the migration penalty fee value on the spatial dispersion of Settler and Nomad cells in the habitat (the snapshots are taken at the end of the simulation run under quadratic mortality, the results under linear mortality does not differ significantly). Heatmap scale represents the abundance measured in the number of cells. The “start” nutrient source is located in the upper-left corner while the “distant” source is located in the opposite bottom-right corner. One can see that as the energetic costs of migration increases, the dominance switch occurs in the following cases—turning from scenario with nutrient concentration in a batch equal to 1 × 10^−5^ M and penalty fee value equal to 2% (**a**) to the scenario with the same nutrient concentration in a batch but higher penalty fee value equal to 5% (**b**), and turning from scenario with nutrient concentration in a batch equal to 1 × 10^−6^ M and penalty fee value equal to 5% (**c**) to the scenario with the same nutrient concentration in a batch but higher penalty fee value equal to 10% (**d**).

It is worth noting that there is a certain turning point in migration penalty fee value that determines quite narrow area of parameter space where Nomad strategy turns out to be successful and the value of that point depends on environmental conditions, in particular on the nutrient concentration in a batch. In this respect, the picture of spatial dispersion of cells belonging to different populations might answer what the causes underlying this dominance switch are (see Figure 4).

It is evident from Figure 4 that if there is no clear dominance of either Settler or Nomad (see Figure 4a,c), both populations separate spatially and divide control over different nutrient sources—Nomad takes over the “distant” source while Settler firmly occupies the “start” source. This is in good correspondence with the previous studies [17] showing that such competition-dispersal trade-offs do occur in microorganisms and bring about ecologically differentiating populations who either specialize to colonize particles by attaching and growing biofilms, or hone rapid detection and swimming to reach short-lived particle patches. If the dominance switch occurs abruptly after increasing the penalty fee value (see Table 3 and Figure 4 switches from a to b and from c to d scenarios), it is usually caused by the capture of both nutrient source by Settler who manages to push Nomad back while the latter sustains losses (Figure 4b,d).

A detailed investigation into the impact of the nutrient batch period is an arduous task that could be a matter of a distinct study. However, a rough estimate for the boundary cases of “rich” and “extremely scarce” (1 × 10^−10^ M nutrient concentration in a batch) environments inferred for short and long batch periods (see Table 5) demonstrates that the nutrient concentration in a batch and energetic costs of migration might play a more significant role in determining the outcome of Settler–Nomad competition than the nutrient batch period does. However, there is no conclusive analysis of the impact of the nutrient batch period at the moment, and this point is open for the further discussion.

Furthermore, since the critical value of the migration penalty fee is too close to zero, it means that actually in the “extremely scarce” environment, the Nomad strategy cannot confer real benefit if energetic costs of migration are significant (≥0.4% of maximal cell’s energy reserve). However, as was demonstrated above, there is a certain parameter interval between the “extremely scarce” and “rich” environments where it is possible and the Nomad population does gain an advantage of its motility to take the dominant position in the ecosystem.

## 4. Discussion

A capacity for adaptive migrations plays an important role in the eco-evolutionary dynamics of microbial populations determining their success in the ecosystem. However, the trade-off between migratory energy costs and investment into reproduction imposes specific constraints on employing migration-based foraging strategies. The results of our simulation study show that the success in the interspecific competition brought by a potential advantage conferred by the capacity for active migrations is limited by ecological and population factors. Moreover, under certain conditions, the Settler strategy appears to be more successful than the Nomad one, even without introducing any penalties that account for energetic costs of migration. However, the fact that in the nutrient-rich environments, even under linear mortality, Nomad concedes the leadership to Settler needs to be explained. We attribute that effect to the emergence of “biotic desert” around the Settler occupying the “start” nutrient source (see Figure S1). Though from the viewpoint of Nomad’s dispersion it is reminiscent of a “volcano effect” [42], which is the situation when steady-state bacterial aggregation forms a ring of higher density some distance away from an optimal environment, it has a different underlying cause. While the biological cause of the “volcano effect” is that the bacterium cannot sense rapid changes in its environment quickly enough, in our model, we instead have a situation stemming from the competition of two different populations. Since the Settler cells decrease the local nutrient availability in the source’s vicinity, Nomad cannot break through this self-organized “exclusion zone” to the nutrient source because the nutrient concentration around the source is low and it signals that the environmental conditions are unfavourable. Thus, fugitive motile populations relying on a locally optimal foraging strategy tend to follow benign conditions and fail enduring stress associated with crossing the valleys of suboptimal nutrient availability. However, these conclusions hold under the set of assumptions that describe a particular situation when both populations invade a vacant habitat through the same spot, while the model of blinking nutrient source is quite reductive. Though such a simplistic approach is a good way to start, to assess different cell dispersion scenarios and to reflect the transient nature of resource hotspots in the ocean, it is reasonable to take into account various initial distributions of cells as well as more a complex model of a resource landscape that changes over time and is characterized by the continuous appearance of new spots. Thus, further investigation is needed to determine whether such an effect is robust in relation to the initial location of the sedentary population and to various dynamic modes of nutrient supply.

The results of our simulation study indicate that there is a turning point in the migration penalty fee value determining whether the Nomad strategy of being a motile population is successful or not. However, this value heavily depends on the nutrient availability, and the overall spectrum of conditions favouring the Nomad strategy is estimated to be quite narrow, imposing constraints on Nomad’s trade-off between spending energy on migration and reproduction investment. It turned out that more dynamic and scarce environments favour the dominance of fugitive motile populations, whereas the cost of motility exceeds the benefit in nutrient-rich and stagnant environments, which tend to promote the dominance of sedentary microorganisms. Therefore, these constraints shape eco-evolutionary dynamics in such aquatic microbial systems affecting the processes of assembly and the evolution of microbial communities. Moreover, density-dependent quadratic mortality is more detrimental for a motile population than a linear one since chemotaxis drives its cells to organize into densely populated areas. Furthermore, though providing more adequate description, taking into account the energetic costs of migration does not change these major trends, yet it allows us to portray a more detailed picture of the competition between two types of microorganisms, relying on different strategies in terms of motility.

Notably, the results of our theoretical investigation are in accordance with some previous findings in the field. For instance, ref. [23] have shown with experiment and theory that in poor nutritional conditions, *Escherichia coli* increases its investment in motility in proportion to the reproductive fitness advantage provided by the ability to follow nutrient gradients. Though the difference of studies is quite remarkable, specifically in the used phenomenological laws of cell growth, taking into account autoinducers and the fact that they consider the nutritional value of carbon source rather than nutrient concentration in a batch, we believe that our results qualitatively relate to a comparable setting. Furthermore, the scenarios observed in our simulation study under nutrient poor conditions and bearable costs for migration exhibit spatial separation of two different populations: Nomad establishes around the “distant” source while Settler controls the “start” source. It echoes the results of [17], who showed on two recently diverged populations of *Vibrio cyclitrophicus* that while cells of one of the populations increase their uptake rate via attachment to the nutrient patches, another population exploits the temporal variability of the resource landscape employing chemotaxis. Thus, both studies stress the phenomenon of ecological differentiation between a strategy that maximizes the access to resources at the patch level and one that favours access to resources at the landscape level. However, the novelty of our results is of a systematic study of variation in migratory costs and its impact on the corresponding eco-evolutionary patterns. It should be also noted that we cannot consider the Settler population as completely nonmotile since the passive transport coefficient may be interpreted as undirected movement similar to diffusion for the substrate. From this point of view, Settler–Nomad competition portrays itself as a rivalry between blindly motile unhasty cells and swift fugitive cells possessing sensory and signalling apparatus associated with chemotaxis.

Motile forms have an advantage in nutrient-poor conditions corresponding to environments inhabited by marine bacteria. However, since we observe the prosperity of both strategies in nature and neither of the two completely supplants another, the issue of how such diversity is sustained is an intriguing question for further investigations to answer. As some studies indicate [17], the dynamic and micro-heterogeneous nature of aquatic environments might be one of the possible factors that results in a constantly changing direction of selection hindering the occurrence of selective sweeps. Another compelling problem is how both genetic and non-genetic population diversity in motility traits influences a population’s fitness. There is a number of studies addressing this question [43,44], though a comprehensive view is yet to be integrated.

## 5. Conclusions

The results presented in this paper elucidate principal aspects of competition between bacteria with different foraging strategies—Nomad of motile population actively pursuing the spots of high concentration of the nutrient and Settler of sedentary one, which trades chemotaxis ability for increased reproduction rate. While motility grants tangible advantage for the nutrient searching behavior and, therefore, has an impact on fitness, there are also energetic costs of migration, energy expenditure associated with active motility that could be invested into reproduction instead. Moreover, depending on availability of nutrients and the rate of change of their localization in the environment, the benefit of chemotaxis varies by its contribution into species fitness—variable and nutrient poor environments actualize this advantage, nutrient rich and stagnant habitats tend to diminish its adaptive potential. Altogether, it shapes the parameter space of ecological factors, which influence the success of a particular strategy.

It is important to note a number of limitations stemming from the assumptions taken in our model concerning time scale, structure of the environment and genetic variation. First, since the focus of our work is centered around the assessment of the impact of motility on the evolutionary success of a population, we stick to the time scale of generational turnover. On the other hand, fine micro-scale simulation approaches acting on a single cell level are computationally demanding for modelling of microbial populations, which limit the number of simulation scenarios that can be investigated. Whereas, we focused on the population level effects where the differences in local nutrient histories mostly average out to the aggregate dynamics. Evidently, such an approach has its own limitations, especially for the cases when small cell numbers matter but since we investigate the population processes, we believe that we stay within its constraints. Second, as we mentioned we consider simplified environment, yet spatially structured and dynamic, in order to draw the baseline and to understand the mechanics of Settler-Nomad competition in such a simple setting. This is a foundation to move towards future development of present model, taking into account various natural environments characterized by more complex nutrient distribution factors, which correspond to widely present in natural aquatic environments organic sources originating from the activity of zoo- and phyto-plankton and other biological macro-objects. Third, we do not cover the aspects associated with genetic variation in the current paper. However, natural selection is able to act upon populations of microorganisms affecting function of genetic machineries that control cell motility and efficient utilization of substrates. Taking into account these factors in more sophisticated models come across as being a prospective direction of further investigations.

That being said, we have shown in this simulation study that even under the above-mentioned assumptions there is the growth-motility trade-off in the system and a potential advantage conferred by a microbial motility is constrained by different factors—both population and environmental ones, especially under the competition with other species. Therefore, one should take into account these factors to conclude whether this potential advantage becomes actually adaptive or not. We also show that there is a certain parameter space where two populations coexist and divide the control under the environment. Though current study elucidates some of the aspects, mechanisms and constraints of competition between different strategies in terms of bacterial motility, the interrelation between adaptive migrations of microorganisms and their fitness, in general case, remains to be a fundamental problem yet to be investigated using both computational and experimental approaches.

## Figures and Tables

**Table 1:** Settler–Nomad model parameter values for the simulation scenarios considered in the study. For the full set of tested combinations of parameters see the Supplementary Materials.

	Values										
Parameters	Nutrient concentration in a batch (M)	1 × 10^−1^	1 × 10^−2^	1 × 10^−3^	1 × 10^−4^	1 × 10^−5^	1 × 10^−6^	1 × 10^−7^	1 × 10^−8^	1 × 10^−9^	1 × 10^−10^
	Migratory costs (%)	0	2	5	10	15	20	25	33.33	50	99.99
	Period (generations)	1	25	50	100	Intermediate values were considered for some cases				500	1000
	Mortality term (categorical)	Quadratic mortality					Linear mortality				

**Table 2:** Pivot table of the outcomes (who is dominant in the system) for various groups of parameter values. The batch period is measured in generations.

Mortality term	Linear	Period 50	Period 1000	Period 1	Period 1000
		Nomad	Nomad	Settler (almost parity with Nomad)	Settler
	Quadratic	period≤127	period≥128	period 1	period 1000
		Nomad	Settler	Settler (Nomad extincts)	Settler (Nomad extincts)
		“Poor” environment (1 × 10^−5^ M nutrient concentration in a batch)		“Rich” environment (1 × 10^−1^ M nutrient concentration in a batch)	
		Nutrient abundance			

**Table 3:** Nomad’s abundance fraction against migration penalty fee in the environments varying by nutrient concentration in a batch from “extremely scarce” to the “rich” ones. The batch period equals 50 generations. The quadratic mortality term is used.

			Migration Penalty Fee (%)								
**Nutrient concentration in a batch (M)**		**0%**	**2%**	**5%**	**10%**	**15%**	**20%**	**25%**	**33.33%**	**50%**	**99.99%**
	**1 × 10^−1^**	0%	0%	0%	0%	0%	0%	0%	0%	0%	0%
	**1 × 10^−2^**	0%	0%	0%	0%	0%	0%	0%	0%	0%	0%
	**1 × 10^−3^**	66%	0%	0%	0%	0%	0%	0%	0%	0%	0%
	**1 × 10^−4^**	99%	0%	0%	0%	0%	0%	0%	0%	0%	0%
	**1 × 10^−5^**	83%	56%	1%	1%	1%	1%	1%	1%	1%	1%
	**1 × 10^−6^**	41%	67%	65%	11%	11%	11%	11%	11%	11%	11%
	**1 × 10^−7^**	14%	50%	50%	50%	50%	50%	50%	50%	50%	50%
	**1 × 10^−8^**	61%	50%	50%	50%	50%	50%	50%	50%	50%	50%
	**1 × 10^−9^**	77%	50%	50%	50%	50%	50%	50%	50%	50%	50%
	**1 × 10^−10^**	78%	50%	50%	50%	50%	50%	50%	50%	50%	50%

**Table 4:** Nomad’s abundance fraction against migration penalty fee in the environments varying by nutrient concentration in a batch from “extremely scarce” to the “rich” ones. The batch period equals to 50 generations. The linear mortality term is used.

			Migration Penalty Fee (%)								
		**0%**	**2%**	**5%**	**10%**	**15%**	**20%**	**25%**	**33.33%**	**50%**	**99.99%**
**Nutrient concentration in a batch (M)**	**1 × 10^−1^**	49%	0%	0%	0%	0%	0%	0%	0%	0%	0%
	**1 × 10^−2^**	49%	0%	0%	0%	0%	0%	0%	0%	0%	0%
	**1 × 10^−3^**	50%	0%	0%	0%	0%	0%	0%	0%	0%	0%
	**1 × 10^−4^**	52%	51%	1%	1%	1%	1%	1%	1%	1%	1%
	**1 × 10^−5^**	99%	65%	65%	64%	6%	6%	6%	6%	6%	6%
	**1 × 10^−6^**	96%	81%	80%	20%	20%	20%	20%	20%	20%	20%
	**1 × 10^−7^**	61%	50%	50%	50%	50%	50%	50%	50%	50%	50%
	**1 × 10^−8^**	71%	50%	50%	50%	50%	50%	50%	50%	50%	50%
	**1 × 10^−9^**	72%	50%	50%	50%	50%	50%	50%	50%	50%	50%
	**1 × 10^−10^**	72%	50%	50%	50%	50%	50%	50%	50%	50%	50%

**Table 5:** Pivot table of outcomes (who is dominant in the system) for various groups of parameter values. The results do not differ for either quadratic and linear mortality except for the cases marked with an asterisk (*) where under linear mortality the following outcome is observed: Settler/Nomad (full parity). Batch period is measured in generations.

**Migration Penalty Fee**	**Fee Value ≤ 0.3%**	**Period 50**	**Period 1000**	**Period 1**	**Period 1000**
		Nomad	Nomad	Settler *	Settler
	**Fee Value ≥ 0.4%**	**Period 50**	**Period 1000**	**Period 1**	**Period 1000**
		Settler/Nomad (Full Parity).	Settler/Nomad (Full Parity).	Settler	Settler
		**“Extremely Scarce” Environment**		**“Rich” Environment**	
		**Nutrient Abundance**			

## Data Availability

The data presented in this study are available in the Supplementary Materials. The HEC software used for generating the simulation results is available at https://evol-constructor.bionet.nsc.ru/?page_id=34&lang=enpage (accessed on 8 October 2021).

## Supplementary Materials

The following are available online at https://www.mdpi.com/article/10.3390/biology10101019/s1: Figure S1: An example of “abiotic desert” emerging around the “start” nutrient source occupied by Settler population, Table S1: Settler–Nomad model state variables, Table S2: All the parameters of the Settler–Nomad model, Document S1: “Haploid evolutionary constructor” modelling approach, and additional materials.
