# Supplementary material for: Leave or Stay: Simulating Motility and Fitness of Microorganisms in Dynamic Aquatic Ecosystems
Source: Biology (Basel). 2021 Oct 9;10(10):1019. doi: 10.3390/biology10101019 (PMC8533222; doi:10.3390/biology10101019)
Supplement: Supplementary file 1 [file biology-10-01019-s001.zip › 2021.09.07.Document S1.Model supplementary.pdf]

“Haploid e volutionary constructor” modelling approach

1 Simulation of spatially distributed habitats

The new version of the “Haploid evolutionary constructor” (HEC) software implements an extension of the methodology developed for only 0D (well-mixed) environments [1]. In the 0D case, the habitat had just one spatial parameter *volume*, and no others. Cells and substances assumed to be uniformly distributed in the environment. Let us call 0D environments as “nodes”. Spatially distributed (1D, 2D or 3D) environment may be considered as a set of such nodes. Calculation of new states for each node (which is independent and can be performed simultaneously) includes simulation of the following processes: consumption of nutrients (substrates), utilization of nutrients, reproduction, substrates synthesis and secretion. This stage is apparently inherited from the former HEC 0D version. The additional simulation stage is redistribution of substrates and cells in the whole environment.

Central point of the HEC extension is the simulation of the processes of transport of substances and cells between nodes. Two processes account for transport of substances: diffusion and flow. For illustrative purposes, let us consider examples for 2-dimensional case. Processes in 1D and 3D are described similarly by changing dimension of vectors and connectivity of nodes. Let the habitat be described by a set of matrices $E_t^s = \begin{pmatrix} e_{11} & \cdots & e_{1n} \\ \vdots & \ddots & \vdots \\ e_{m1} & \cdots & e_{mn} \end{pmatrix}$, where e_{ij} is amount of substances **s** in **(i,j)** node at iteration **t**. In order to determine spatial distribution of the substance at next step, i.e. to calculate E_{t+1}^s, we need to take into account flow and diffusion. We assume the matrix to be put in the following way: $E_{t+1}^s = E_t^s + F(E_t^s) + D(E_t^s)$, where $F(E_t^s)$ and $D(E_t^s)$ are state-transition matrices for substances (their elements might be positive and negative) in **(i,j)** node for flow and diffusion, correspondingly. Besides the flow and passive transport acting similar to diffusion, transport of cells is determined by chemotaxis (see details below).

At each iteration step, movement of cells and nutrients occurs between four neighbour nodes for 2D case for each node (2 neighbours for 1D, 6 neighbours for 3D). Therefore, the move through **n** nodes in this approach requires **n** iterations. One- and three-dimensional cases are formalized similarly. In the latter case, the $E_t^s = (e_{ijk})$ matrices are three-dimensional, and the nodes are hexalaterally connected.

1.1 Simulation of the flow

The flow is described by a vector of corresponding size comprised by the flow intensity coefficients. Vector components determine portions of cells and substances moved by the flow in each direction. By this means, a user can set the direction of flow and its intensity. Let us consider the flow vector $\overrightarrow{fl} = \begin{pmatrix} fl_x \\ fl_y \end{pmatrix}$ in Cartesian axes (in 1D and 3D cases vectors consist of one and three components, correspondingly). The meaning of $\overrightarrow{fl}$ is the portion of substance removed from the node by the flow per iteration additionally normalized to obtain $fl_x + fl_y + 4k_d = 1$, where k_d is normalized diffusion coefficient. It is obvious that $|fl_x| + |fl_y| \leq 1$ because the amount of removed substance can not exceed the total amount. Let us denote $\overrightarrow{fl_{\text{abs}}} = \begin{pmatrix} |fl_x| \\ |fl_y| \end{pmatrix}$. $\vec{\imath} = (1,0)$ and $\vec{\jmath} = (0,1)$ are the basis vectors. Let us define the matrix element $F(E_t^s) = (f_{ij})$ as a dot product of two vectors: $f_{ij} = \overrightarrow{fl_{\text{abs}}} \cdot (c_x - e_{ij}, c_y - e_{ij})$, where

$$c_x = \begin{cases} e_{i,j-1}, \text{if } \overrightarrow{fl} \cdot \vec{\imath} \geq 0, \\ e_{i,j+1}, \text{otherwise} \end{cases} \quad c_y = \begin{cases} e_{i-1,j}, \text{ if } \overrightarrow{fl} \cdot \vec{\jmath} \geq 0, \\ e_{i+1,j}, \text{otherwise} \end{cases}$$

1.2 Simulation of the diffusion

Diffusion is the process of indirect propagation of substance conditioned by random molecular motion in the environment. In this study, the diffusion coefficient describes the portion of substance diffusing out of the current node. Change of substance amount resulted from diffusion is equal to the difference between influx amount of substance come from neighbour nodes and efflux amount of substance moved to neighbour nodes. Thus, the diffusion is conditioned by

difference in concentrations in neighbour nodes. To take into account the diffusion, let us consider the matrix $D(E_t^s) = (d_{ij})$, where $d_{ij} = k_d \cdot (e_{i,j-1} + e_{i,j+1} + e_{i-1,j} + e_{i+1,j} - 4e_{ij})$, k_d is the normalized diffusion coefficient, i.e. the portion of substance diffusing from the current node into one of four neighbour nodes (in 2D case). Note, that we consider quadruply connected nodes (4-cross neighbourhood) and cover all in- and out-flows. The passive transport of cells caused by cell shoving is described in the same manner as diffusion.

1.3 Simulation of the chemotaxis

Flow and diffusion involve both microorganisms and substances similarly. However, to model cell motion adequately, it is necessary to take into account the following factors. First, as microbial cells might adhere to a bacterial mat, only limited portion of "free"-floating, plankton, cells might move. Second is the active motion via chemotaxis. In our method, the portion of "free" cells is described by its own coefficient k_{free}, and chemotaxis is described by its own term. Similarly to the intensity coefficients of diffusion and flow, the fraction of actively moving cells **m** is defined as a population trait. Therefore, $k_a = k_{free} \cdot m \cdot c$ is the portion of a population that is moved via chemotaxis, where c is a normalization coefficient.

Thus, redistribution of cells in the environment is described similar to substances, but with the extended formula: $E_{t+1}^p = E_t^p + F(E_t^p) + D(E_t^p) + A(E_t^p)$, where $A(E_t^p)$ is the state-transition matrix for the population size change in **(i,j)** node resulted from active motion of the **p** population. Each element of this matrix is calculated as follows: $a_{ij} = p_{in} - p_{out}$, where $\mathbf{p_{in}}$ is amount of cells belonging to this population that migrate to the current node from neighbour nodes with less favourable conditions and p_{out} is amount of cells belonging to this population that migrate from the current node to neighbour nodes with more favourable conditions.

At first we estimate the attraction of environmental conditions in neighbour nodes based on the difference between attractants and repellents in the neighbour and the current nodes. The attraction values for all neighbours are calculated as follows: $A_{pq} = \sum(b_k \cdot c_k)$, where $\mathbf{c_k}$ is the concentration of **k**-th substance and

$$b_k = \begin{cases} 1, \text{if } \mathbf{k} - \text{th substance is the attractant for } \mathbf{p}, \\ -1, \text{if } \mathbf{k} - \text{th substance is the repellent for } \mathbf{p}, \\ 0, \text{if } \mathbf{p} \text{ does not consume } \mathbf{k} - \text{th substance.} \end{cases}$$

After that, two lists of neighbours are formed. In the first one, there are repulsive nodes that possess lower attraction values than the current one, and in the second list, there are attractive nodes, which possess higher attraction values. Change of population size in the current node is defined as the sum of the ones who came in (the first list) minus sum of the ones who went out (the second list). Moreover, the portion of actively moving cells is divided by all directions and share of the direction is proportional to the normalized weight coefficient determined by the attraction values of the respective neighbour node. It should be noted that while normalizing values of $\overrightarrow{fl}$ and k_d, k_a is also taken into account.

Finally, $p_{out} = \sum_{q \in N(i,j)} k_a \cdot \frac{A_q}{A_{ij} + \sum_{t \in N(i,j)} A_t} \cdot e_q$, where k_a is the portion of a population that is moved via chemotaxis; **N(i,j)** is a set of neighbours of the current **(i,j)** node; $\mathbf{e_q}$ is abundance (number of cells) of **p**-th population in the **q**-th node, where $q \in N(i,j)$; $\mathbf{A_q}$ is the attraction value of the **q**-th node, $\mathbf{A_{ij}}$ is the attraction value of the current node. And ultimately, $\mathbf{p_{in}}$ is calculated based on $\mathbf{p_{out}}$ values in the neighbour nodes: $p_{in} = \sum_{t \in N(i,j)} w_q \cdot \mathrm{p}_{out}^t$, where $\mathbf{w_q}$ is the portion of $\mathbf{p_{out}}$ from the neighbour node **q** that comes to the current node.

1.4 Spatial redistribution factors and cells' energy reserves

In HEC, cells' energy reserve is represented by the consumed nonspecific substrate molecules per cell in a particular microbial population (see 2.1 for the description of consumption of nutrients). With that, the described above redistribution factors acting on microbial cells affect the current energy reserves in any particular node. These factors can be split into two parts – passive transport (with the flow or undirectedly) and active migration via chemotaxis. Let us denote $\mathbf{r_{pass\_immigr}}$ and $\mathbf{r_{pass\_emigr}}$ the energy reserve income and outcome via passive transport of cells of the population into and out of the current node respectively. Then $r_{pass\_emigr} = \frac{r_n[i,j]}{P_n[i,j]} \cdot P_{pass\_out}$

and $r_{pass\_immigr} = \sum_{q \in N(i,j)} (\frac{r_n^{(q)}}{p(q)} \cdot w_{(q)} \cdot P_{pass\_out}^{(q)})$ where **N(i,j)** is a set of neighbours of the current **(i,j)** node, $\mathbf{P^{(q)}}$ is the population abundance in the neighbour node **q**, $\mathbf{P}_{pass\_out}^{(q)}$ corresponds to the total passive efflux of the cells from a particular node **q** (see paragraph 1.2) and $\mathbf{w_q}$ is the portion of $\mathbf{P}_{pass\_out}^{(q)}$ from the neighbour node **q** that comes to the current node.

Similarly, $\mathbf{r_{act\_immigr}}$ and $\mathbf{r_{act\_emigr}}$ are the energy reserve income and outcome via immigration and emigration of cells of the population into and out of the current node respectively mediated by chemotaxis. Namely, $r_{act\_emigr} = \frac{r_n[i,j]}{P_n[i,j]} \cdot P_{act\_out}$ and $r_{act\_immigr} = \sum_{q \in N(i,j)} (\frac{r_n^{(q)}}{p(q)} \cdot w_{(q)} \cdot P_{act\_out}^{(q)})$ where **N(i,j)** is a set of neighbours of the current **(i,j)** node, $\mathbf{P^{(q)}}$ is the population abundance in the neighbour node **q**, $\mathbf{P}_{act\_out}^{(q)}$ corresponds to the total number of cells that migrated from a particular node **q** (see paragraph 1.3) and $\mathbf{w_q}$ is the portion of $\mathbf{P}_{act\_out}^{(q)}$ from the neighbour node **q** that comes to the current node.

2 Simulation of nutrition and synthesis of nutrients

2.1 Simulation of consumption of nutrients

The processes of secretion and consumption of nutrients are described in Haploid evolutionary constructor based on the following assumptions:

1. Nutrition of bacteria is provided by the transport of nutrients diffusing via the cell wall contacting with the environment (pinotrophy);
2. The surface area to the cell volume ratio matters for the nutrient uptake.

The main idea underlying our submodel of secretion and consumption of nutrients states that there is a volume $V_{consumed}$ flowing through the cell during a certain period of time (one iteration which corresponds to an average cell division time) and this volume is determined by two factors: the area of cell surface and the cell's velocity. $V_{consumed}$ is calculated as follows:

$$V_{consumed}(t) = S_{cell} \cdot v_{cell} \cdot t$$

where S_{cell} is the cell surface area (measured in m^2); v_{cell} is the cell's velocity (measured in m/h); t

is time (measured in h).

Typical value of $V_{consumed}$ for *E.coli* is estimated to be about $5.4 \cdot 10^{-12}$ liters per half an hour with the cell surface area equals to roughly $6 \cdot 10^{-12}$ m^2 and cell's velocity equals to $1.8 \cdot 10^{-11}$ m/h [Sundararaj et al., 2004].

Under the assumption that all the cells have the same $V_{consumed}$ in any well-mixed point-environment (node), the change of nutrient concentration in the particular node (S_{env}) is described in the following way:

$$S_{env}(t+1) = S_{env}(t) \cdot (1 - P\frac{V_{consumed}}{V_{total}})$$

where P is the abundance of the population (number of cells) in the node; V_{total} is the volume of the node. The number of molecules of the nutrient consumed by the cells belonging to the population under consideration (S_{pop}) is calculated as follows:

$$S_{pop}(t+1) = S_{pop}(t) + S_{consumed}(t)$$

where $S_{consumed}(t) = P \cdot V_{consumed} \cdot S_{env}(t) \cdot N_A$ and N_A is Avogadro constant.

If there are too many cells in the node (i.e. the inequality below is true) they can't filter all the $V_{consumed}$ volume.

$$\sum_{i \in I_{POP}} P_i \cdot V_{consumed} > \eta \cdot V_{total}$$

where I_{pop} is the population set and $\eta \leq 1$ is a coefficient that reflects this «overpopulation» effect. If we define $\tilde{V}_{consumed}$ as

$$\tilde{V}_{consumed} = \eta \cdot V_{total} \Big/ \sum_{i \in I_{POP}} P_i$$

it will describe a corrected consumed volume. Then $\tilde{V}_{consumed}$ is used to take into account density effects that result in a decrease of nutrient uptake.

2.2 Simulation of synthesis of specific nutrients

The processes of synthesis of specific nutrients is described by the following equation, which is calculated for each specific nutrient produced by each population in the environment:

$$\Delta S = \left(\sum_{i \in A} av_i \cdot ac_i\right) \cdot cprod \cdot P$$

where ΔS – amount of substance S synthesized by the cells of the population per iteration; A – set of alleles of the gene controlling production of S by the cells of the population; av_i – particular allele value of the gene controlling production of S by the cells of the population; ac_i – allele concentration of av_i in the population; *cprod* – amount of synthesized molecules for one cell per generation; P – population size (abundance, number of cells).

3 Simulation of the population growth

The following term describes the population growth in the the [i,j]-th node:

$$growth(P_n[i,j], r[i,j], N) = P_n[i,j] \cdot (1 + \frac{r[i,j]/K}{1 + r[i,j]/(B \cdot K + N)})$$

where P_n – population abundance on the n-th step; r – number of accumulated nutrient molecules by the cells of the population (population energy reserve); N – genetically determined nutrient utilization efficacy.

This is a hyperbolic curve – both as a function of r and as a function of N.

K is a dimensionless parameter, which affects the coordinates of the inflection point for the curves $growth(N)$ and $growth(r)$. The same parameter determines the range of $growth(N)$ under the fixed set of allele values $N \in \{n_1, \dots, n_m\}$. The lesser is K, the greater is the range, i.e. the difference between the effect on the growth of n_{min} and n_{max} allele values.

B is a dimensionless parameter, which determines the y-value of the intersection point of the curve $growth(N)$ with the line N=0. The greater is B, the higher is the intersection point (under the fixed set of allele values). In other words, this parameter controls the value of basal amount of growth. If we examine the hyperbolic $growth(r)$ under the fixed N, we see that the greater is B, the more amount of nutrient molecules per cell (r) is needed to attain the same level of population growth.

4 References

1. Lashin, S. A.; Matushkin, Y. G. Haploid Evolutionary Constructor: New Features and Further Challenges. *In Silico Biol.* **2011**, *11* (3), 125–135. https://doi.org/10.3233/ISB-2012-0447.
