# Supplementary figures and images for: Leave or Stay: Simulating Motility and Fitness of Microorganisms in Dynamic Aquatic Ecosystems (part 2 of 2)
Source: Biology (Basel). 2021 Oct 9;10(10):1019. doi: 10.3390/biology10101019 (PMC8533222; doi:10.3390/biology10101019)

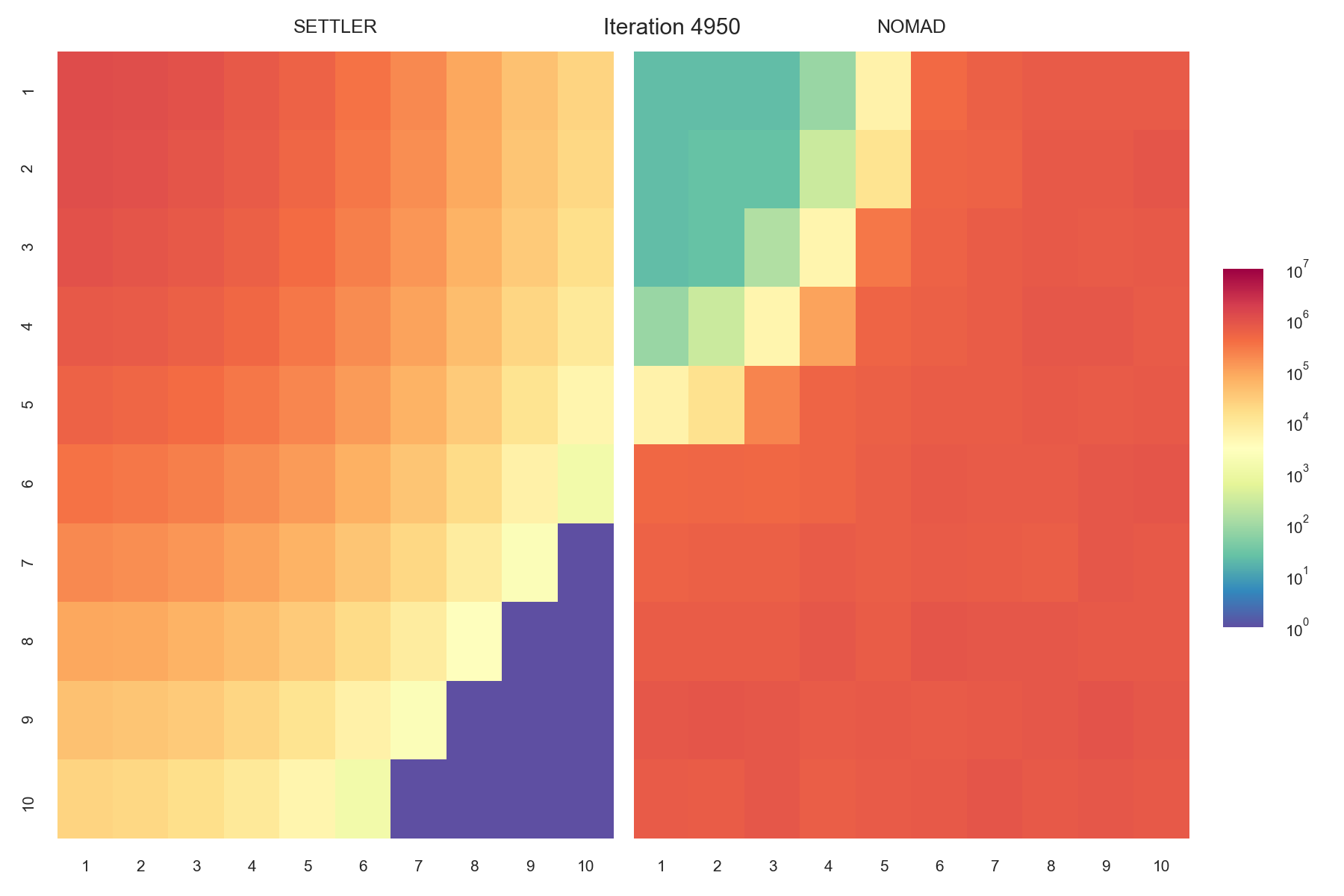

Supplement: Supplementary file 1 [file biology-10-01019-s001.zip › Spatio-temporal dynamics heatmaps/chempenoff_extremelyscarce_lindeath_period1000/4950.png]

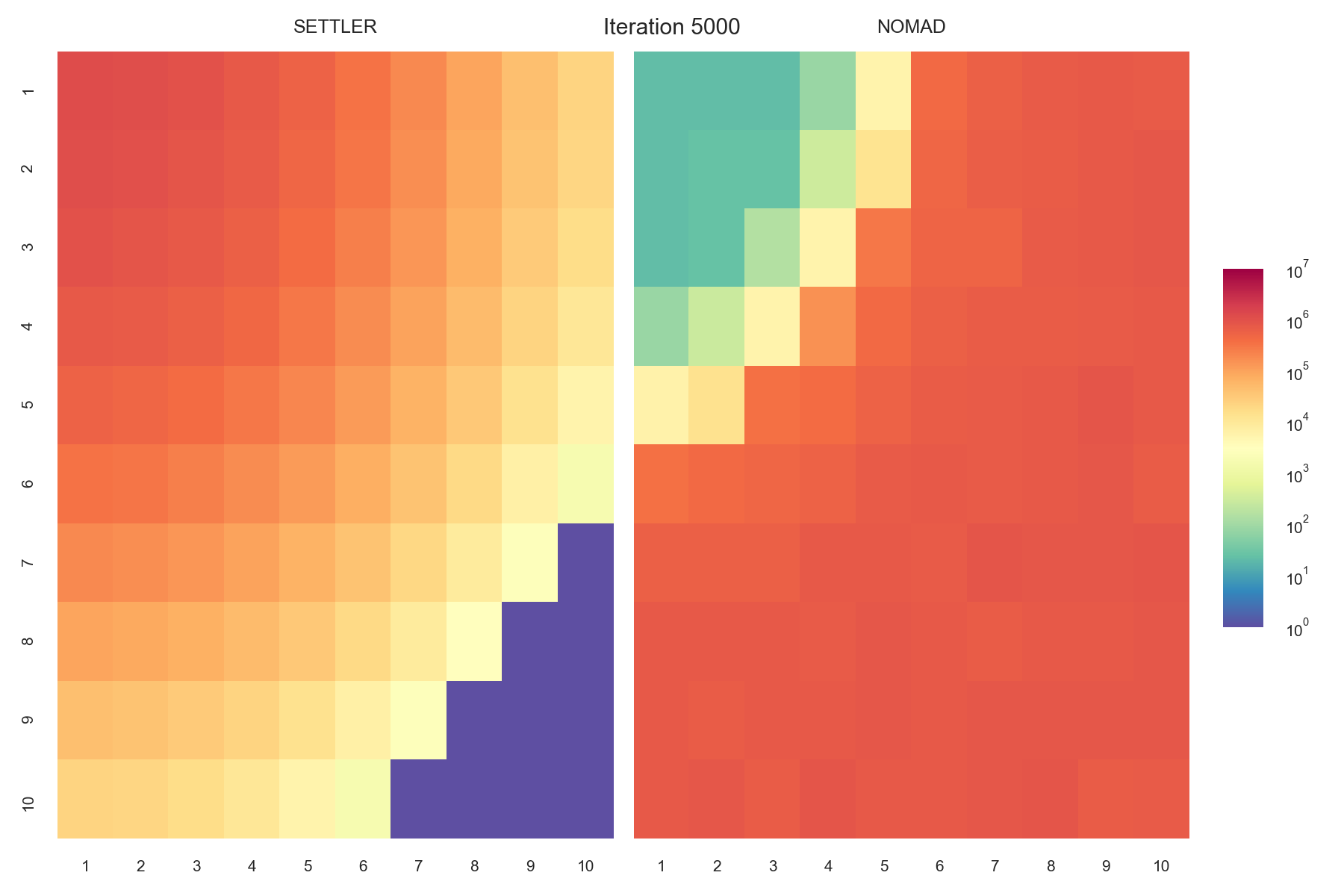

Supplement: Supplementary file 1 [file biology-10-01019-s001.zip › Spatio-temporal dynamics heatmaps/chempenoff_extremelyscarce_lindeath_period1000/5000.png]

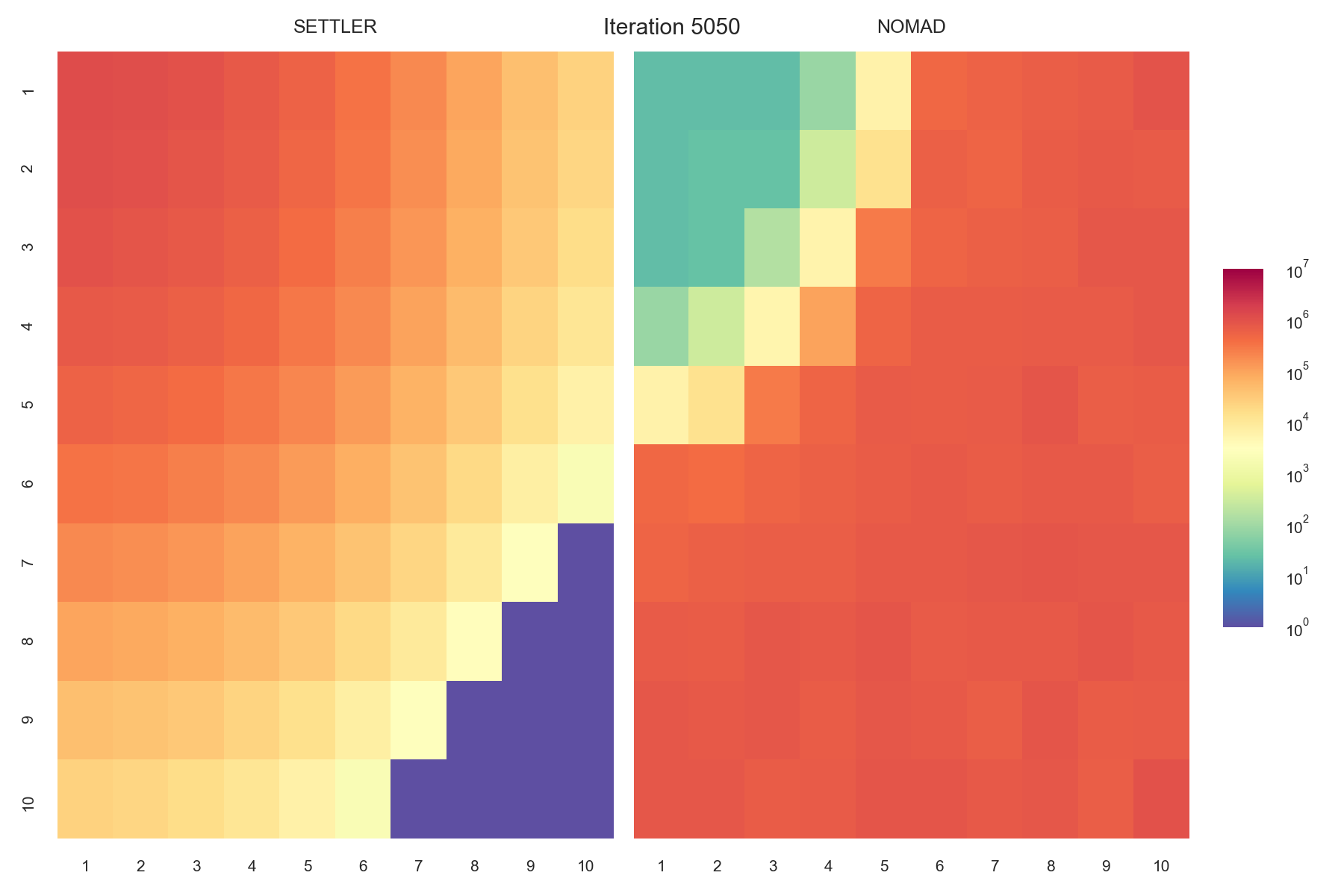

Supplement: Supplementary file 1 [file biology-10-01019-s001.zip › Spatio-temporal dynamics heatmaps/chempenoff_extremelyscarce_lindeath_period1000/5050.png]

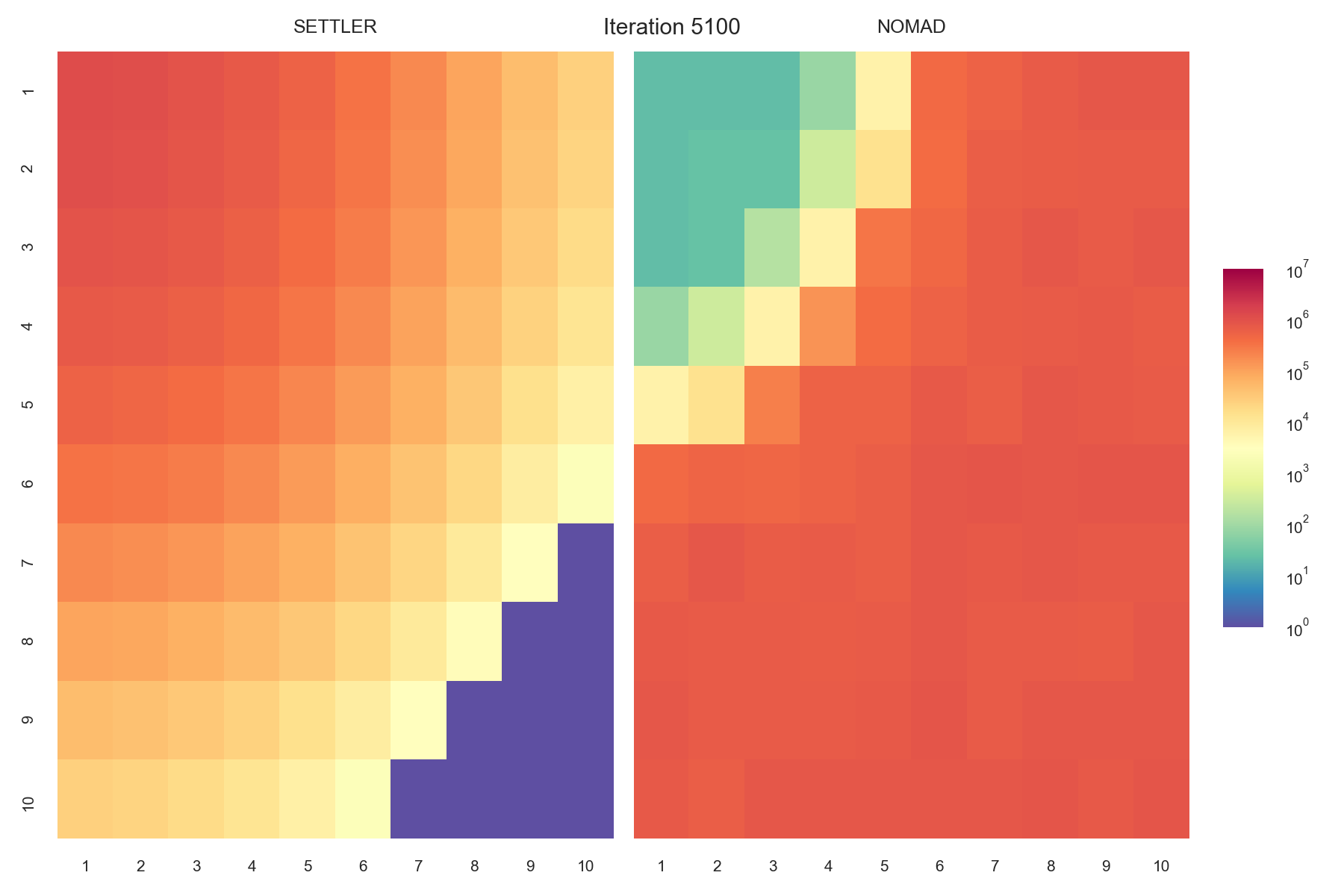

Supplement: Supplementary file 1 [file biology-10-01019-s001.zip › Spatio-temporal dynamics heatmaps/chempenoff_extremelyscarce_lindeath_period1000/5100.png]

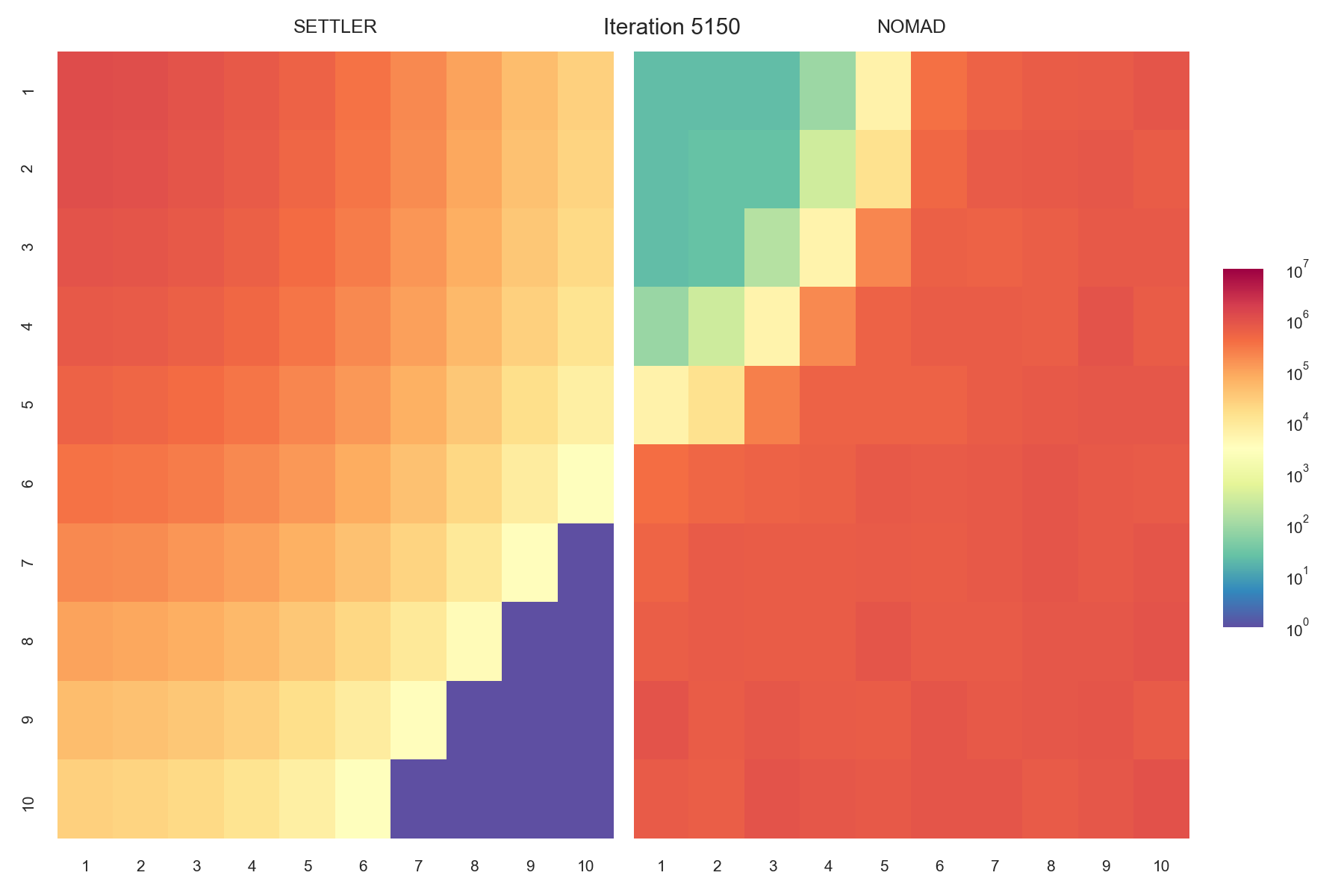

Supplement: Supplementary file 1 [file biology-10-01019-s001.zip › Spatio-temporal dynamics heatmaps/chempenoff_extremelyscarce_lindeath_period1000/5150.png]

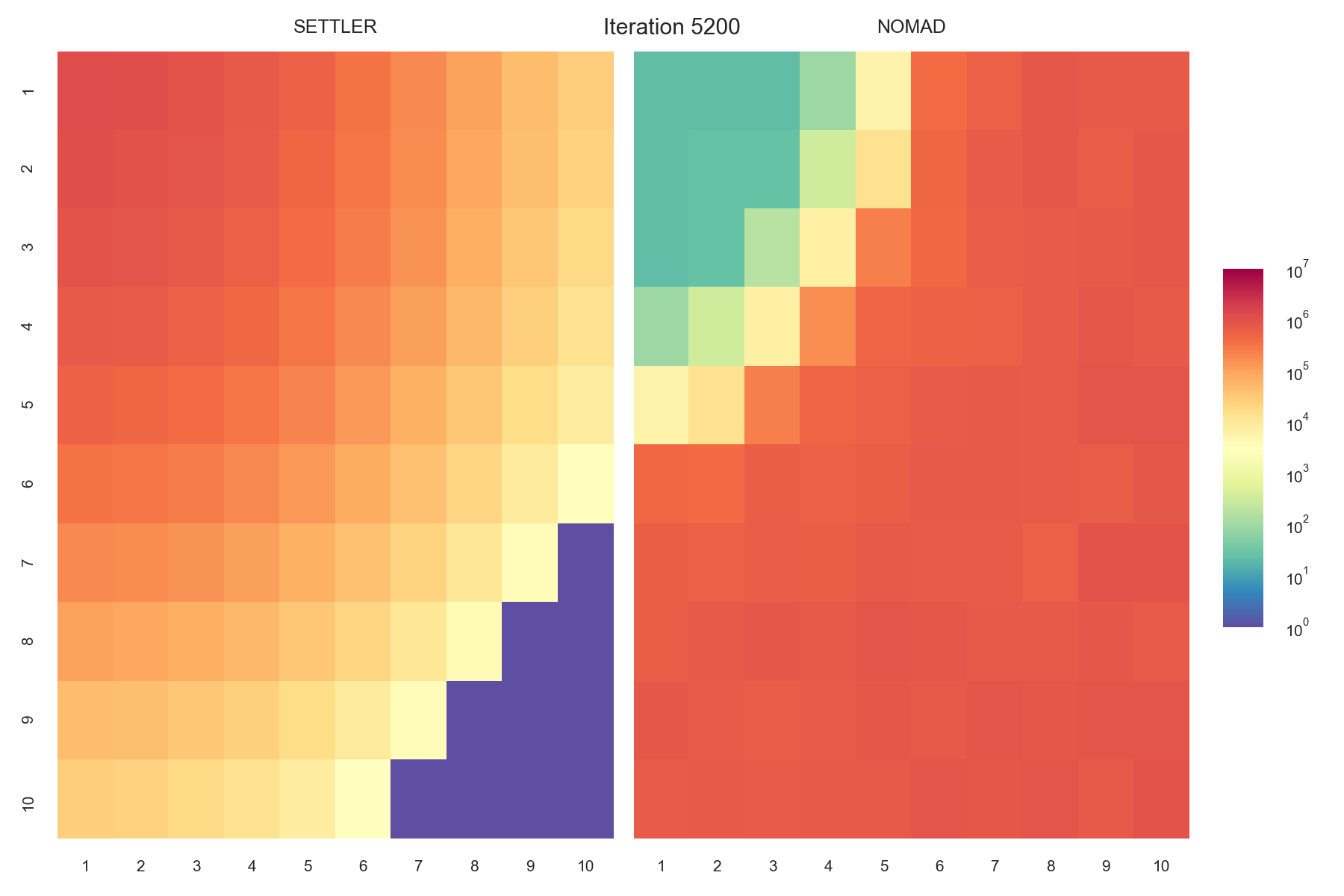

Supplement: Supplementary file 1 [file biology-10-01019-s001.zip › Spatio-temporal dynamics heatmaps/chempenoff_extremelyscarce_lindeath_period1000/5200.png]

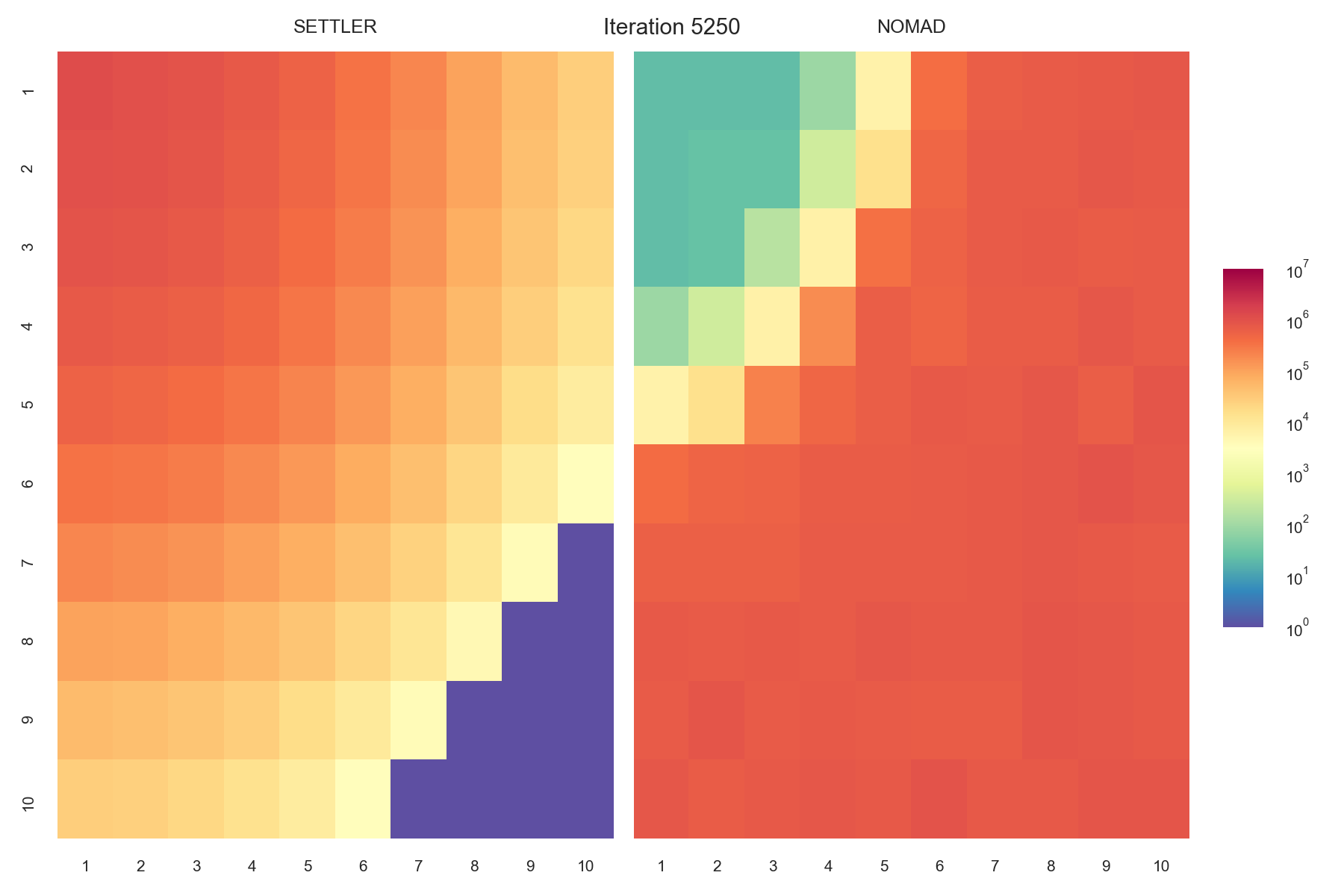

Supplement: Supplementary file 1 [file biology-10-01019-s001.zip › Spatio-temporal dynamics heatmaps/chempenoff_extremelyscarce_lindeath_period1000/5250.png]

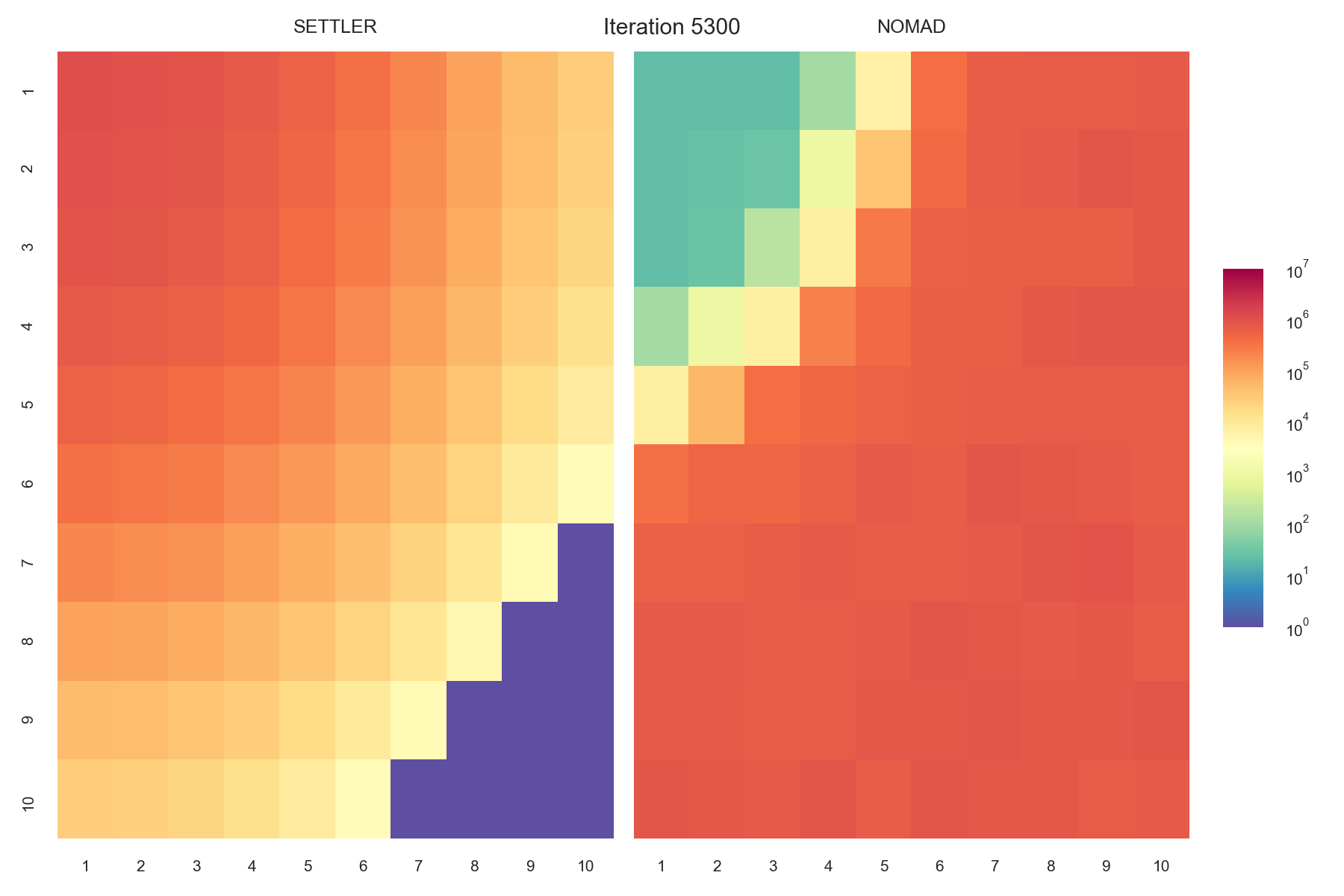

Supplement: Supplementary file 1 [file biology-10-01019-s001.zip › Spatio-temporal dynamics heatmaps/chempenoff_extremelyscarce_lindeath_period1000/5300.png]

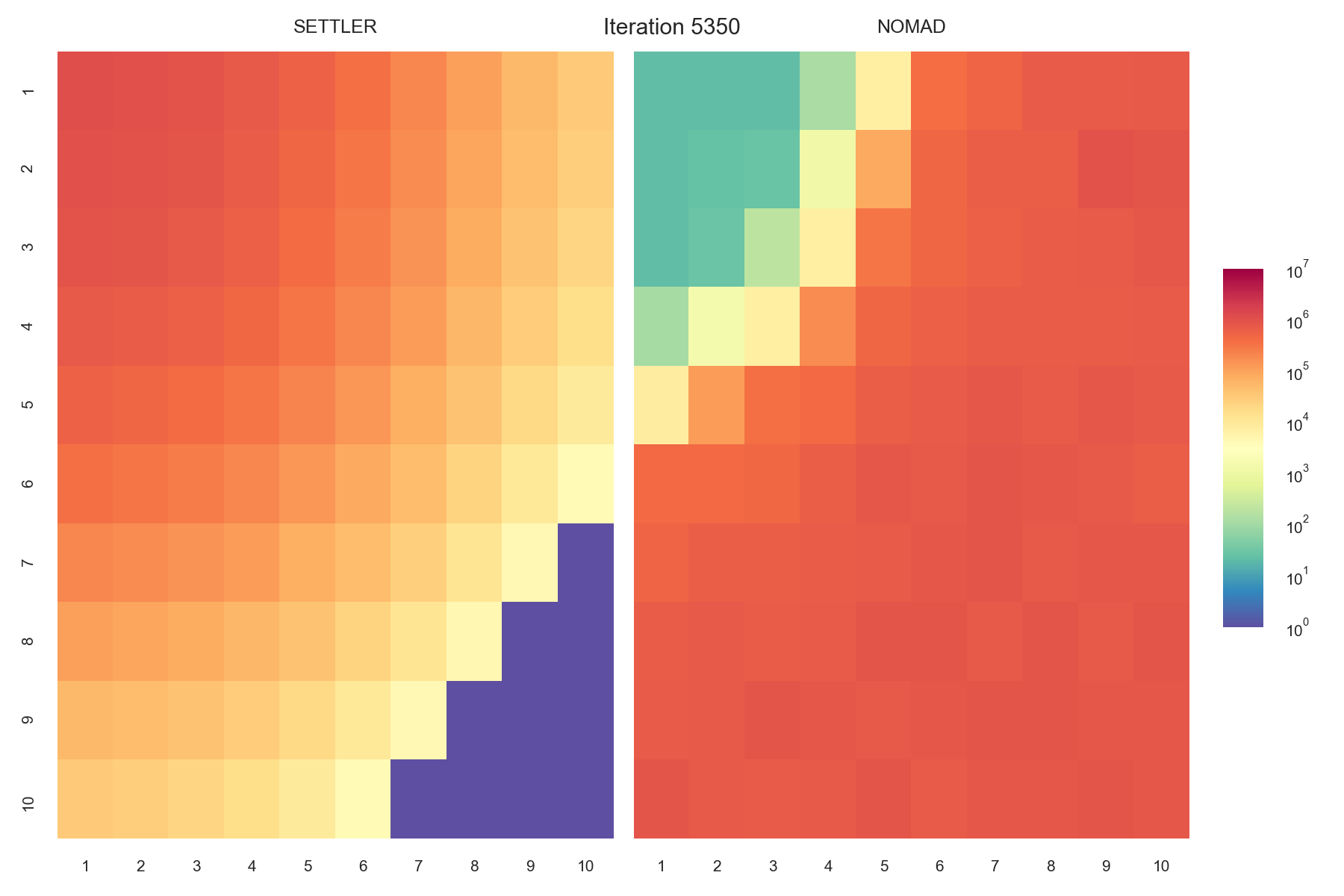

Supplement: Supplementary file 1 [file biology-10-01019-s001.zip › Spatio-temporal dynamics heatmaps/chempenoff_extremelyscarce_lindeath_period1000/5350.png]

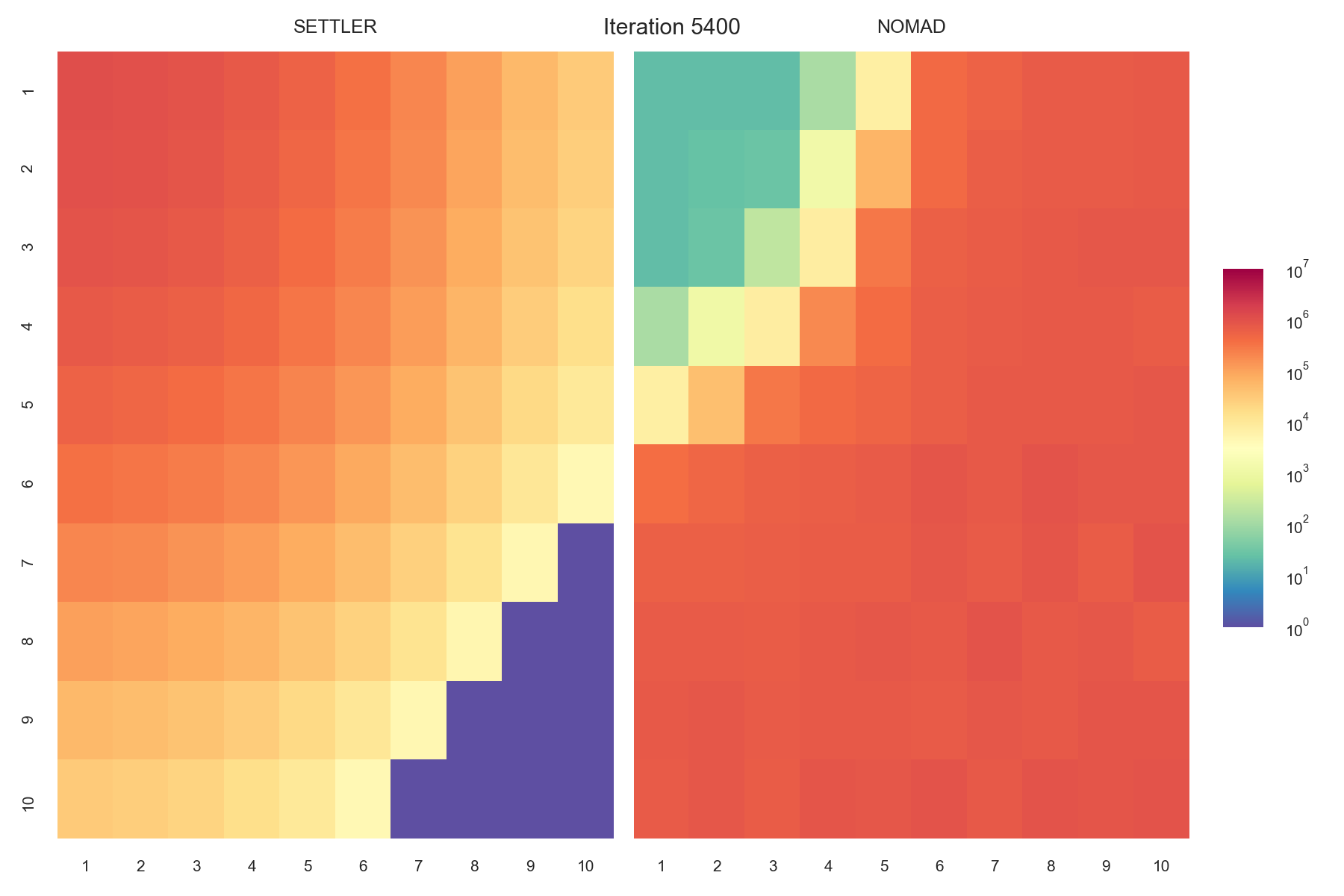

Supplement: Supplementary file 1 [file biology-10-01019-s001.zip › Spatio-temporal dynamics heatmaps/chempenoff_extremelyscarce_lindeath_period1000/5400.png]

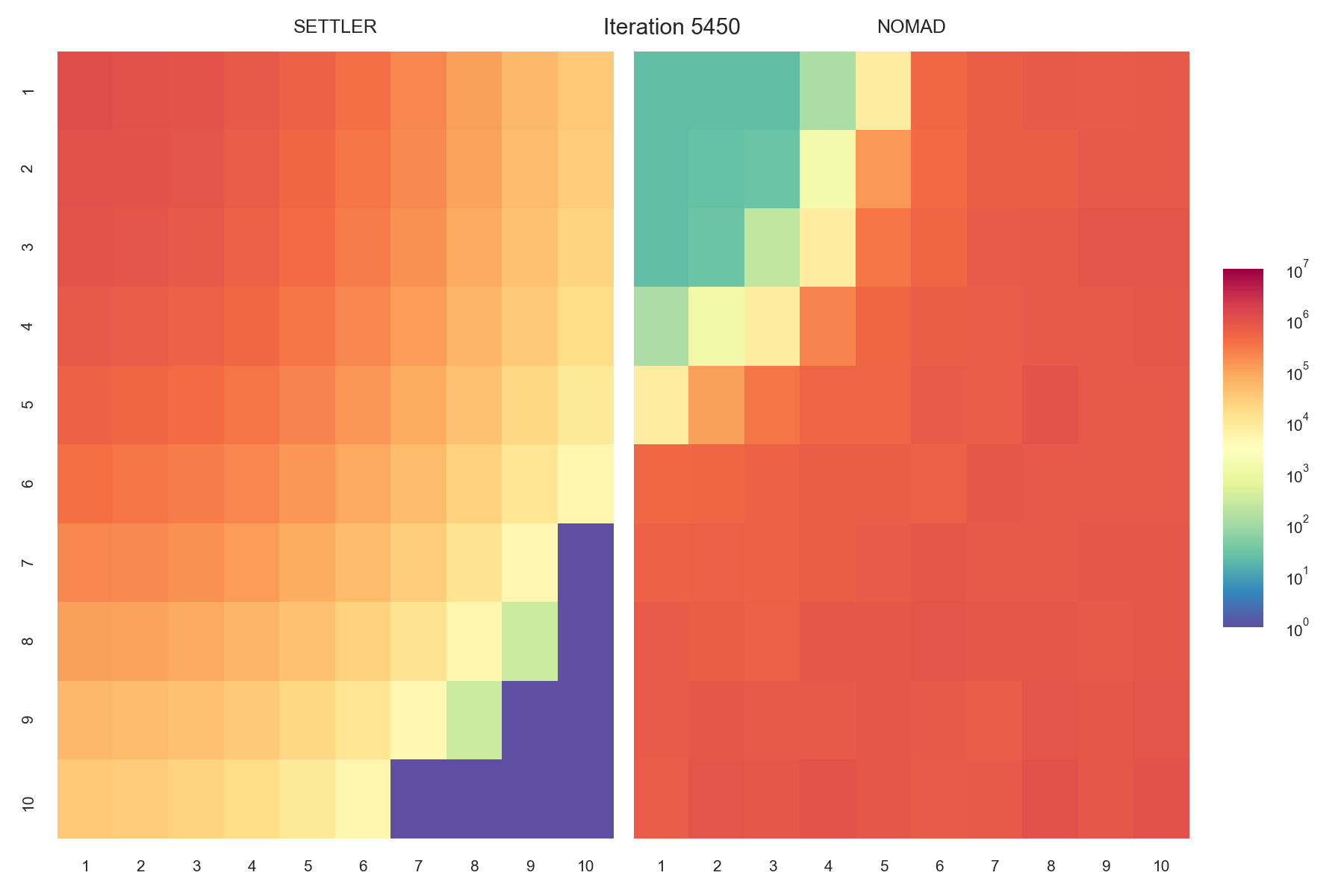

Supplement: Supplementary file 1 [file biology-10-01019-s001.zip › Spatio-temporal dynamics heatmaps/chempenoff_extremelyscarce_lindeath_period1000/5450.png]

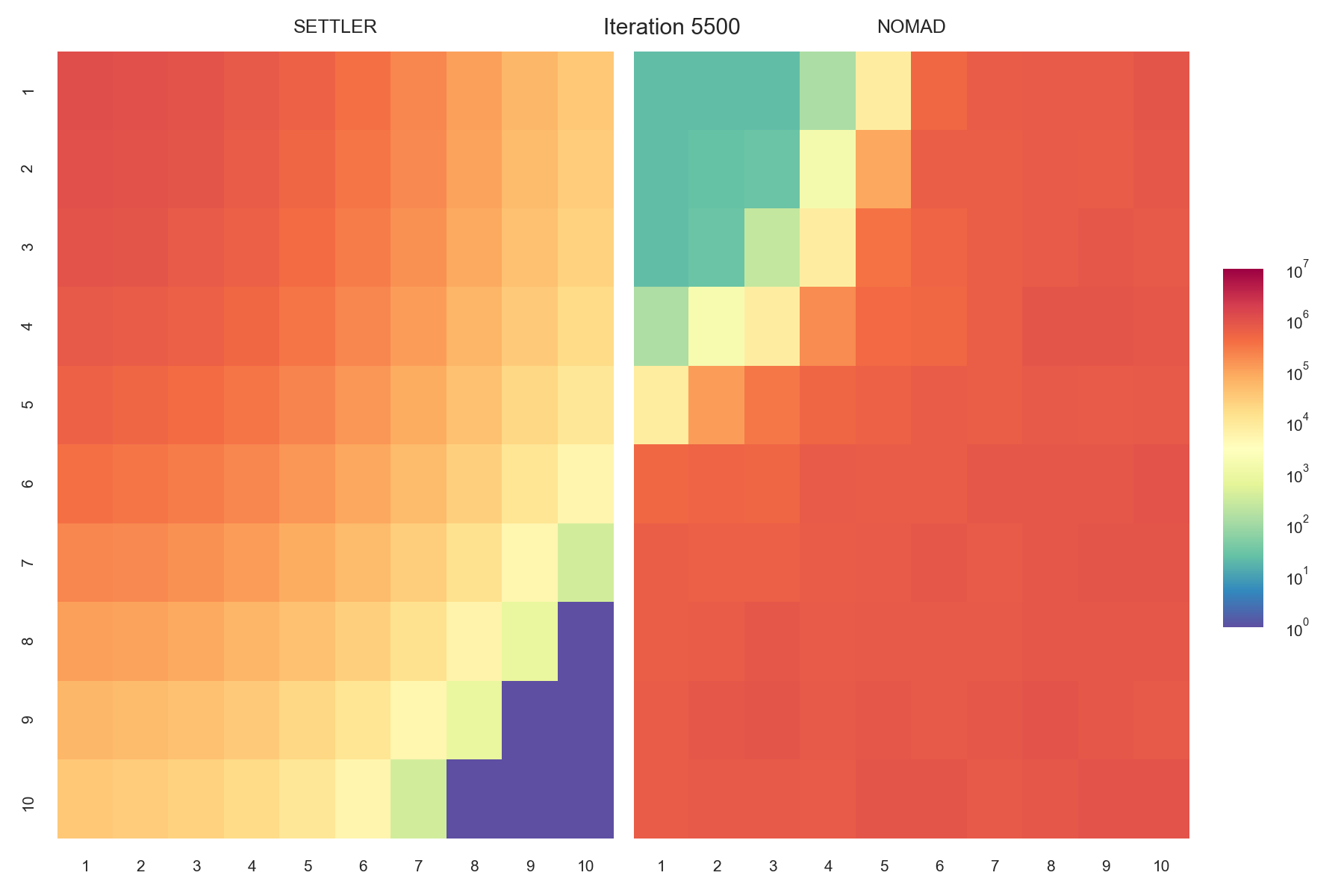

Supplement: Supplementary file 1 [file biology-10-01019-s001.zip › Spatio-temporal dynamics heatmaps/chempenoff_extremelyscarce_lindeath_period1000/5500.png]

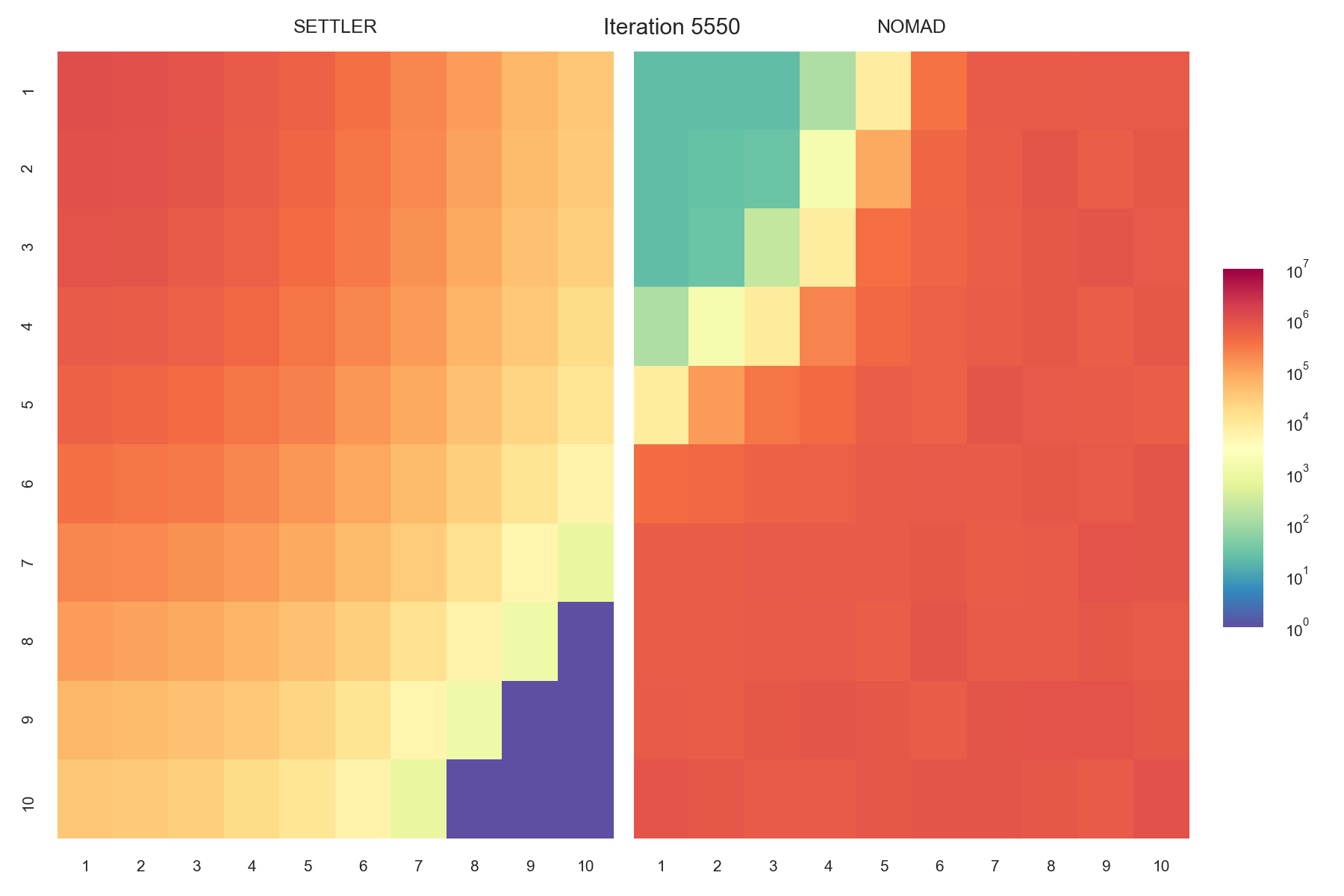

Supplement: Supplementary file 1 [file biology-10-01019-s001.zip › Spatio-temporal dynamics heatmaps/chempenoff_extremelyscarce_lindeath_period1000/5550.png]

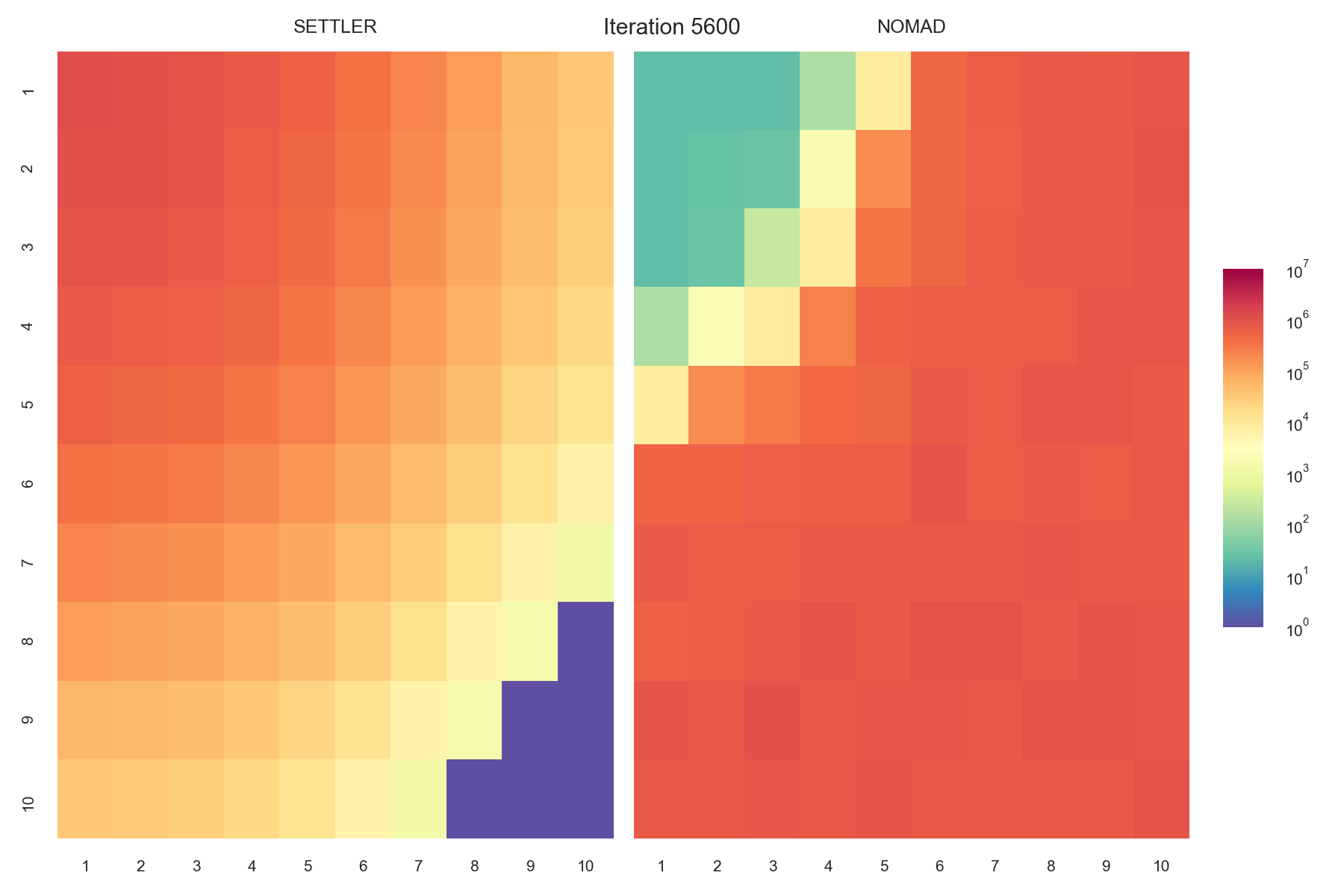

Supplement: Supplementary file 1 [file biology-10-01019-s001.zip › Spatio-temporal dynamics heatmaps/chempenoff_extremelyscarce_lindeath_period1000/5600.png]

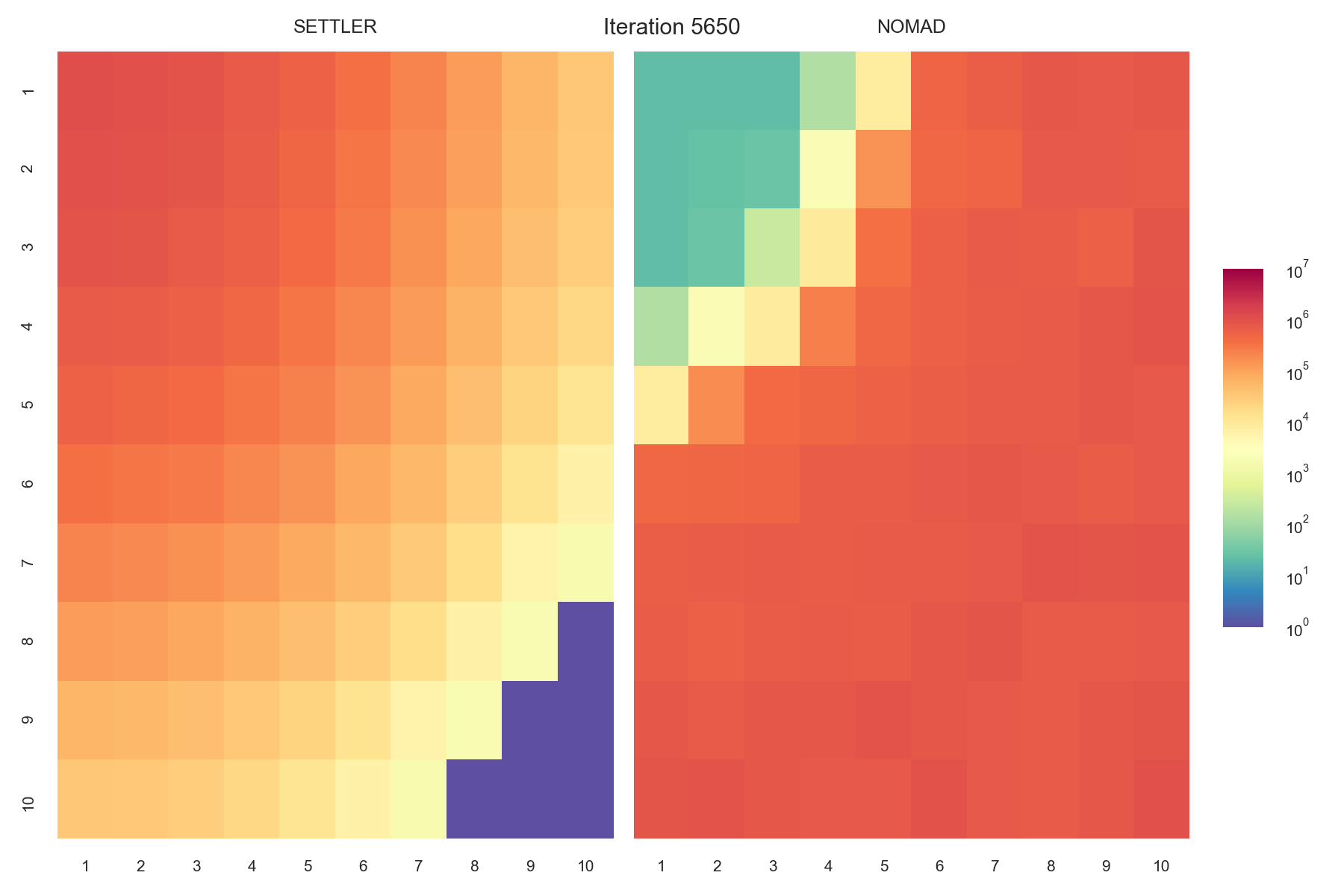

Supplement: Supplementary file 1 [file biology-10-01019-s001.zip › Spatio-temporal dynamics heatmaps/chempenoff_extremelyscarce_lindeath_period1000/5650.png]

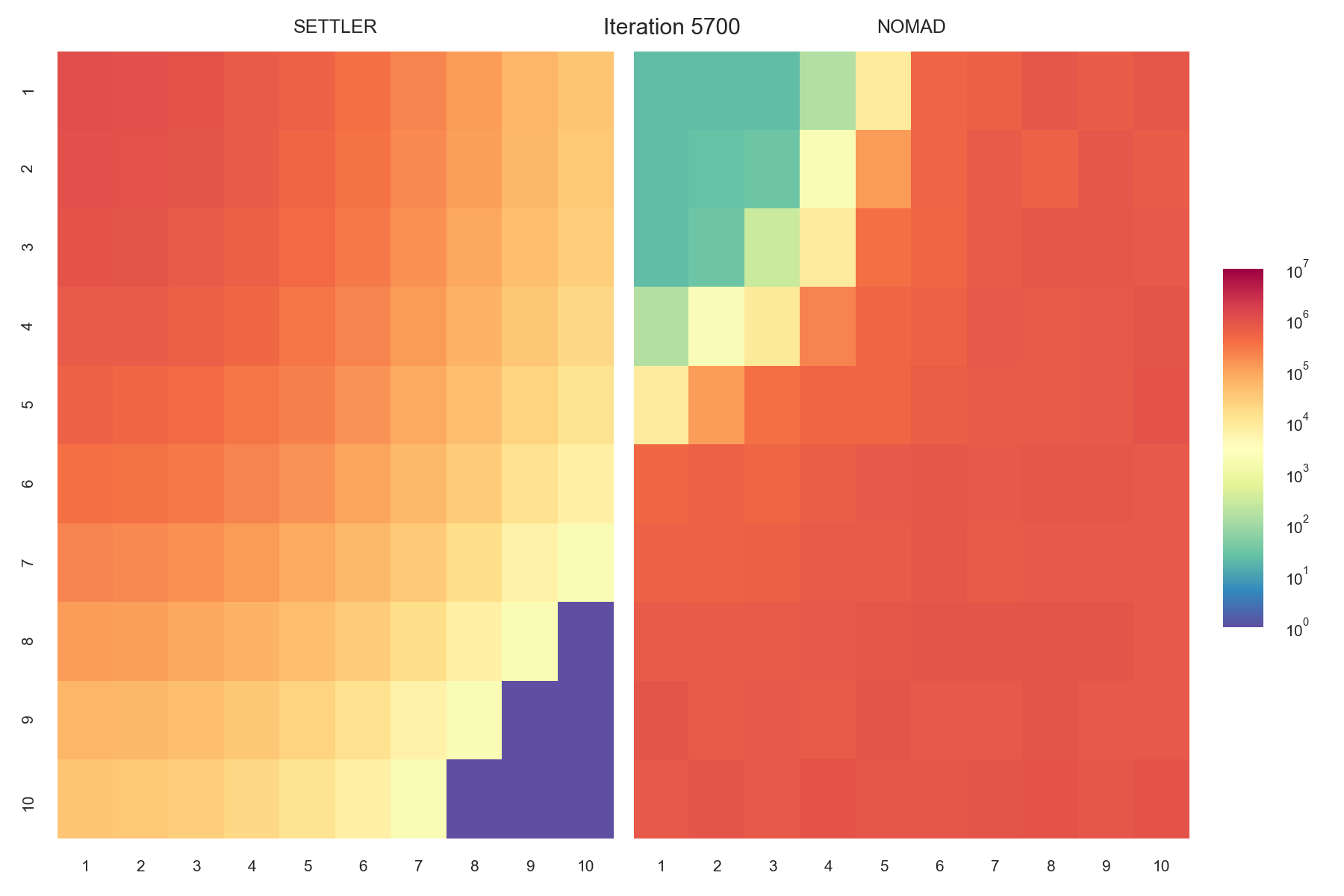

Supplement: Supplementary file 1 [file biology-10-01019-s001.zip › Spatio-temporal dynamics heatmaps/chempenoff_extremelyscarce_lindeath_period1000/5700.png]

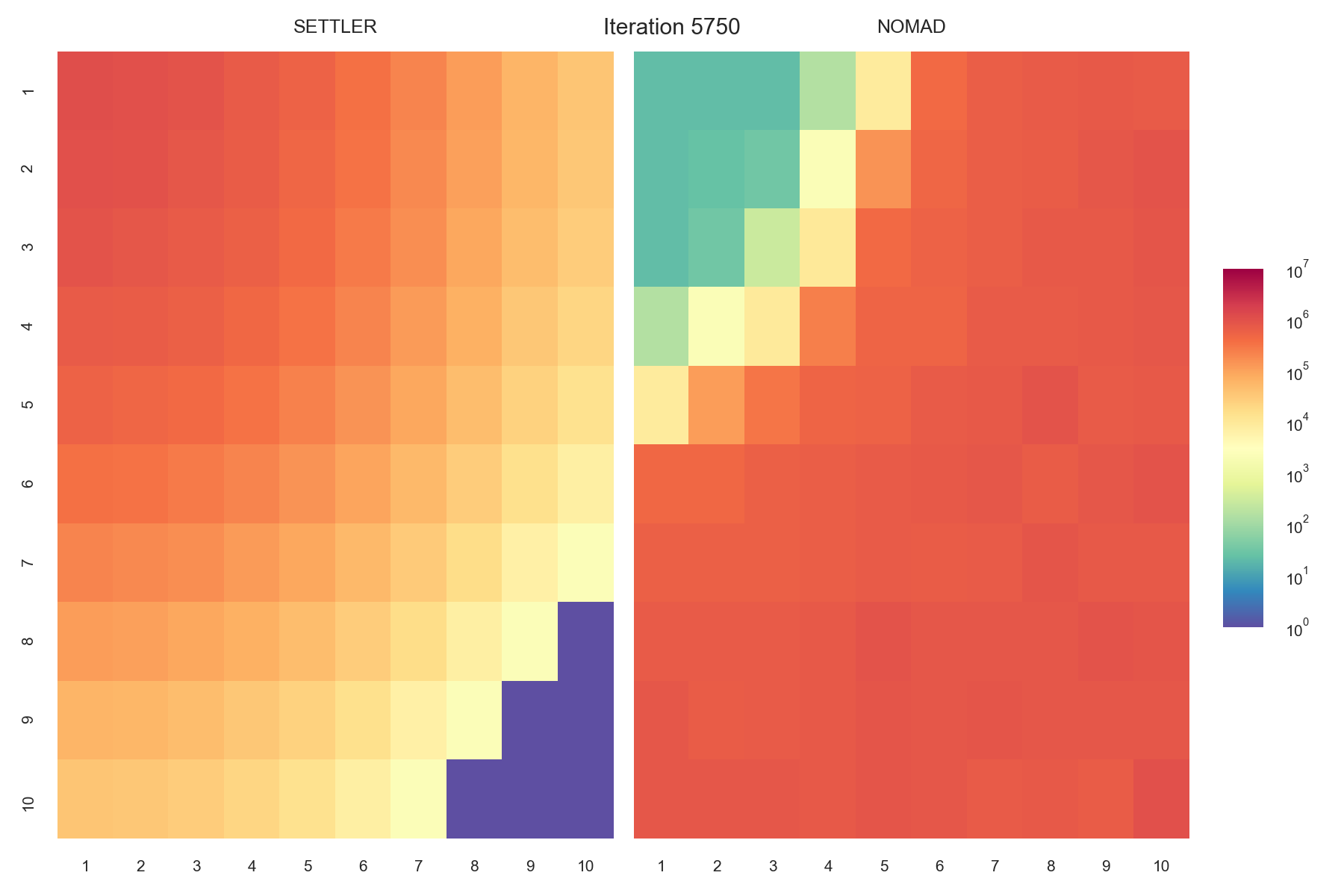

Supplement: Supplementary file 1 [file biology-10-01019-s001.zip › Spatio-temporal dynamics heatmaps/chempenoff_extremelyscarce_lindeath_period1000/5750.png]

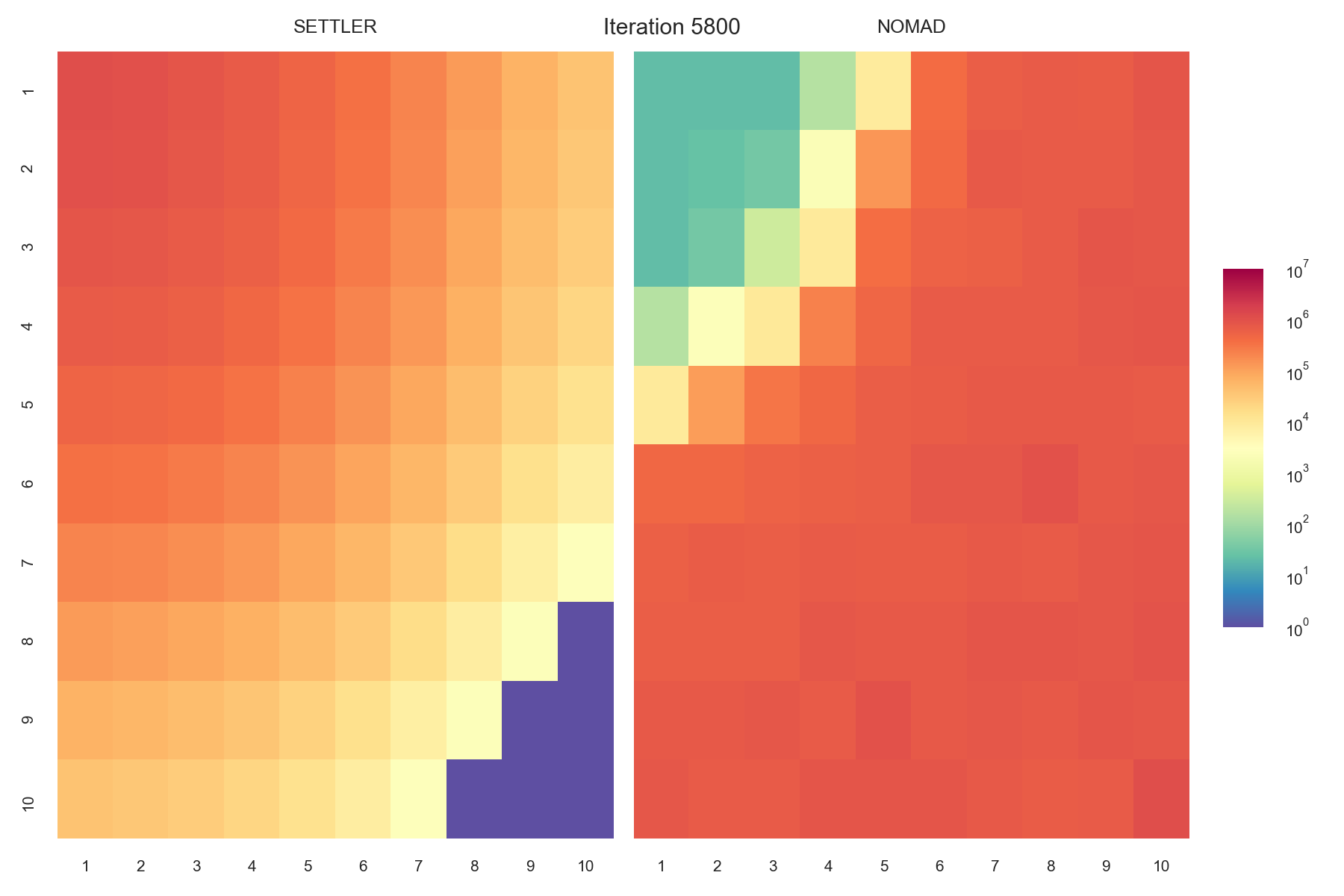

Supplement: Supplementary file 1 [file biology-10-01019-s001.zip › Spatio-temporal dynamics heatmaps/chempenoff_extremelyscarce_lindeath_period1000/5800.png]

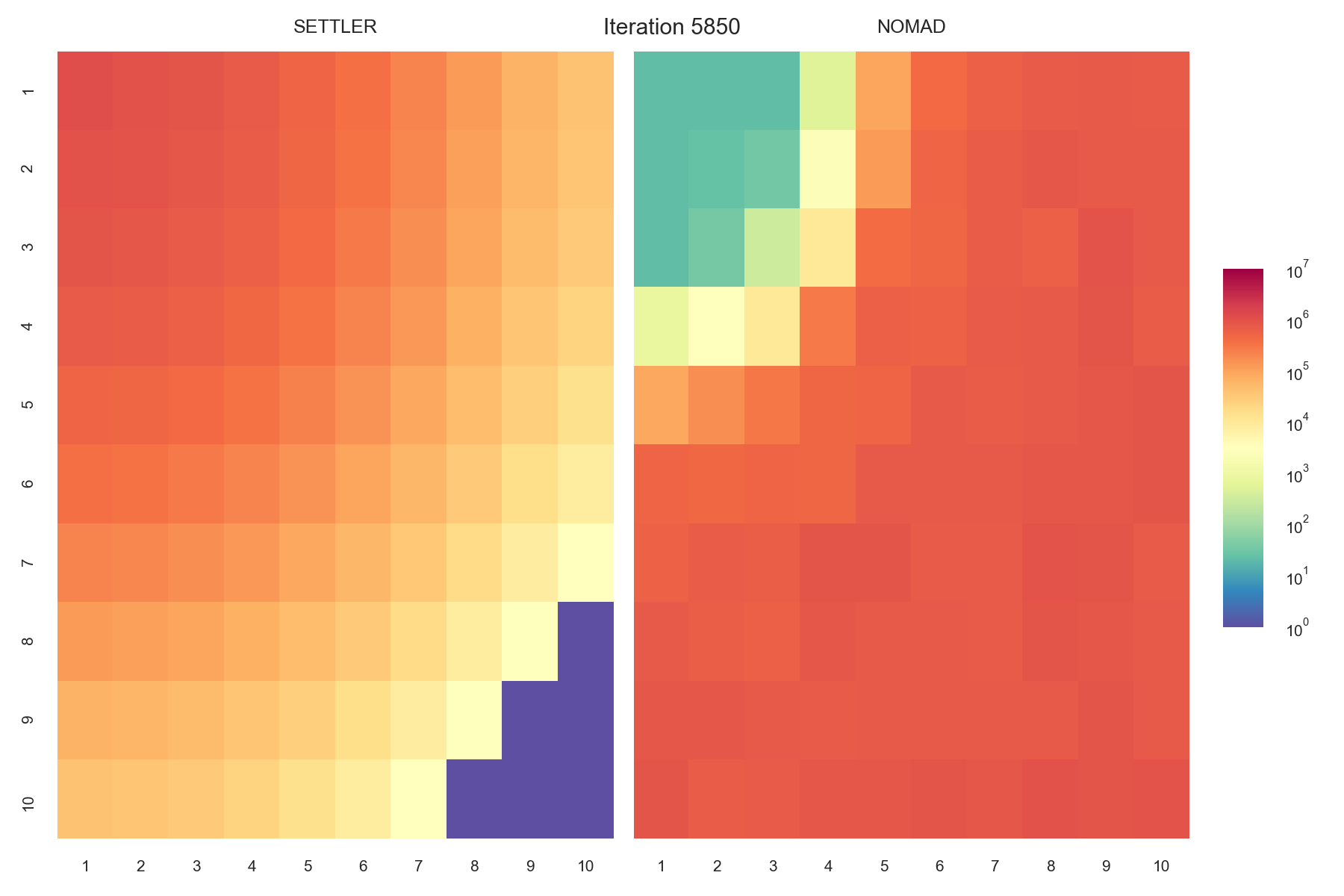

Supplement: Supplementary file 1 [file biology-10-01019-s001.zip › Spatio-temporal dynamics heatmaps/chempenoff_extremelyscarce_lindeath_period1000/5850.png]

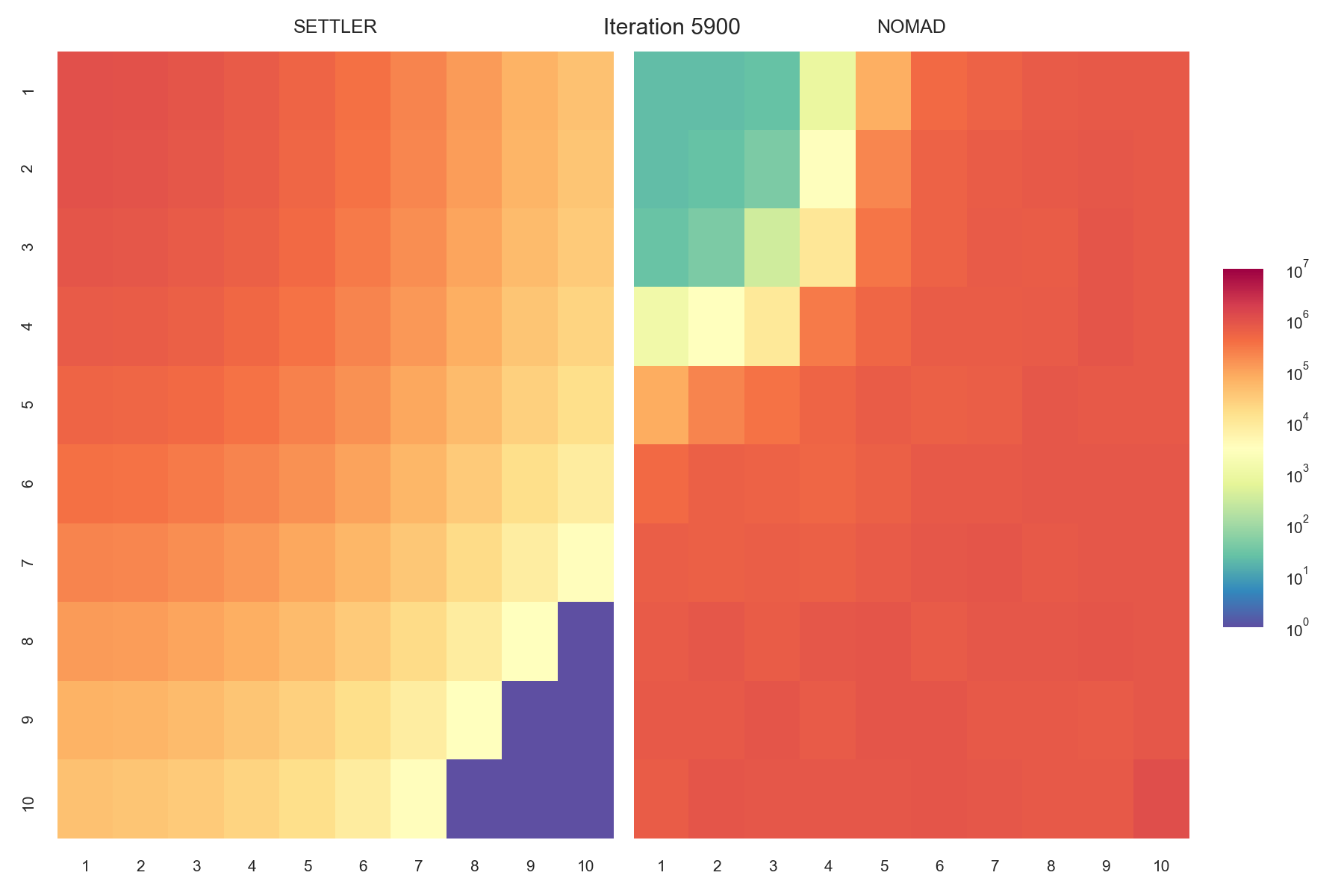

Supplement: Supplementary file 1 [file biology-10-01019-s001.zip › Spatio-temporal dynamics heatmaps/chempenoff_extremelyscarce_lindeath_period1000/5900.png]

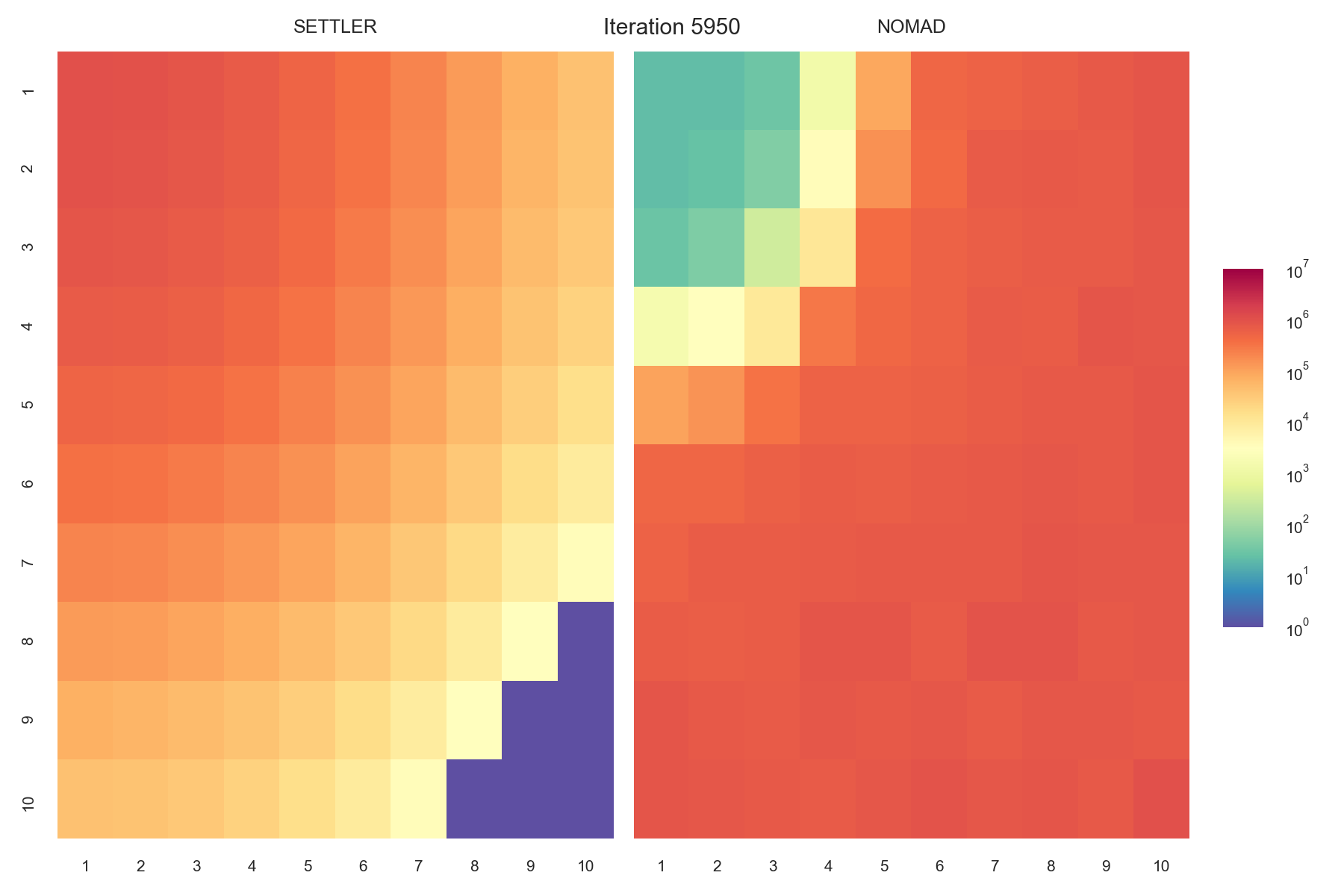

Supplement: Supplementary file 1 [file biology-10-01019-s001.zip › Spatio-temporal dynamics heatmaps/chempenoff_extremelyscarce_lindeath_period1000/5950.png]

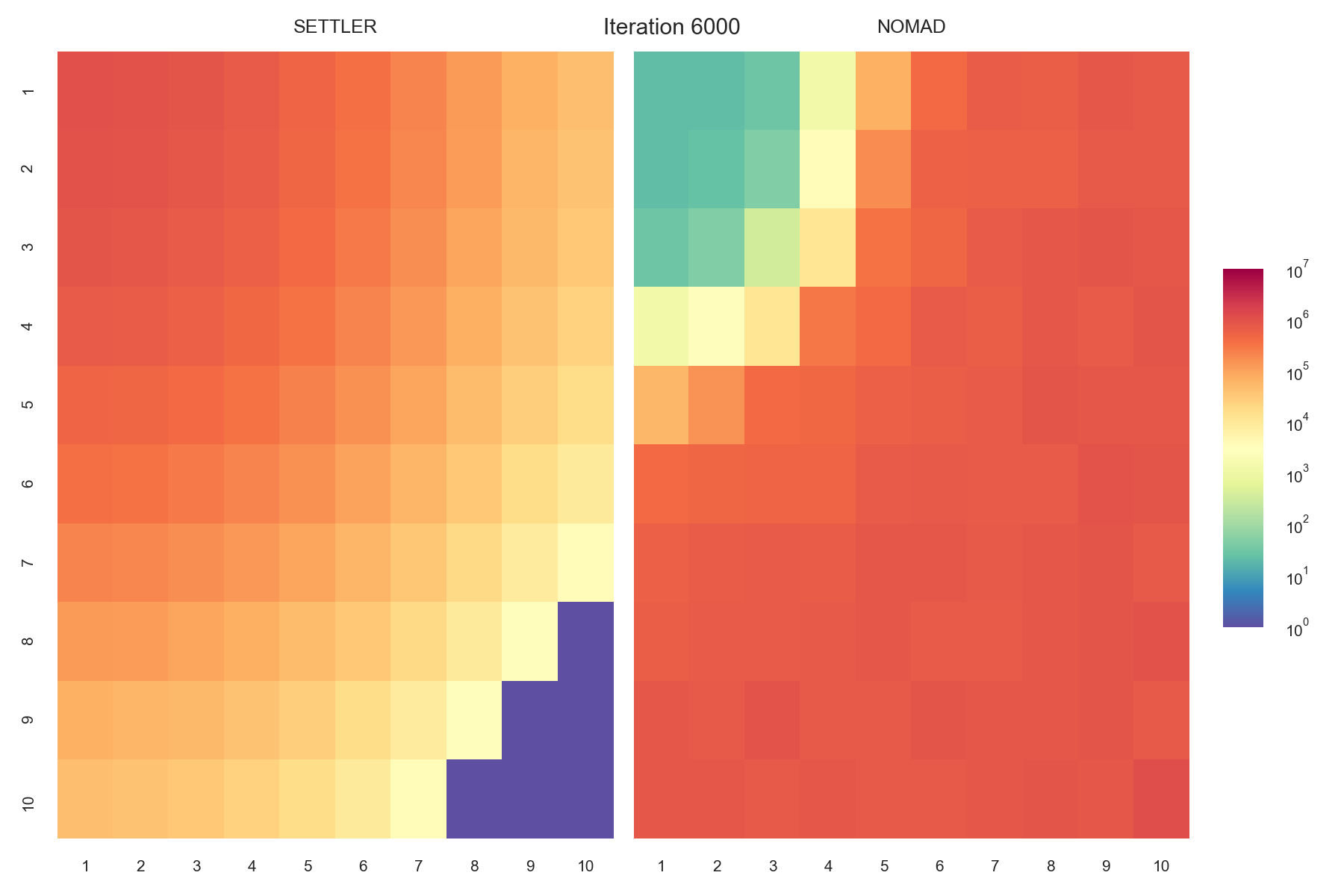

Supplement: Supplementary file 1 [file biology-10-01019-s001.zip › Spatio-temporal dynamics heatmaps/chempenoff_extremelyscarce_lindeath_period1000/6000.png]

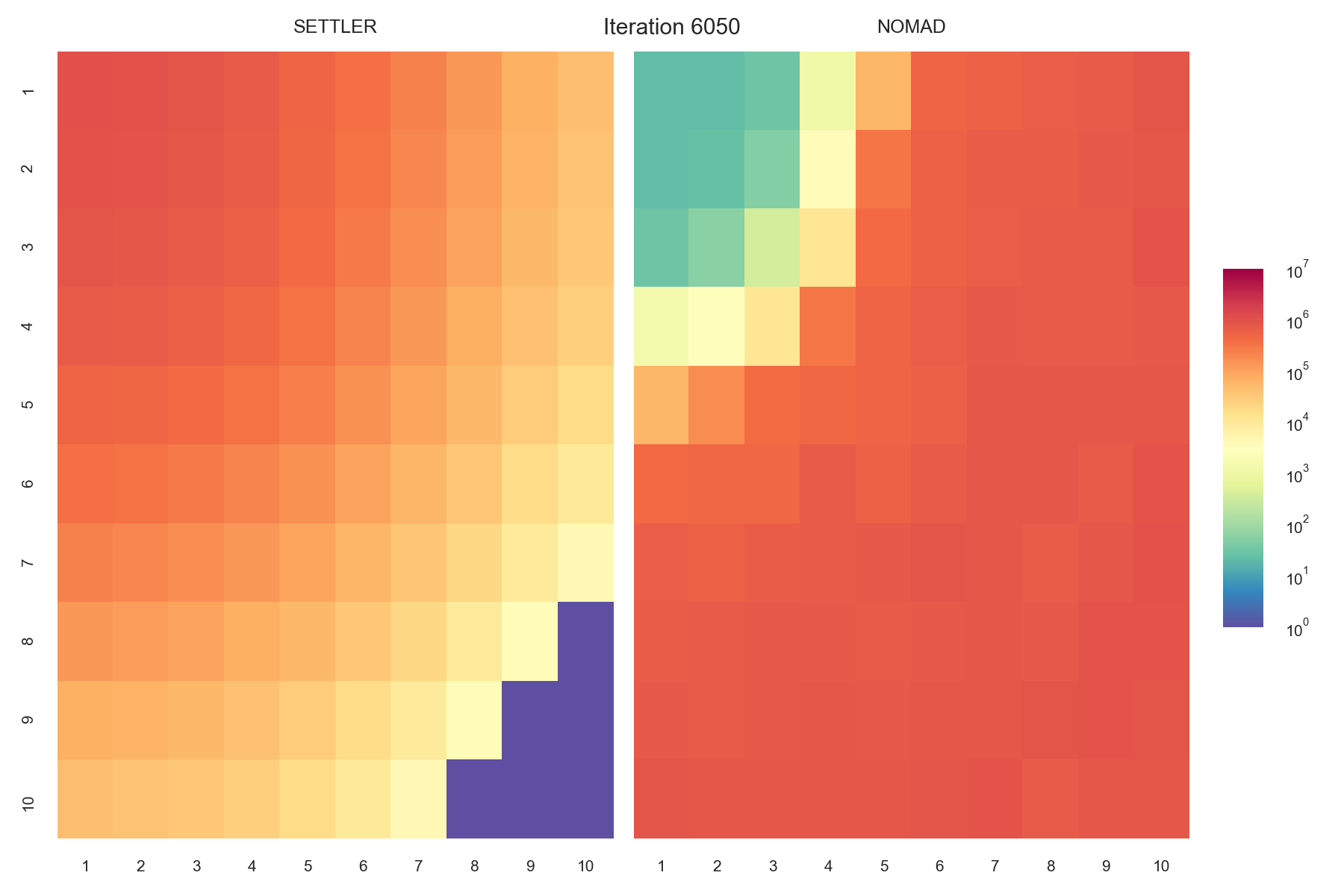

Supplement: Supplementary file 1 [file biology-10-01019-s001.zip › Spatio-temporal dynamics heatmaps/chempenoff_extremelyscarce_lindeath_period1000/6050.png]

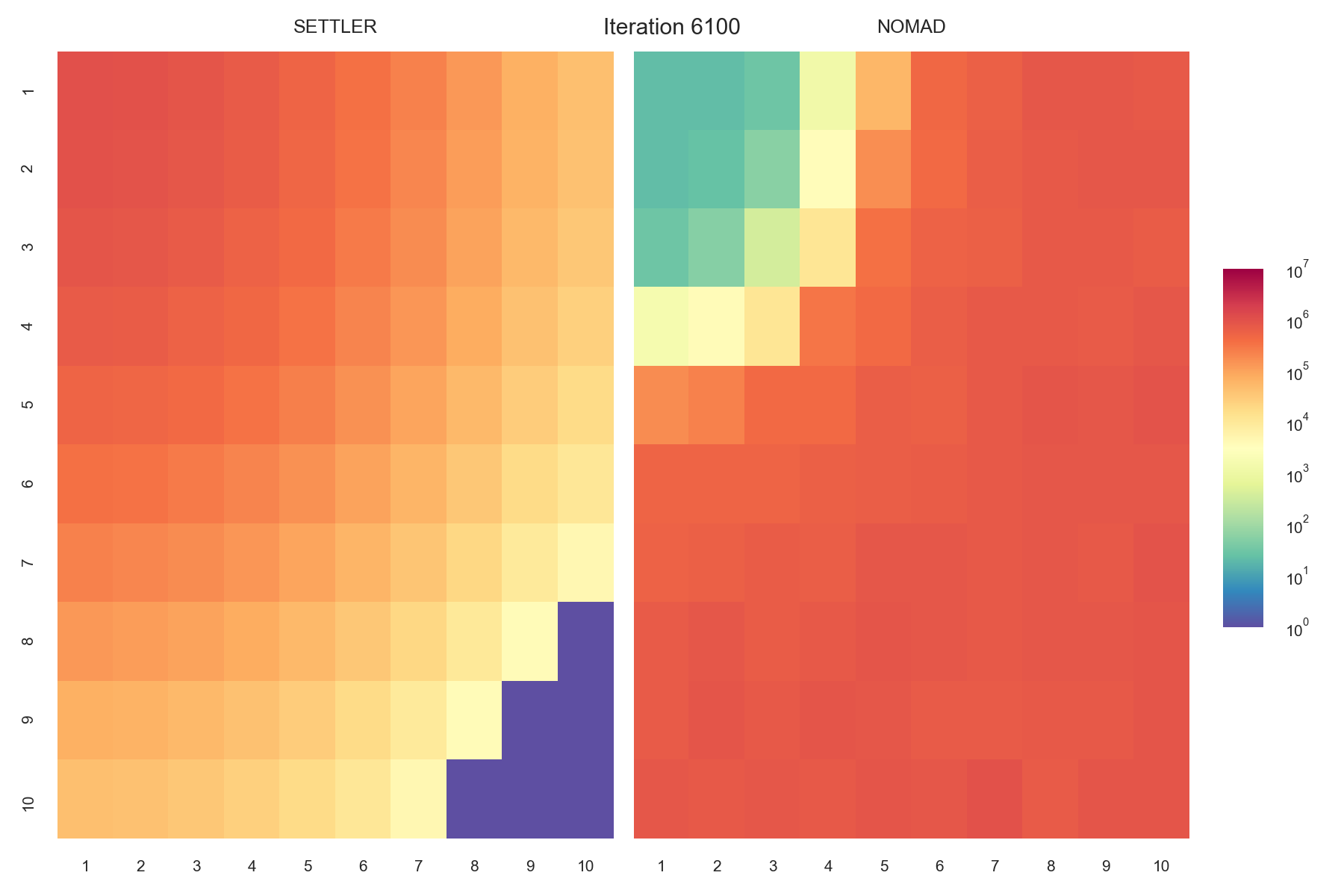

Supplement: Supplementary file 1 [file biology-10-01019-s001.zip › Spatio-temporal dynamics heatmaps/chempenoff_extremelyscarce_lindeath_period1000/6100.png]

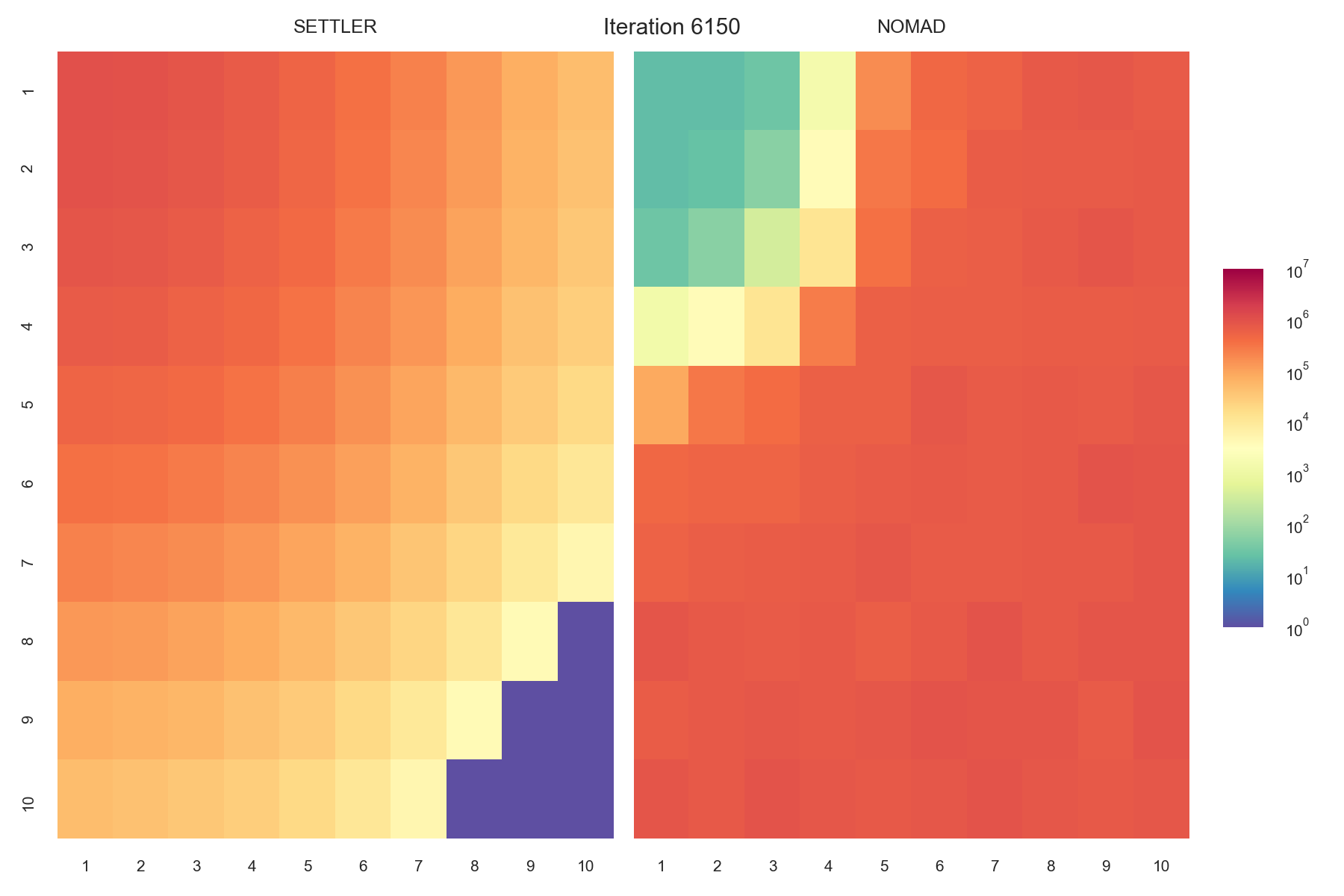

Supplement: Supplementary file 1 [file biology-10-01019-s001.zip › Spatio-temporal dynamics heatmaps/chempenoff_extremelyscarce_lindeath_period1000/6150.png]

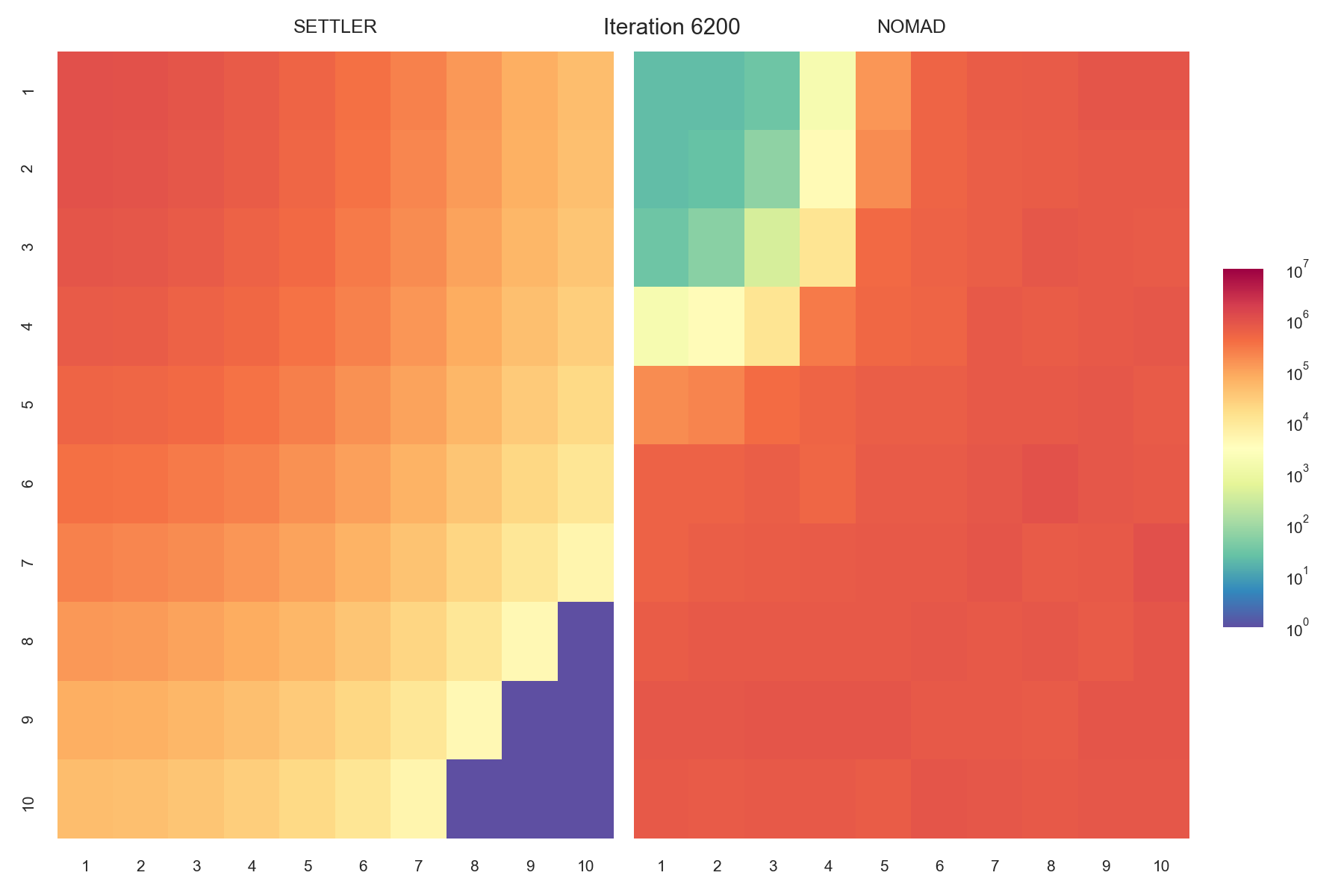

Supplement: Supplementary file 1 [file biology-10-01019-s001.zip › Spatio-temporal dynamics heatmaps/chempenoff_extremelyscarce_lindeath_period1000/6200.png]

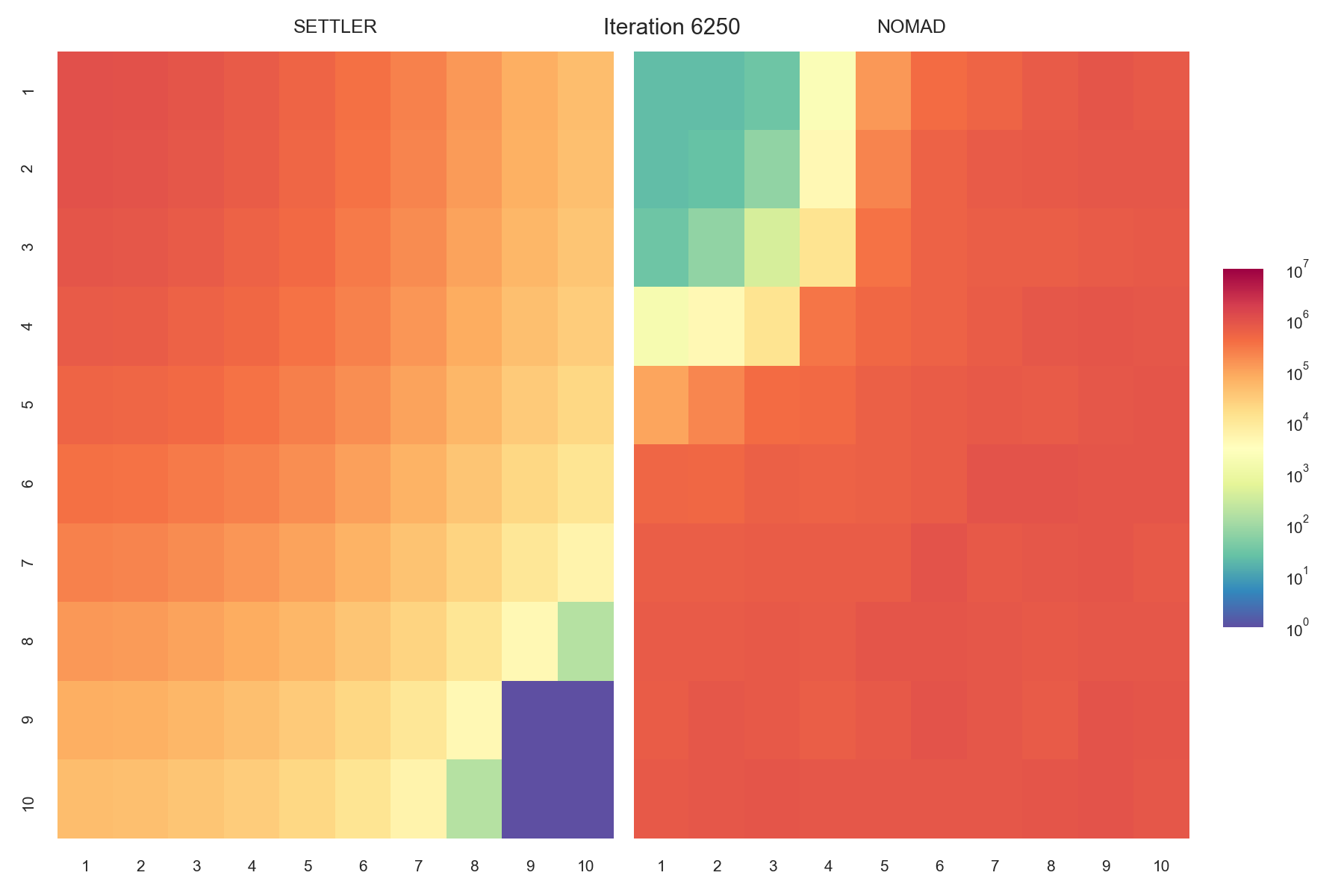

Supplement: Supplementary file 1 [file biology-10-01019-s001.zip › Spatio-temporal dynamics heatmaps/chempenoff_extremelyscarce_lindeath_period1000/6250.png]

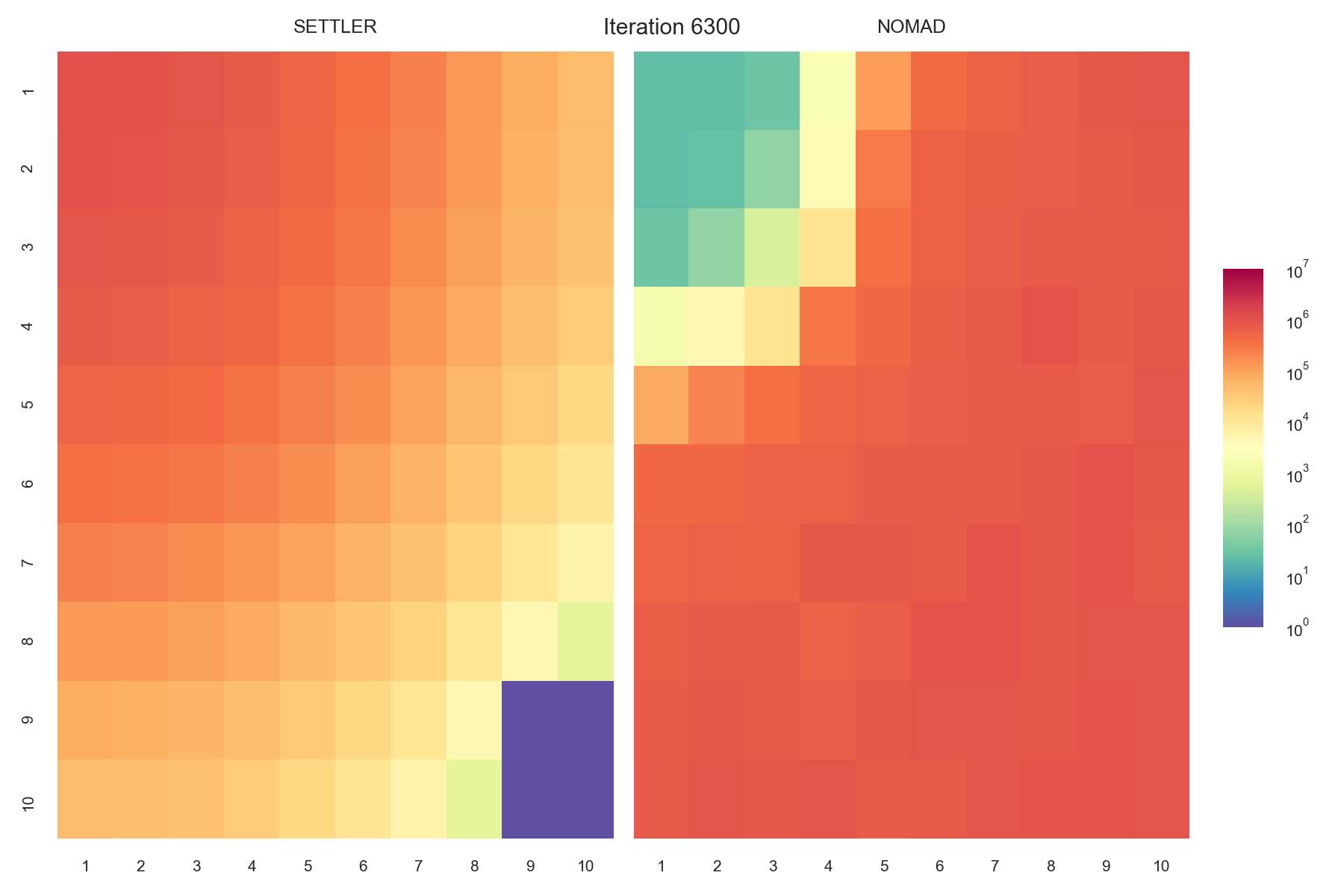

Supplement: Supplementary file 1 [file biology-10-01019-s001.zip › Spatio-temporal dynamics heatmaps/chempenoff_extremelyscarce_lindeath_period1000/6300.png]

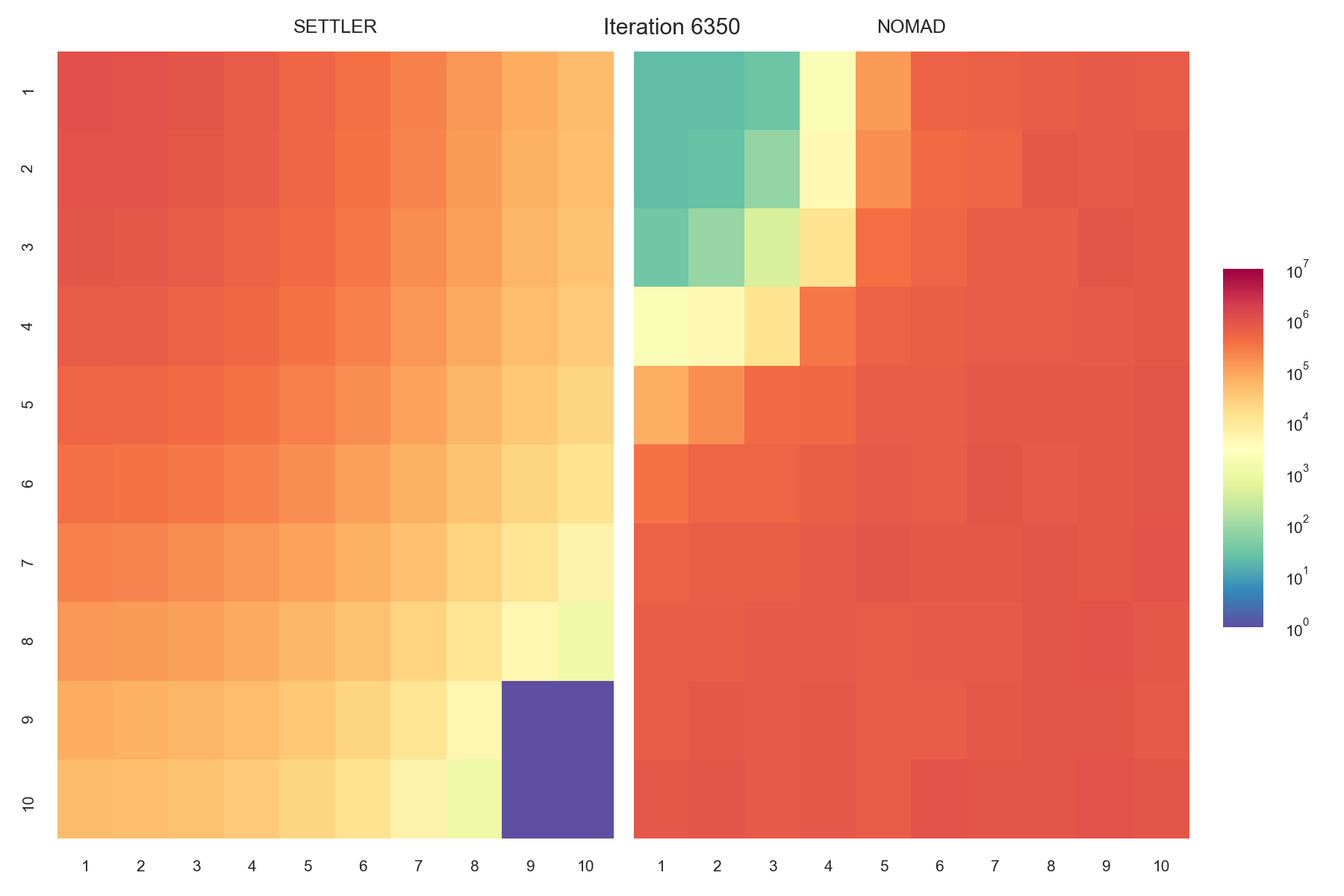

Supplement: Supplementary file 1 [file biology-10-01019-s001.zip › Spatio-temporal dynamics heatmaps/chempenoff_extremelyscarce_lindeath_period1000/6350.png]

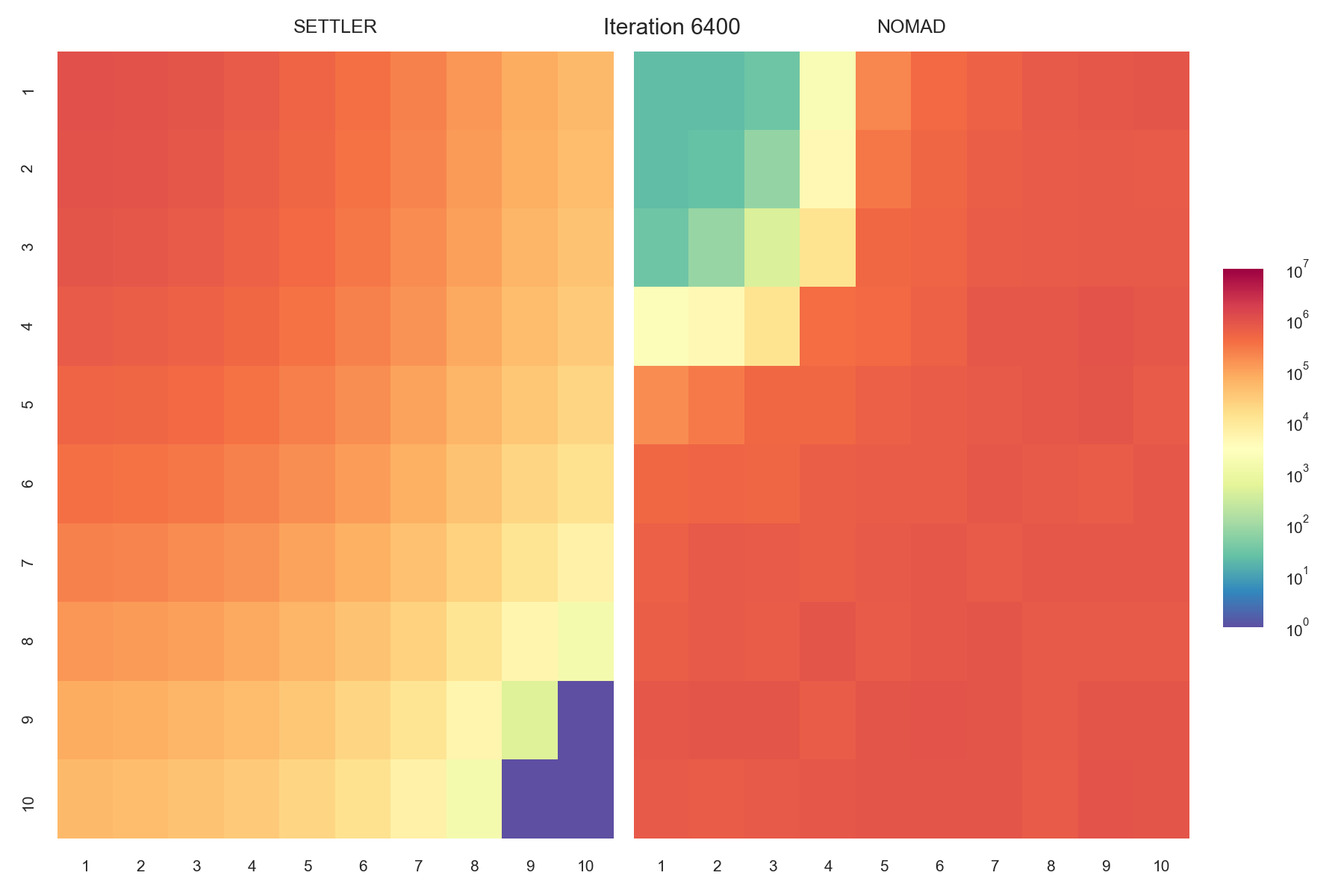

Supplement: Supplementary file 1 [file biology-10-01019-s001.zip › Spatio-temporal dynamics heatmaps/chempenoff_extremelyscarce_lindeath_period1000/6400.png]

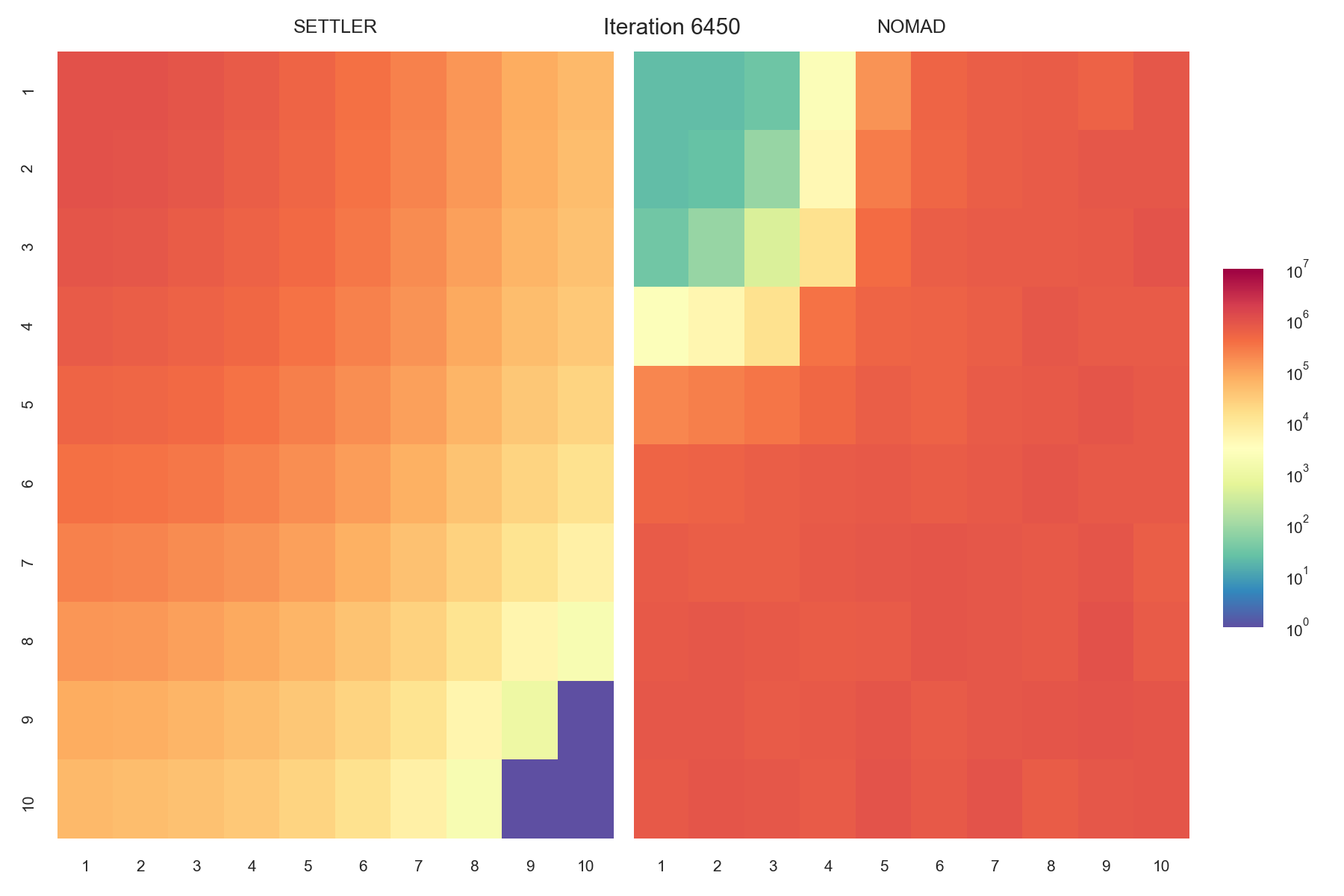

Supplement: Supplementary file 1 [file biology-10-01019-s001.zip › Spatio-temporal dynamics heatmaps/chempenoff_extremelyscarce_lindeath_period1000/6450.png]

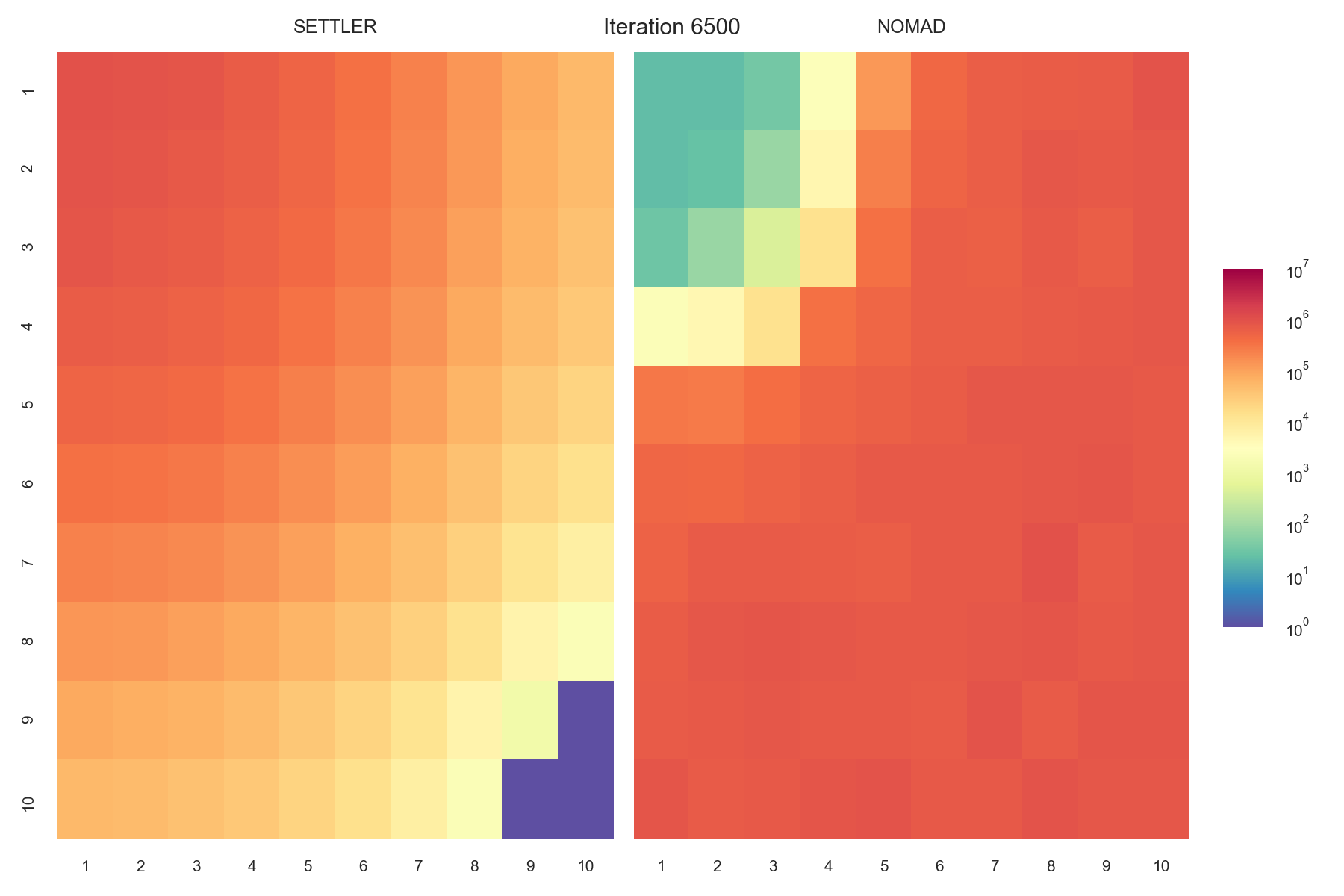

Supplement: Supplementary file 1 [file biology-10-01019-s001.zip › Spatio-temporal dynamics heatmaps/chempenoff_extremelyscarce_lindeath_period1000/6500.png]

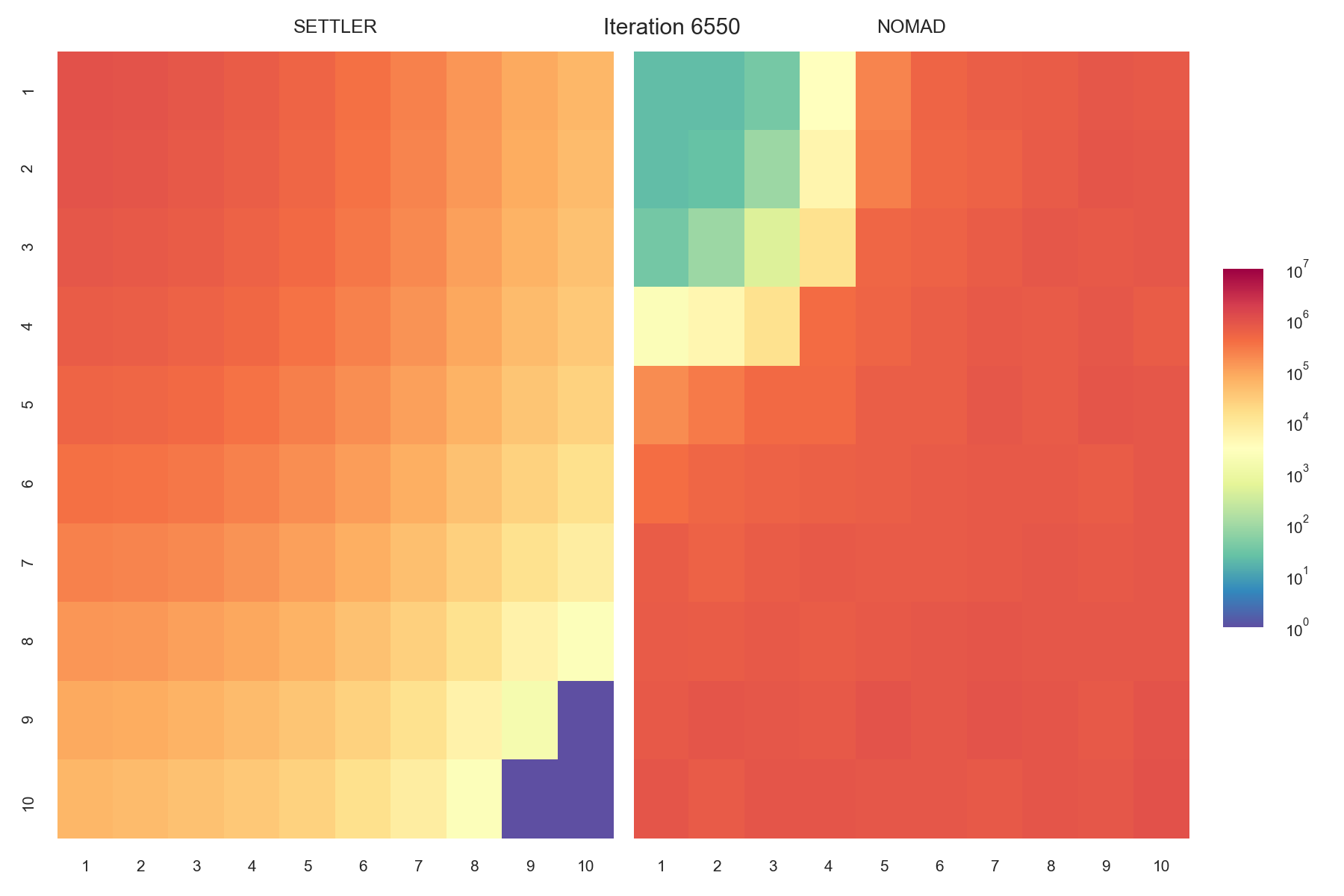

Supplement: Supplementary file 1 [file biology-10-01019-s001.zip › Spatio-temporal dynamics heatmaps/chempenoff_extremelyscarce_lindeath_period1000/6550.png]

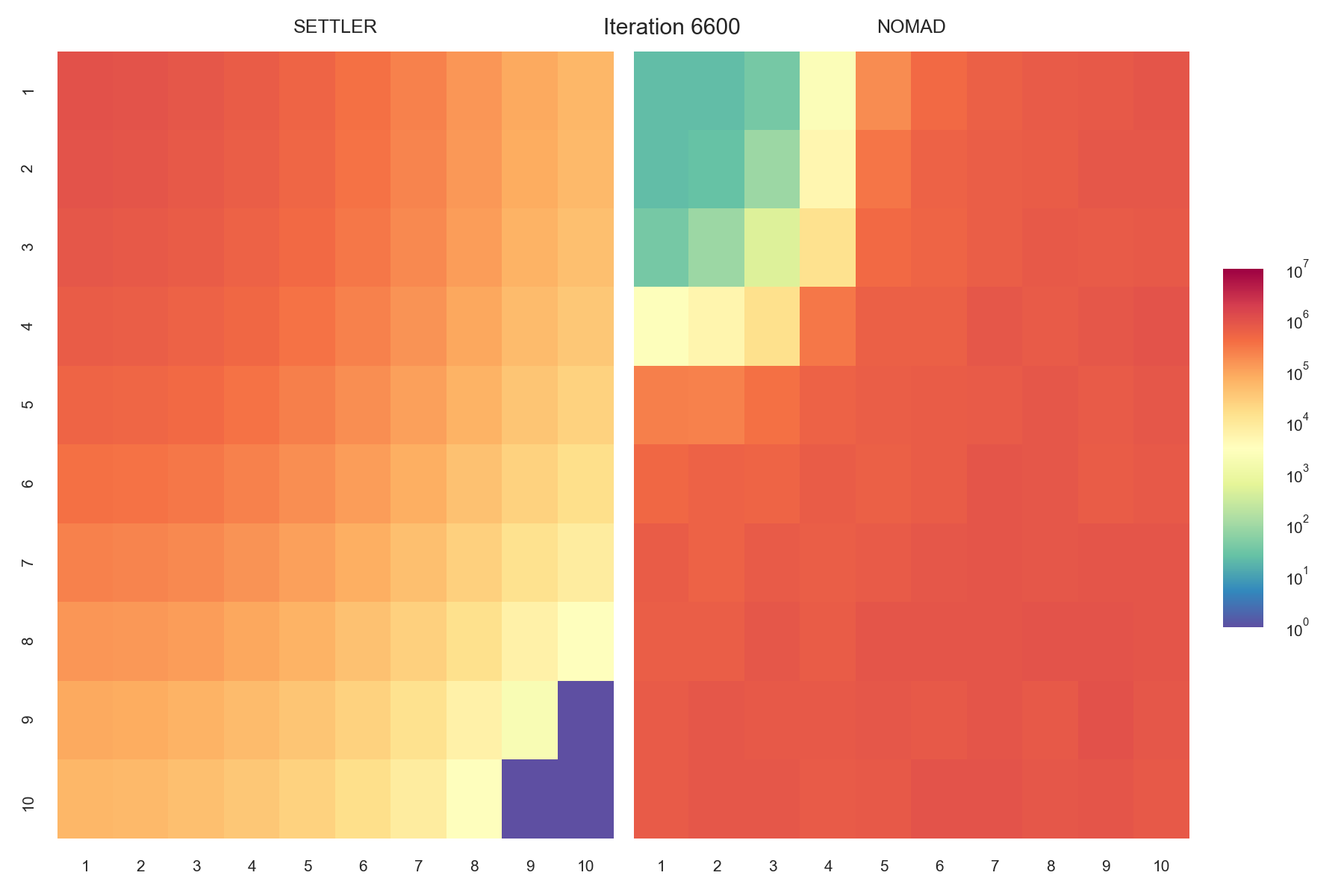

Supplement: Supplementary file 1 [file biology-10-01019-s001.zip › Spatio-temporal dynamics heatmaps/chempenoff_extremelyscarce_lindeath_period1000/6600.png]

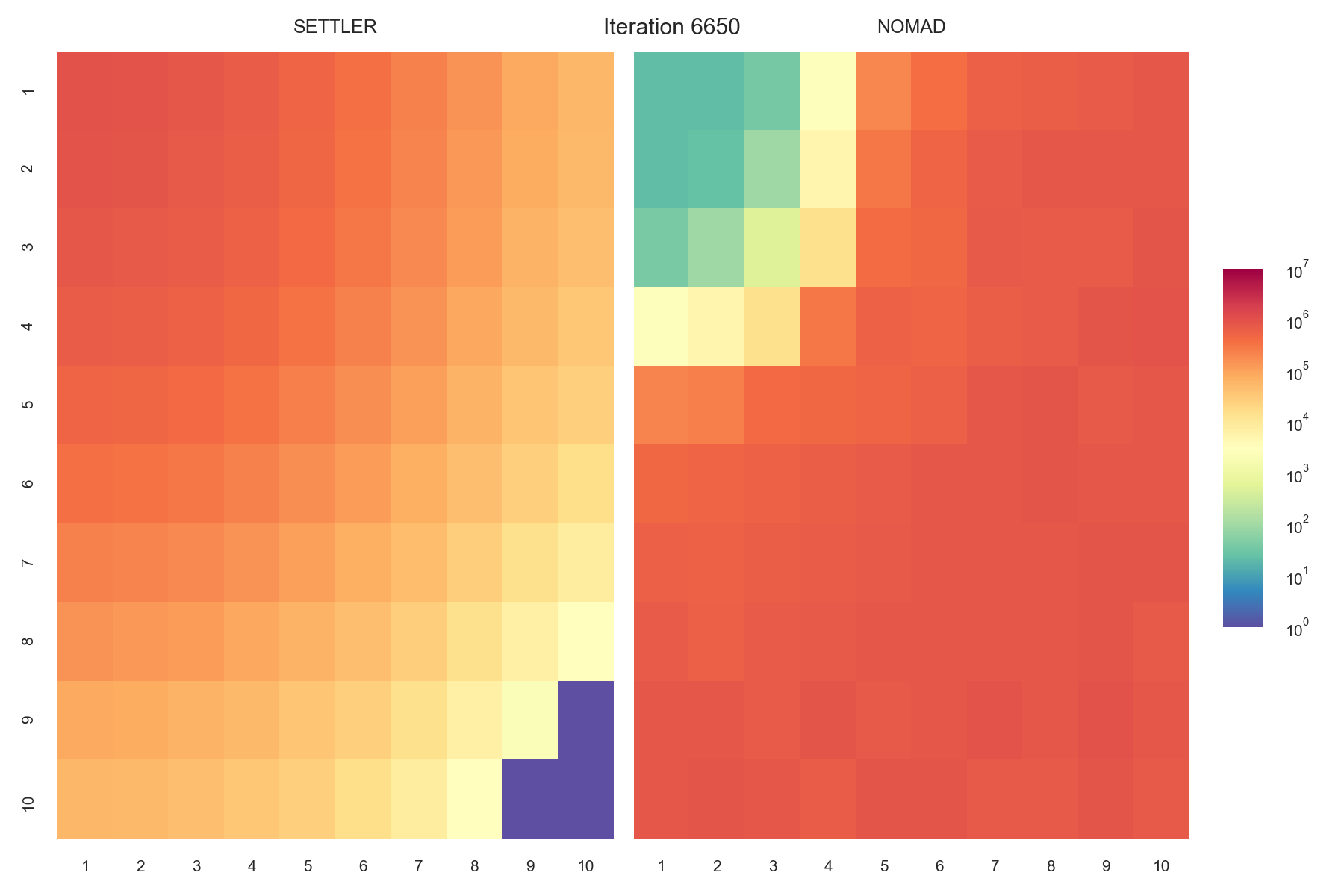

Supplement: Supplementary file 1 [file biology-10-01019-s001.zip › Spatio-temporal dynamics heatmaps/chempenoff_extremelyscarce_lindeath_period1000/6650.png]

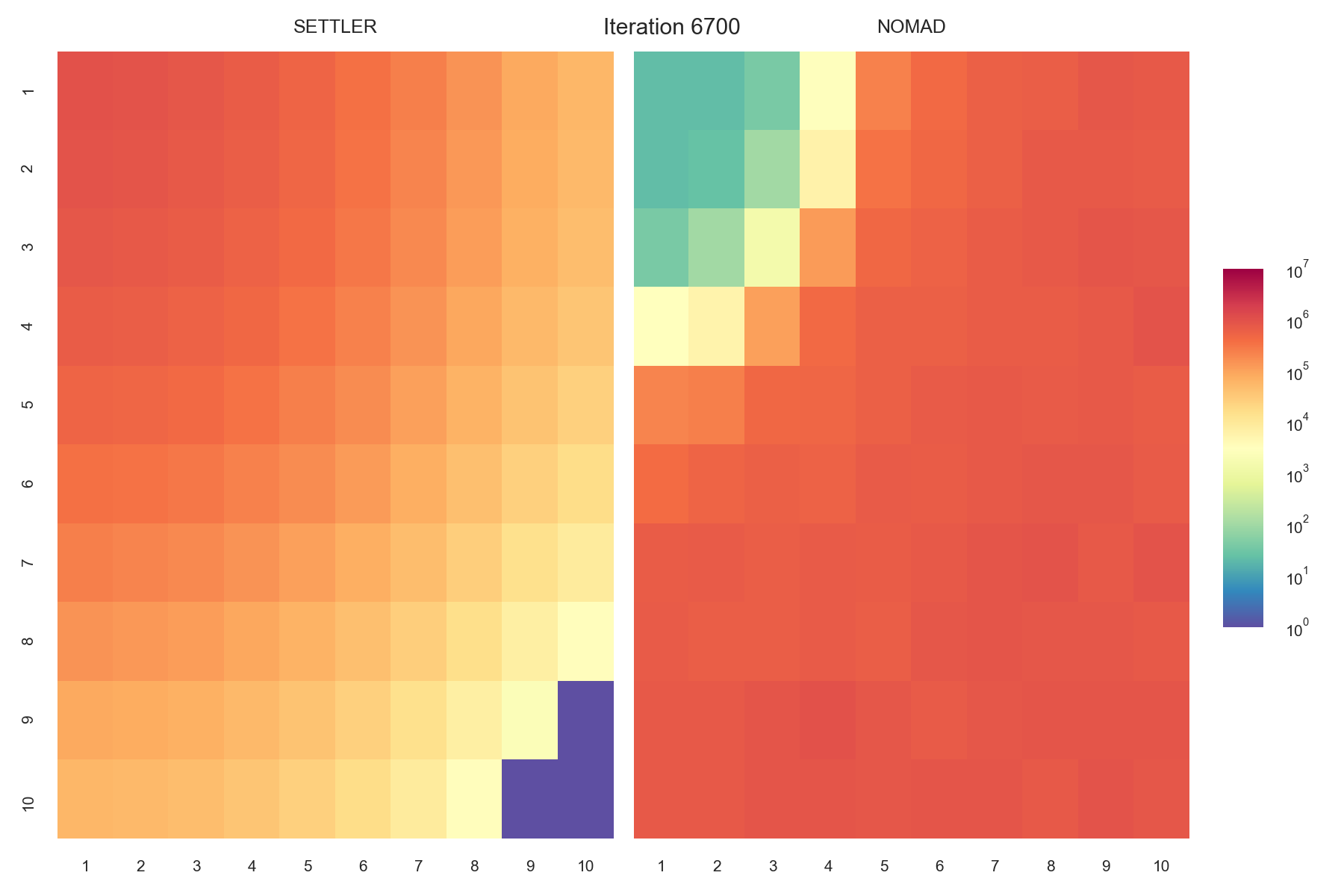

Supplement: Supplementary file 1 [file biology-10-01019-s001.zip › Spatio-temporal dynamics heatmaps/chempenoff_extremelyscarce_lindeath_period1000/6700.png]

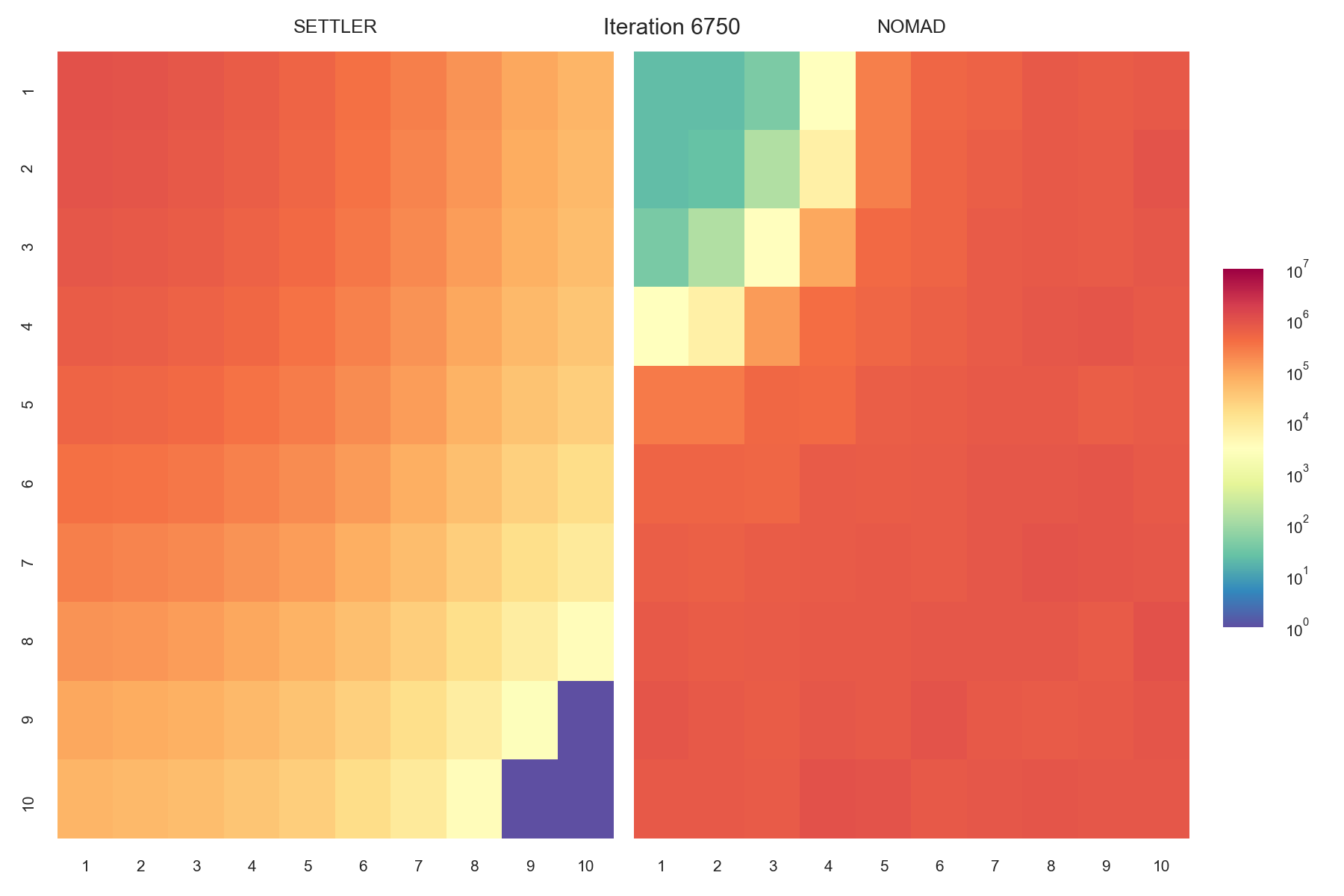

Supplement: Supplementary file 1 [file biology-10-01019-s001.zip › Spatio-temporal dynamics heatmaps/chempenoff_extremelyscarce_lindeath_period1000/6750.png]

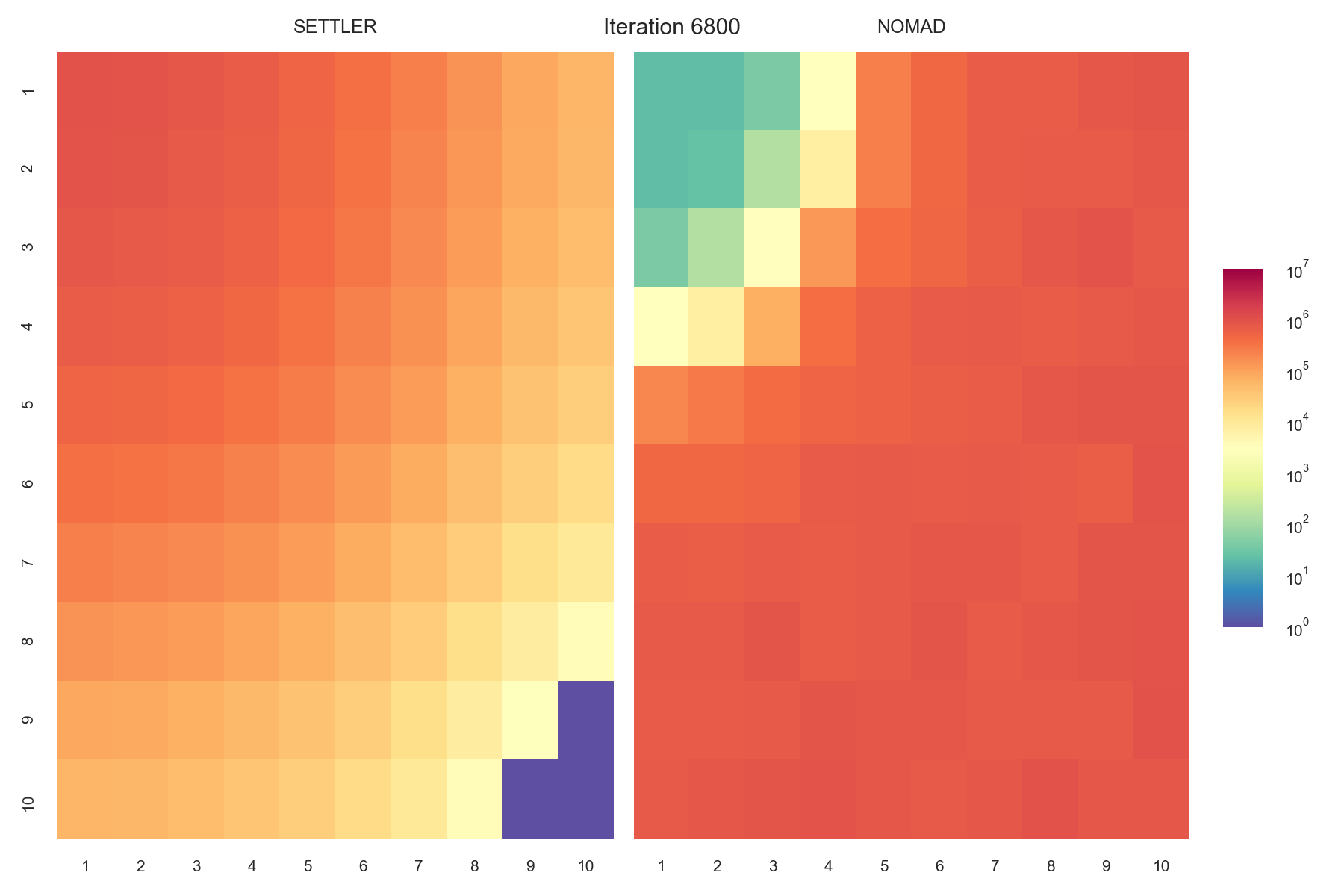

Supplement: Supplementary file 1 [file biology-10-01019-s001.zip › Spatio-temporal dynamics heatmaps/chempenoff_extremelyscarce_lindeath_period1000/6800.png]

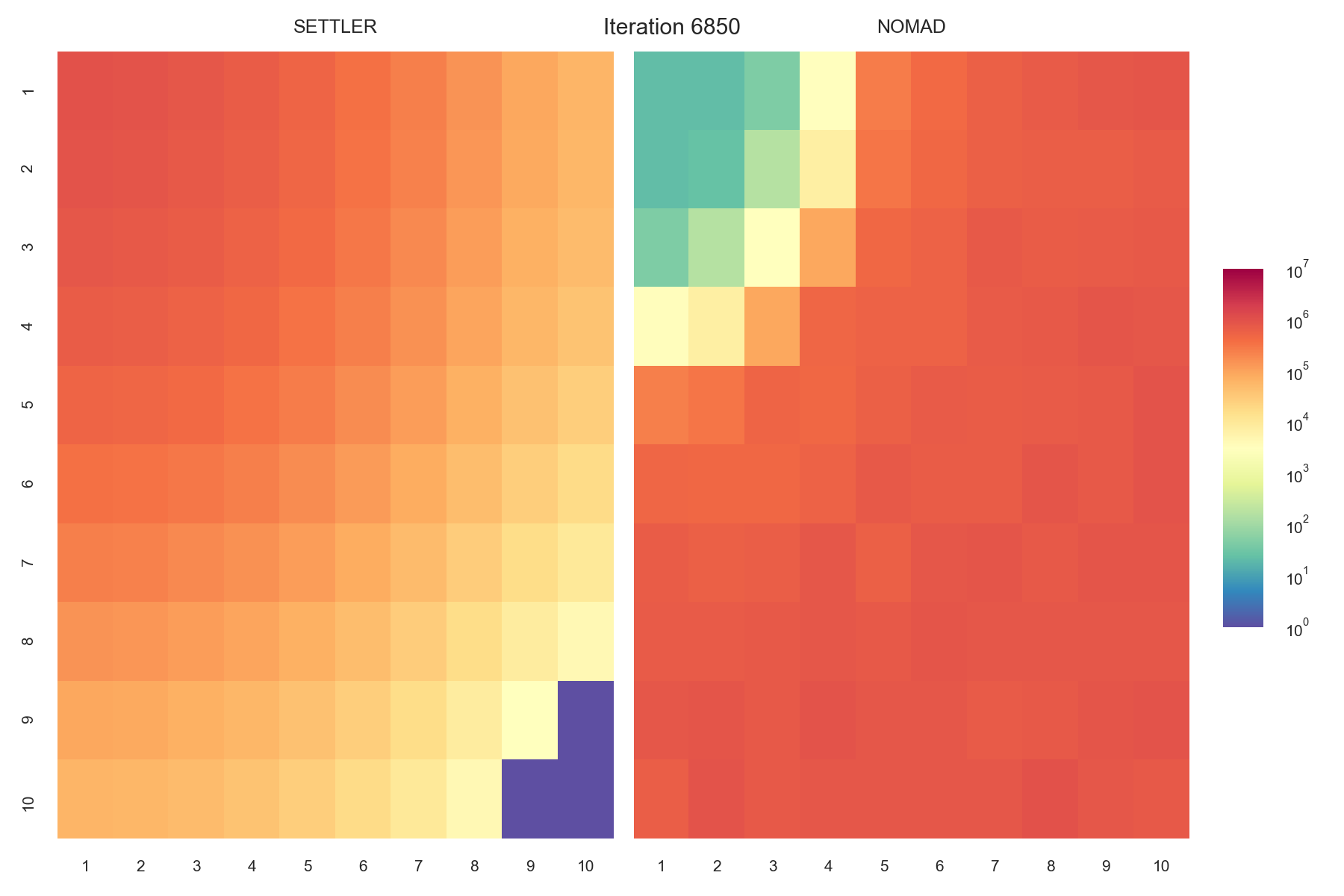

Supplement: Supplementary file 1 [file biology-10-01019-s001.zip › Spatio-temporal dynamics heatmaps/chempenoff_extremelyscarce_lindeath_period1000/6850.png]

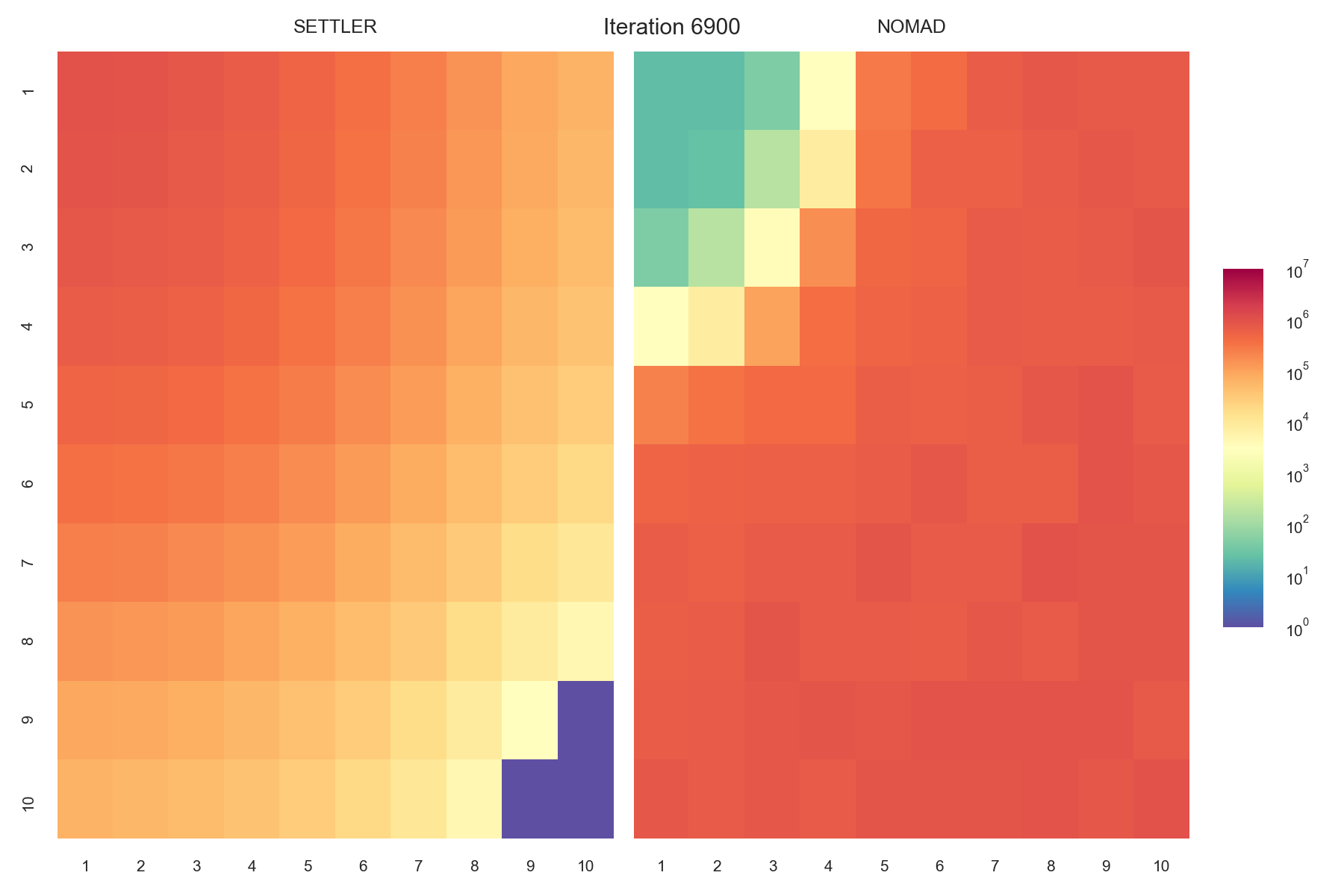

Supplement: Supplementary file 1 [file biology-10-01019-s001.zip › Spatio-temporal dynamics heatmaps/chempenoff_extremelyscarce_lindeath_period1000/6900.png]

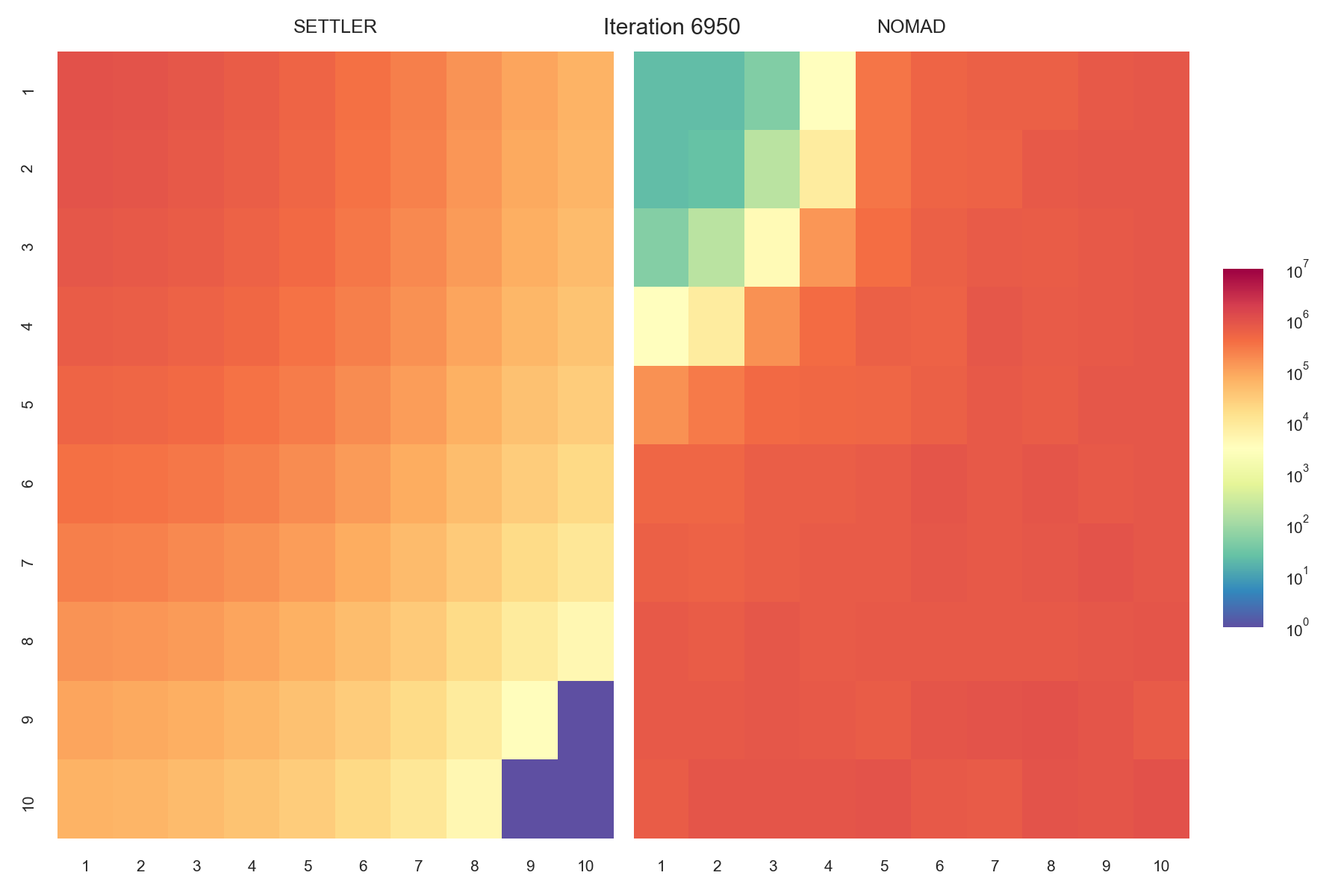

Supplement: Supplementary file 1 [file biology-10-01019-s001.zip › Spatio-temporal dynamics heatmaps/chempenoff_extremelyscarce_lindeath_period1000/6950.png]

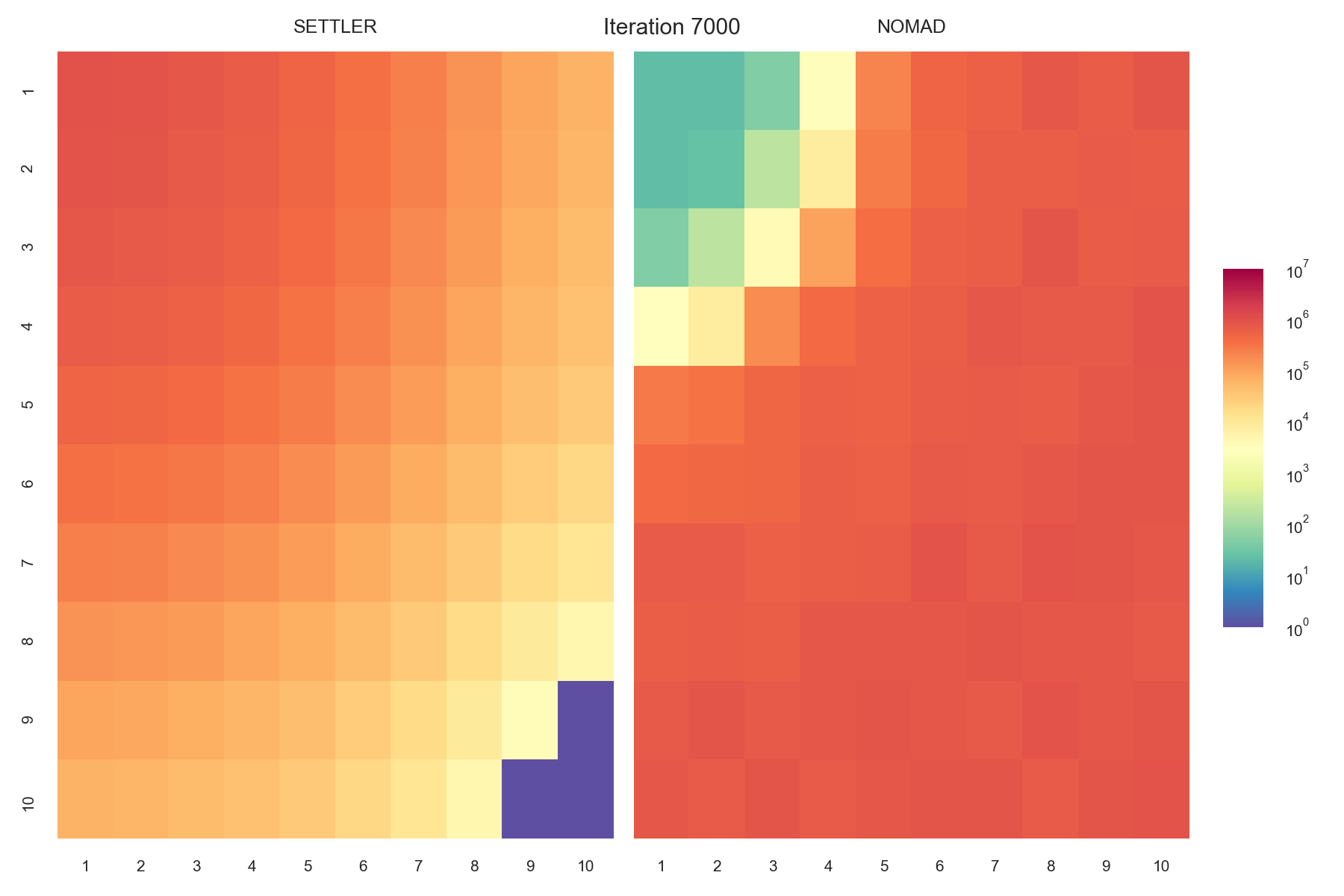

Supplement: Supplementary file 1 [file biology-10-01019-s001.zip › Spatio-temporal dynamics heatmaps/chempenoff_extremelyscarce_lindeath_period1000/7000.png]

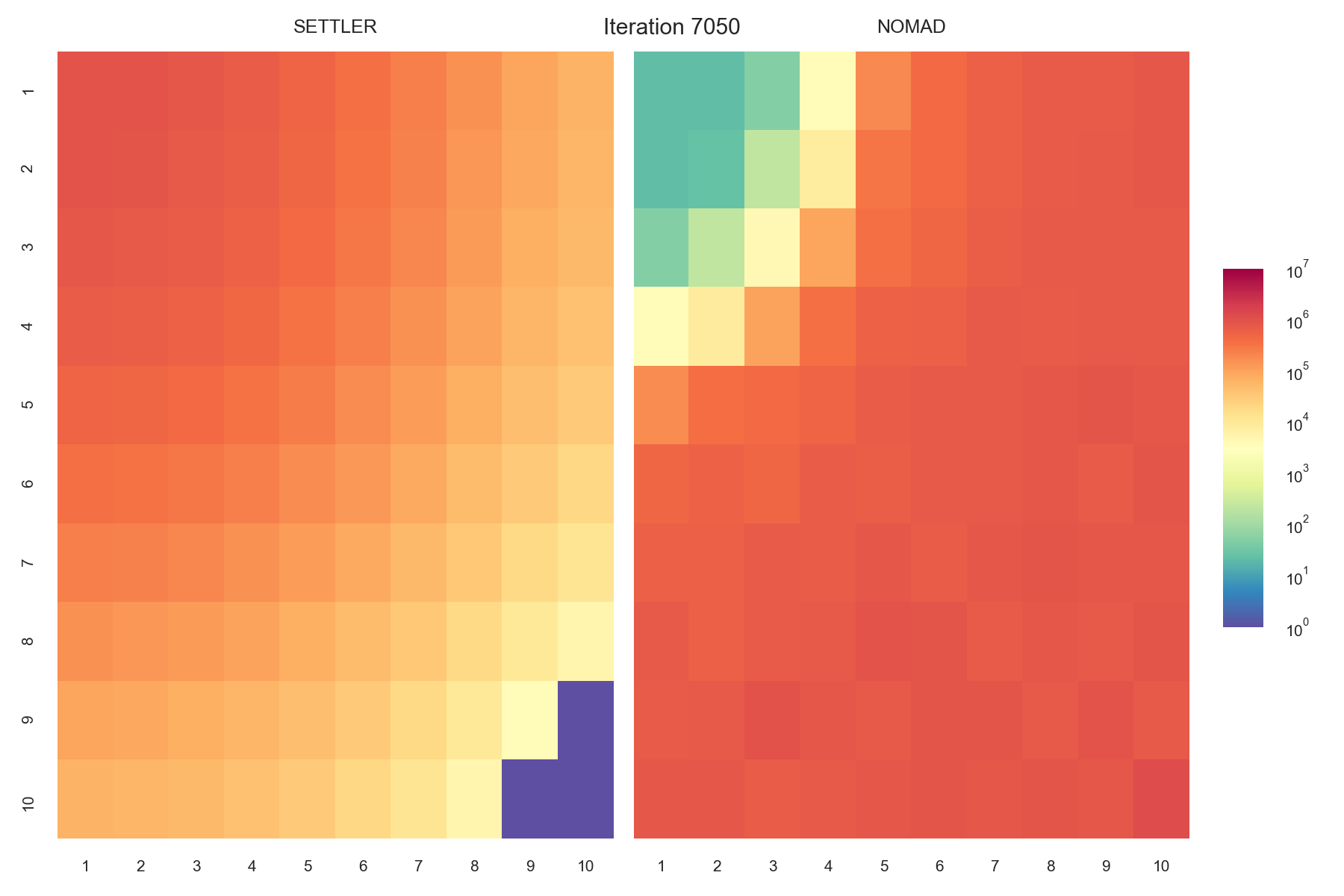

Supplement: Supplementary file 1 [file biology-10-01019-s001.zip › Spatio-temporal dynamics heatmaps/chempenoff_extremelyscarce_lindeath_period1000/7050.png]

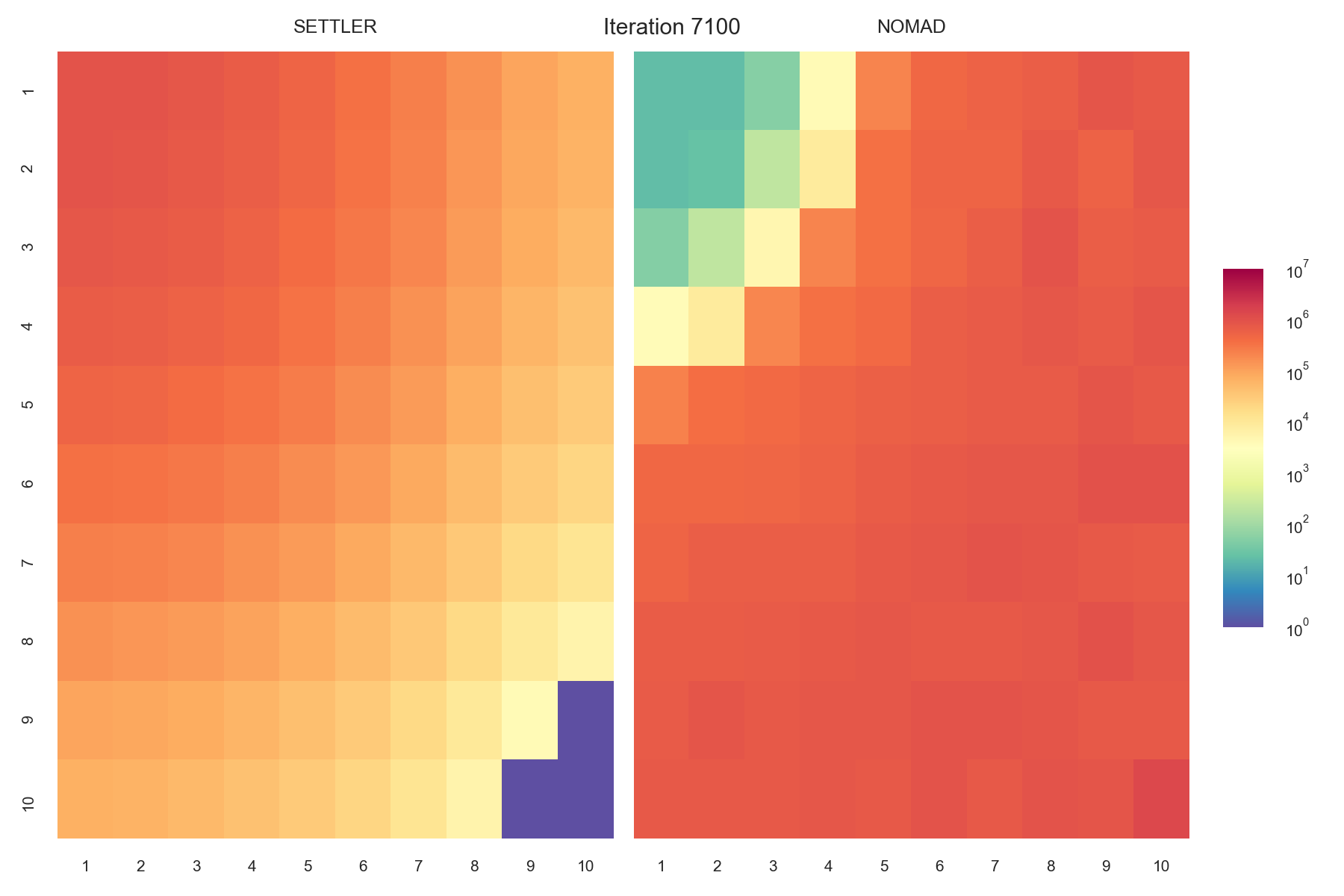

Supplement: Supplementary file 1 [file biology-10-01019-s001.zip › Spatio-temporal dynamics heatmaps/chempenoff_extremelyscarce_lindeath_period1000/7100.png]

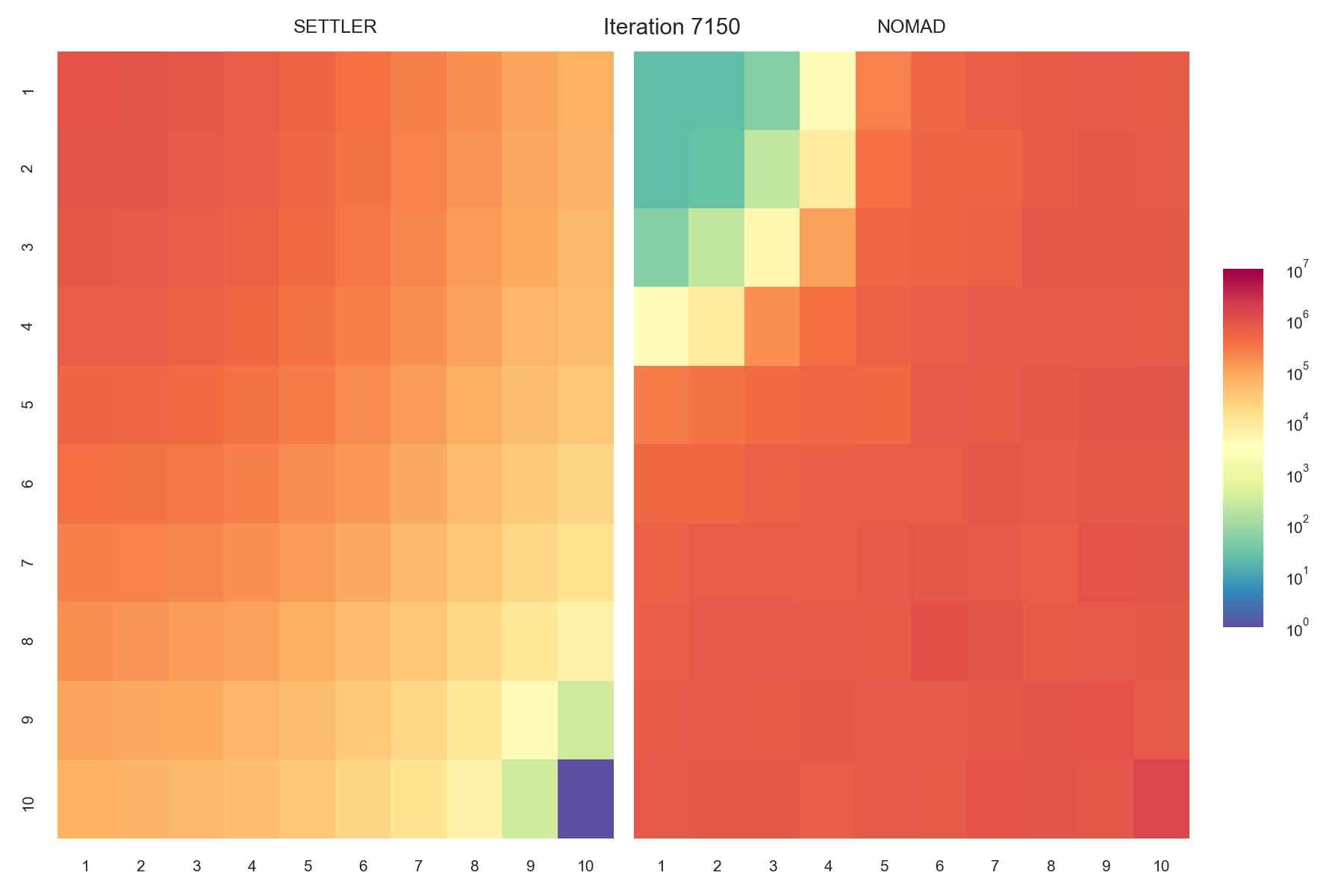

Supplement: Supplementary file 1 [file biology-10-01019-s001.zip › Spatio-temporal dynamics heatmaps/chempenoff_extremelyscarce_lindeath_period1000/7150.png]

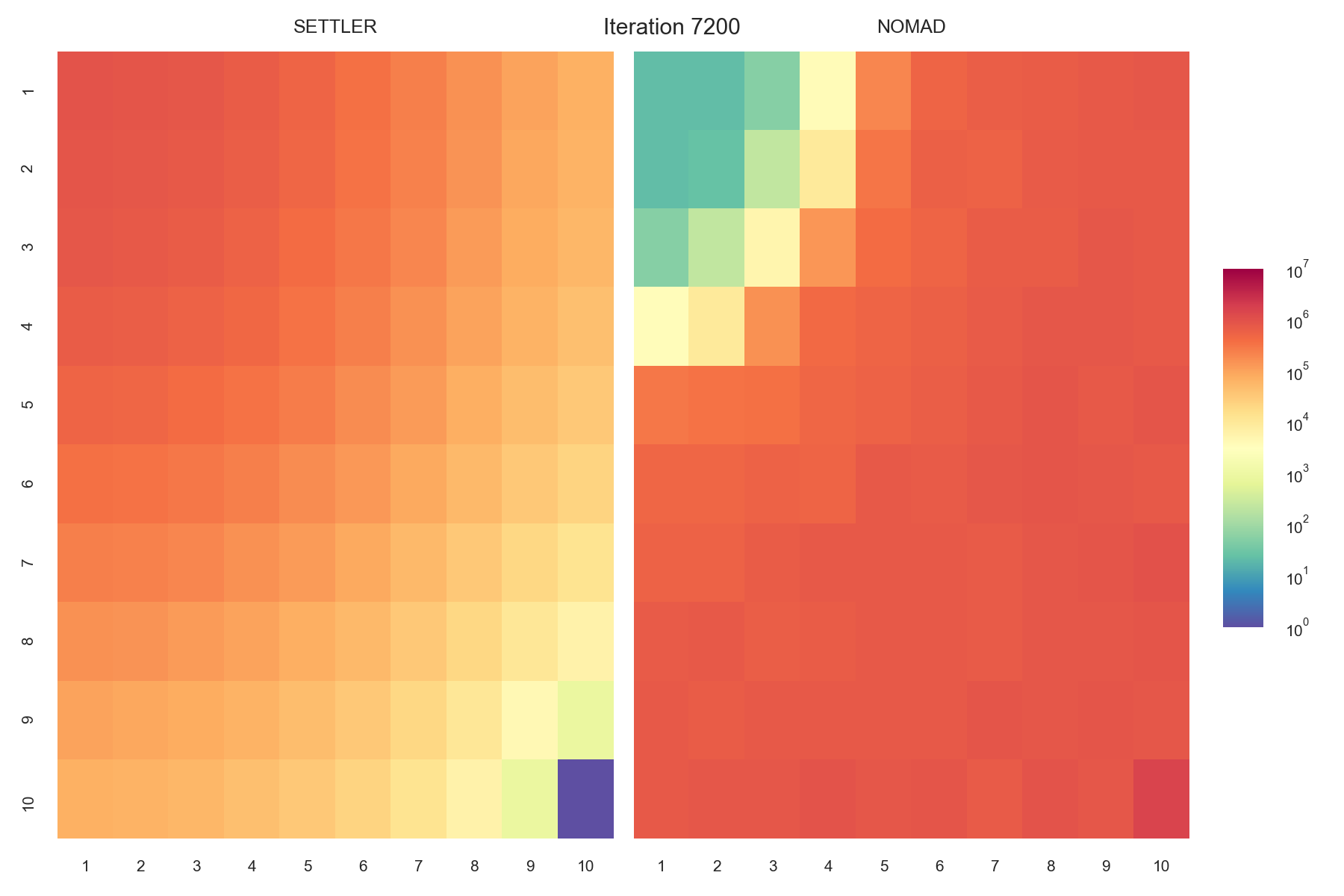

Supplement: Supplementary file 1 [file biology-10-01019-s001.zip › Spatio-temporal dynamics heatmaps/chempenoff_extremelyscarce_lindeath_period1000/7200.png]

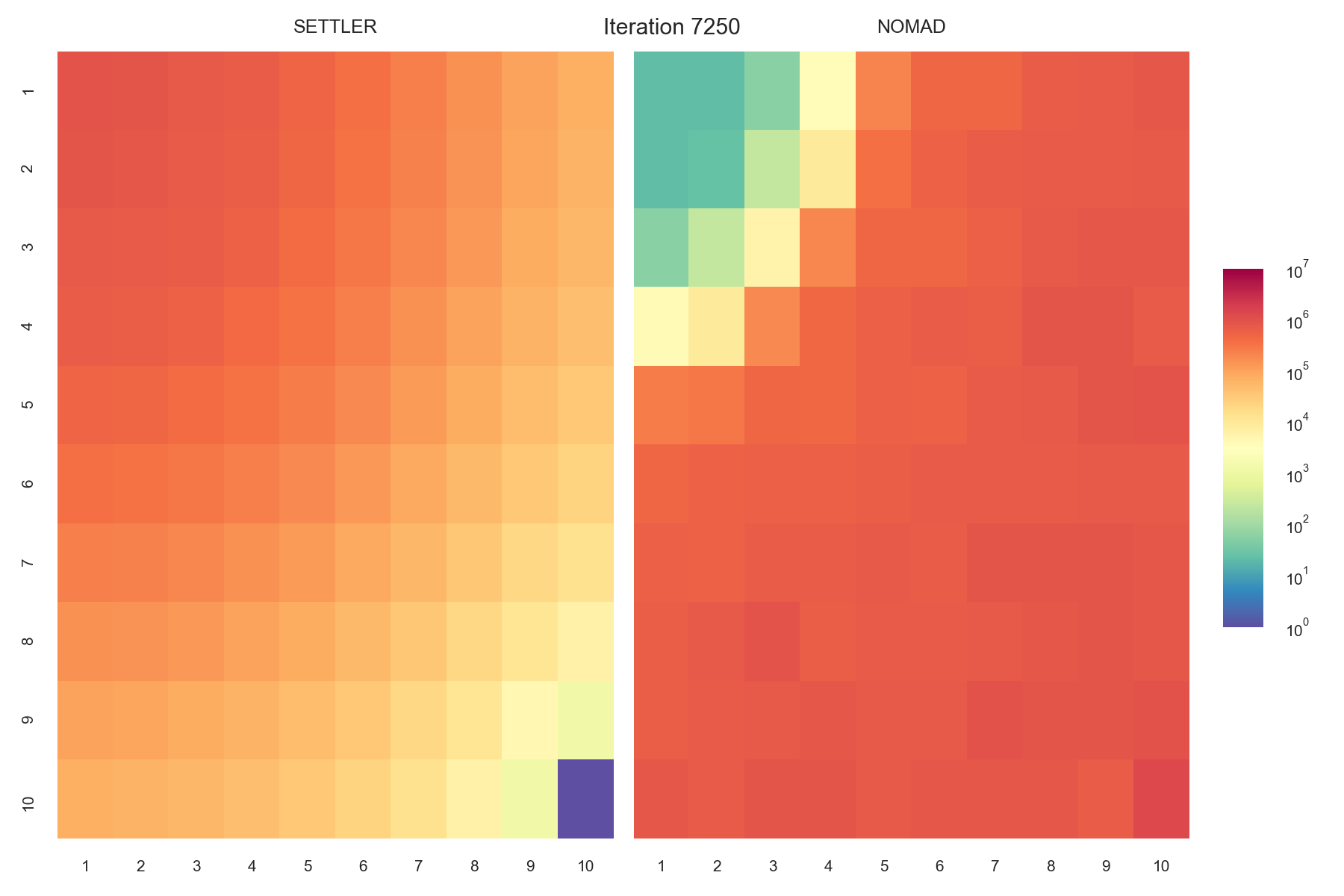

Supplement: Supplementary file 1 [file biology-10-01019-s001.zip › Spatio-temporal dynamics heatmaps/chempenoff_extremelyscarce_lindeath_period1000/7250.png]

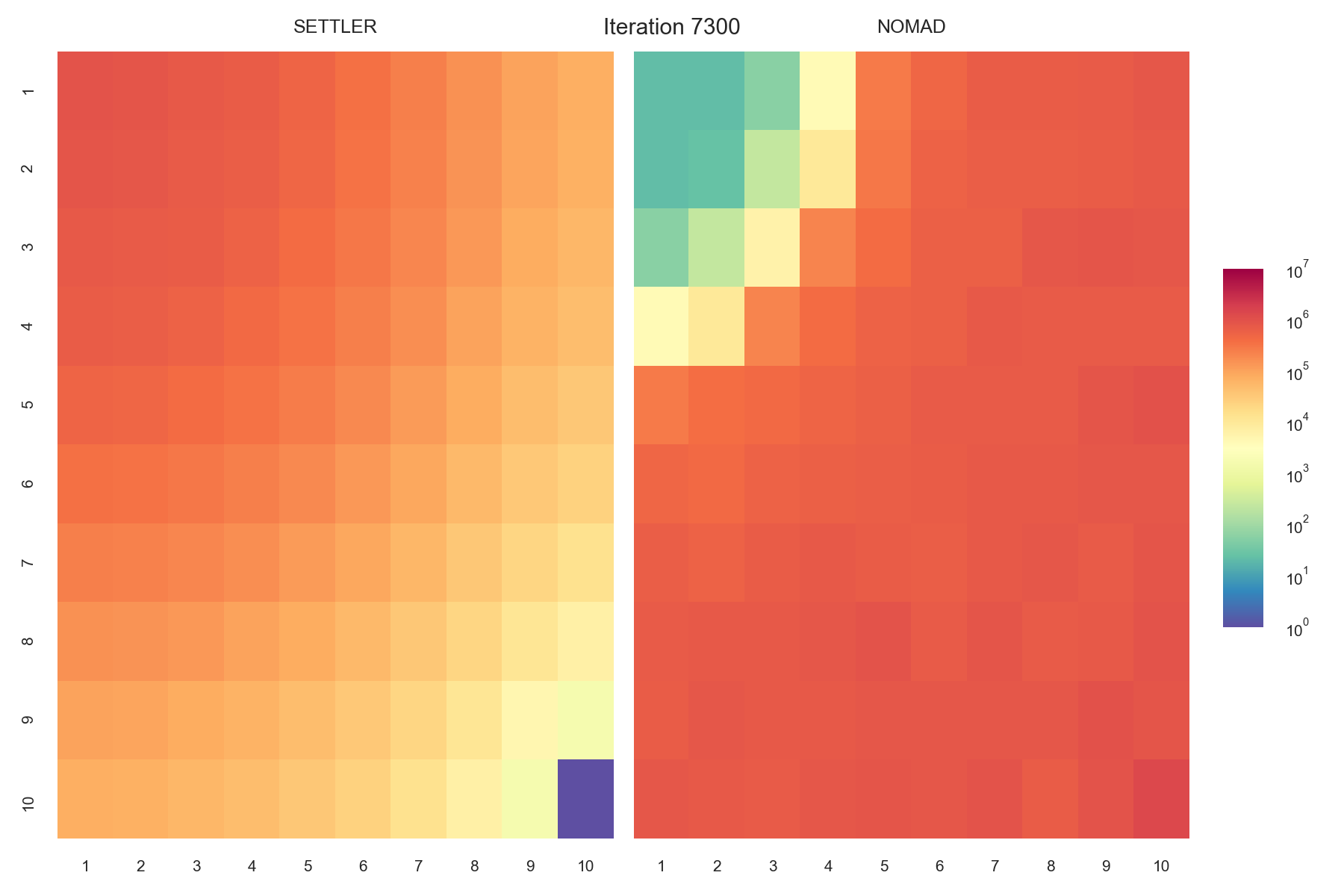

Supplement: Supplementary file 1 [file biology-10-01019-s001.zip › Spatio-temporal dynamics heatmaps/chempenoff_extremelyscarce_lindeath_period1000/7300.png]

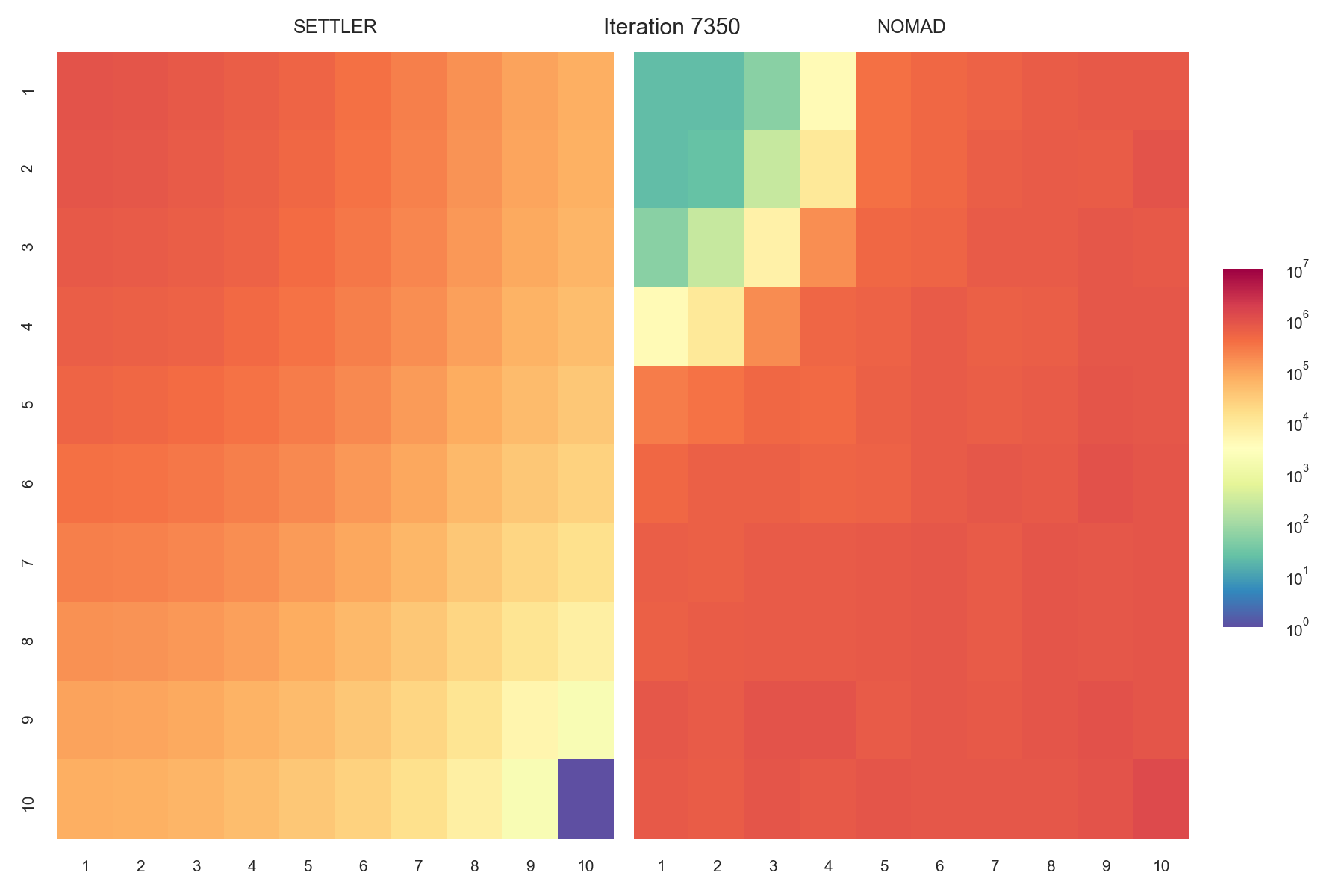

Supplement: Supplementary file 1 [file biology-10-01019-s001.zip › Spatio-temporal dynamics heatmaps/chempenoff_extremelyscarce_lindeath_period1000/7350.png]

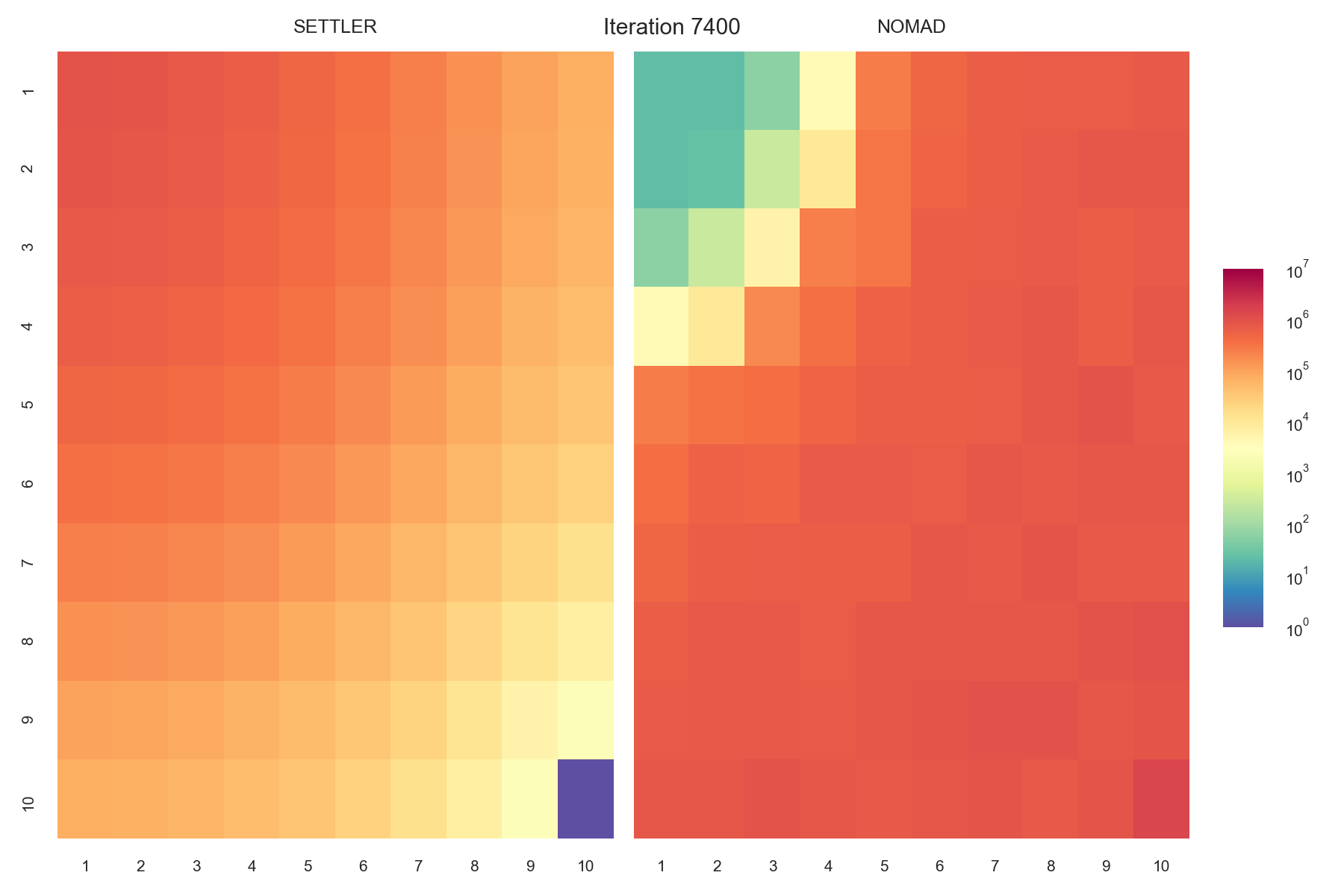

Supplement: Supplementary file 1 [file biology-10-01019-s001.zip › Spatio-temporal dynamics heatmaps/chempenoff_extremelyscarce_lindeath_period1000/7400.png]

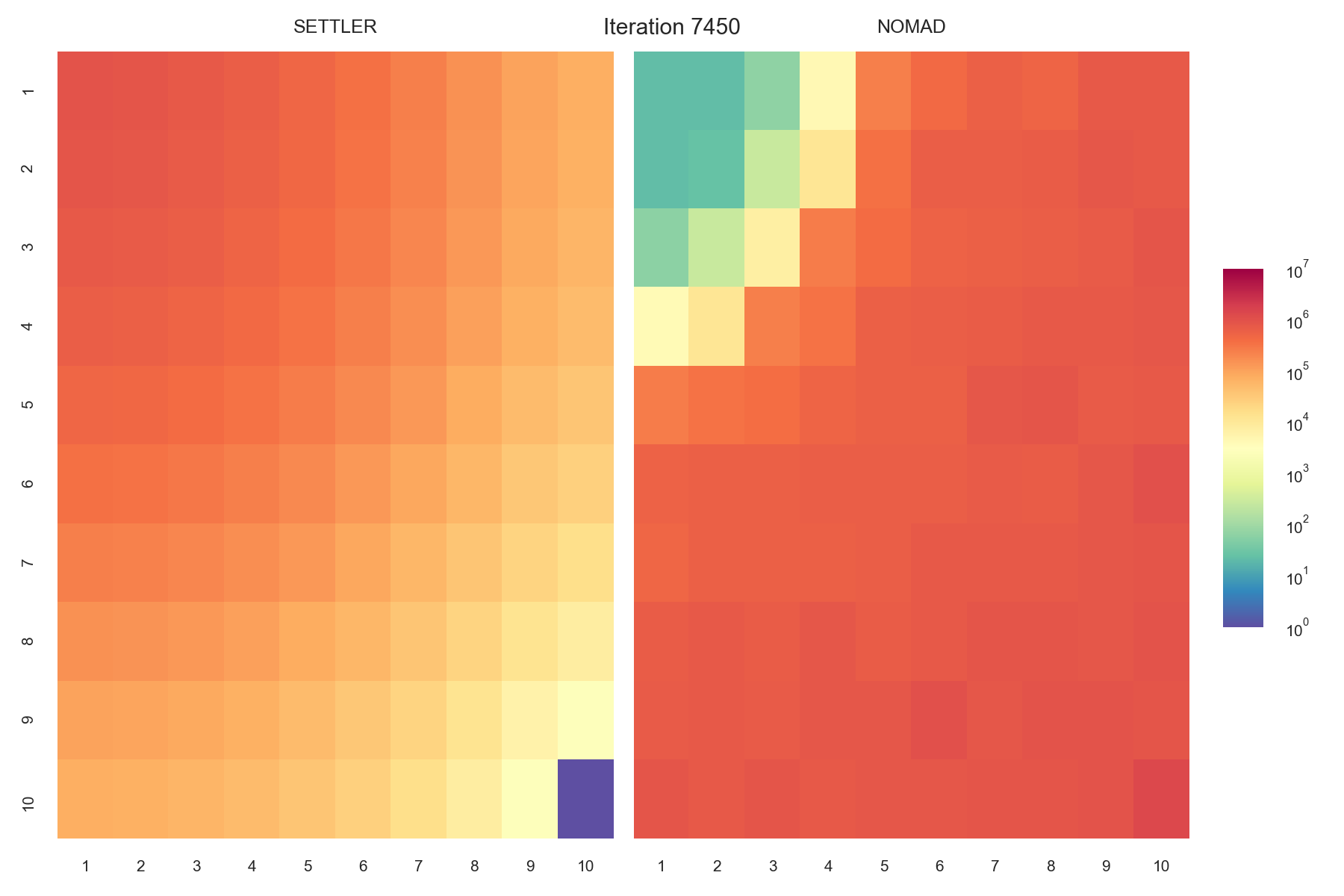

Supplement: Supplementary file 1 [file biology-10-01019-s001.zip › Spatio-temporal dynamics heatmaps/chempenoff_extremelyscarce_lindeath_period1000/7450.png]

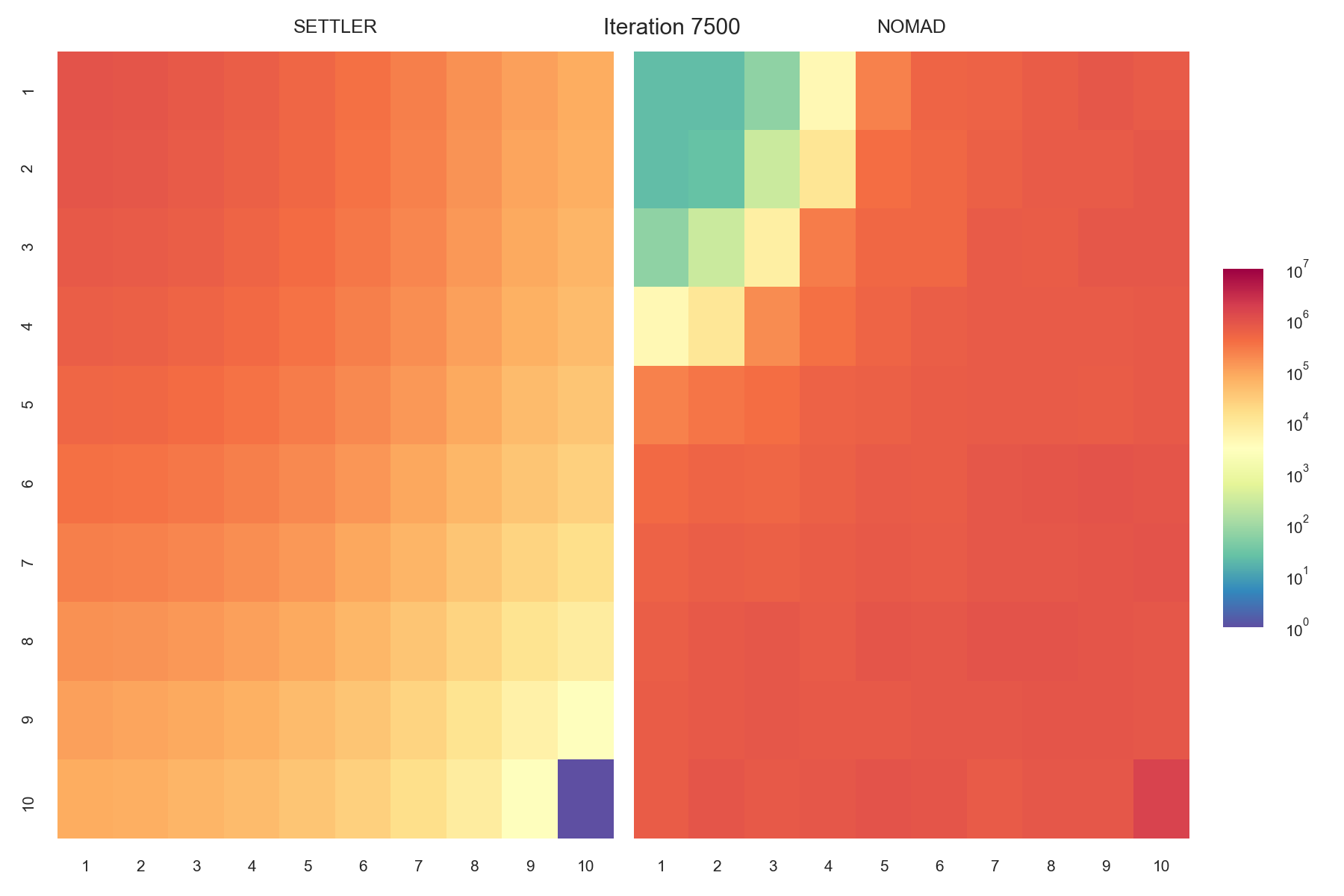

Supplement: Supplementary file 1 [file biology-10-01019-s001.zip › Spatio-temporal dynamics heatmaps/chempenoff_extremelyscarce_lindeath_period1000/7500.png]

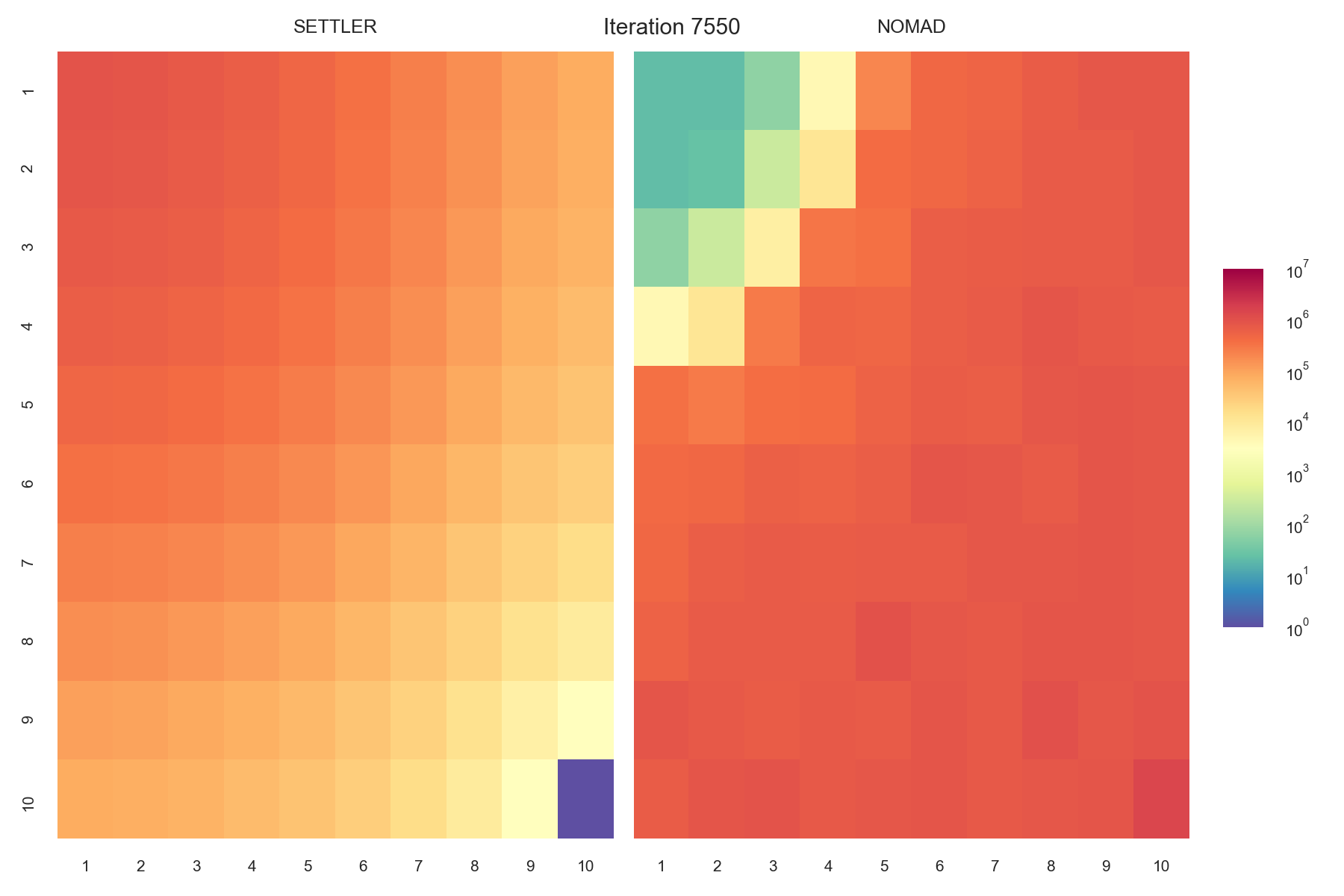

Supplement: Supplementary file 1 [file biology-10-01019-s001.zip › Spatio-temporal dynamics heatmaps/chempenoff_extremelyscarce_lindeath_period1000/7550.png]

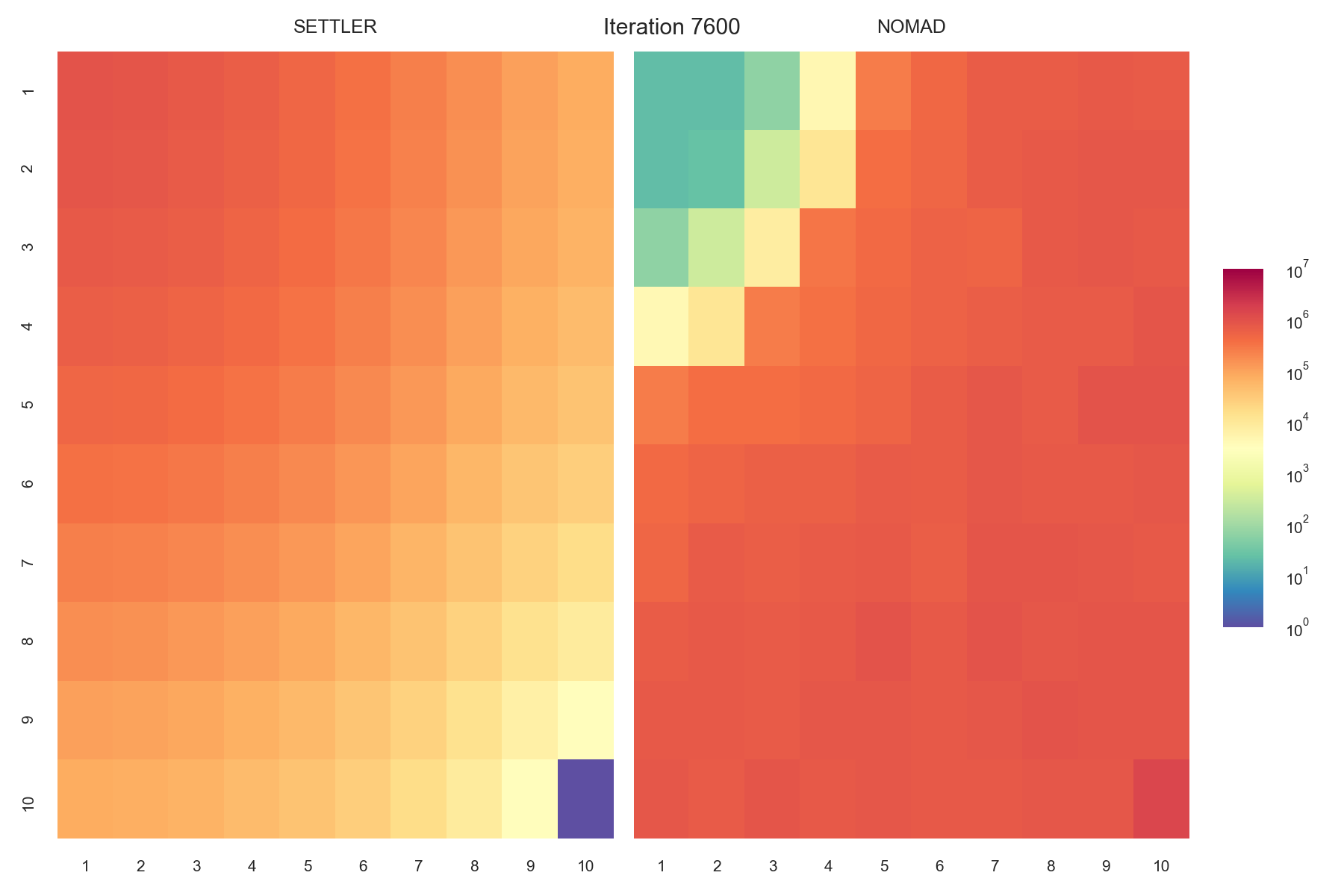

Supplement: Supplementary file 1 [file biology-10-01019-s001.zip › Spatio-temporal dynamics heatmaps/chempenoff_extremelyscarce_lindeath_period1000/7600.png]

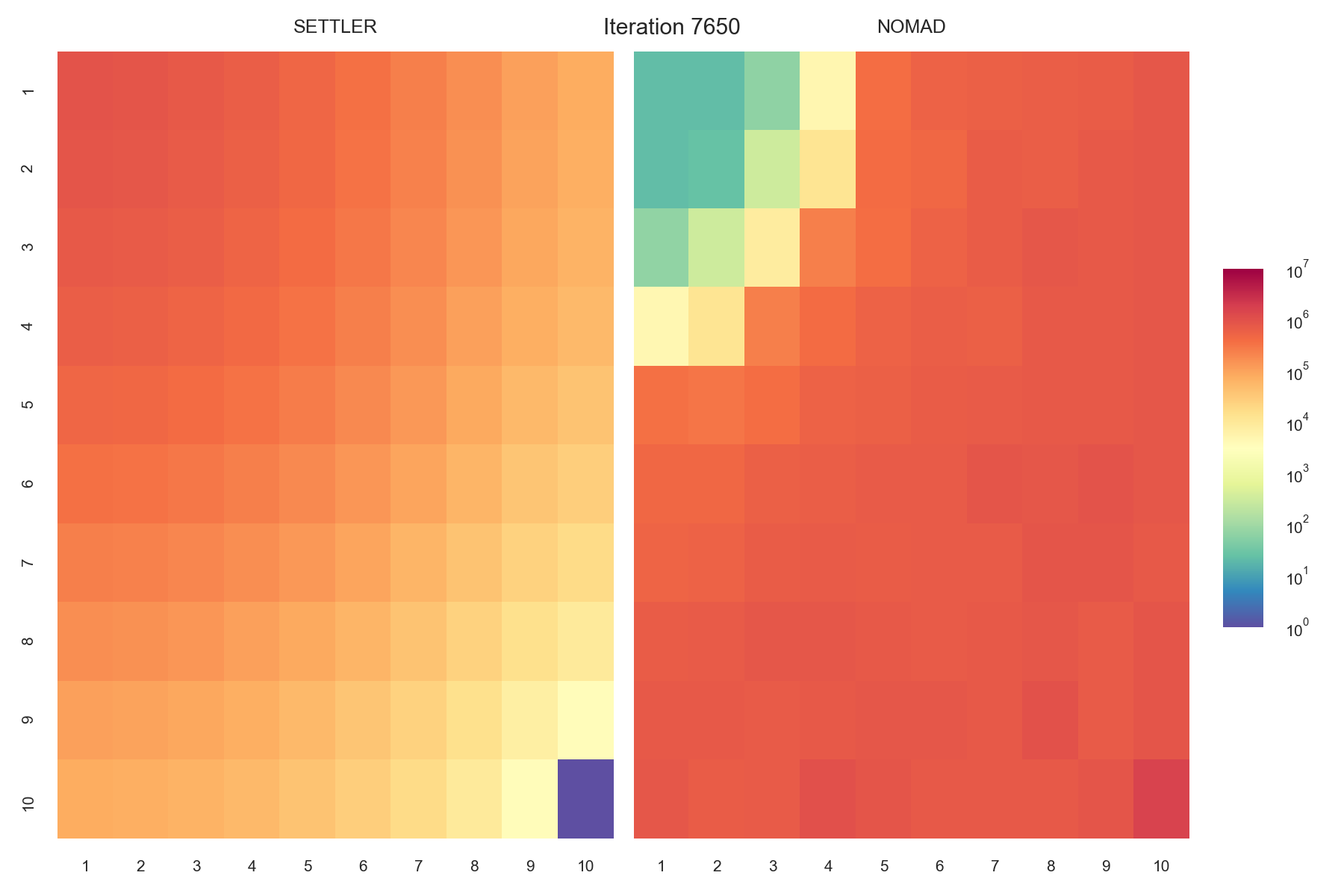

Supplement: Supplementary file 1 [file biology-10-01019-s001.zip › Spatio-temporal dynamics heatmaps/chempenoff_extremelyscarce_lindeath_period1000/7650.png]

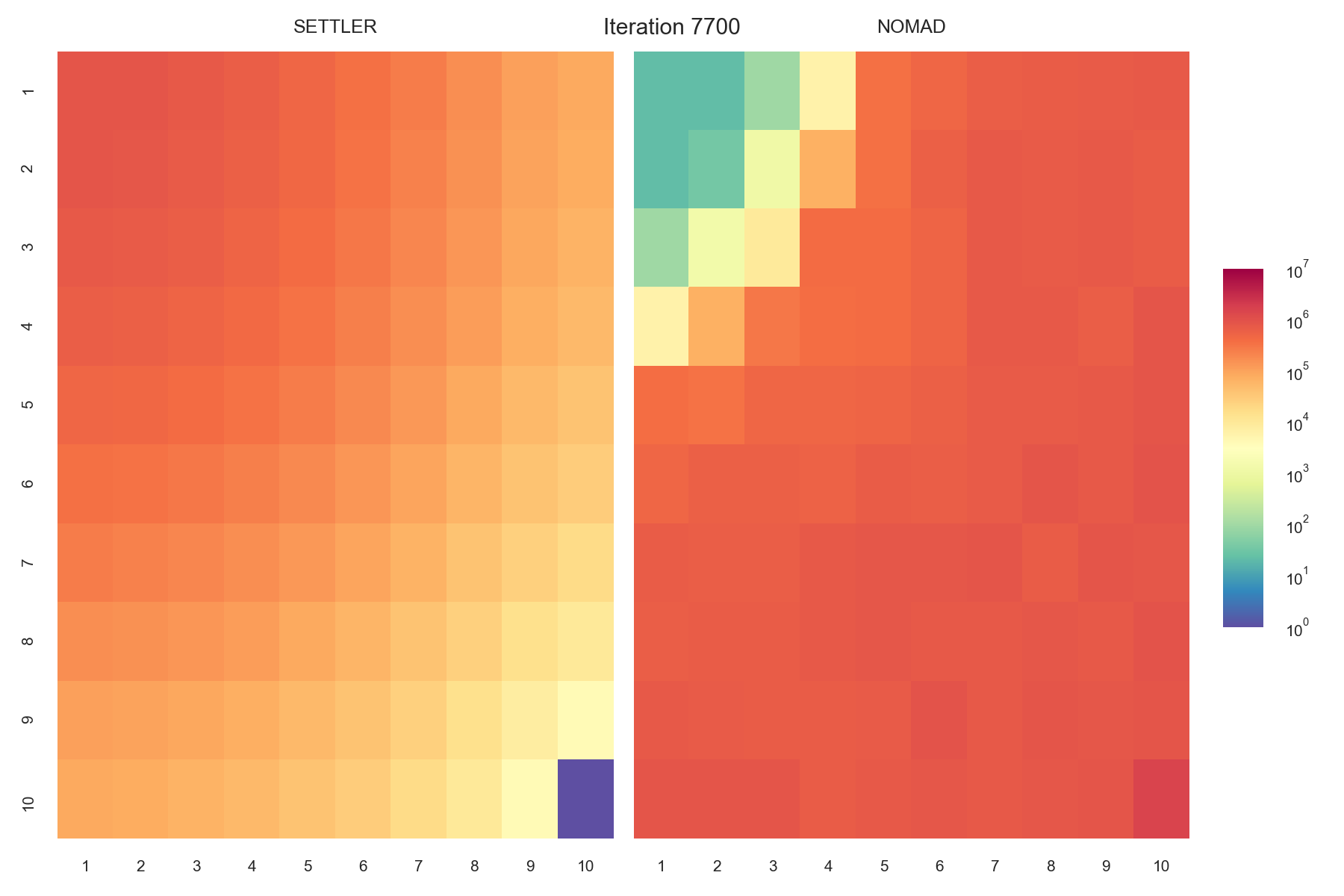

Supplement: Supplementary file 1 [file biology-10-01019-s001.zip › Spatio-temporal dynamics heatmaps/chempenoff_extremelyscarce_lindeath_period1000/7700.png]

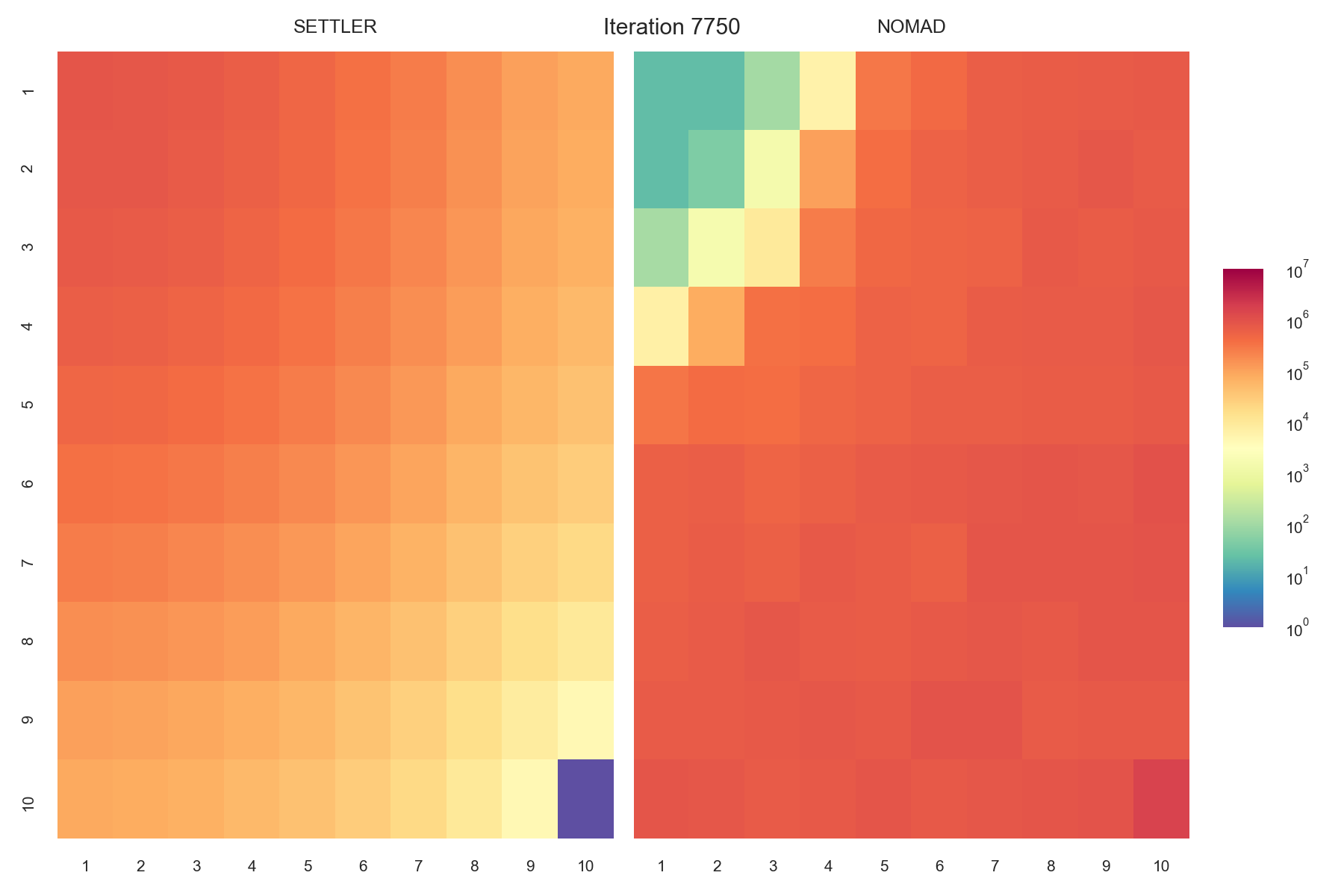

Supplement: Supplementary file 1 [file biology-10-01019-s001.zip › Spatio-temporal dynamics heatmaps/chempenoff_extremelyscarce_lindeath_period1000/7750.png]

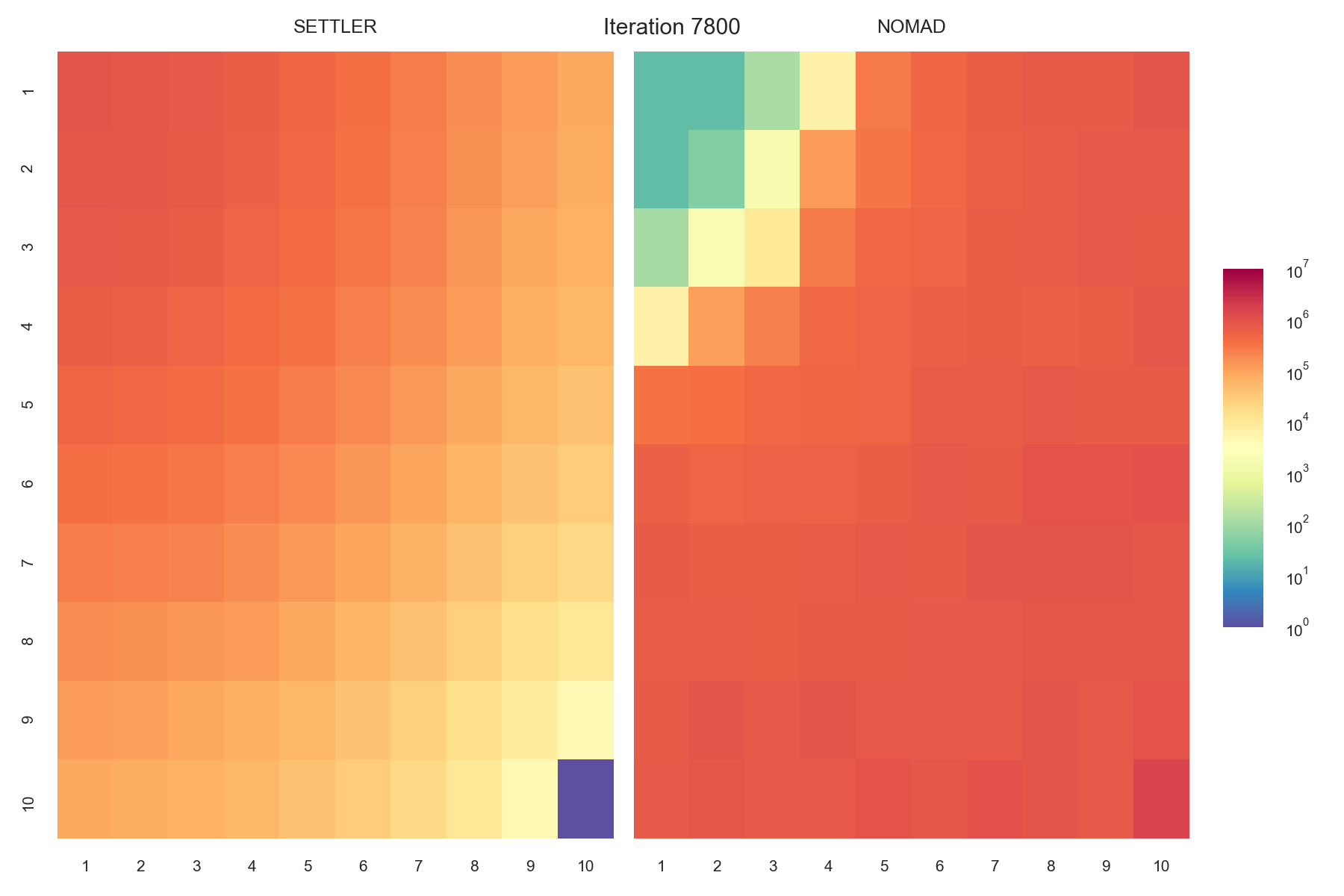

Supplement: Supplementary file 1 [file biology-10-01019-s001.zip › Spatio-temporal dynamics heatmaps/chempenoff_extremelyscarce_lindeath_period1000/7800.png]

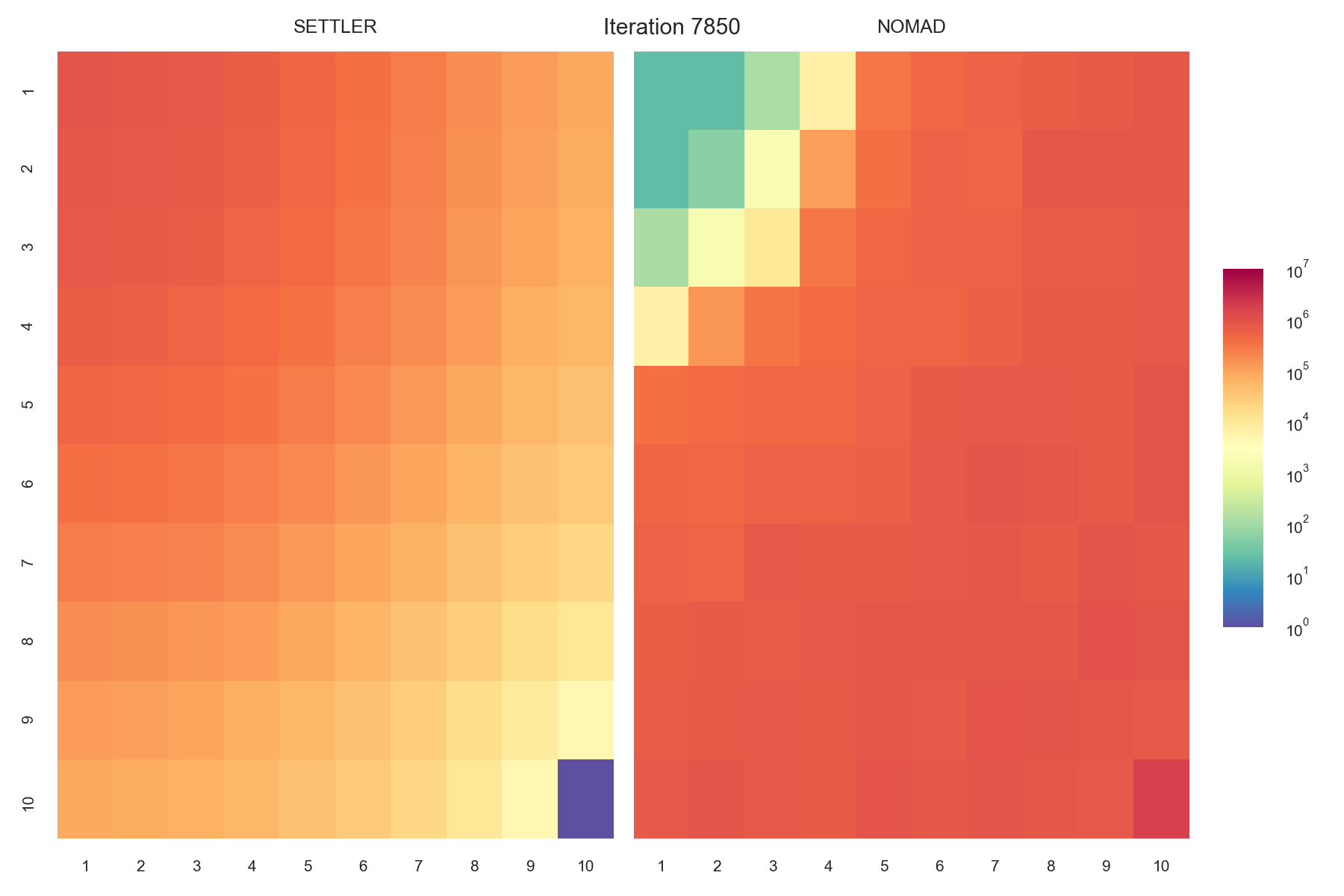

Supplement: Supplementary file 1 [file biology-10-01019-s001.zip › Spatio-temporal dynamics heatmaps/chempenoff_extremelyscarce_lindeath_period1000/7850.png]

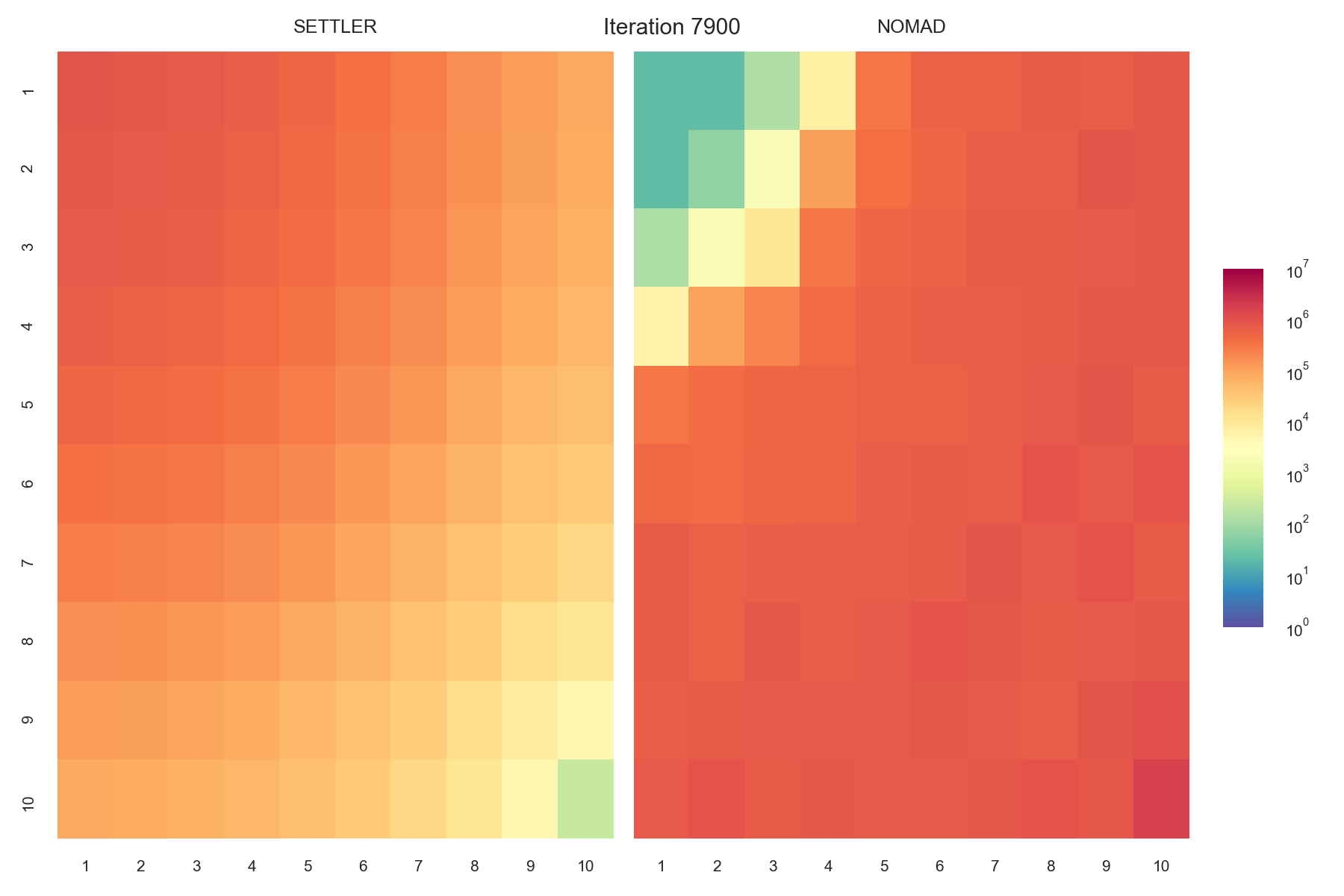

Supplement: Supplementary file 1 [file biology-10-01019-s001.zip › Spatio-temporal dynamics heatmaps/chempenoff_extremelyscarce_lindeath_period1000/7900.png]

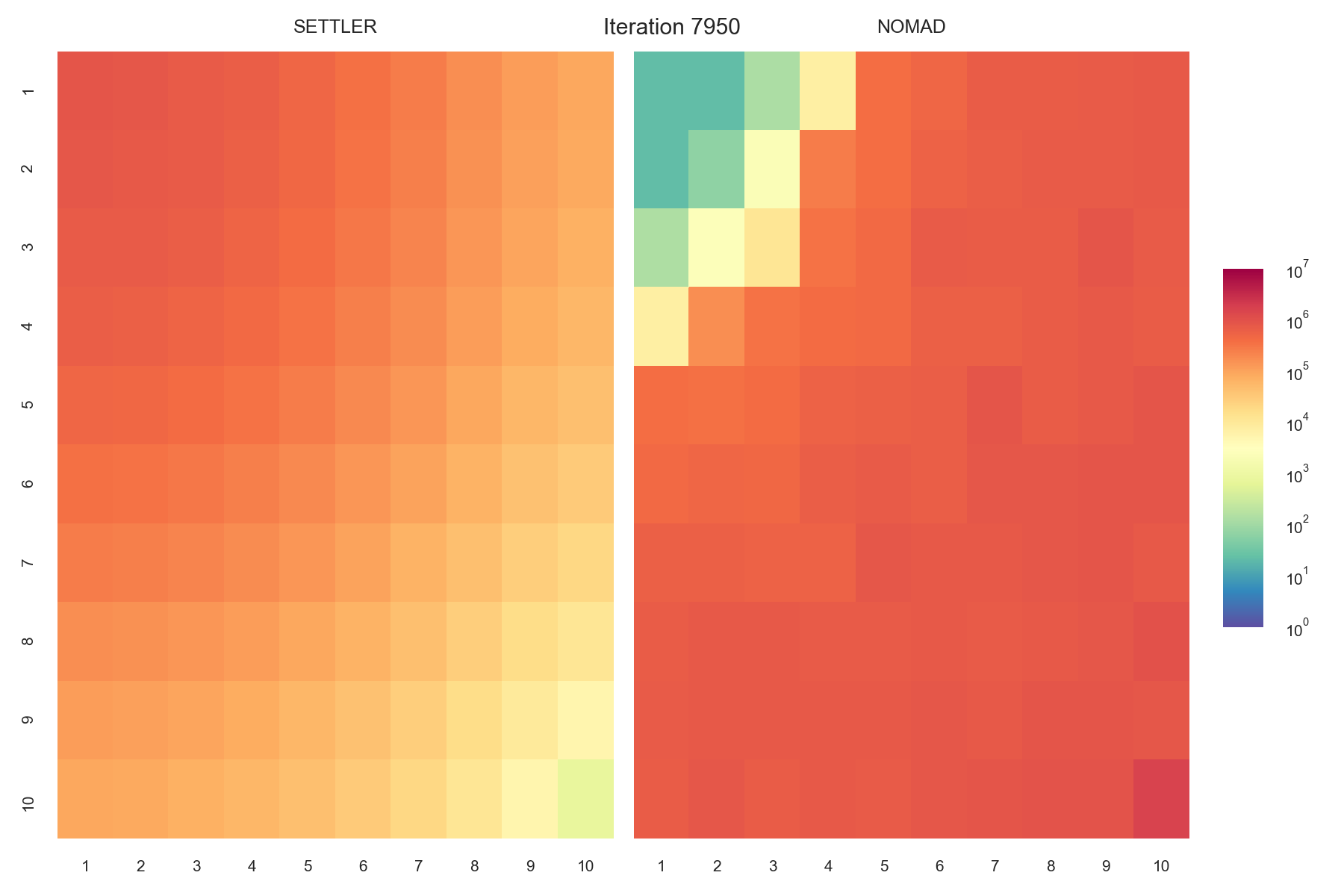

Supplement: Supplementary file 1 [file biology-10-01019-s001.zip › Spatio-temporal dynamics heatmaps/chempenoff_extremelyscarce_lindeath_period1000/7950.png]

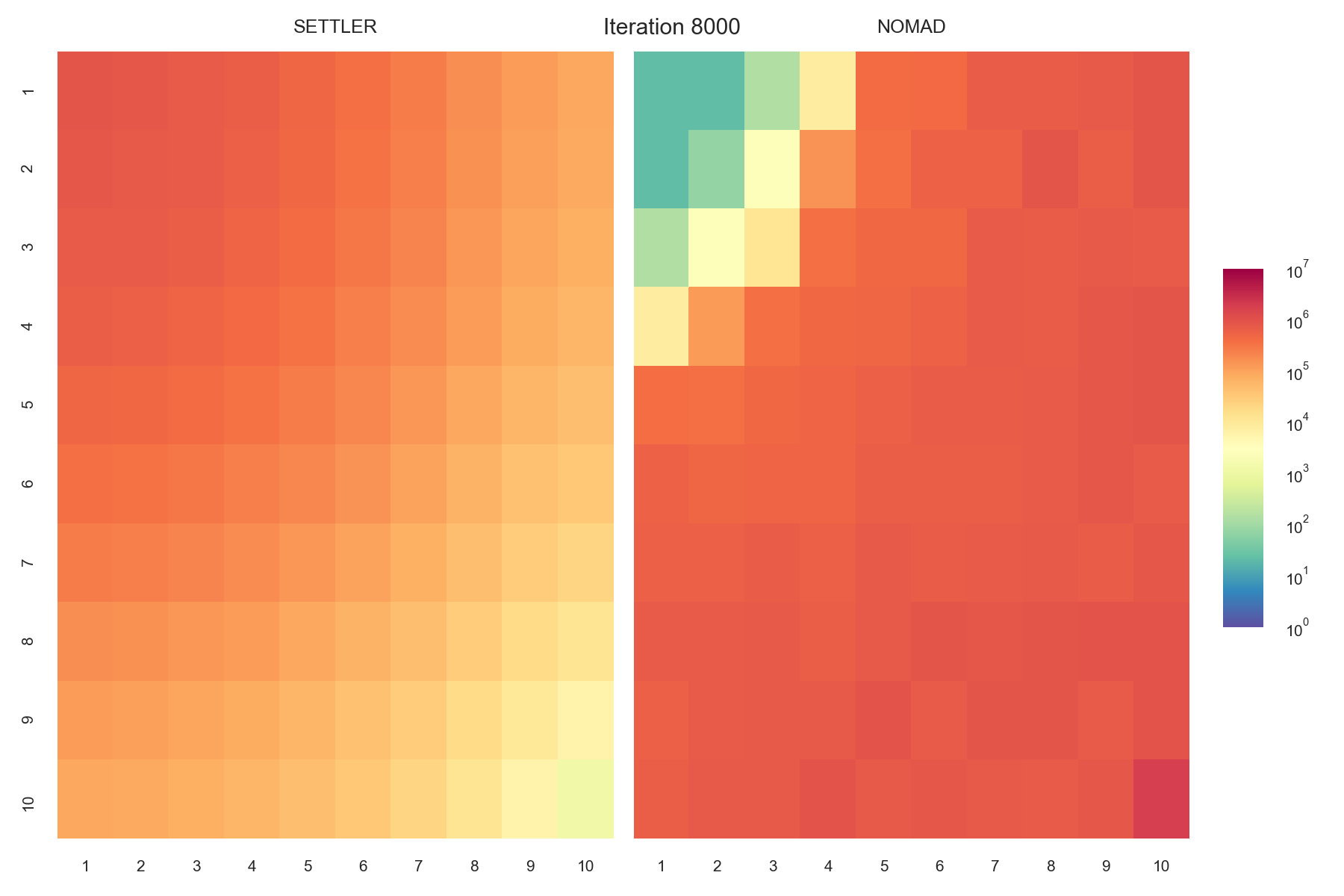

Supplement: Supplementary file 1 [file biology-10-01019-s001.zip › Spatio-temporal dynamics heatmaps/chempenoff_extremelyscarce_lindeath_period1000/8000.png]

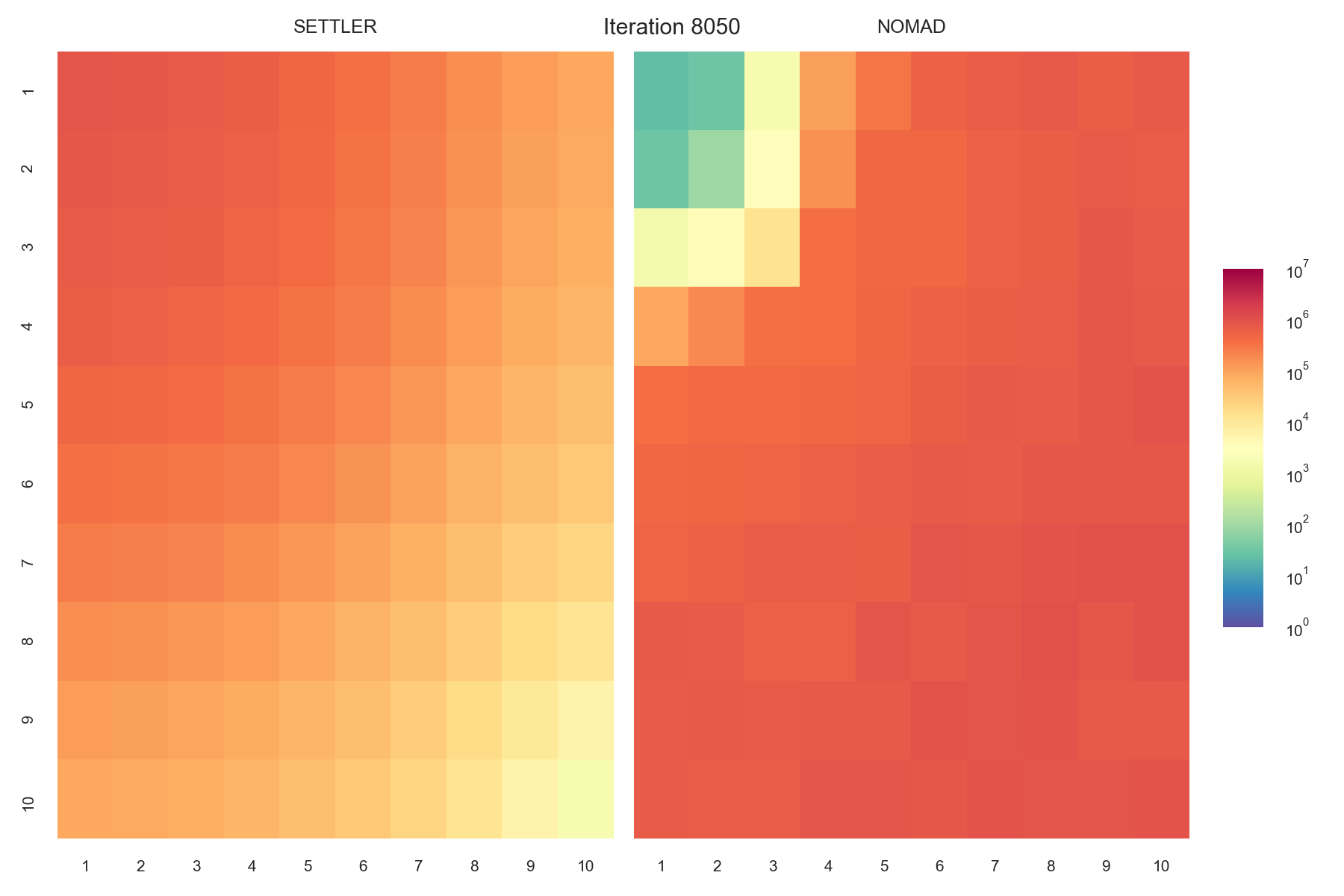

Supplement: Supplementary file 1 [file biology-10-01019-s001.zip › Spatio-temporal dynamics heatmaps/chempenoff_extremelyscarce_lindeath_period1000/8050.png]

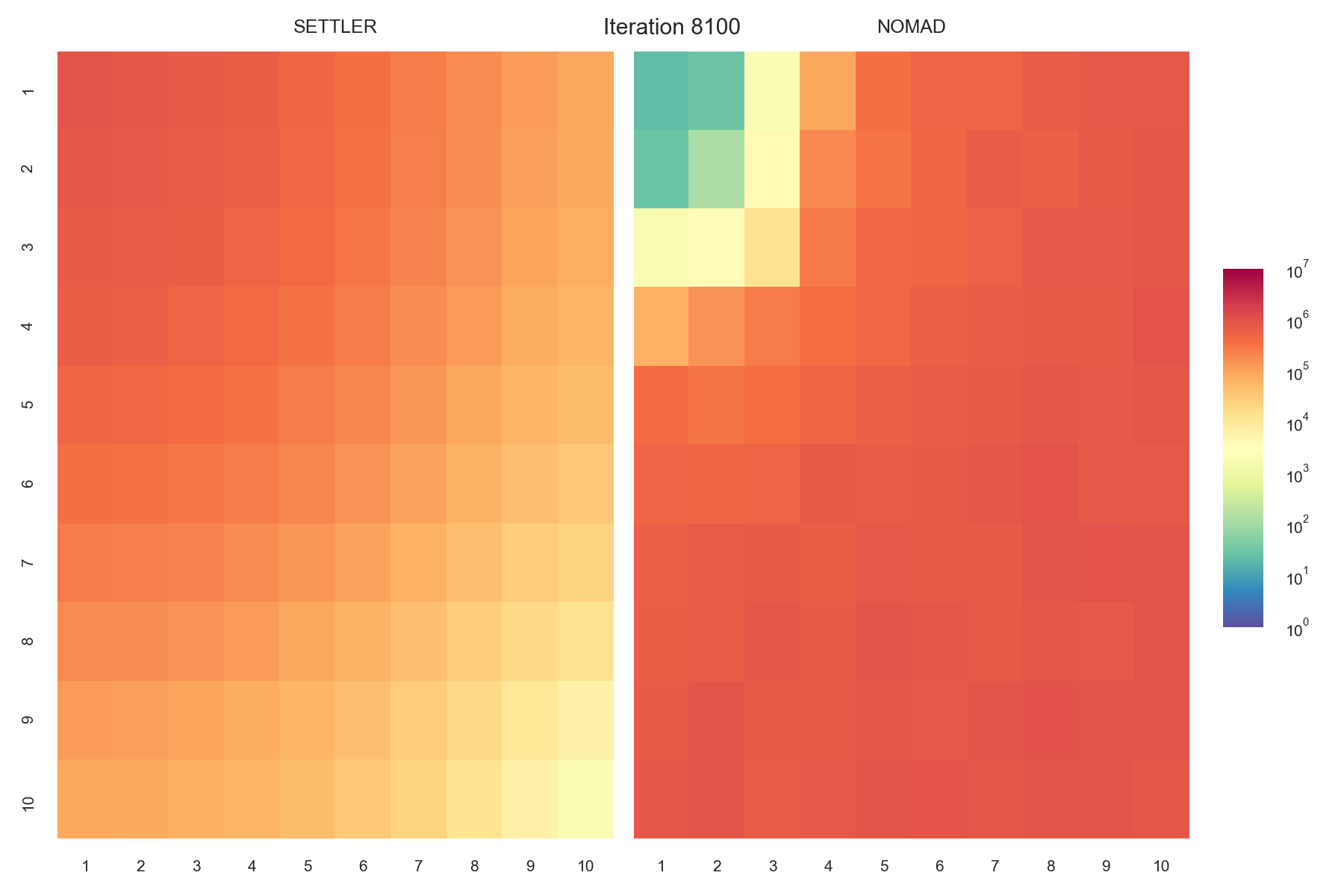

Supplement: Supplementary file 1 [file biology-10-01019-s001.zip › Spatio-temporal dynamics heatmaps/chempenoff_extremelyscarce_lindeath_period1000/8100.png]

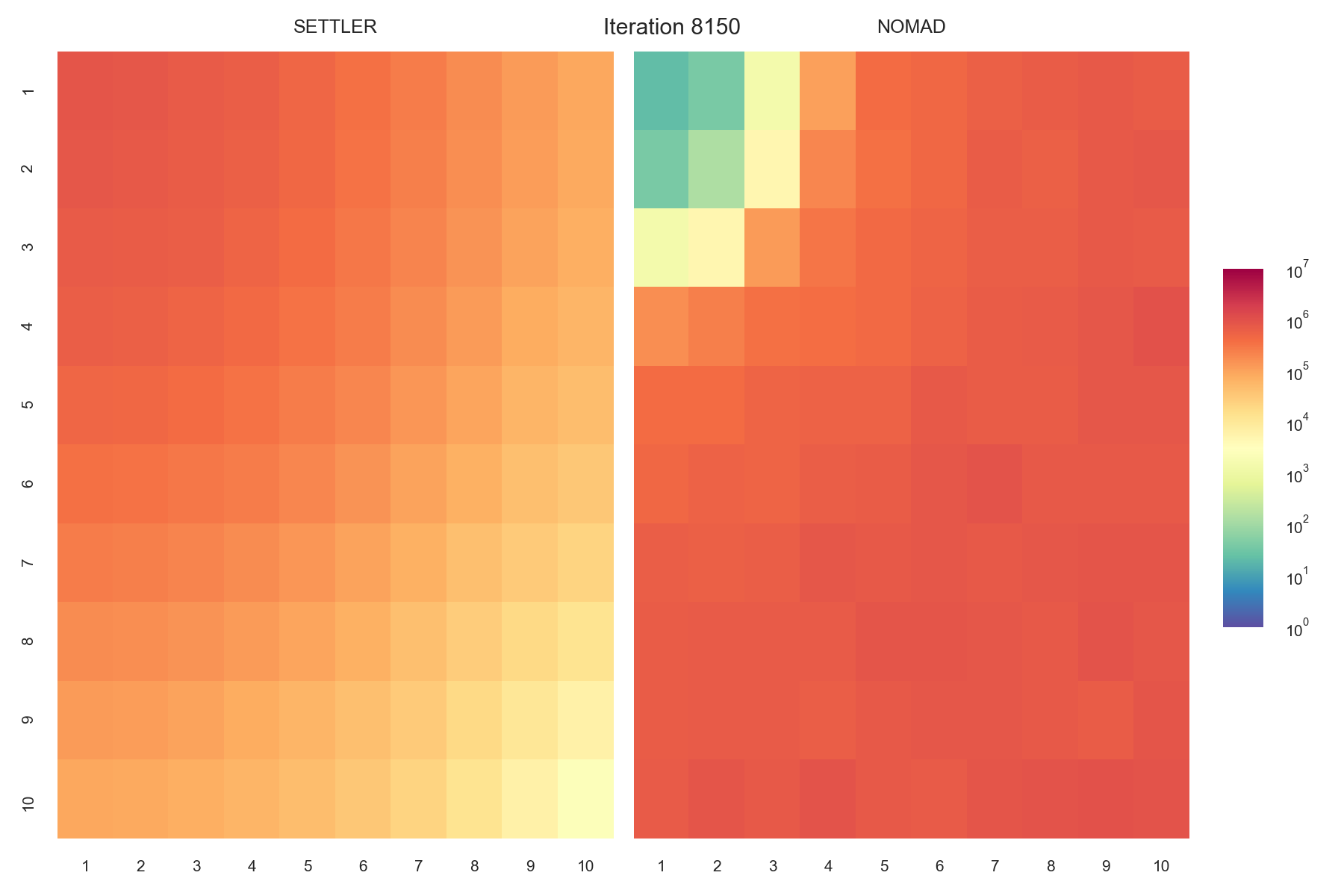

Supplement: Supplementary file 1 [file biology-10-01019-s001.zip › Spatio-temporal dynamics heatmaps/chempenoff_extremelyscarce_lindeath_period1000/8150.png]

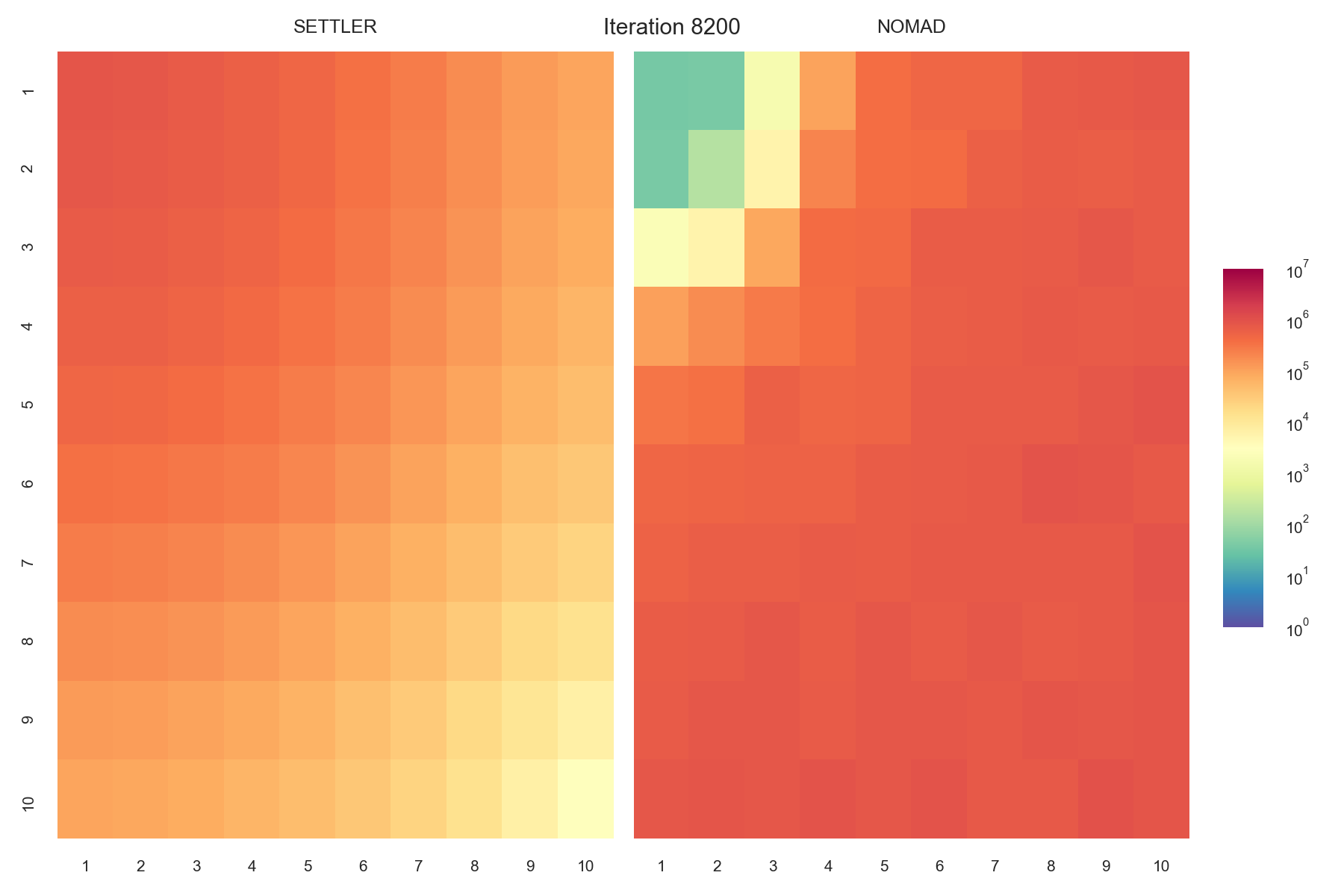

Supplement: Supplementary file 1 [file biology-10-01019-s001.zip › Spatio-temporal dynamics heatmaps/chempenoff_extremelyscarce_lindeath_period1000/8200.png]

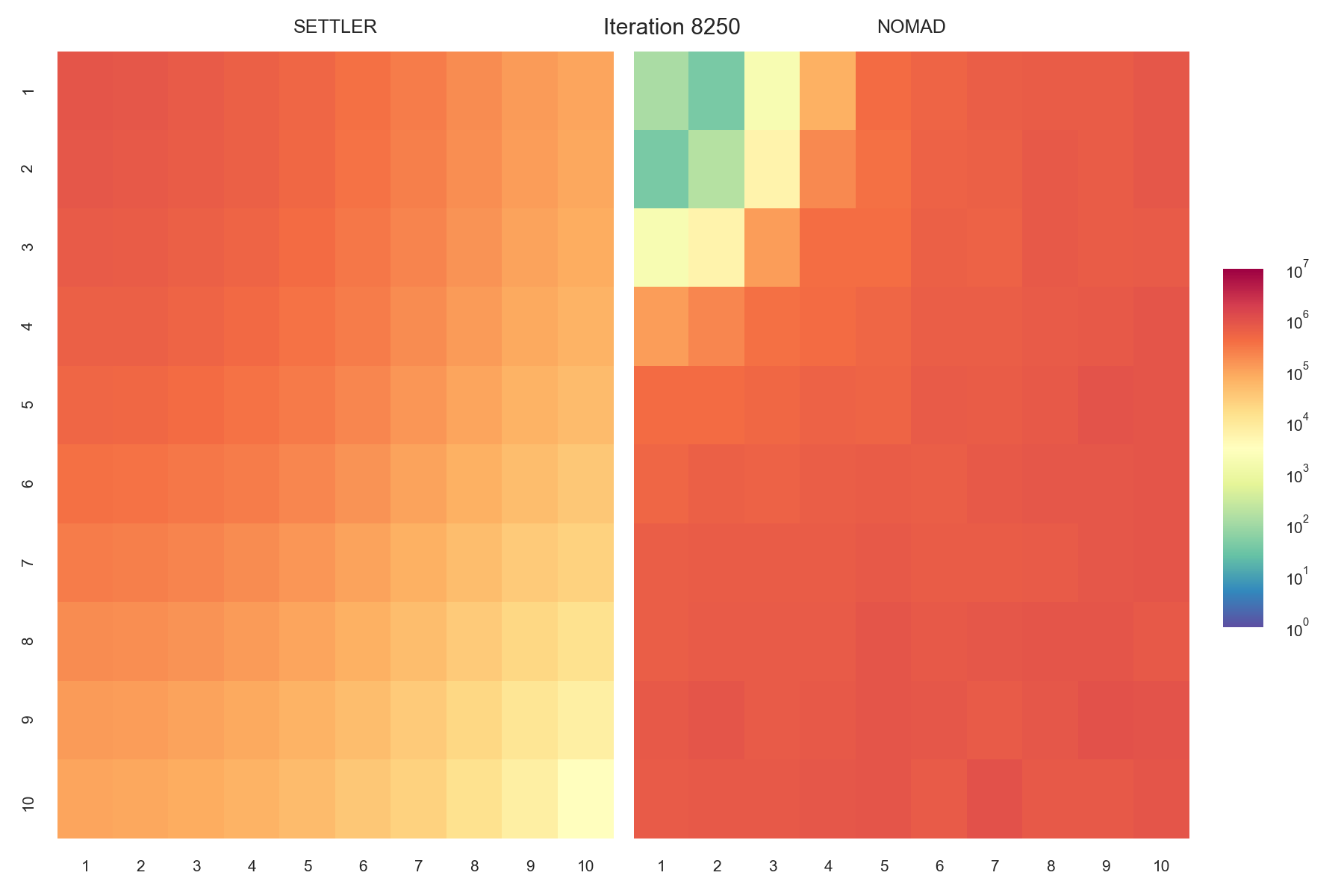

Supplement: Supplementary file 1 [file biology-10-01019-s001.zip › Spatio-temporal dynamics heatmaps/chempenoff_extremelyscarce_lindeath_period1000/8250.png]

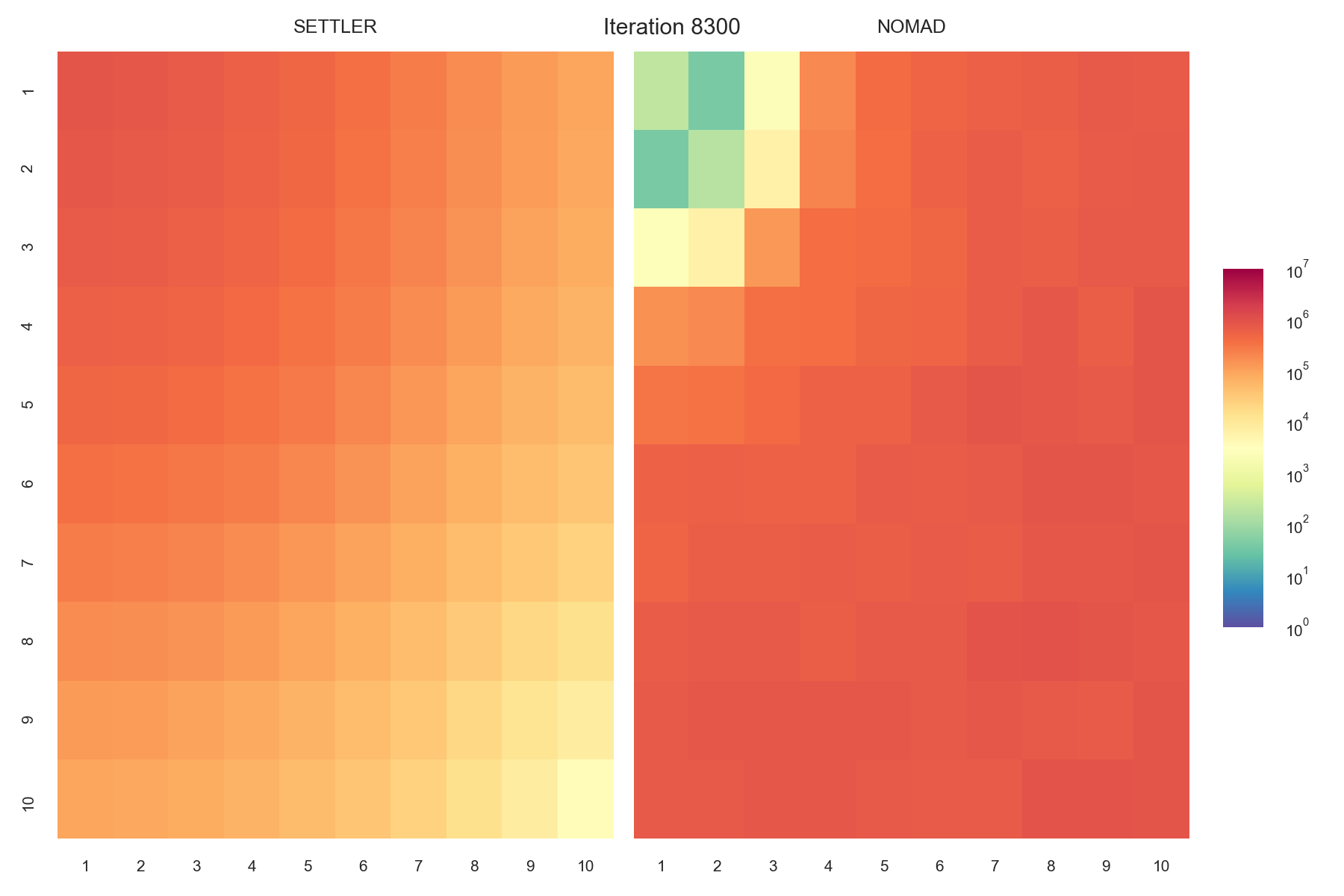

Supplement: Supplementary file 1 [file biology-10-01019-s001.zip › Spatio-temporal dynamics heatmaps/chempenoff_extremelyscarce_lindeath_period1000/8300.png]

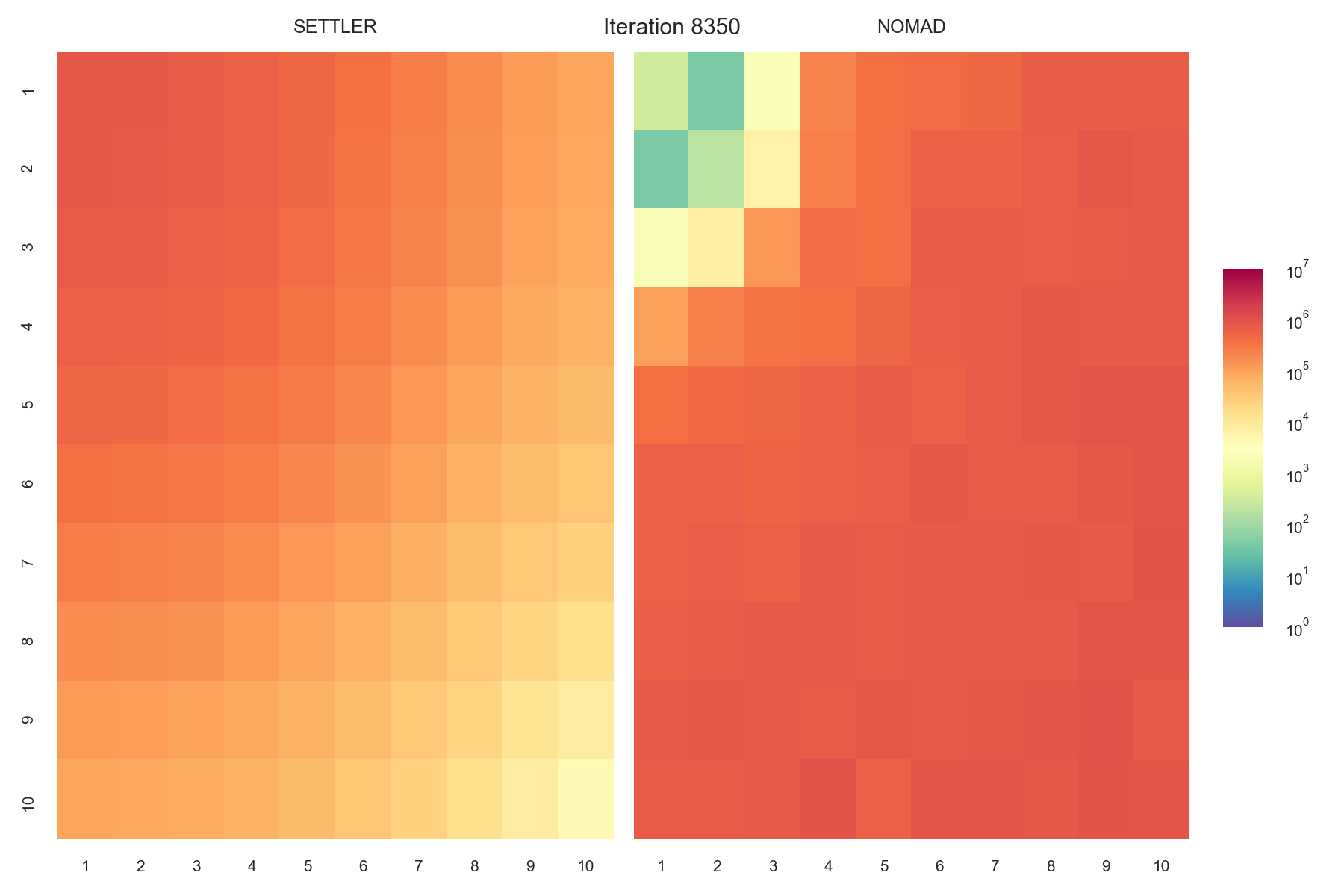

Supplement: Supplementary file 1 [file biology-10-01019-s001.zip › Spatio-temporal dynamics heatmaps/chempenoff_extremelyscarce_lindeath_period1000/8350.png]

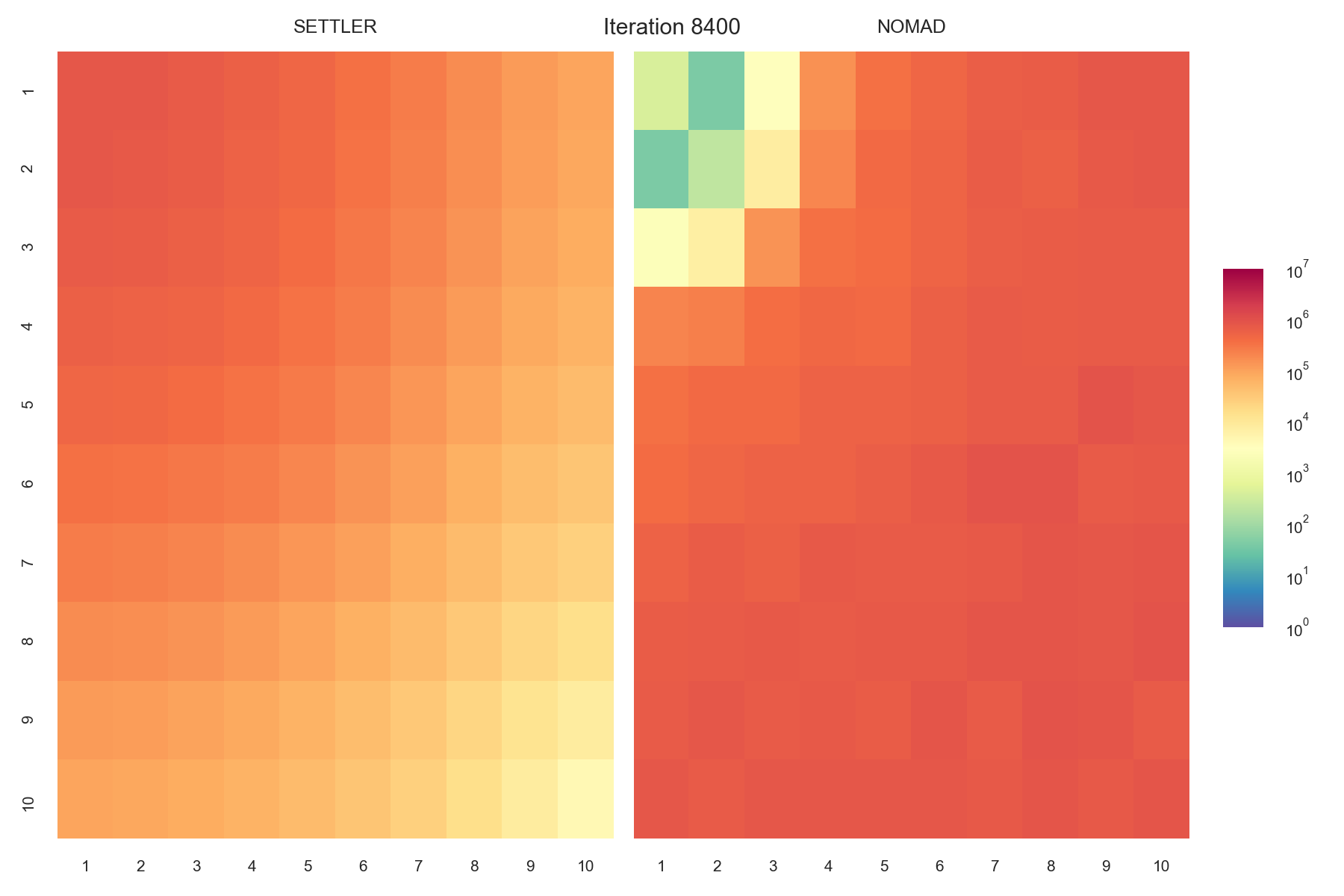

Supplement: Supplementary file 1 [file biology-10-01019-s001.zip › Spatio-temporal dynamics heatmaps/chempenoff_extremelyscarce_lindeath_period1000/8400.png]

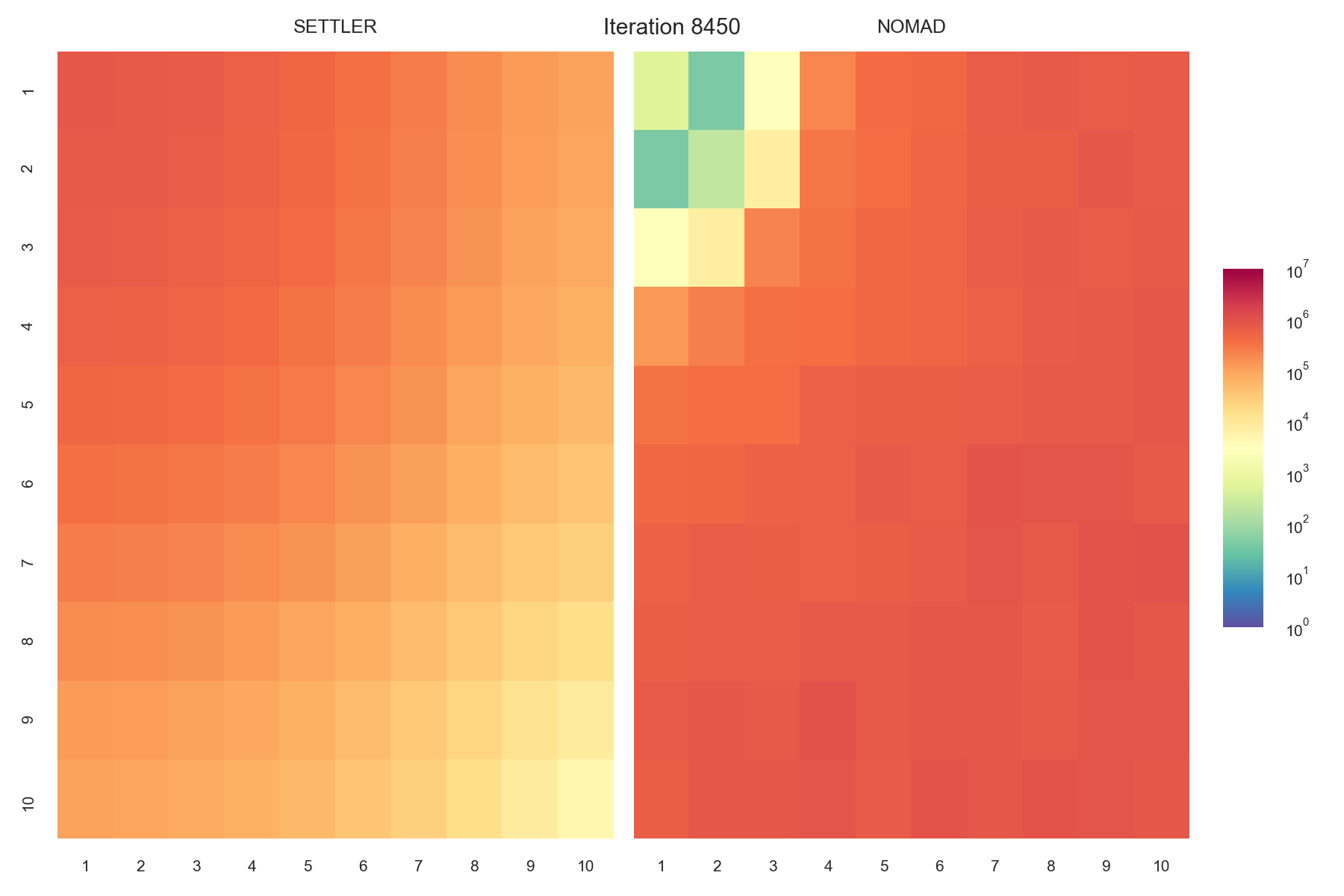

Supplement: Supplementary file 1 [file biology-10-01019-s001.zip › Spatio-temporal dynamics heatmaps/chempenoff_extremelyscarce_lindeath_period1000/8450.png]

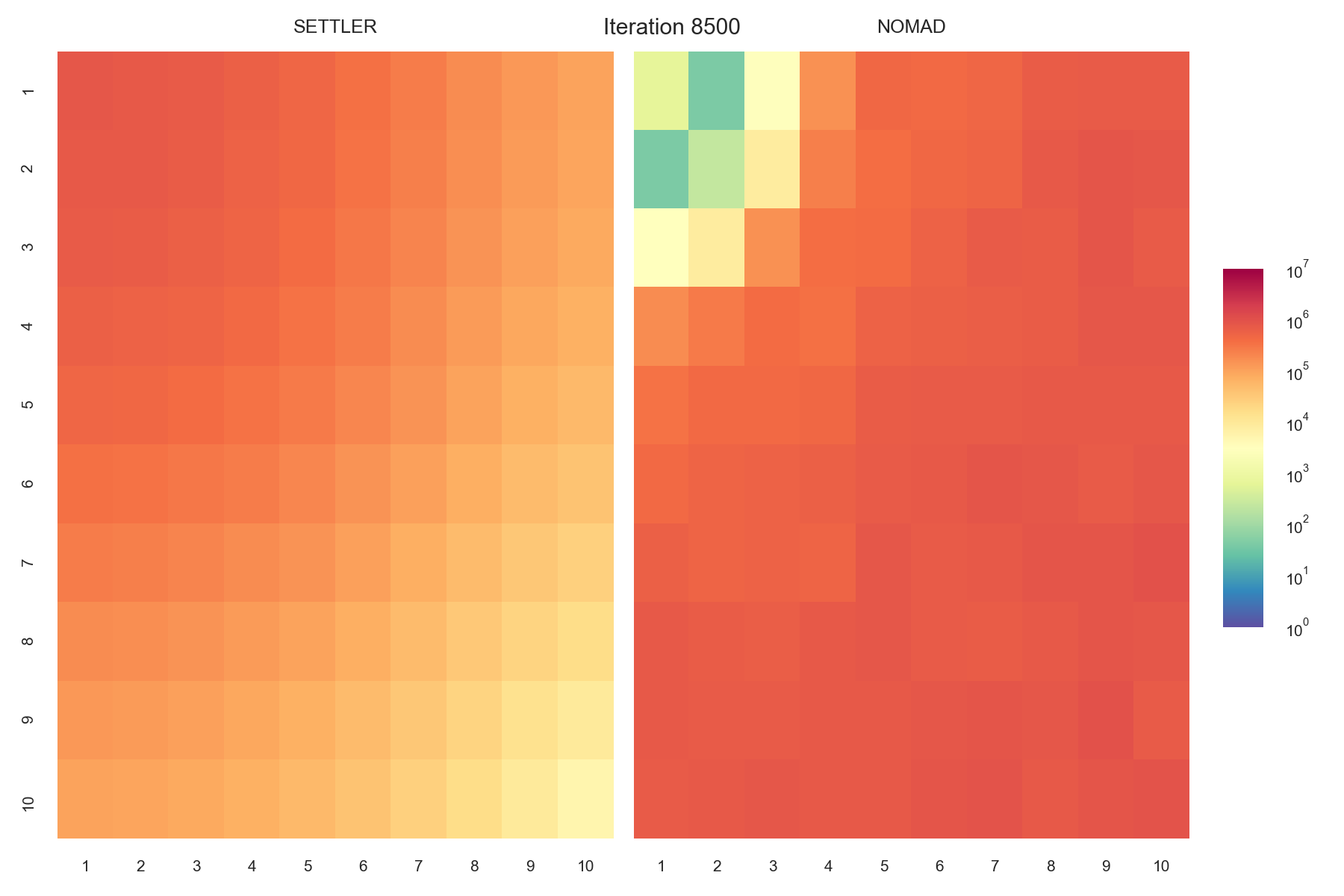

Supplement: Supplementary file 1 [file biology-10-01019-s001.zip › Spatio-temporal dynamics heatmaps/chempenoff_extremelyscarce_lindeath_period1000/8500.png]

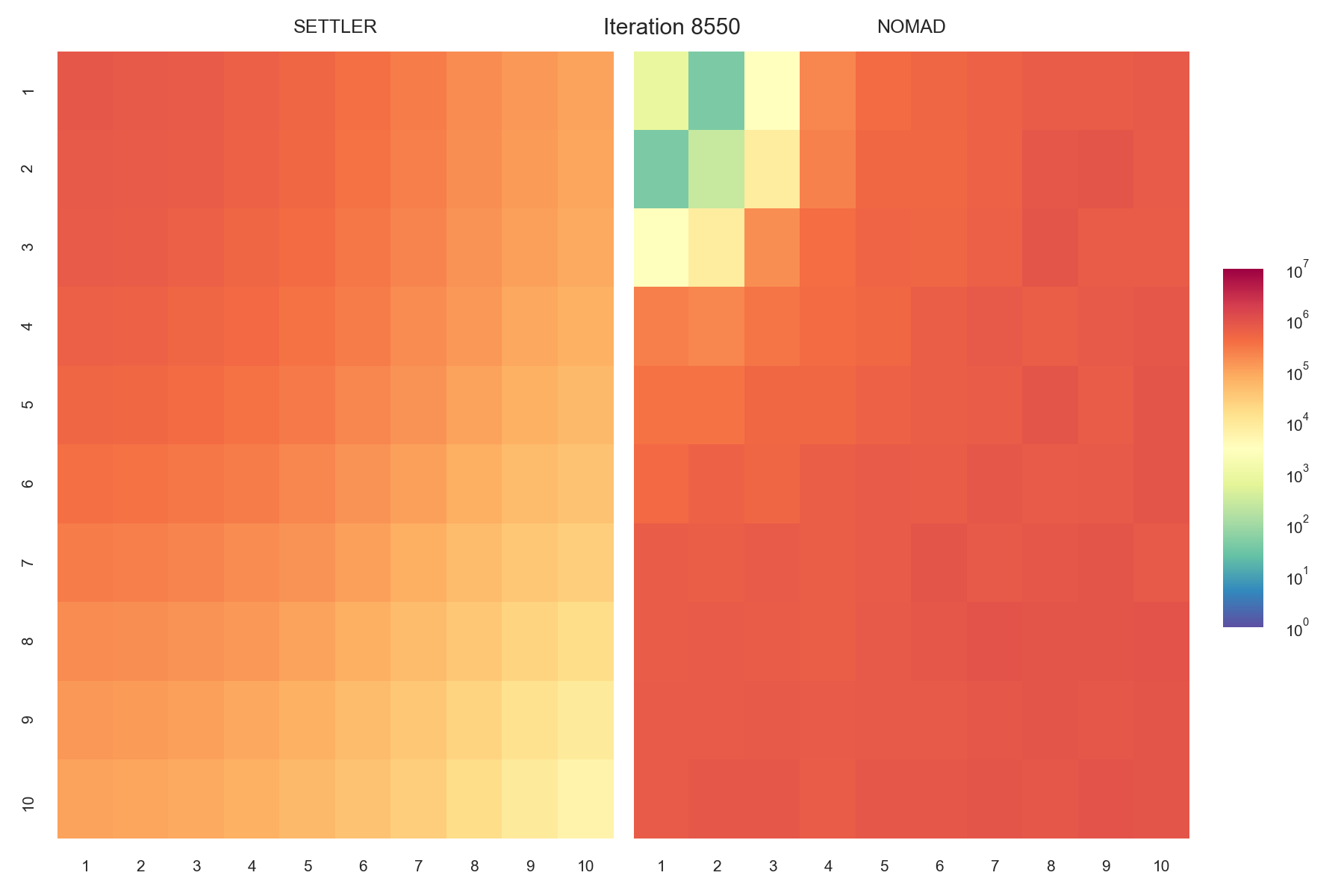

Supplement: Supplementary file 1 [file biology-10-01019-s001.zip › Spatio-temporal dynamics heatmaps/chempenoff_extremelyscarce_lindeath_period1000/8550.png]

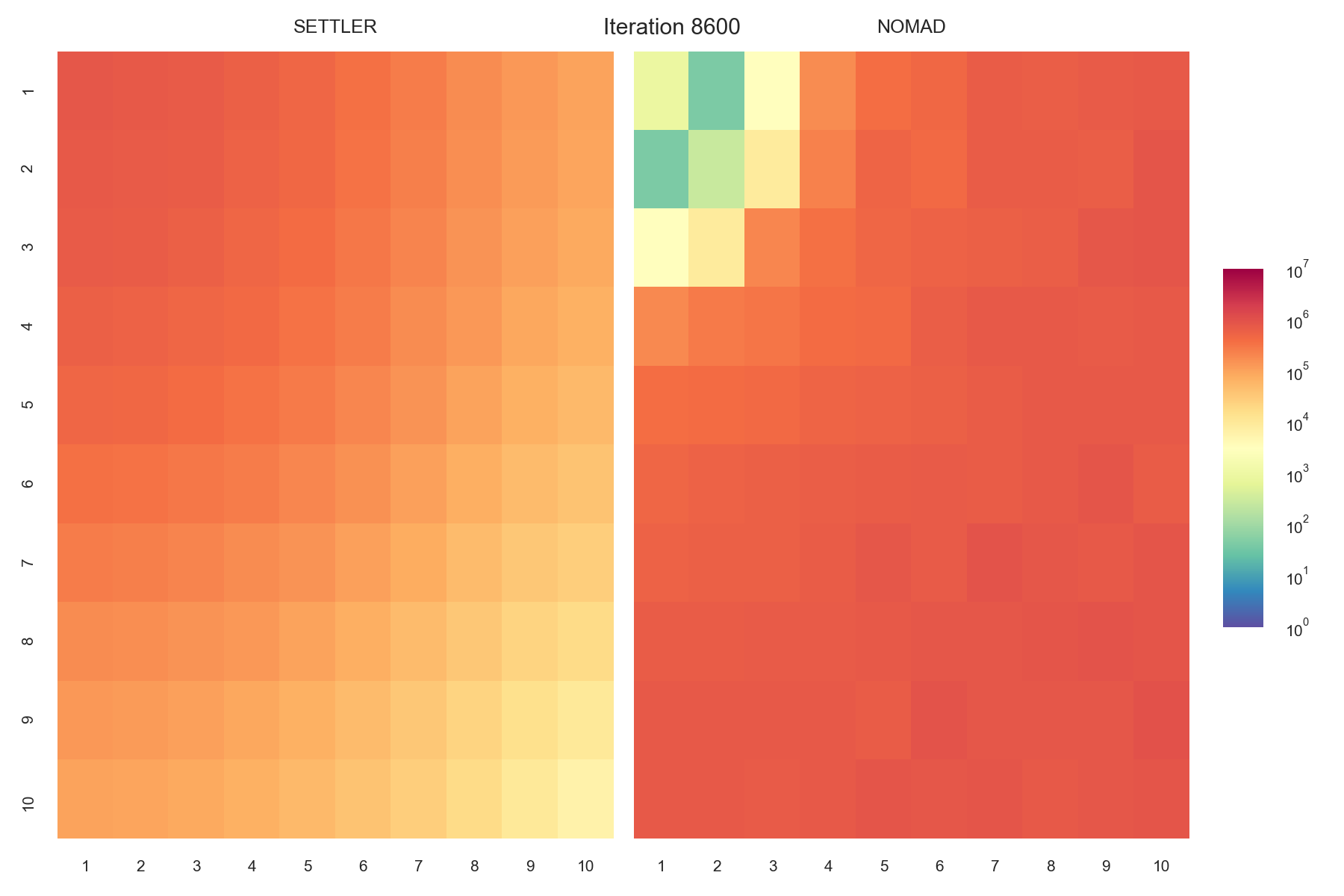

Supplement: Supplementary file 1 [file biology-10-01019-s001.zip › Spatio-temporal dynamics heatmaps/chempenoff_extremelyscarce_lindeath_period1000/8600.png]

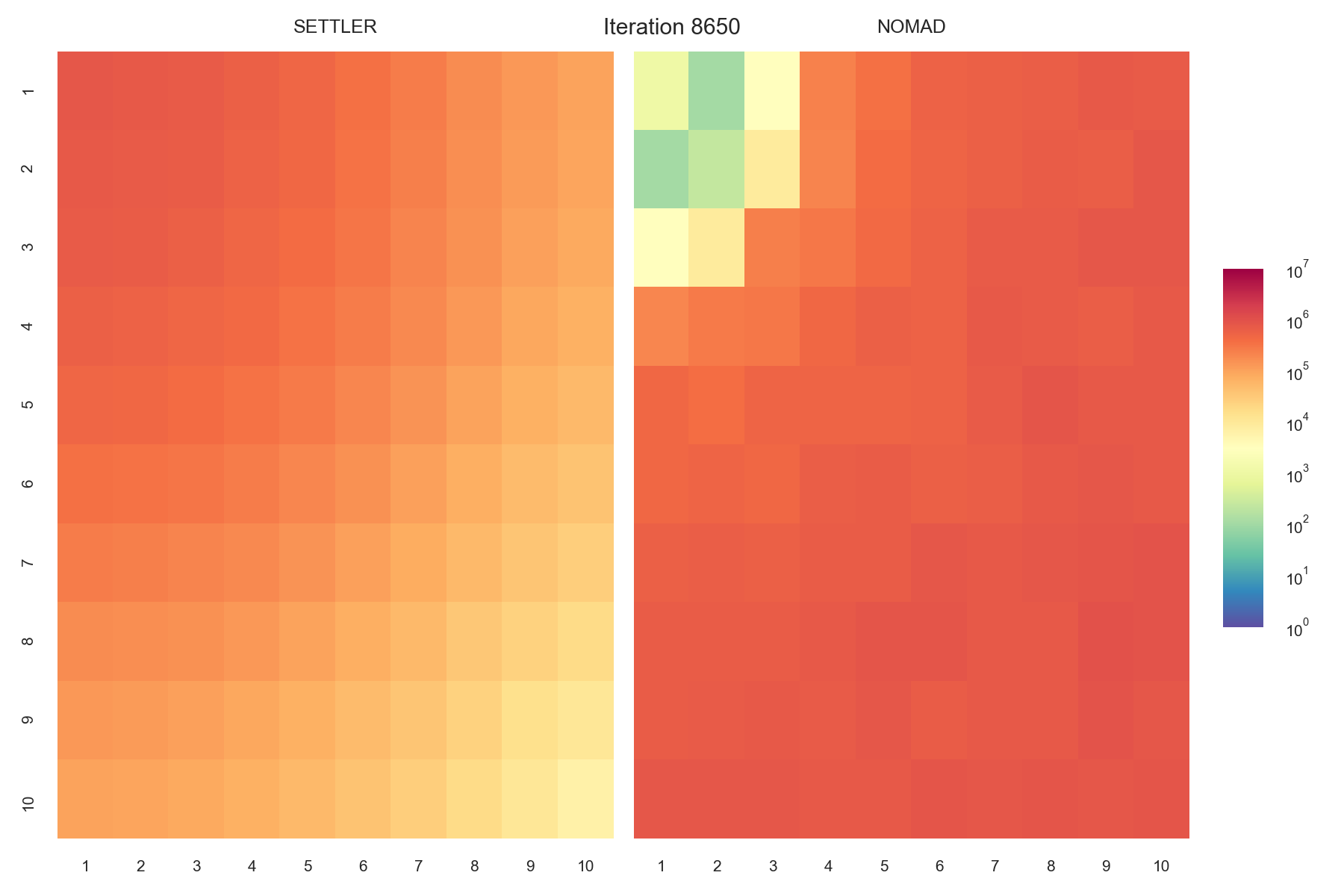

Supplement: Supplementary file 1 [file biology-10-01019-s001.zip › Spatio-temporal dynamics heatmaps/chempenoff_extremelyscarce_lindeath_period1000/8650.png]

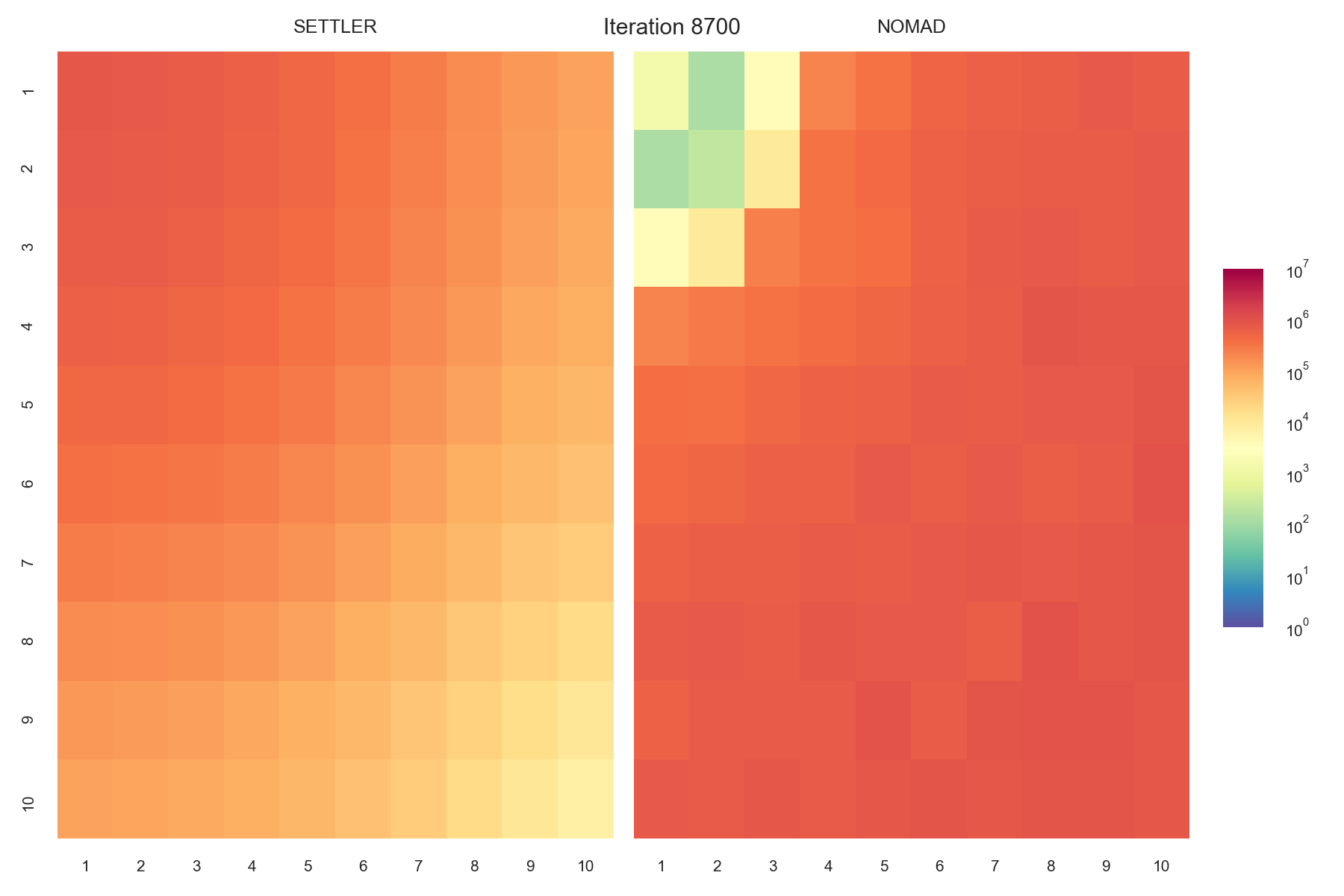

Supplement: Supplementary file 1 [file biology-10-01019-s001.zip › Spatio-temporal dynamics heatmaps/chempenoff_extremelyscarce_lindeath_period1000/8700.png]

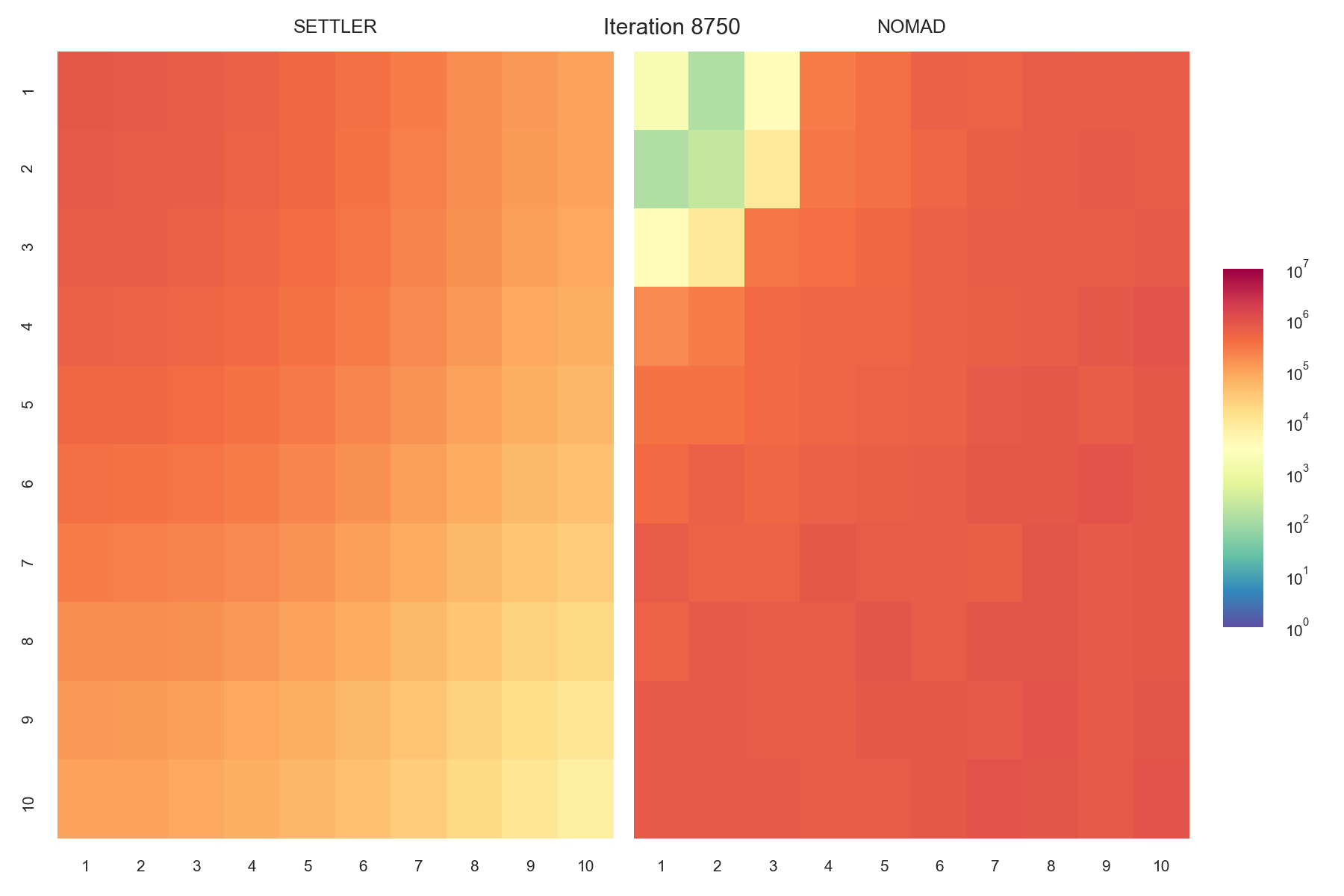

Supplement: Supplementary file 1 [file biology-10-01019-s001.zip › Spatio-temporal dynamics heatmaps/chempenoff_extremelyscarce_lindeath_period1000/8750.png]

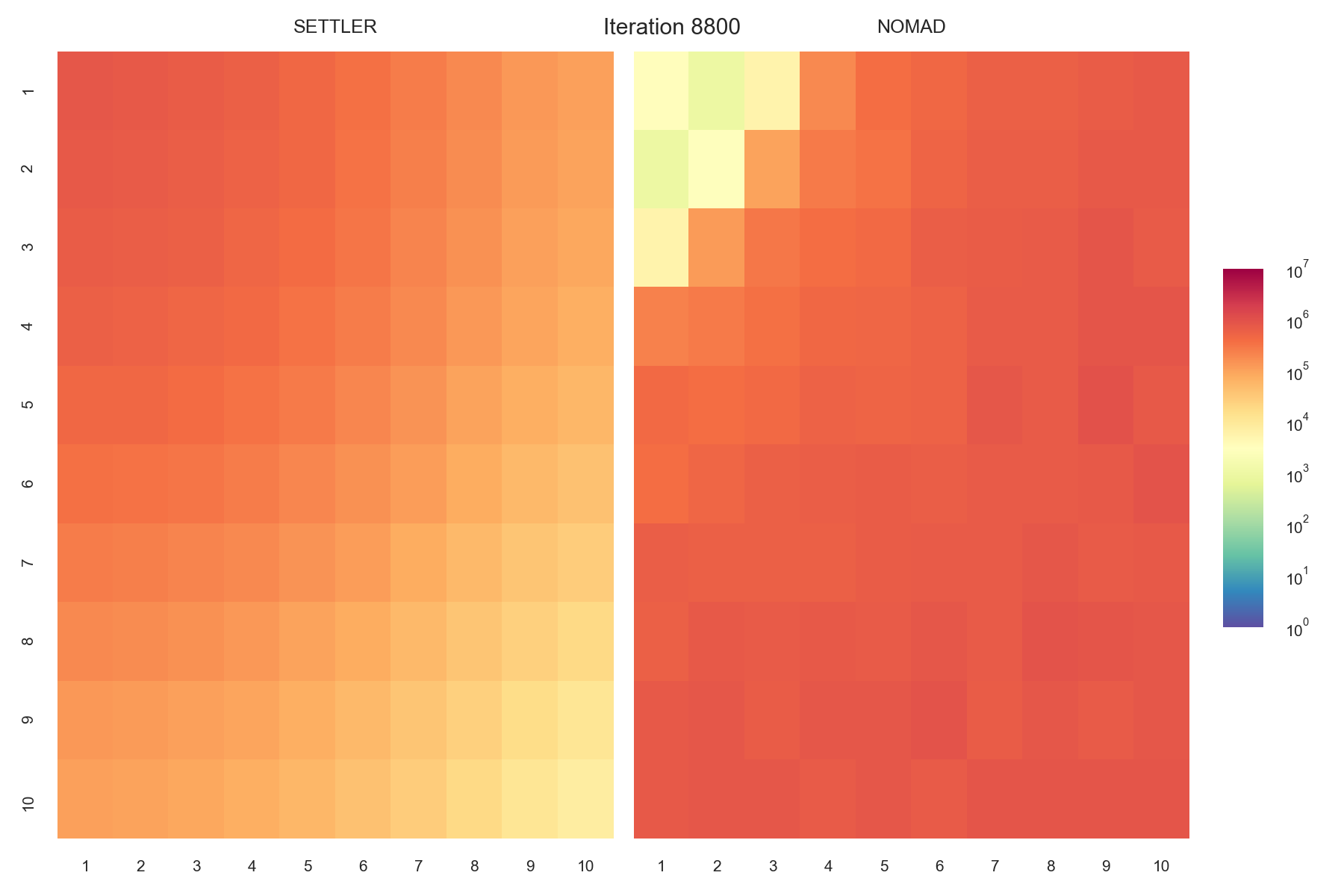

Supplement: Supplementary file 1 [file biology-10-01019-s001.zip › Spatio-temporal dynamics heatmaps/chempenoff_extremelyscarce_lindeath_period1000/8800.png]

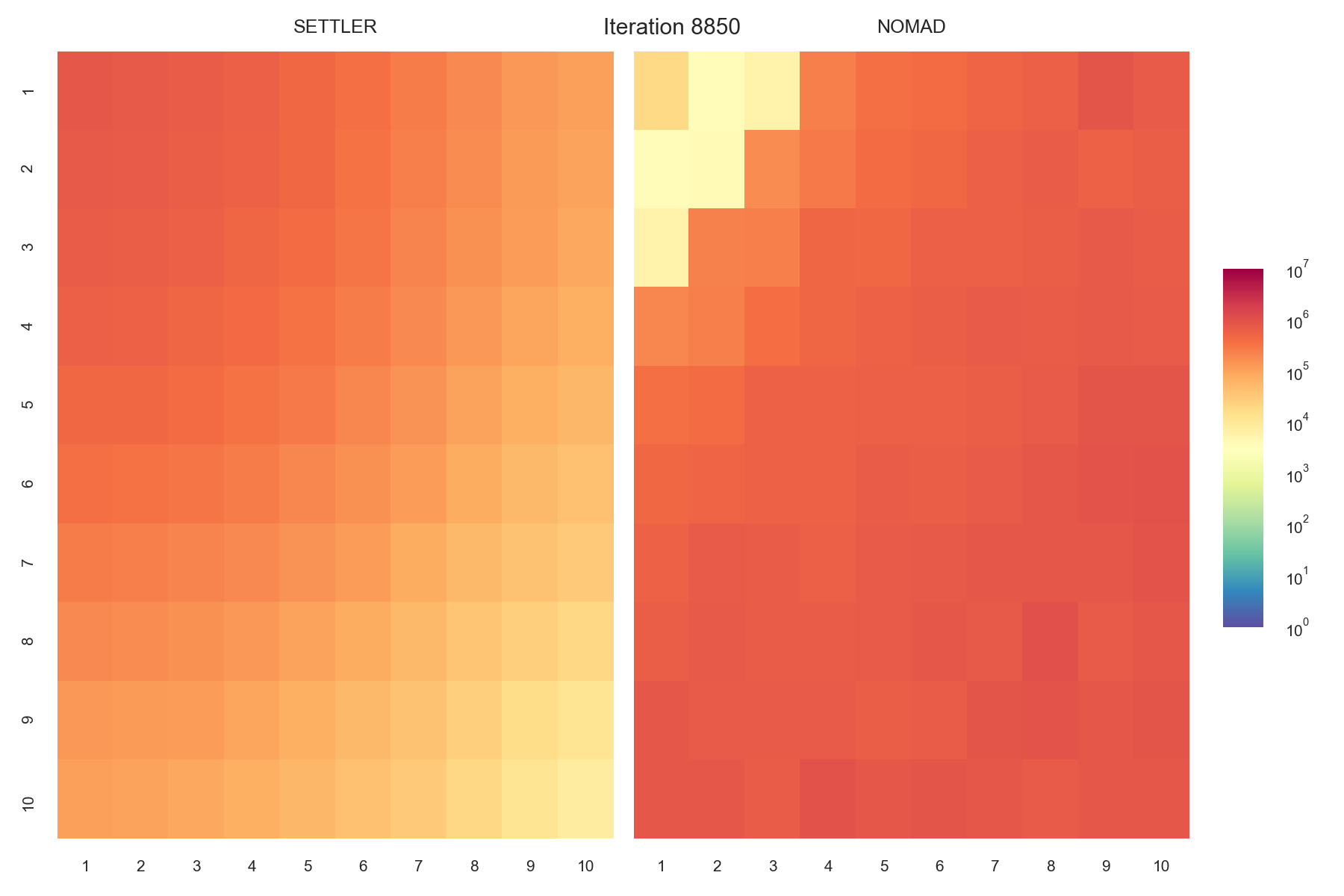

Supplement: Supplementary file 1 [file biology-10-01019-s001.zip › Spatio-temporal dynamics heatmaps/chempenoff_extremelyscarce_lindeath_period1000/8850.png]

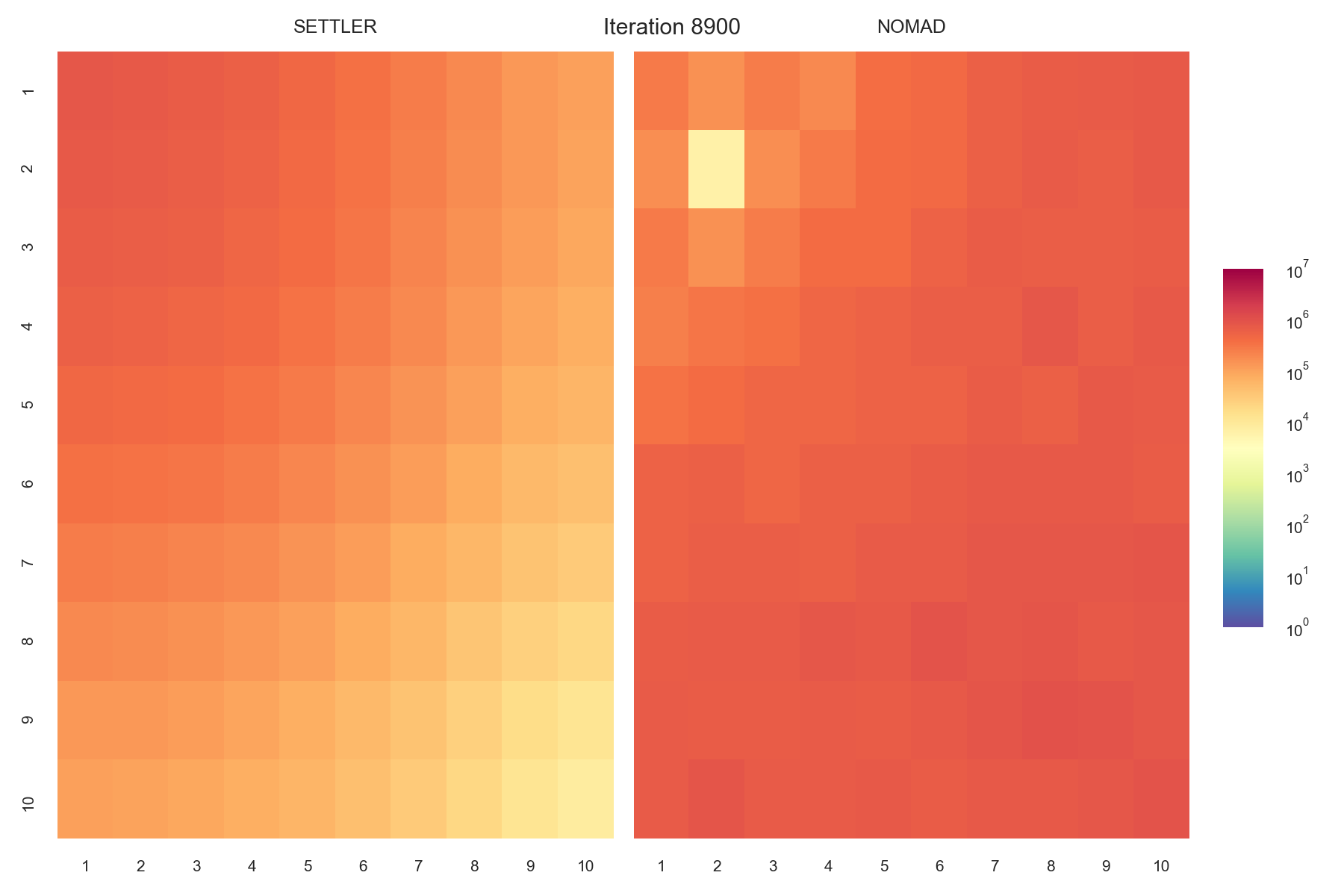

Supplement: Supplementary file 1 [file biology-10-01019-s001.zip › Spatio-temporal dynamics heatmaps/chempenoff_extremelyscarce_lindeath_period1000/8900.png]

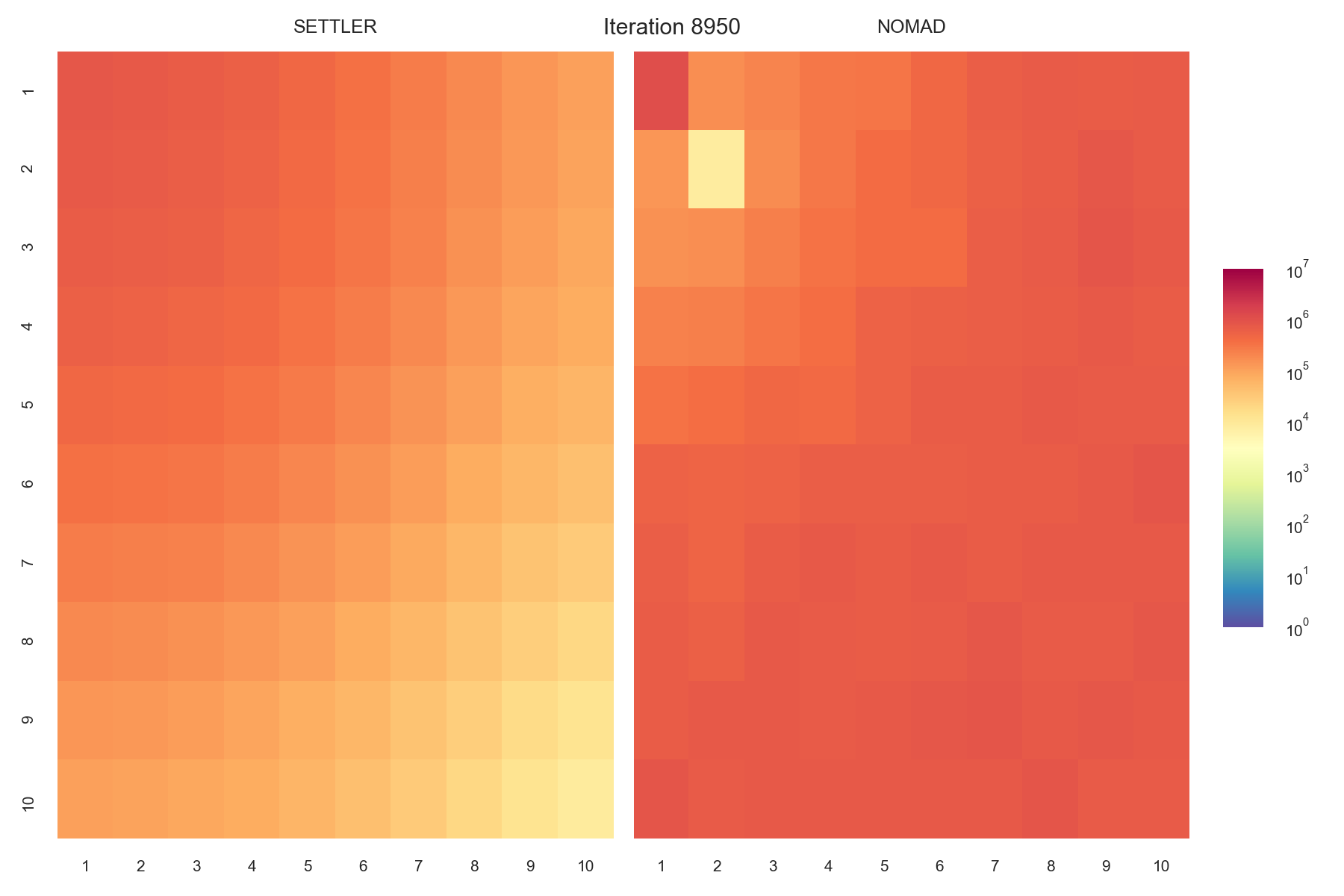

Supplement: Supplementary file 1 [file biology-10-01019-s001.zip › Spatio-temporal dynamics heatmaps/chempenoff_extremelyscarce_lindeath_period1000/8950.png]

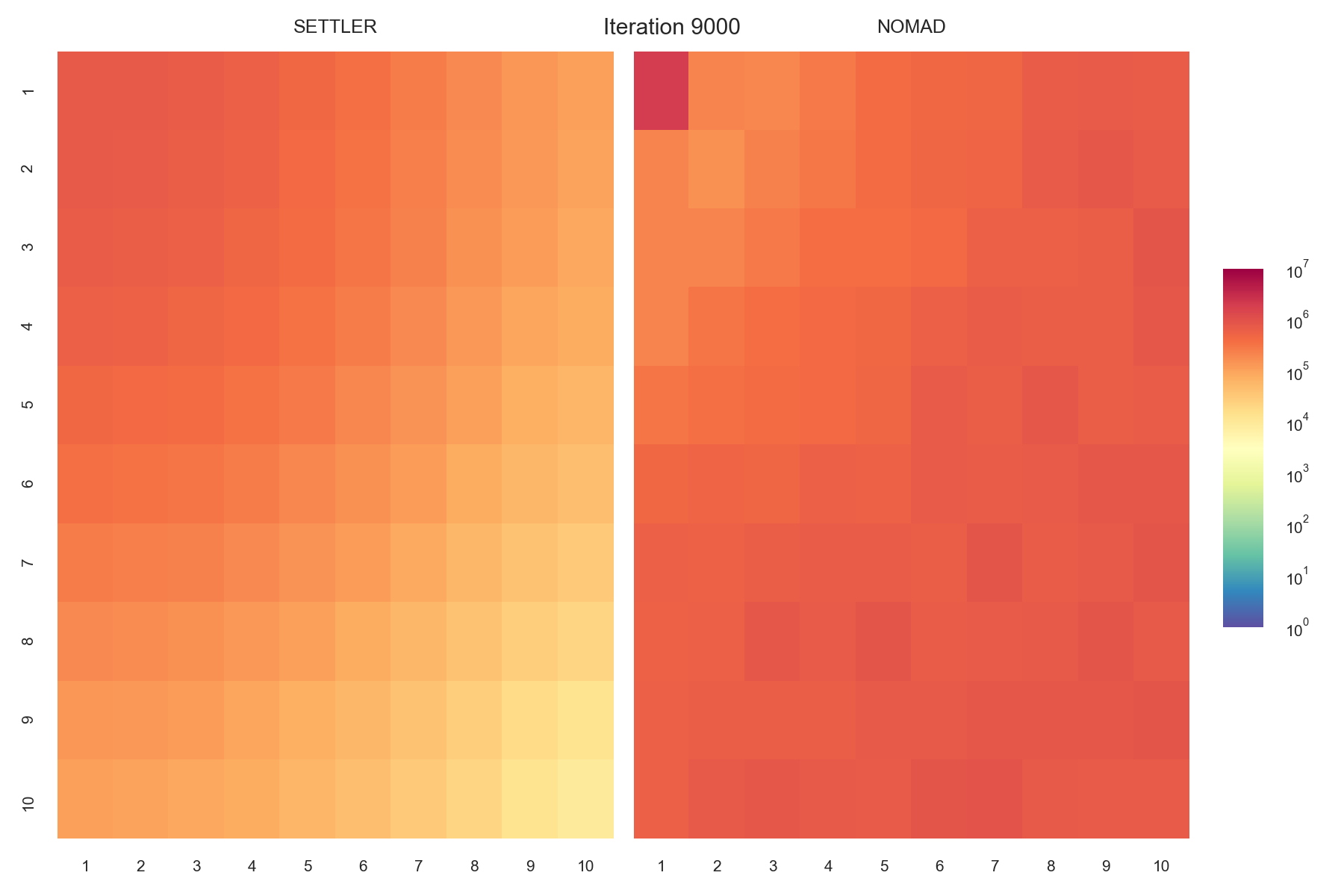

Supplement: Supplementary file 1 [file biology-10-01019-s001.zip › Spatio-temporal dynamics heatmaps/chempenoff_extremelyscarce_lindeath_period1000/9000.png]

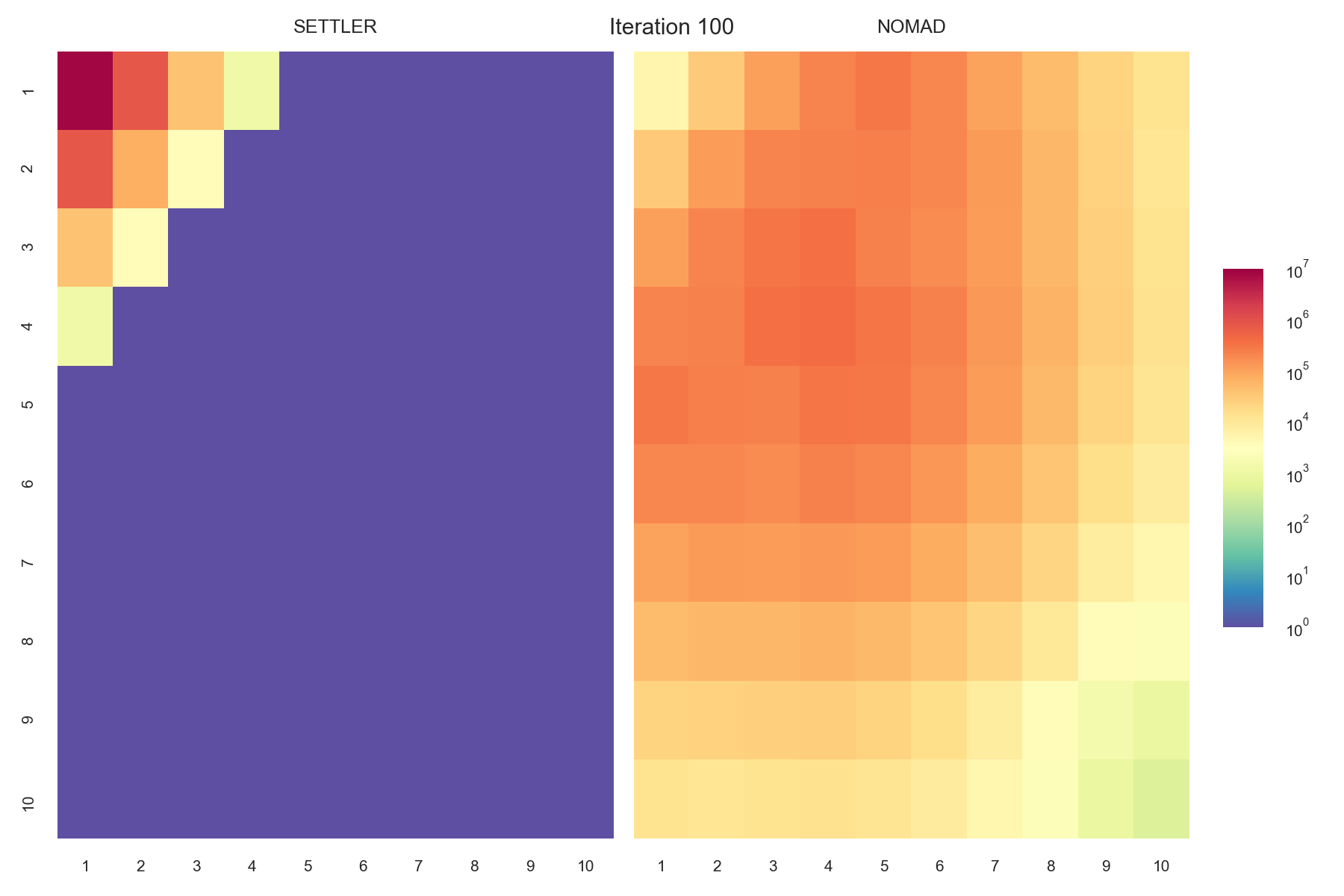

Supplement: Supplementary file 1 [file biology-10-01019-s001.zip › Spatio-temporal dynamics heatmaps/chempenoff_extremelyscarce_lindeath_period50/0100.png]

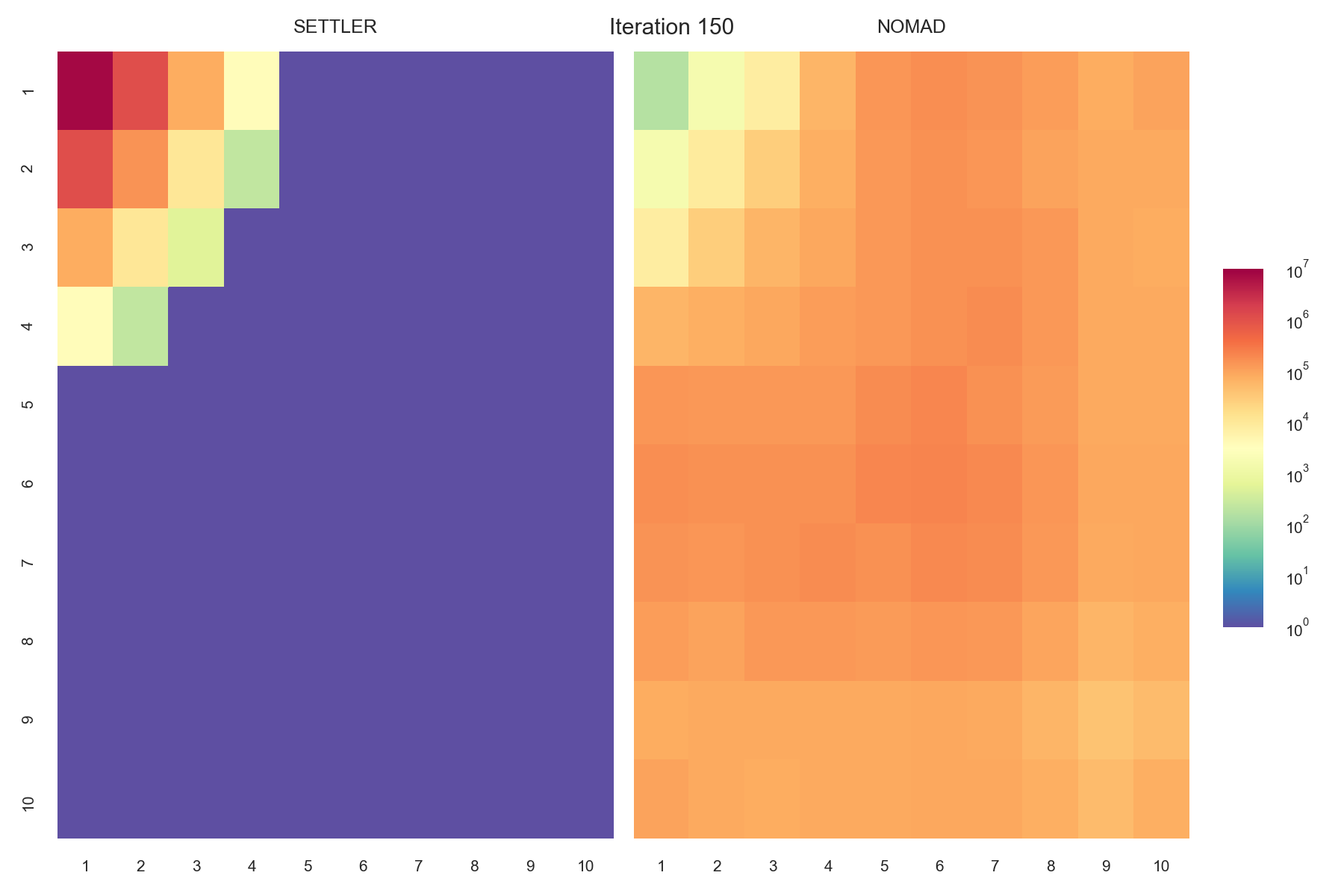

Supplement: Supplementary file 1 [file biology-10-01019-s001.zip › Spatio-temporal dynamics heatmaps/chempenoff_extremelyscarce_lindeath_period50/0150.png]

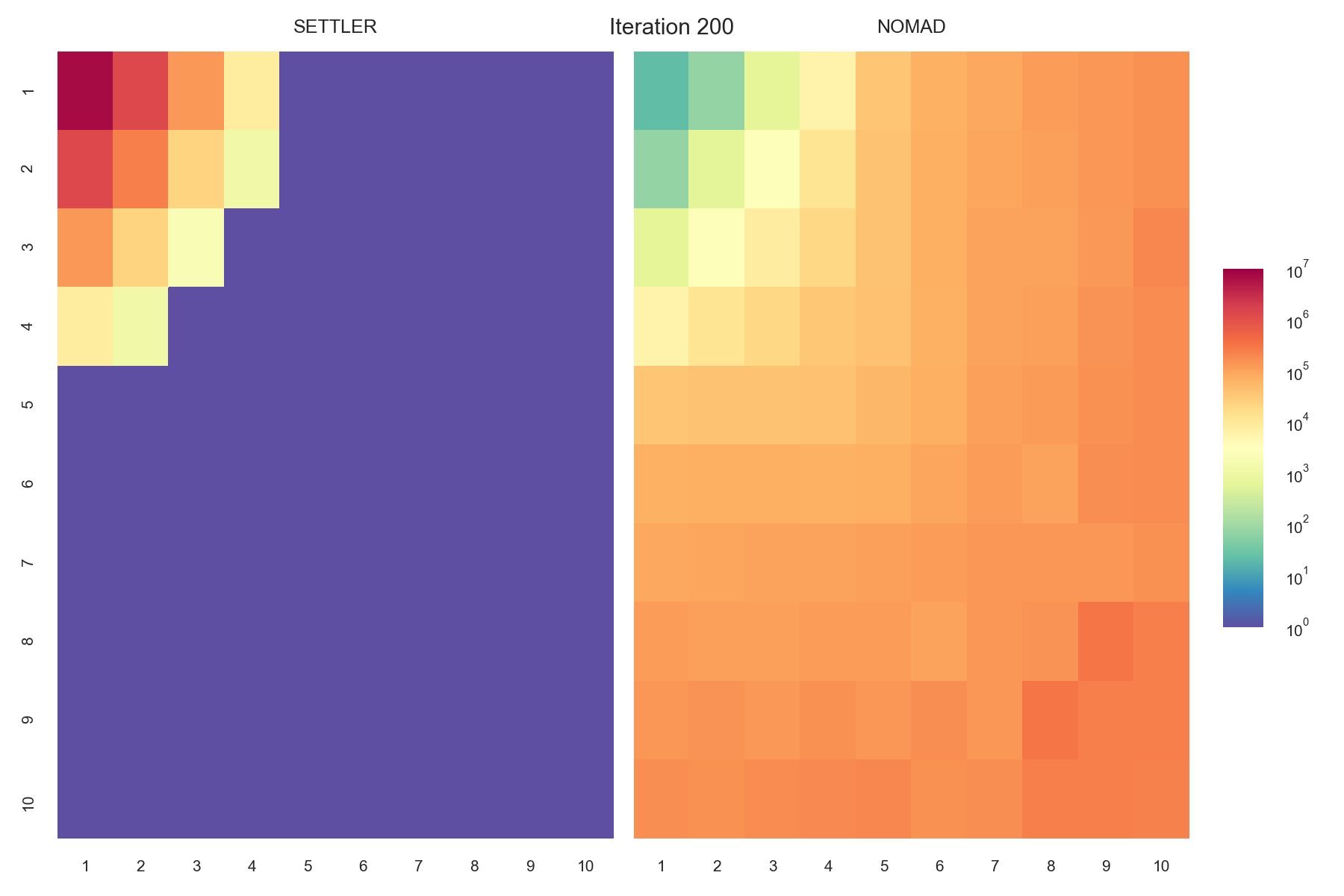

Supplement: Supplementary file 1 [file biology-10-01019-s001.zip › Spatio-temporal dynamics heatmaps/chempenoff_extremelyscarce_lindeath_period50/0200.png]

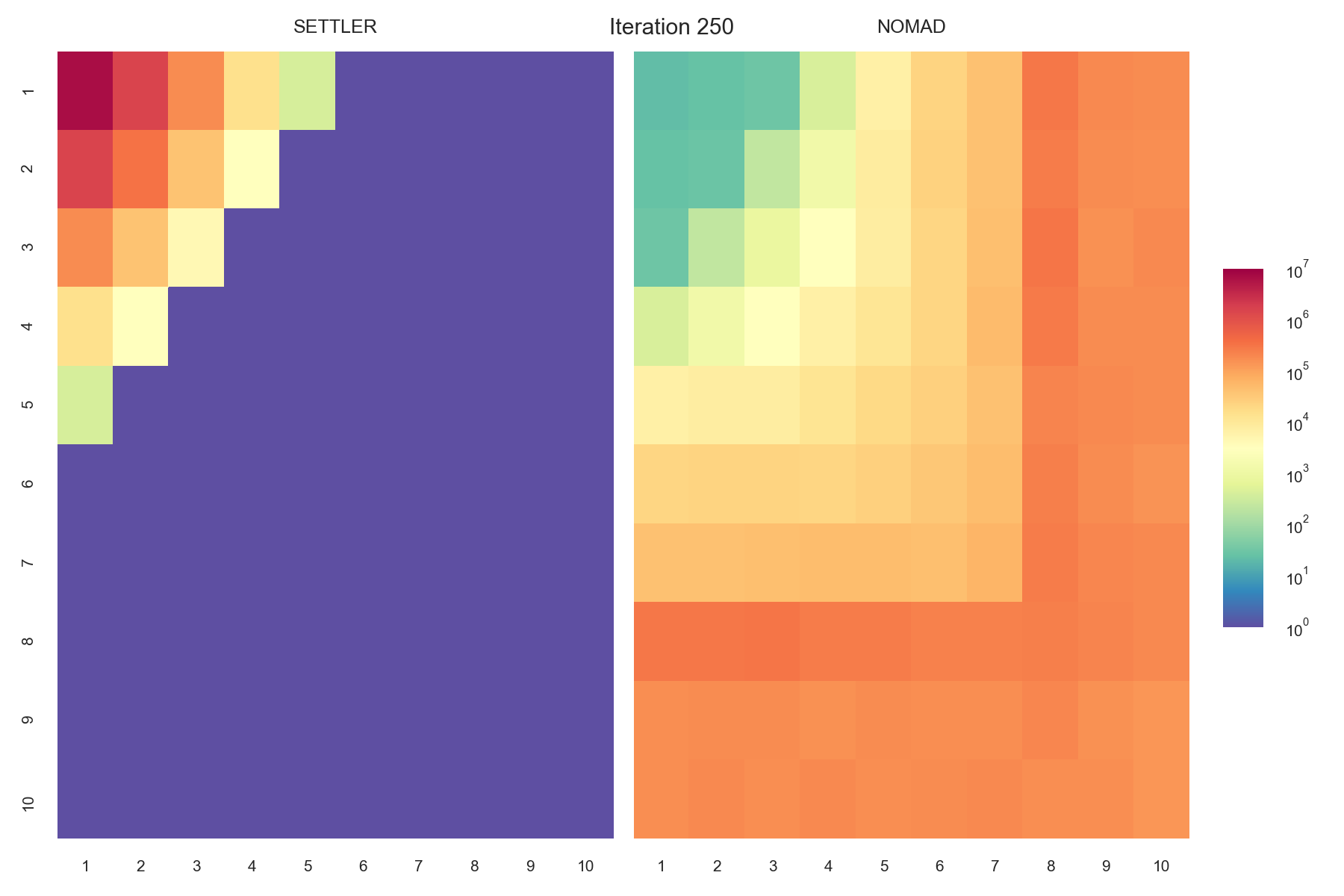

Supplement: Supplementary file 1 [file biology-10-01019-s001.zip › Spatio-temporal dynamics heatmaps/chempenoff_extremelyscarce_lindeath_period50/0250.png]

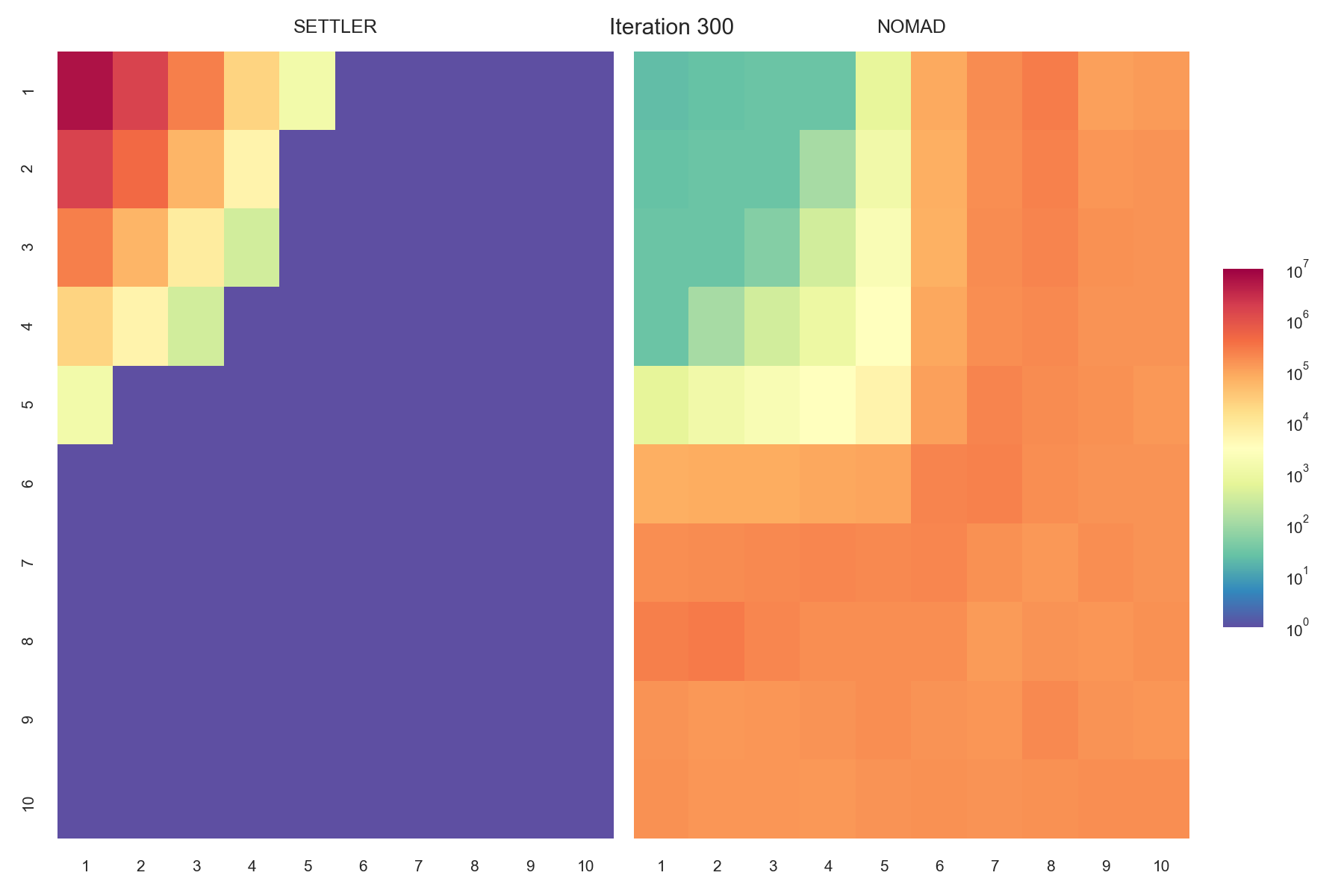

Supplement: Supplementary file 1 [file biology-10-01019-s001.zip › Spatio-temporal dynamics heatmaps/chempenoff_extremelyscarce_lindeath_period50/0300.png]

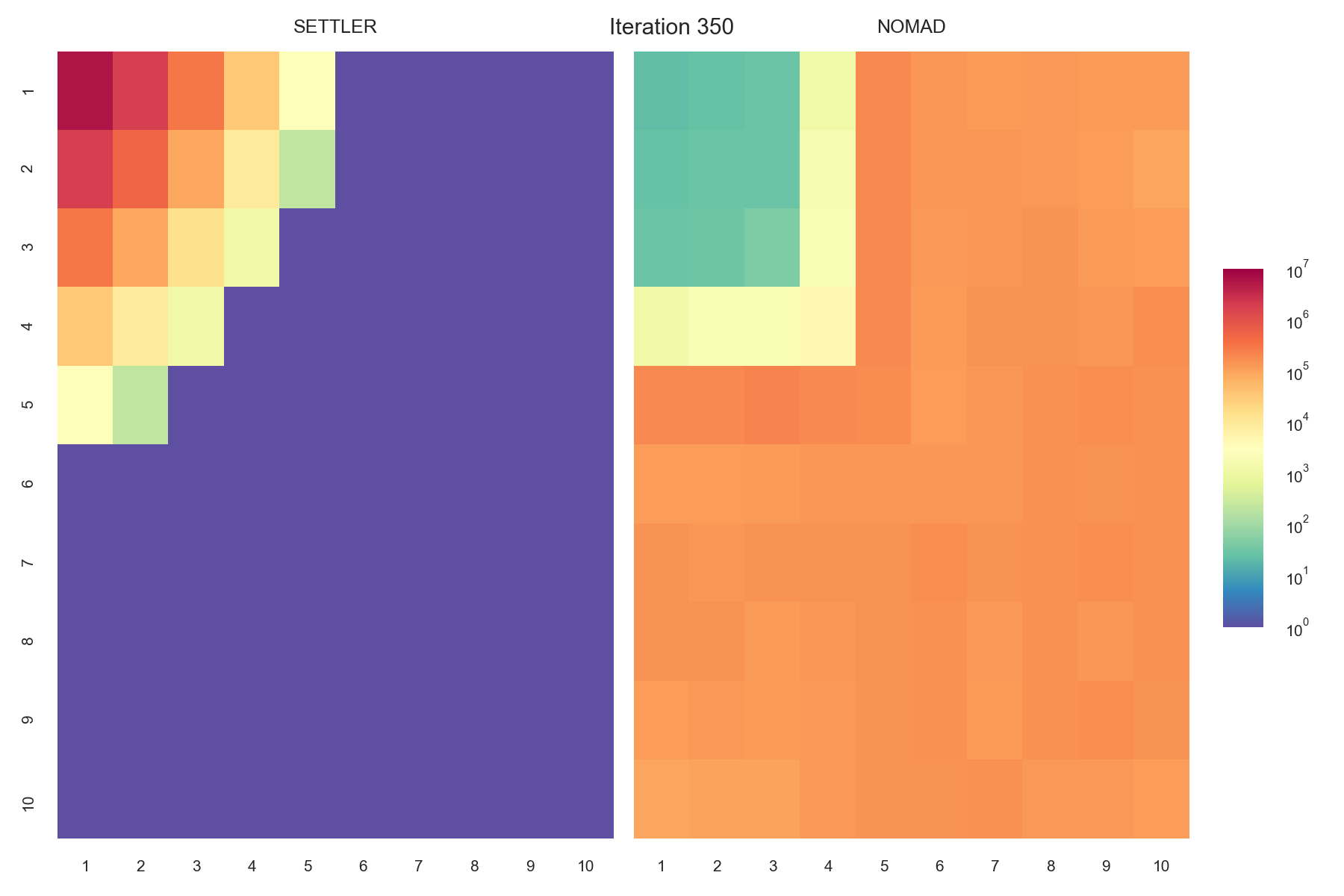

Supplement: Supplementary file 1 [file biology-10-01019-s001.zip › Spatio-temporal dynamics heatmaps/chempenoff_extremelyscarce_lindeath_period50/0350.png]

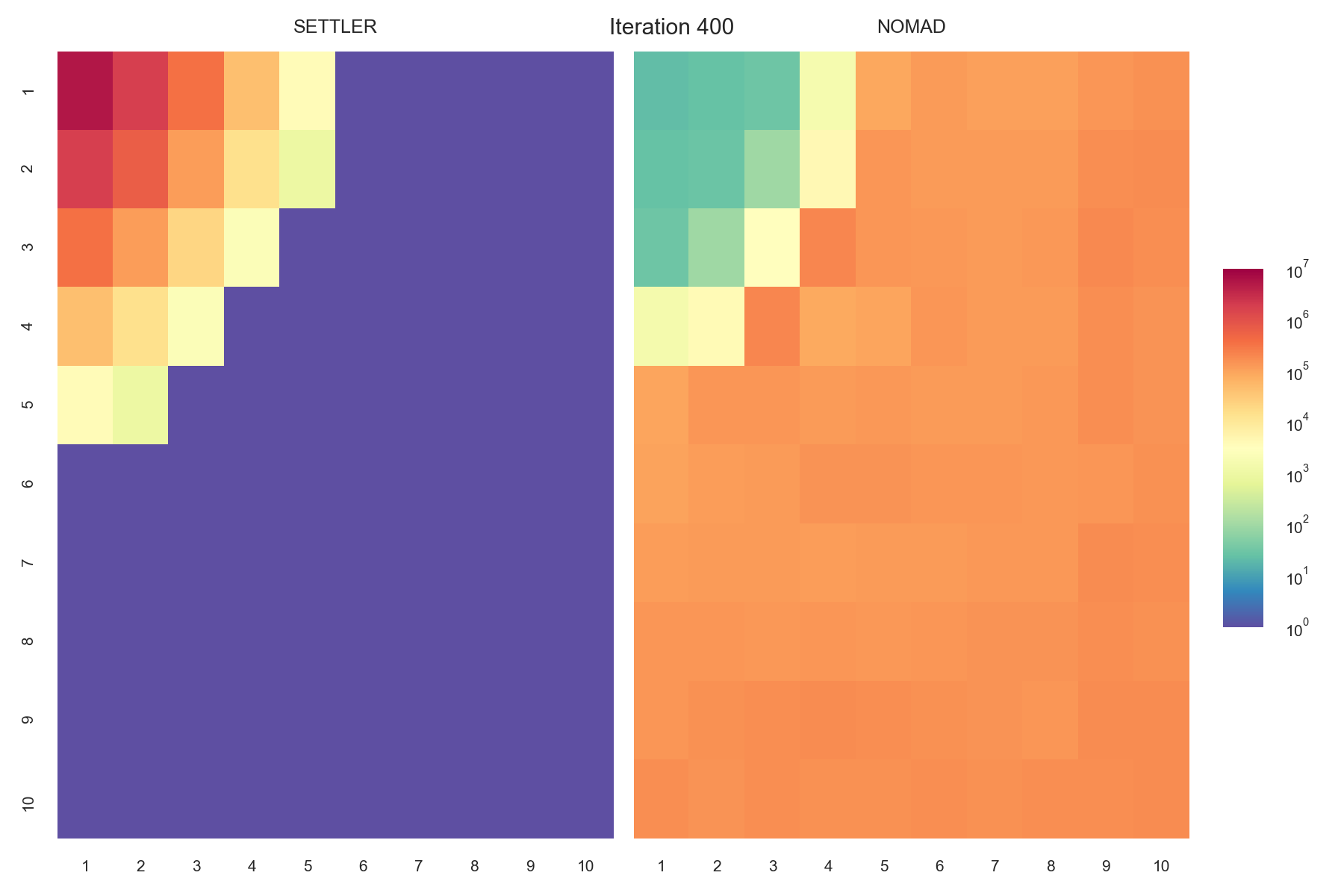

Supplement: Supplementary file 1 [file biology-10-01019-s001.zip › Spatio-temporal dynamics heatmaps/chempenoff_extremelyscarce_lindeath_period50/0400.png]

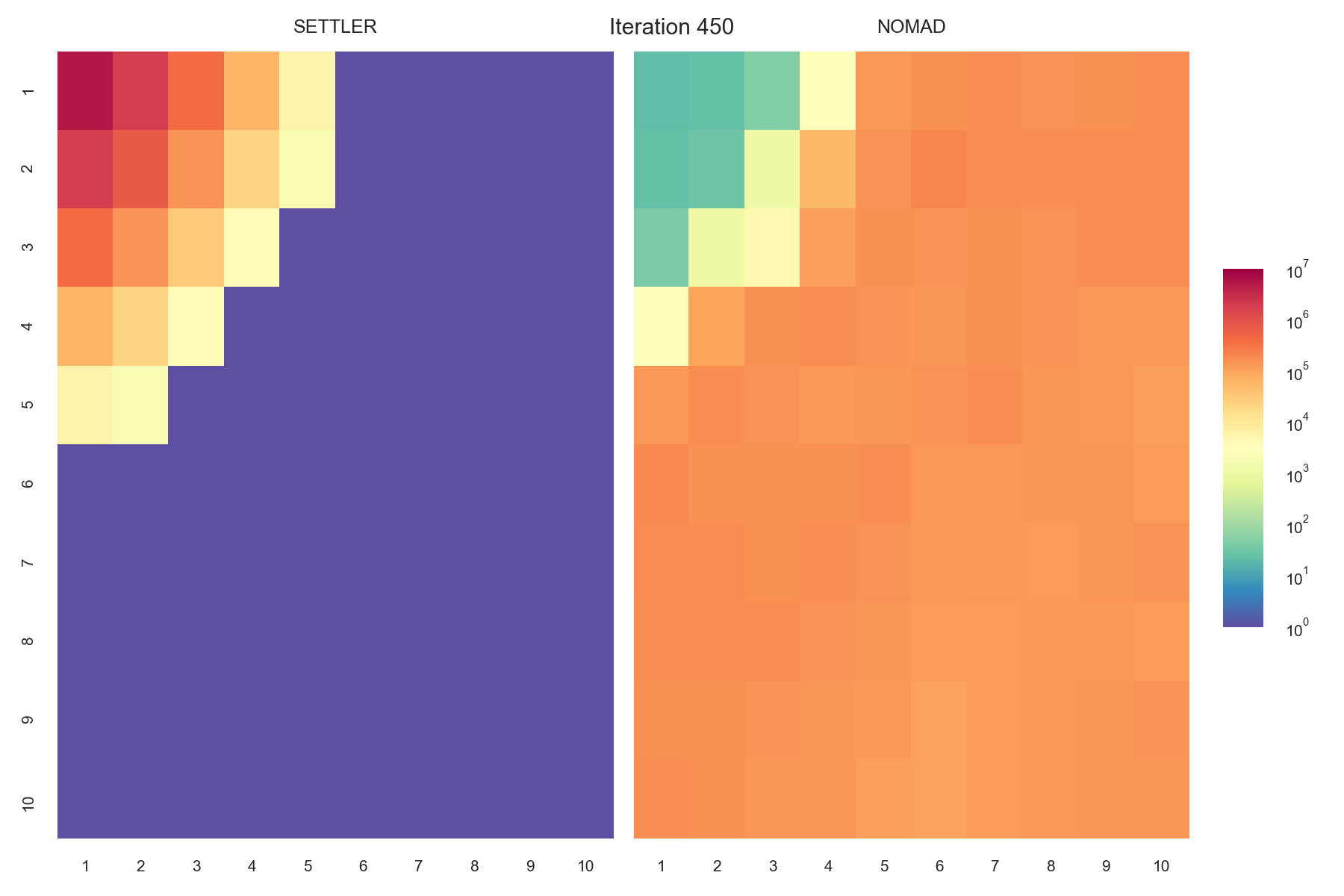

Supplement: Supplementary file 1 [file biology-10-01019-s001.zip › Spatio-temporal dynamics heatmaps/chempenoff_extremelyscarce_lindeath_period50/0450.png]

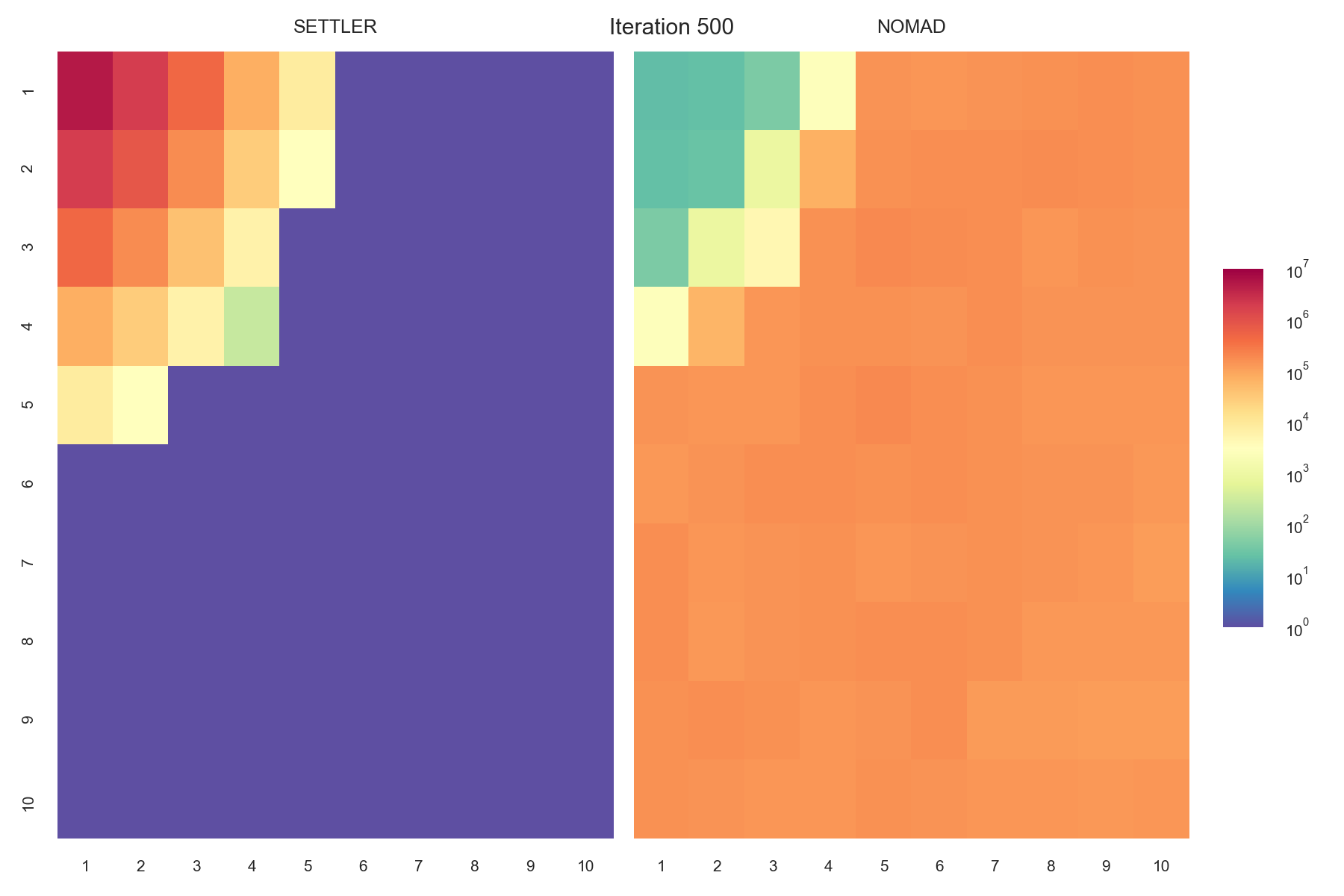

Supplement: Supplementary file 1 [file biology-10-01019-s001.zip › Spatio-temporal dynamics heatmaps/chempenoff_extremelyscarce_lindeath_period50/0500.png]

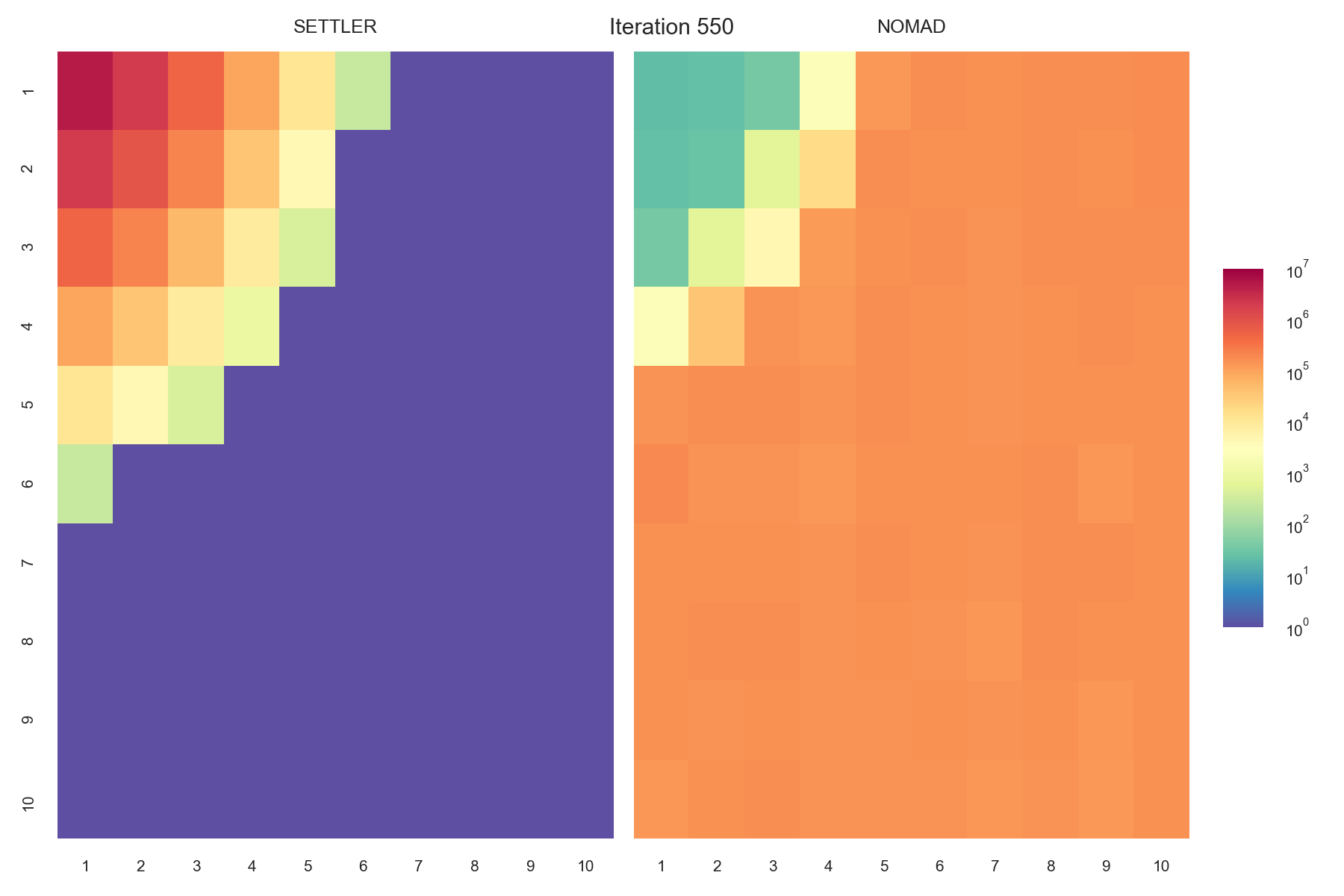

Supplement: Supplementary file 1 [file biology-10-01019-s001.zip › Spatio-temporal dynamics heatmaps/chempenoff_extremelyscarce_lindeath_period50/0550.png]

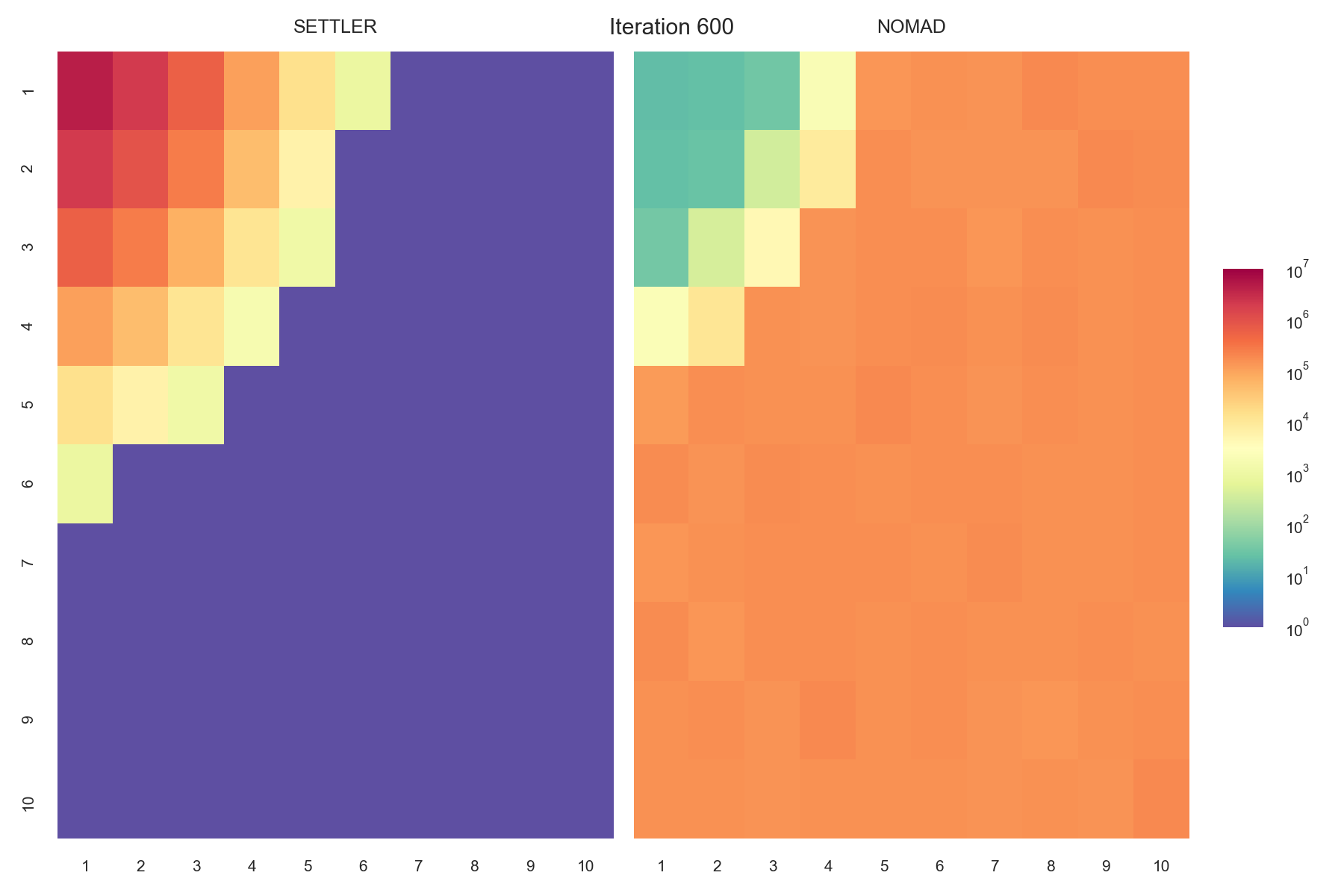

Supplement: Supplementary file 1 [file biology-10-01019-s001.zip › Spatio-temporal dynamics heatmaps/chempenoff_extremelyscarce_lindeath_period50/0600.png]
