# Supplementary figures and images for: Leave or Stay: Simulating Motility and Fitness of Microorganisms in Dynamic Aquatic Ecosystems (part 1 of 2)
Source: Biology (Basel). 2021 Oct 9;10(10):1019. doi: 10.3390/biology10101019 (PMC8533222; doi:10.3390/biology10101019)

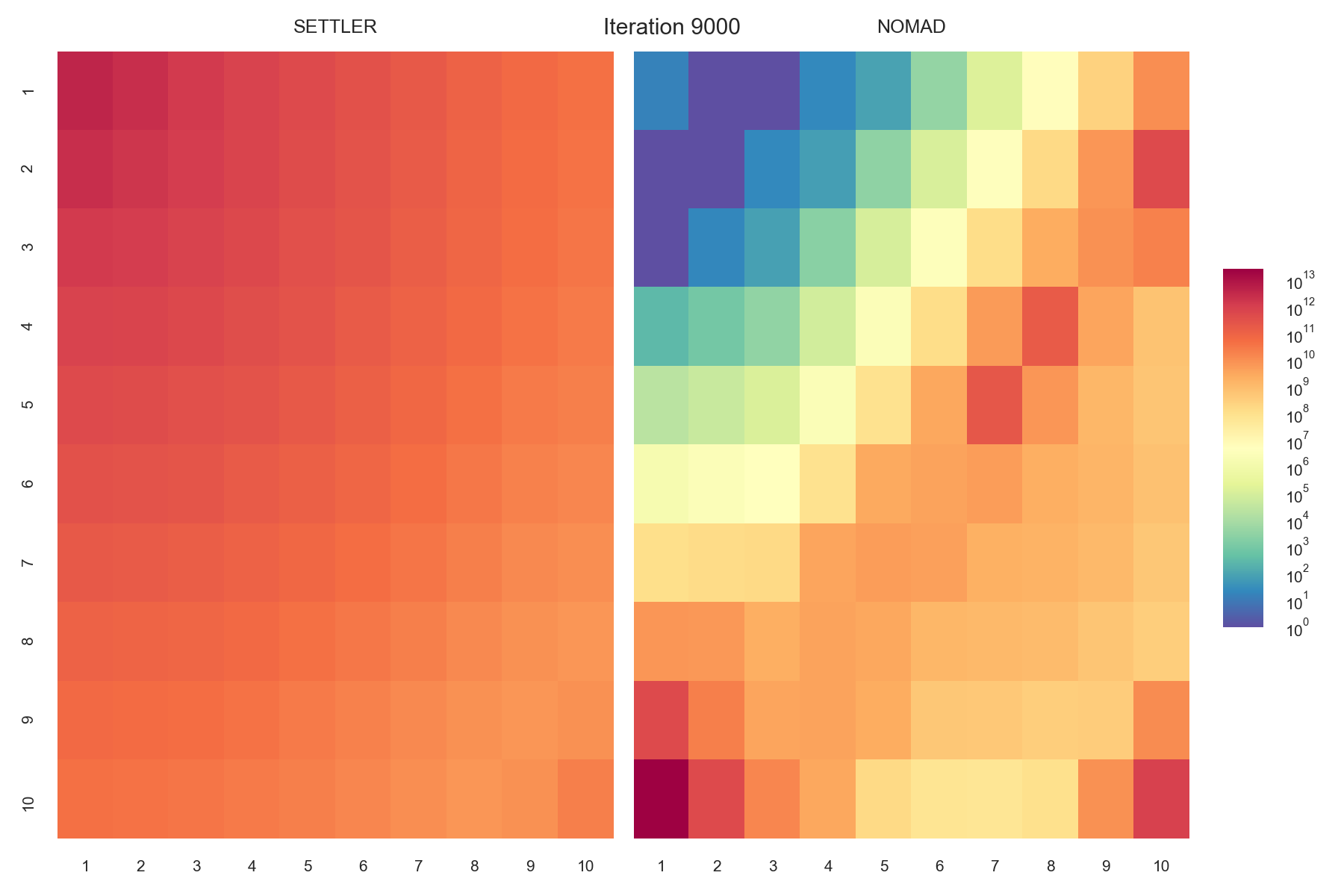

Supplement: Supplementary file 1 [file biology-10-01019-s001.zip › Figure S1.png]

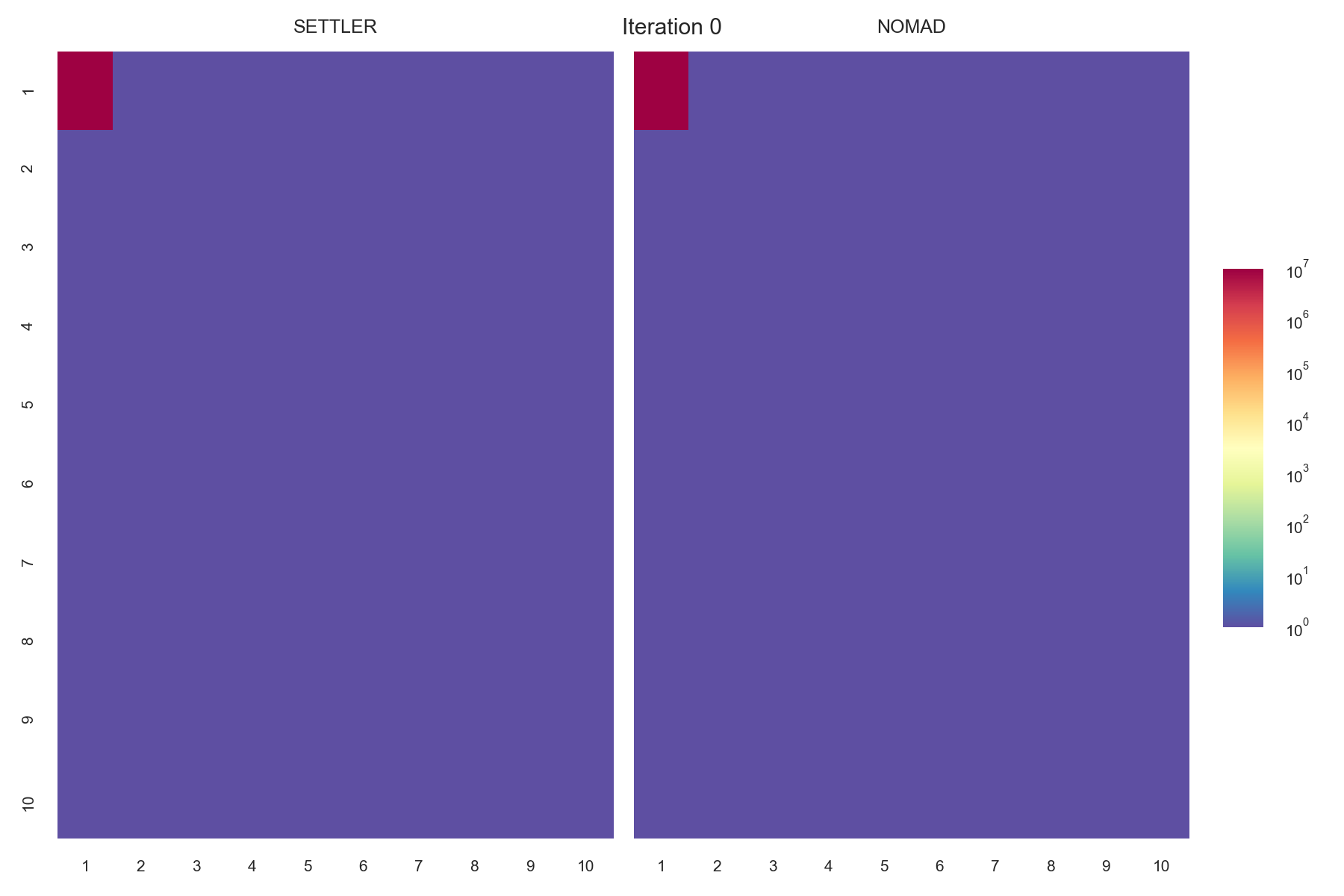

Supplement: Supplementary file 1 [file biology-10-01019-s001.zip › Spatio-temporal dynamics heatmaps/chempenoff_extremelyscarce_lindeath_period1000/0000.png]

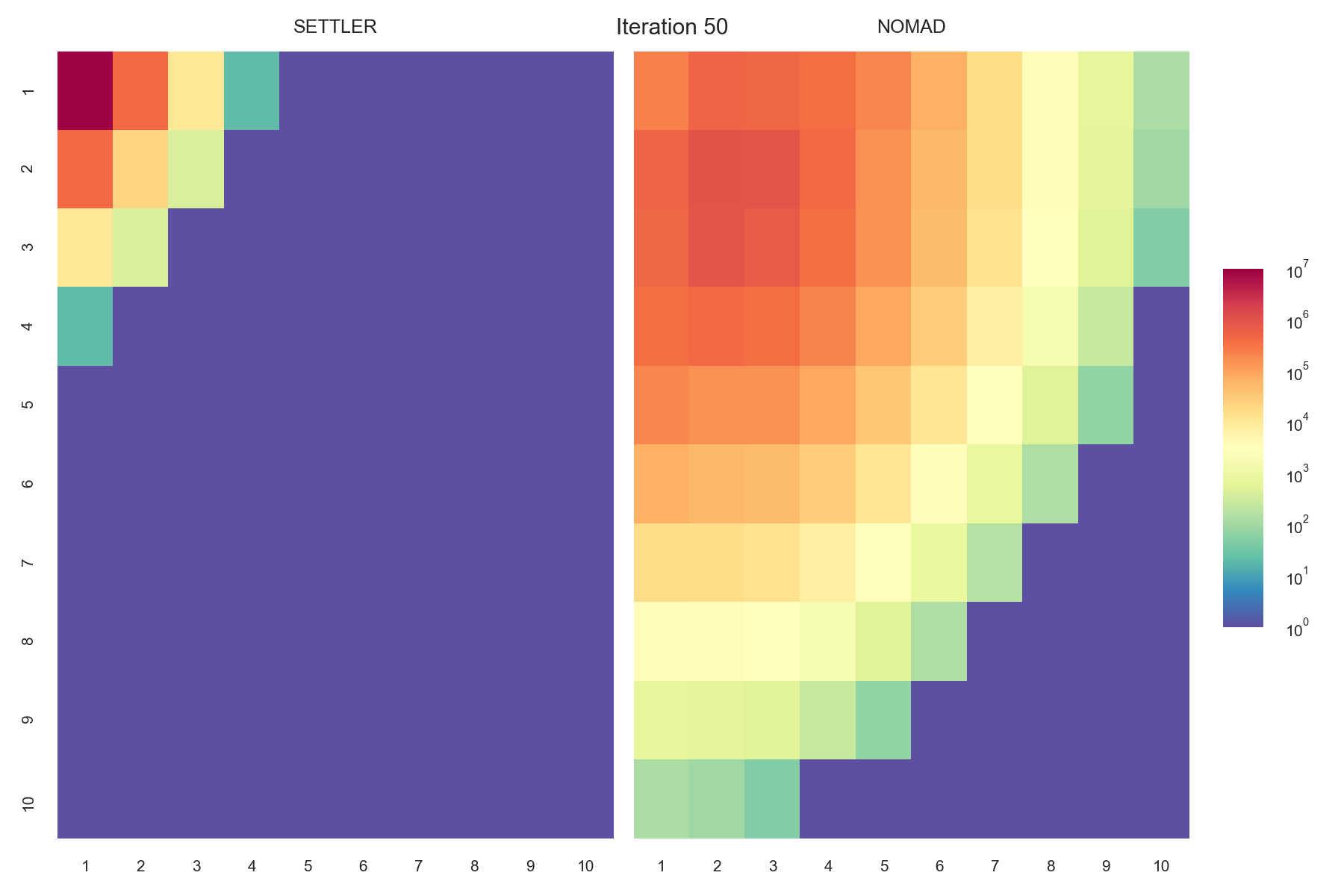

Supplement: Supplementary file 1 [file biology-10-01019-s001.zip › Spatio-temporal dynamics heatmaps/chempenoff_extremelyscarce_lindeath_period1000/0050.png]

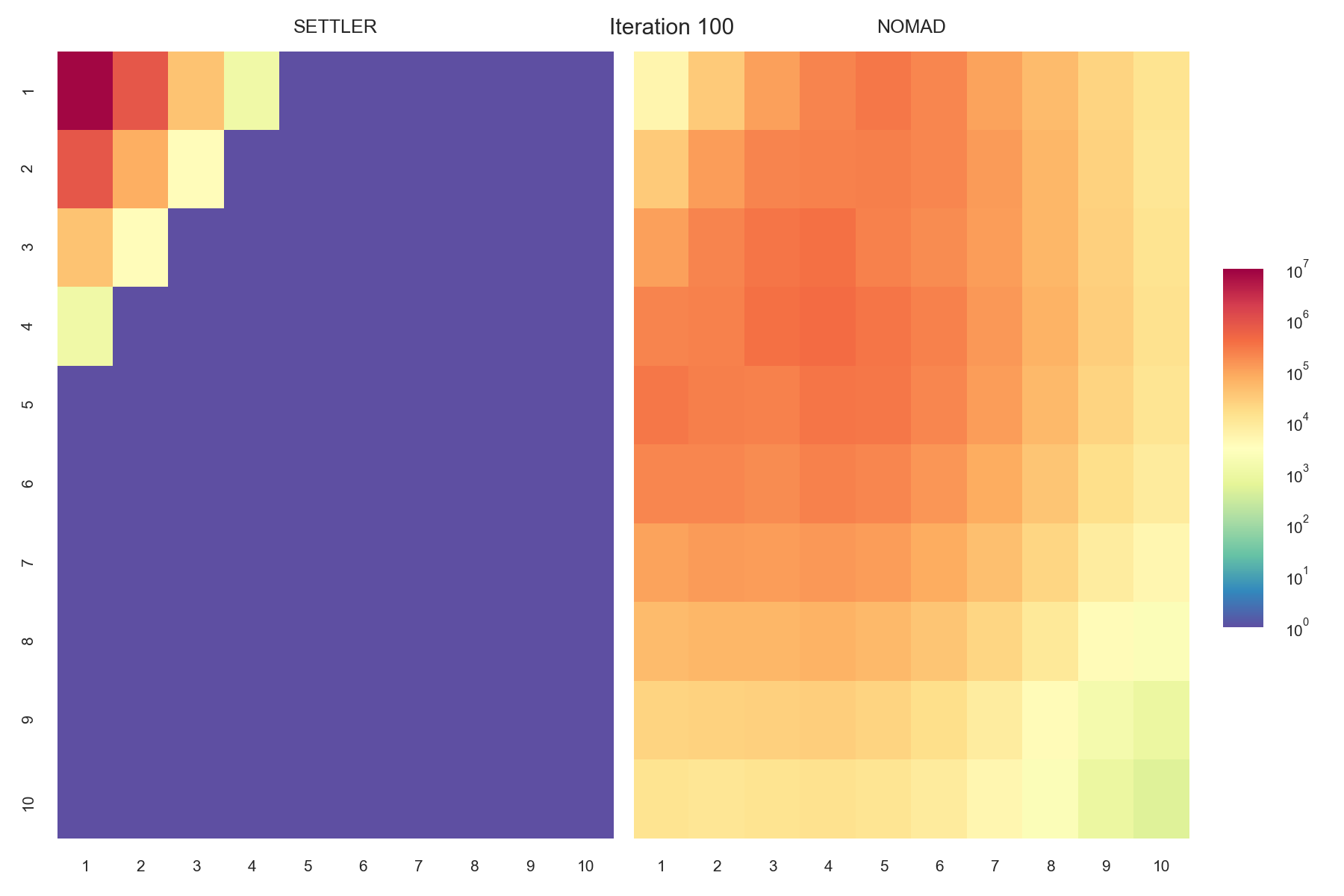

Supplement: Supplementary file 1 [file biology-10-01019-s001.zip › Spatio-temporal dynamics heatmaps/chempenoff_extremelyscarce_lindeath_period1000/0100.png]

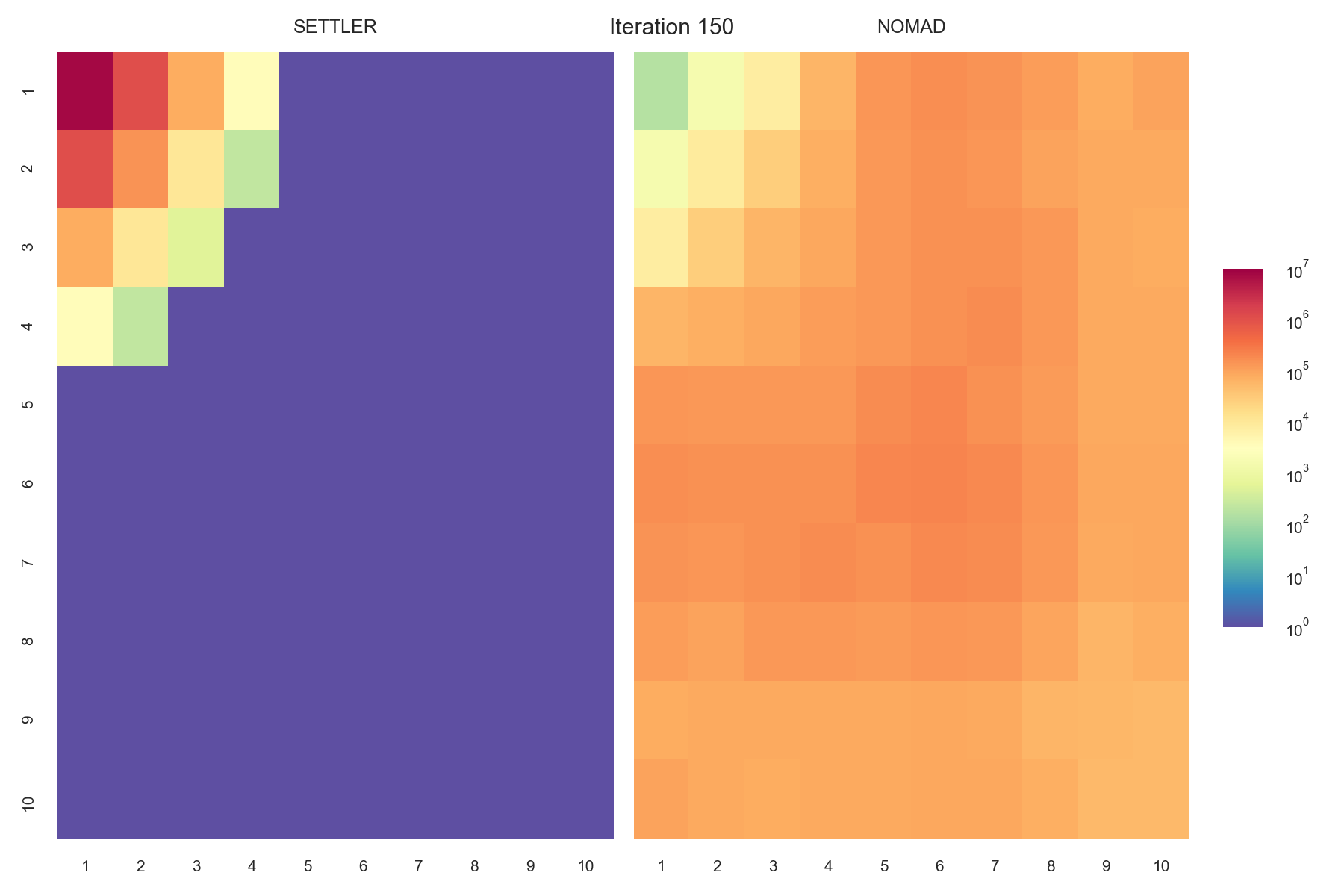

Supplement: Supplementary file 1 [file biology-10-01019-s001.zip › Spatio-temporal dynamics heatmaps/chempenoff_extremelyscarce_lindeath_period1000/0150.png]

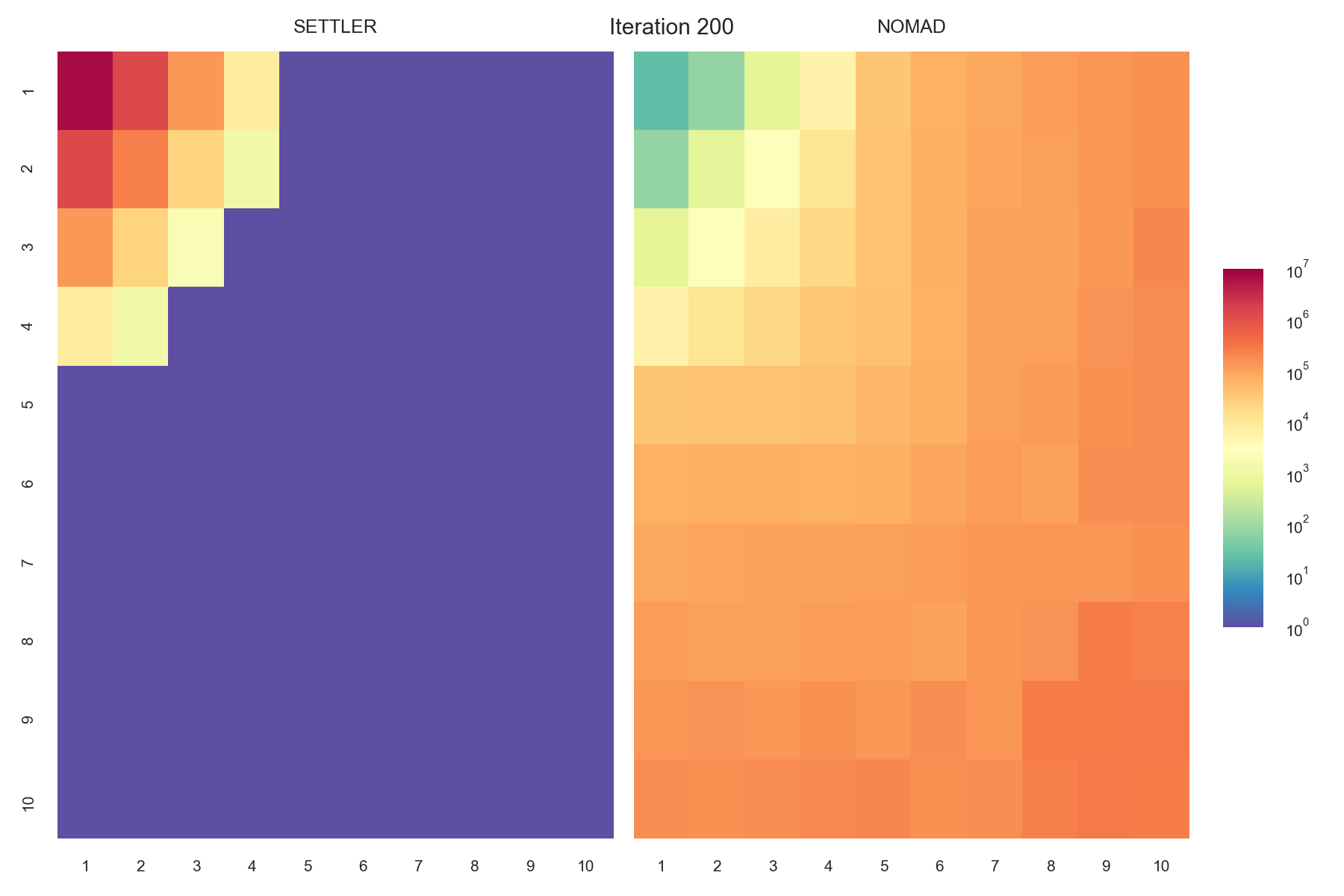

Supplement: Supplementary file 1 [file biology-10-01019-s001.zip › Spatio-temporal dynamics heatmaps/chempenoff_extremelyscarce_lindeath_period1000/0200.png]

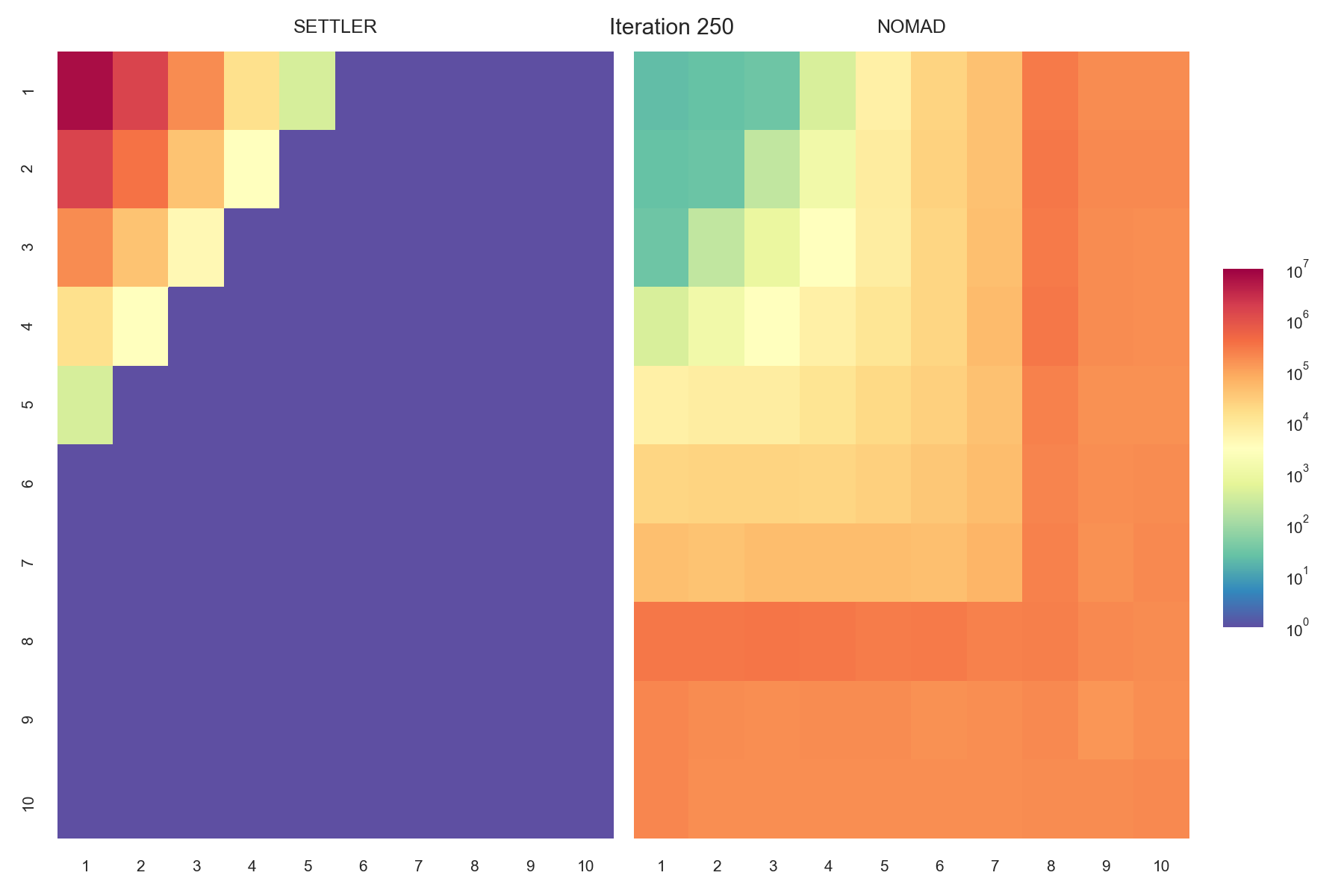

Supplement: Supplementary file 1 [file biology-10-01019-s001.zip › Spatio-temporal dynamics heatmaps/chempenoff_extremelyscarce_lindeath_period1000/0250.png]

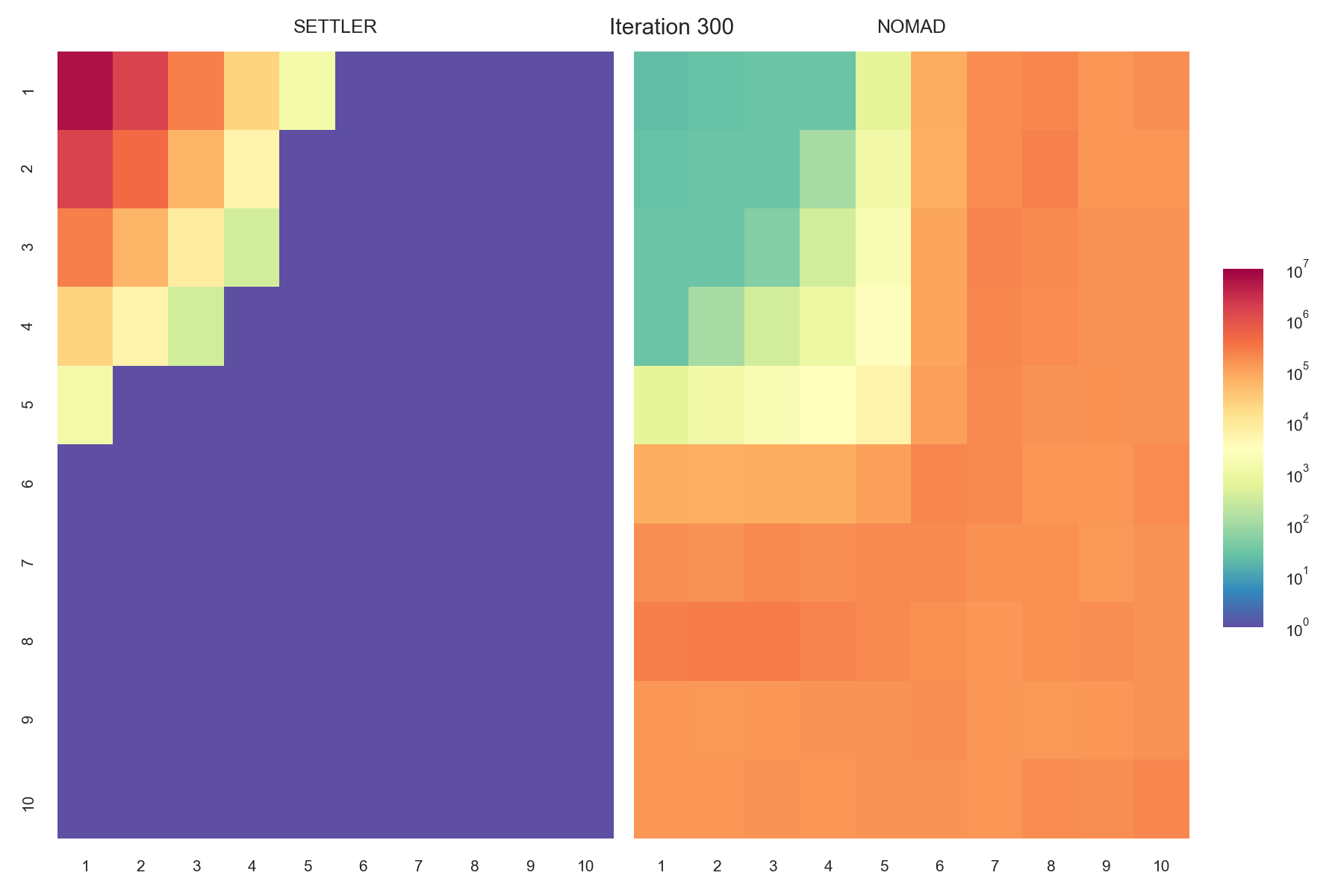

Supplement: Supplementary file 1 [file biology-10-01019-s001.zip › Spatio-temporal dynamics heatmaps/chempenoff_extremelyscarce_lindeath_period1000/0300.png]

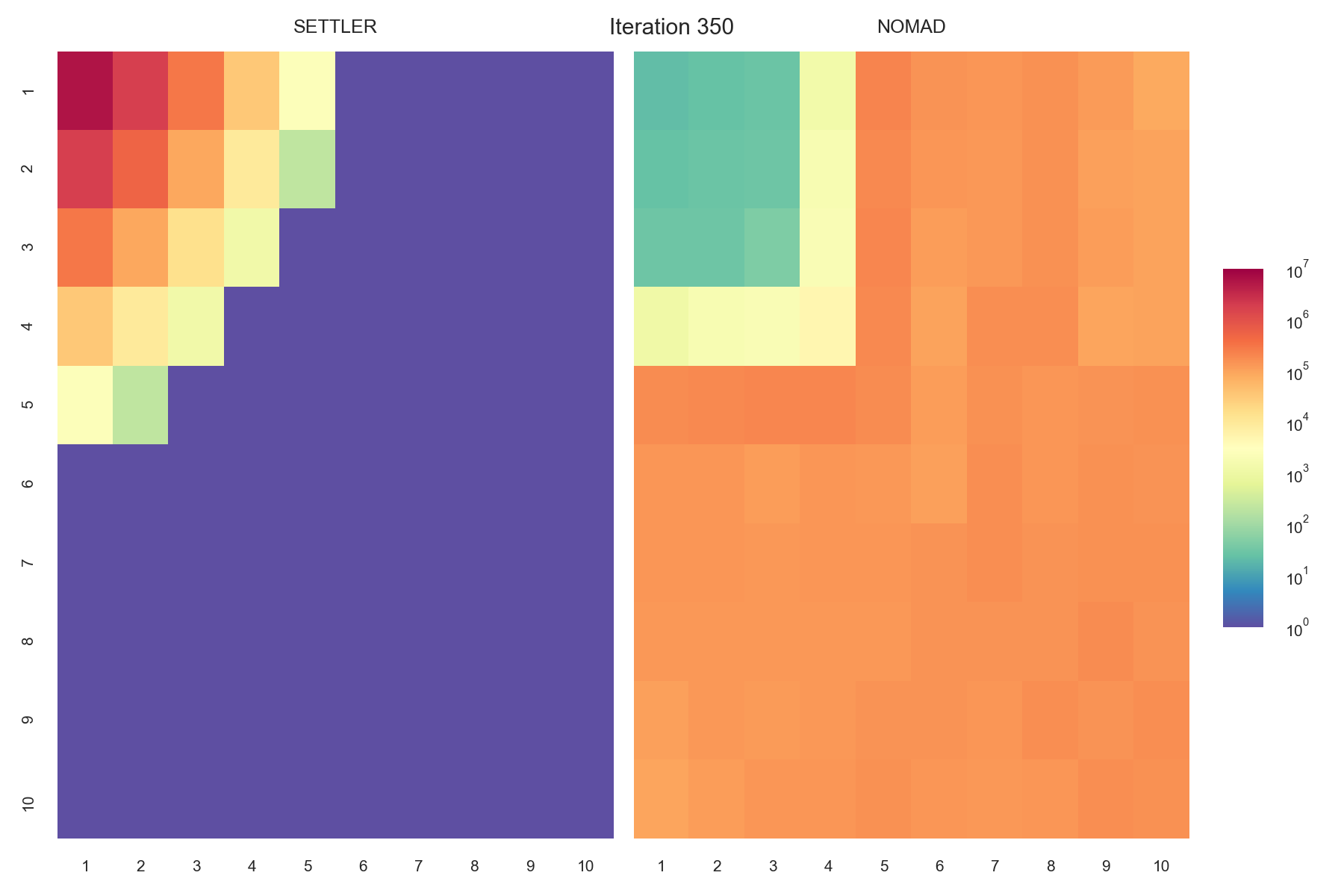

Supplement: Supplementary file 1 [file biology-10-01019-s001.zip › Spatio-temporal dynamics heatmaps/chempenoff_extremelyscarce_lindeath_period1000/0350.png]

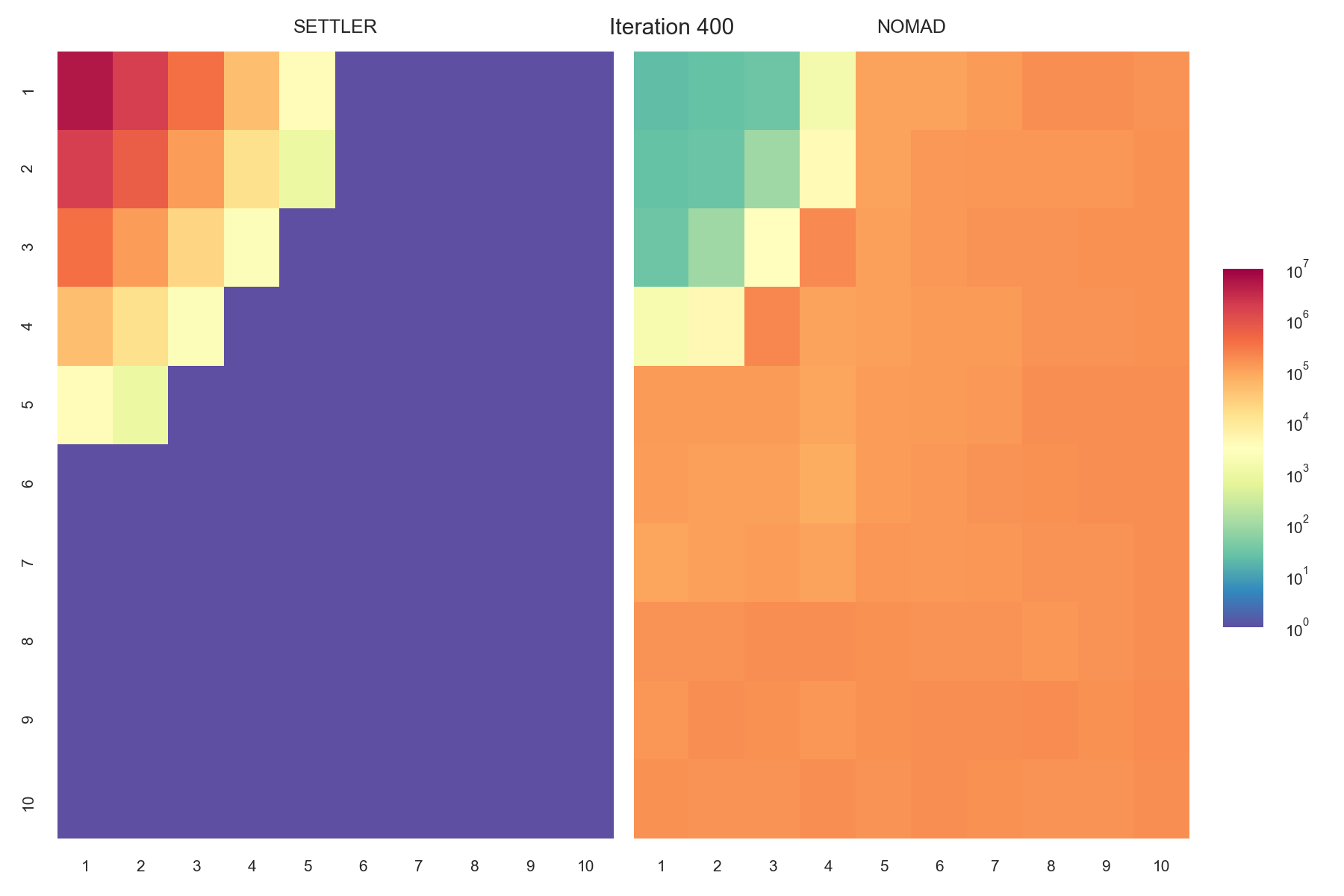

Supplement: Supplementary file 1 [file biology-10-01019-s001.zip › Spatio-temporal dynamics heatmaps/chempenoff_extremelyscarce_lindeath_period1000/0400.png]

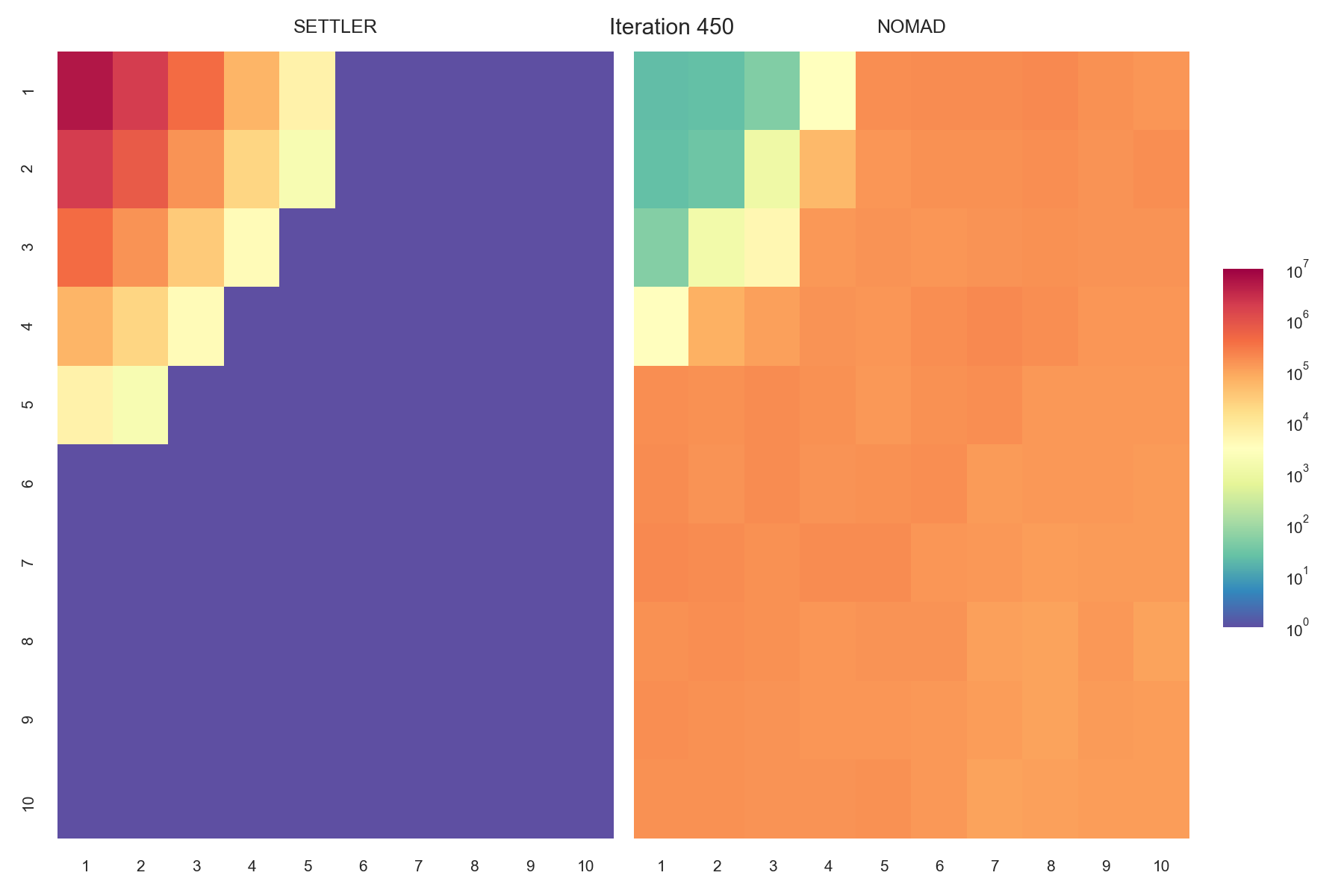

Supplement: Supplementary file 1 [file biology-10-01019-s001.zip › Spatio-temporal dynamics heatmaps/chempenoff_extremelyscarce_lindeath_period1000/0450.png]

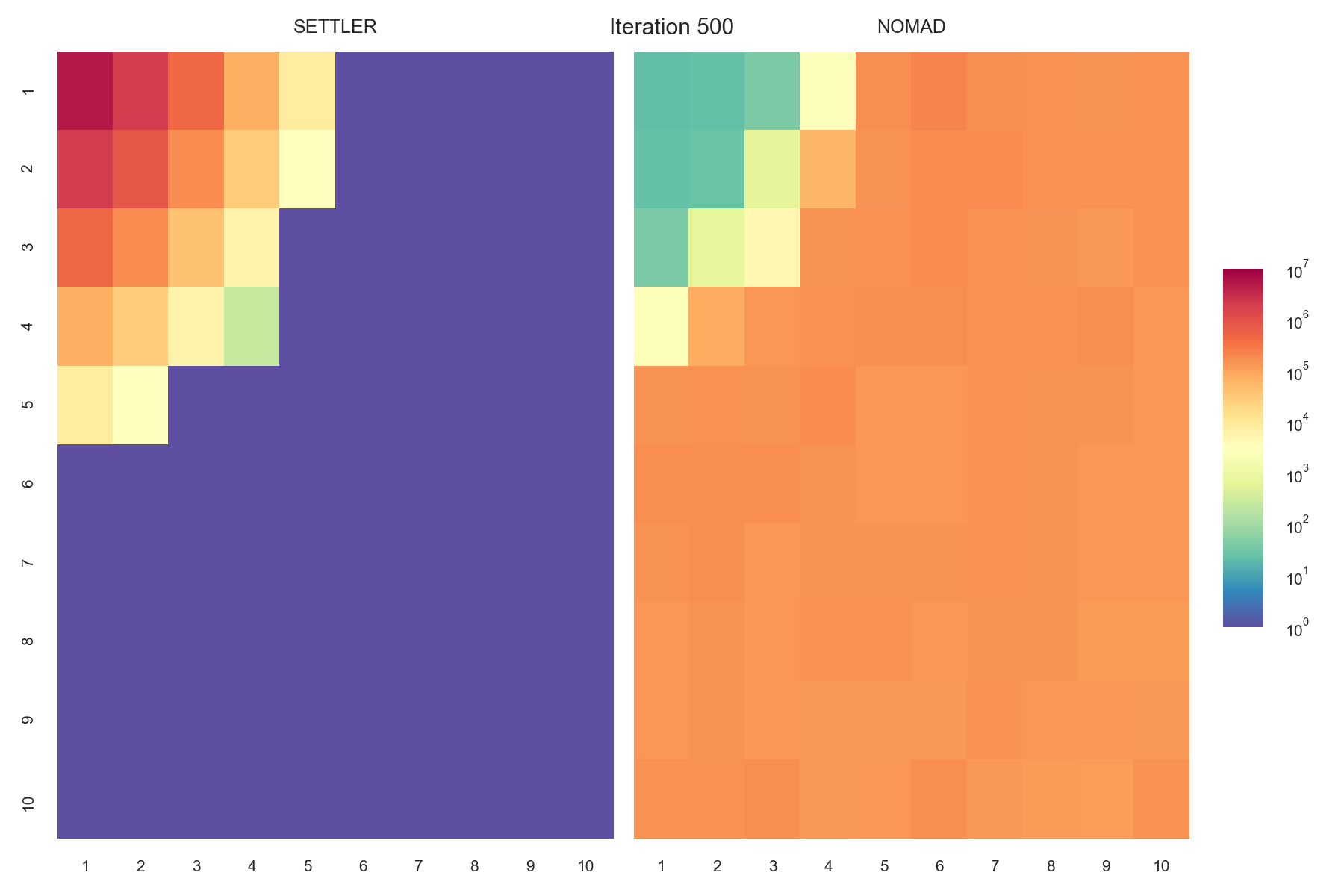

Supplement: Supplementary file 1 [file biology-10-01019-s001.zip › Spatio-temporal dynamics heatmaps/chempenoff_extremelyscarce_lindeath_period1000/0500.png]

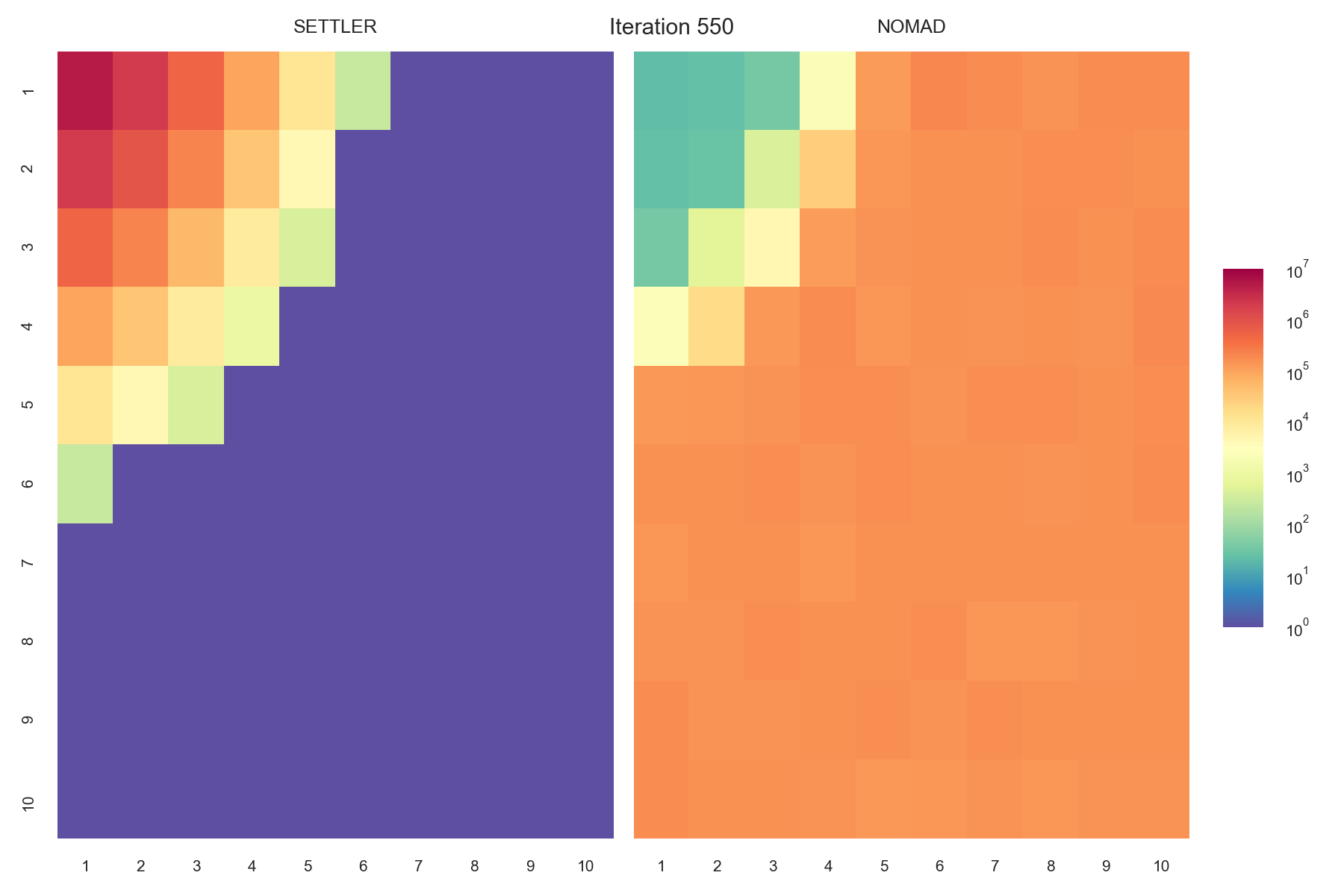

Supplement: Supplementary file 1 [file biology-10-01019-s001.zip › Spatio-temporal dynamics heatmaps/chempenoff_extremelyscarce_lindeath_period1000/0550.png]

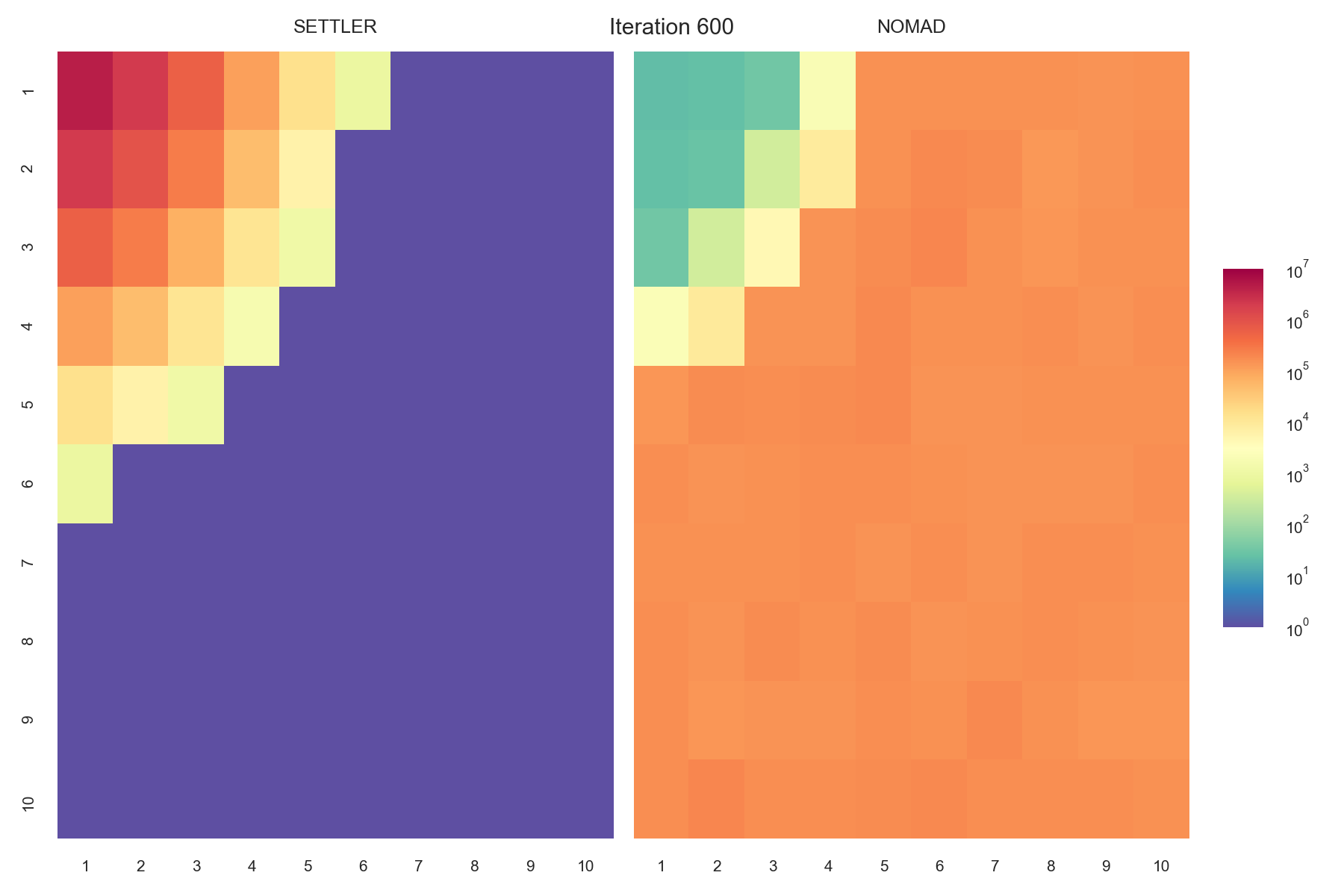

Supplement: Supplementary file 1 [file biology-10-01019-s001.zip › Spatio-temporal dynamics heatmaps/chempenoff_extremelyscarce_lindeath_period1000/0600.png]

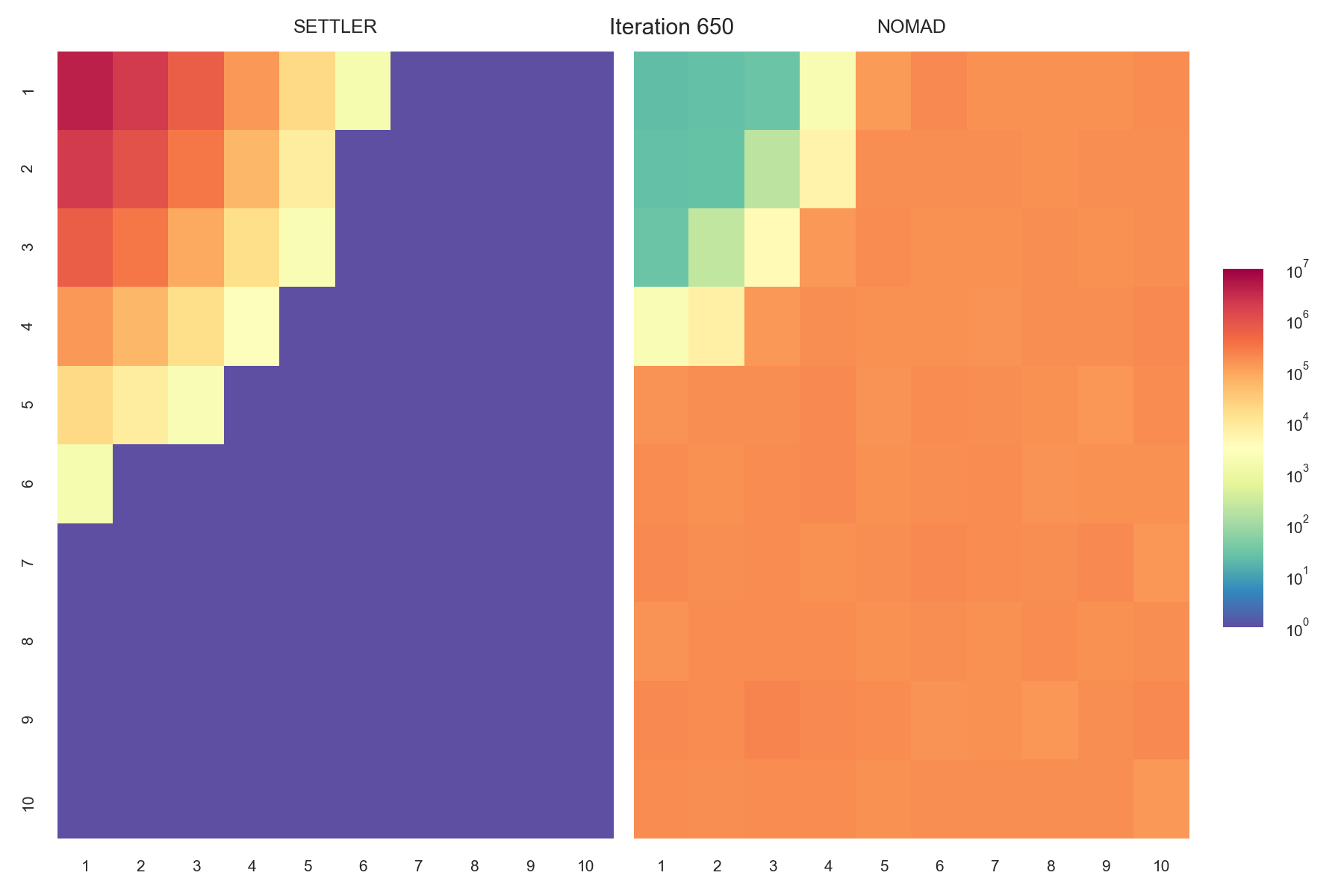

Supplement: Supplementary file 1 [file biology-10-01019-s001.zip › Spatio-temporal dynamics heatmaps/chempenoff_extremelyscarce_lindeath_period1000/0650.png]

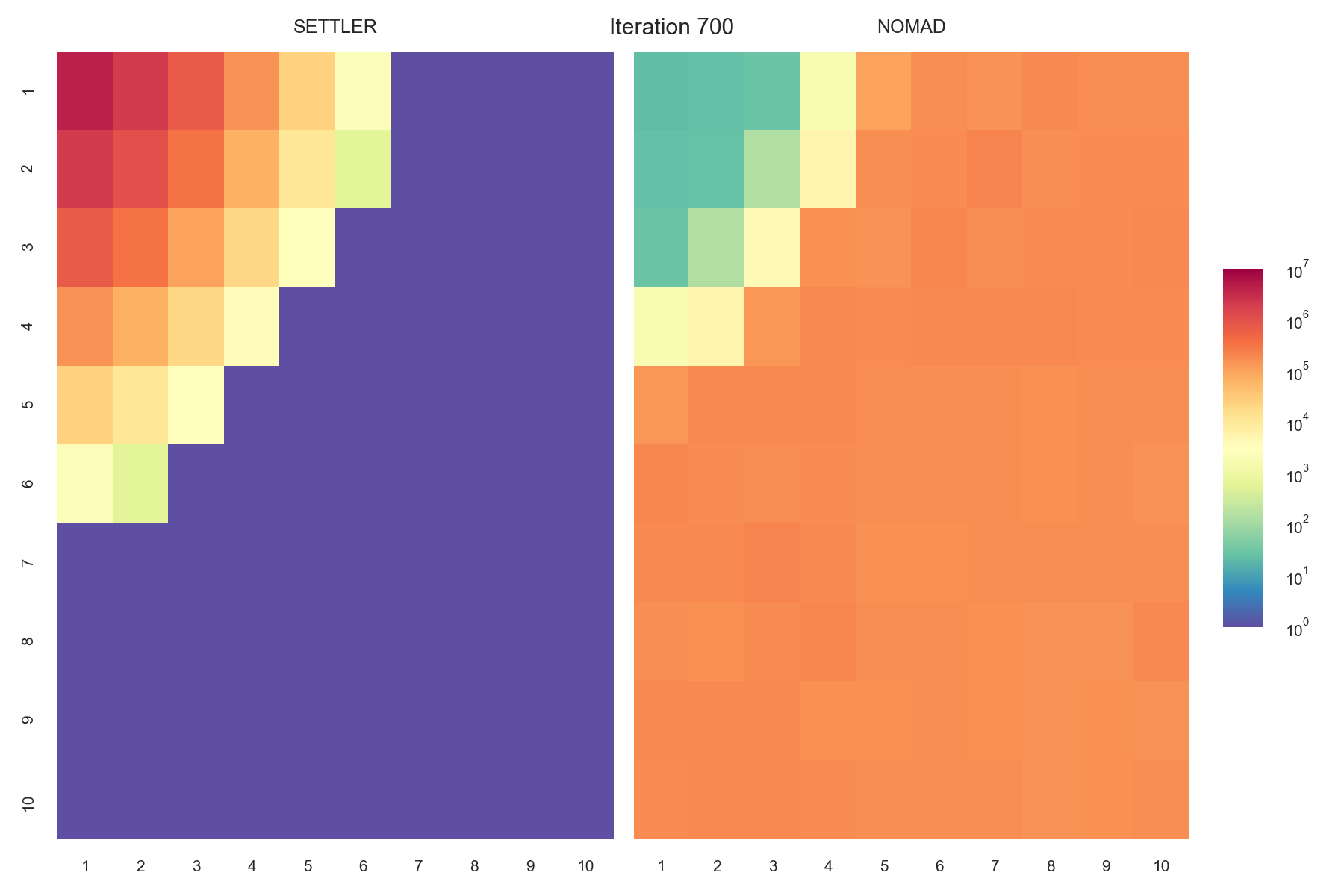

Supplement: Supplementary file 1 [file biology-10-01019-s001.zip › Spatio-temporal dynamics heatmaps/chempenoff_extremelyscarce_lindeath_period1000/0700.png]

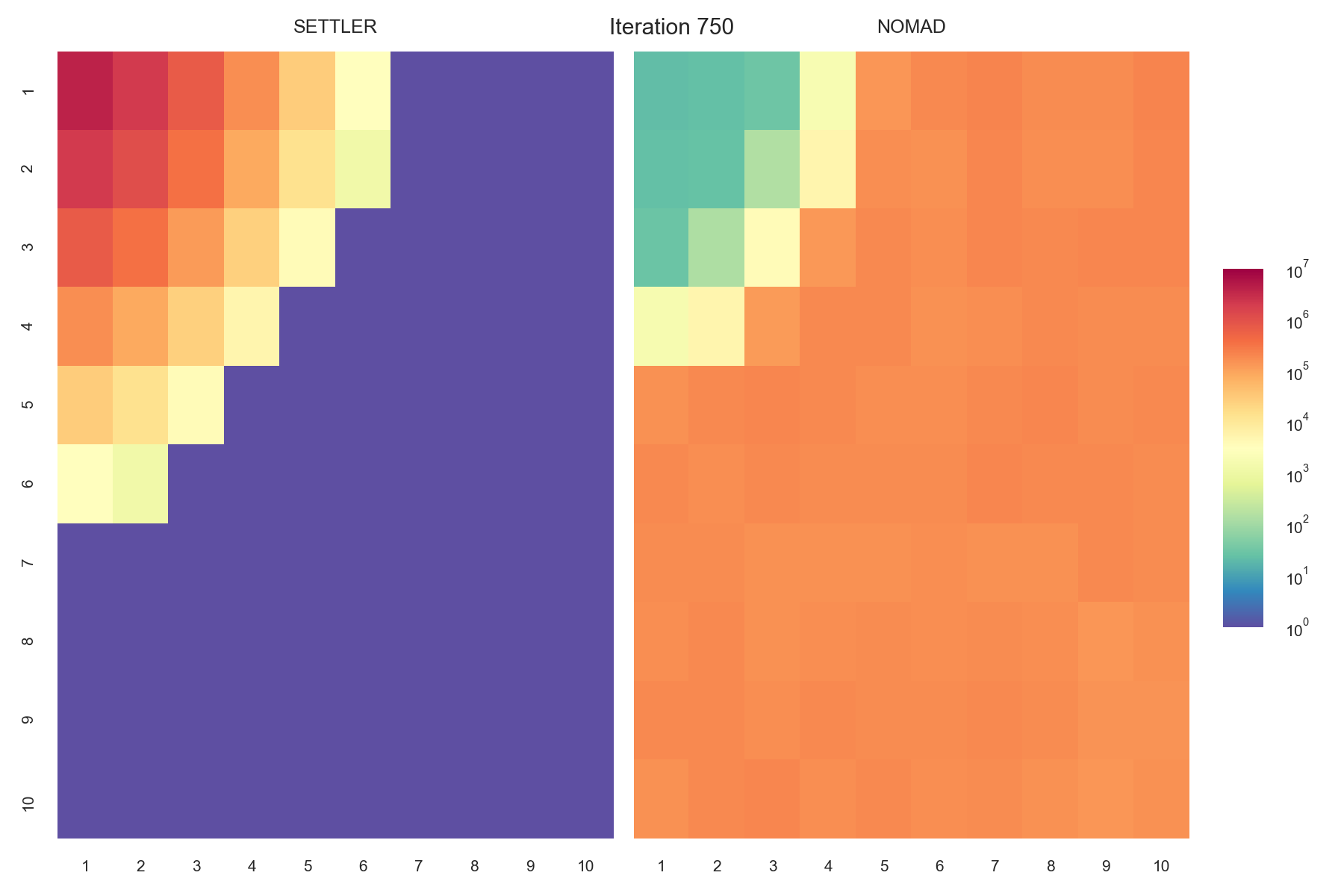

Supplement: Supplementary file 1 [file biology-10-01019-s001.zip › Spatio-temporal dynamics heatmaps/chempenoff_extremelyscarce_lindeath_period1000/0750.png]

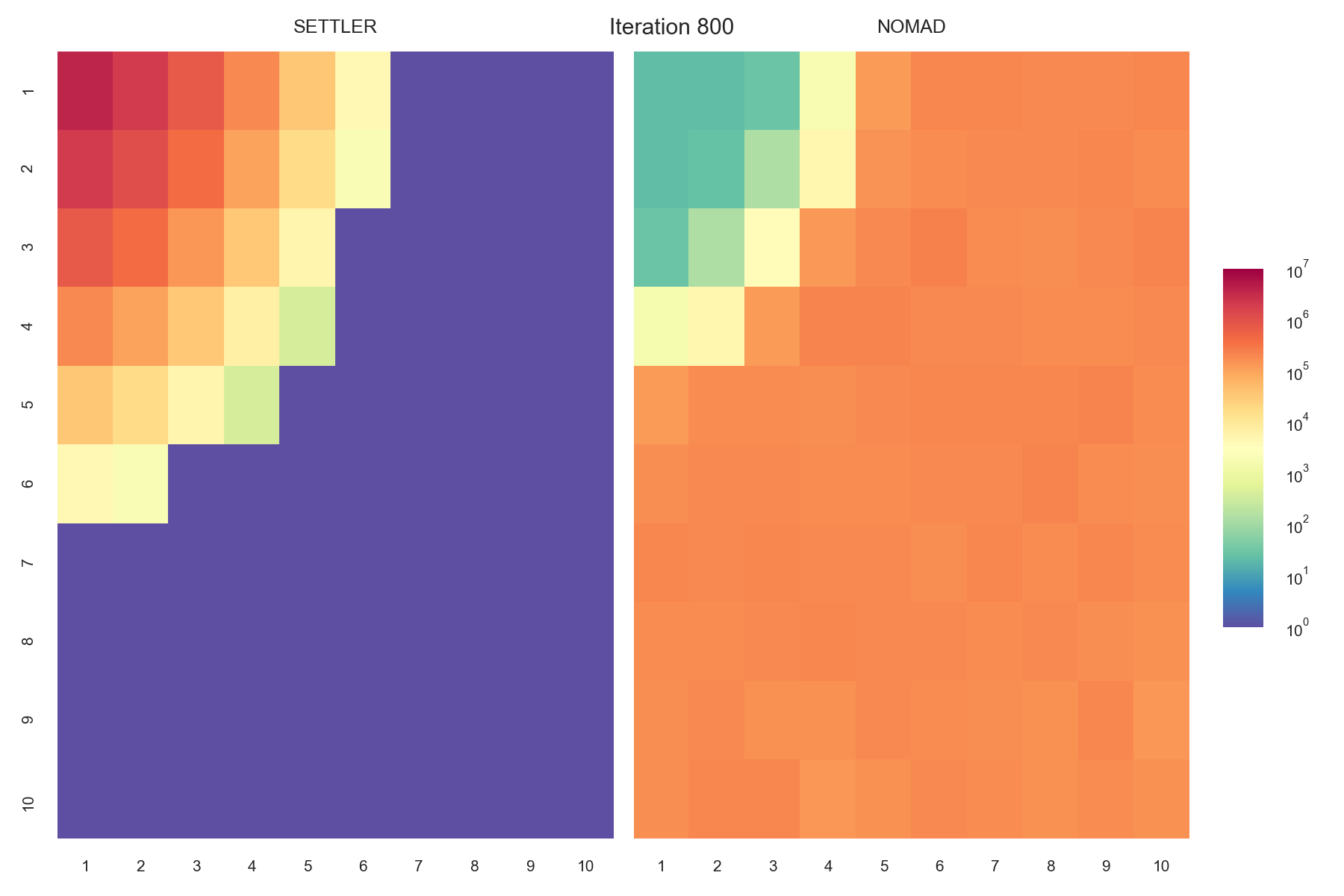

Supplement: Supplementary file 1 [file biology-10-01019-s001.zip › Spatio-temporal dynamics heatmaps/chempenoff_extremelyscarce_lindeath_period1000/0800.png]

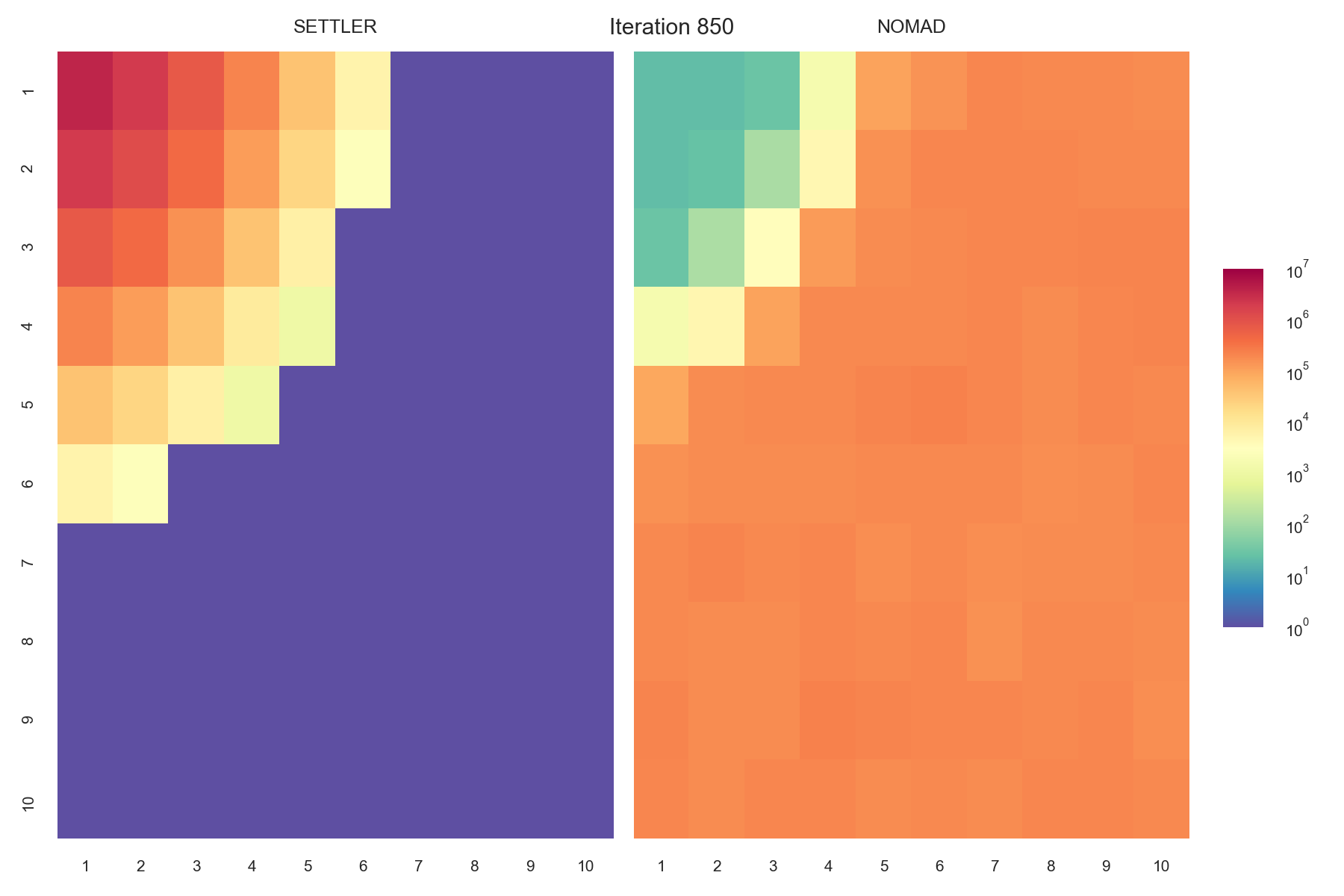

Supplement: Supplementary file 1 [file biology-10-01019-s001.zip › Spatio-temporal dynamics heatmaps/chempenoff_extremelyscarce_lindeath_period1000/0850.png]

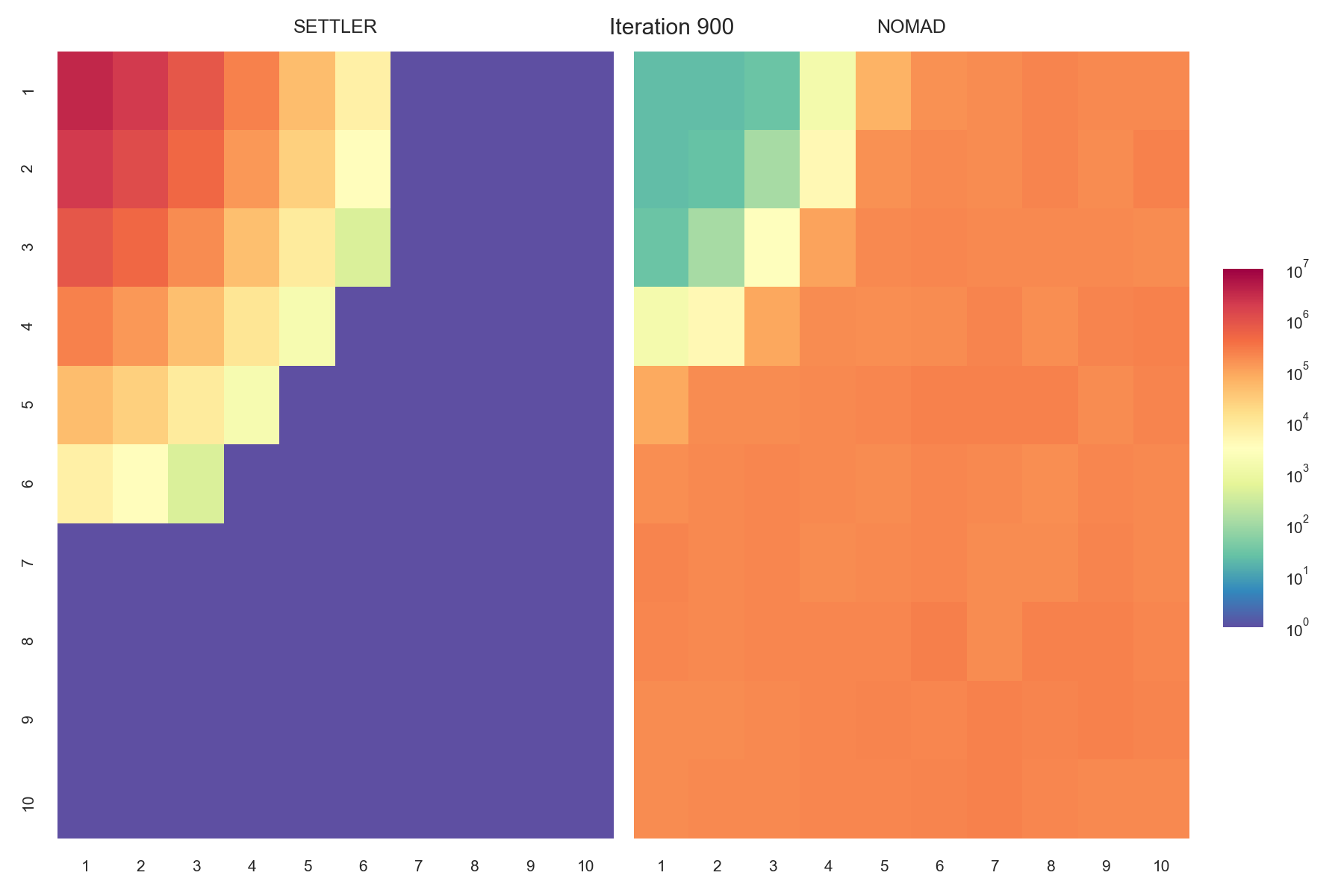

Supplement: Supplementary file 1 [file biology-10-01019-s001.zip › Spatio-temporal dynamics heatmaps/chempenoff_extremelyscarce_lindeath_period1000/0900.png]

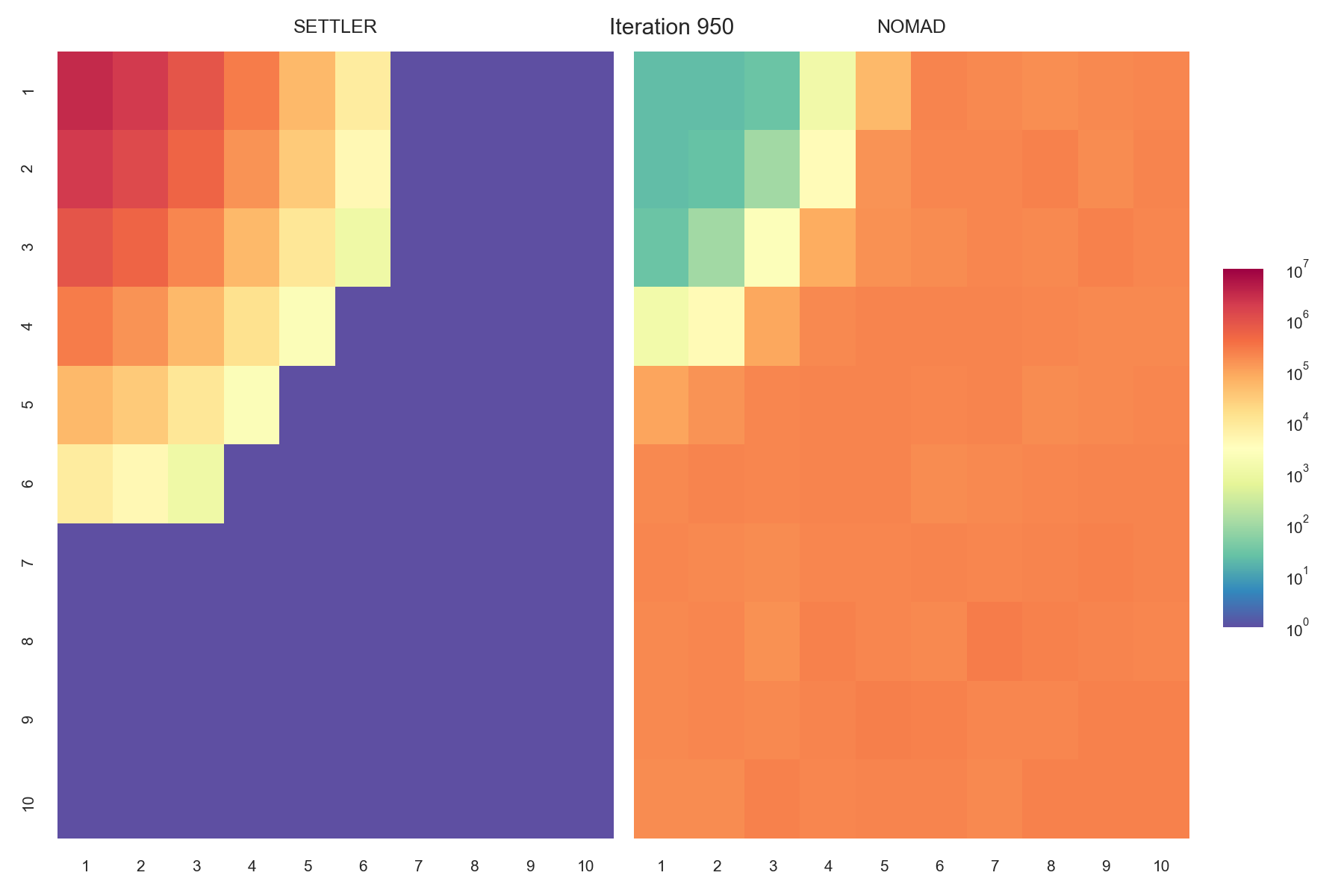

Supplement: Supplementary file 1 [file biology-10-01019-s001.zip › Spatio-temporal dynamics heatmaps/chempenoff_extremelyscarce_lindeath_period1000/0950.png]

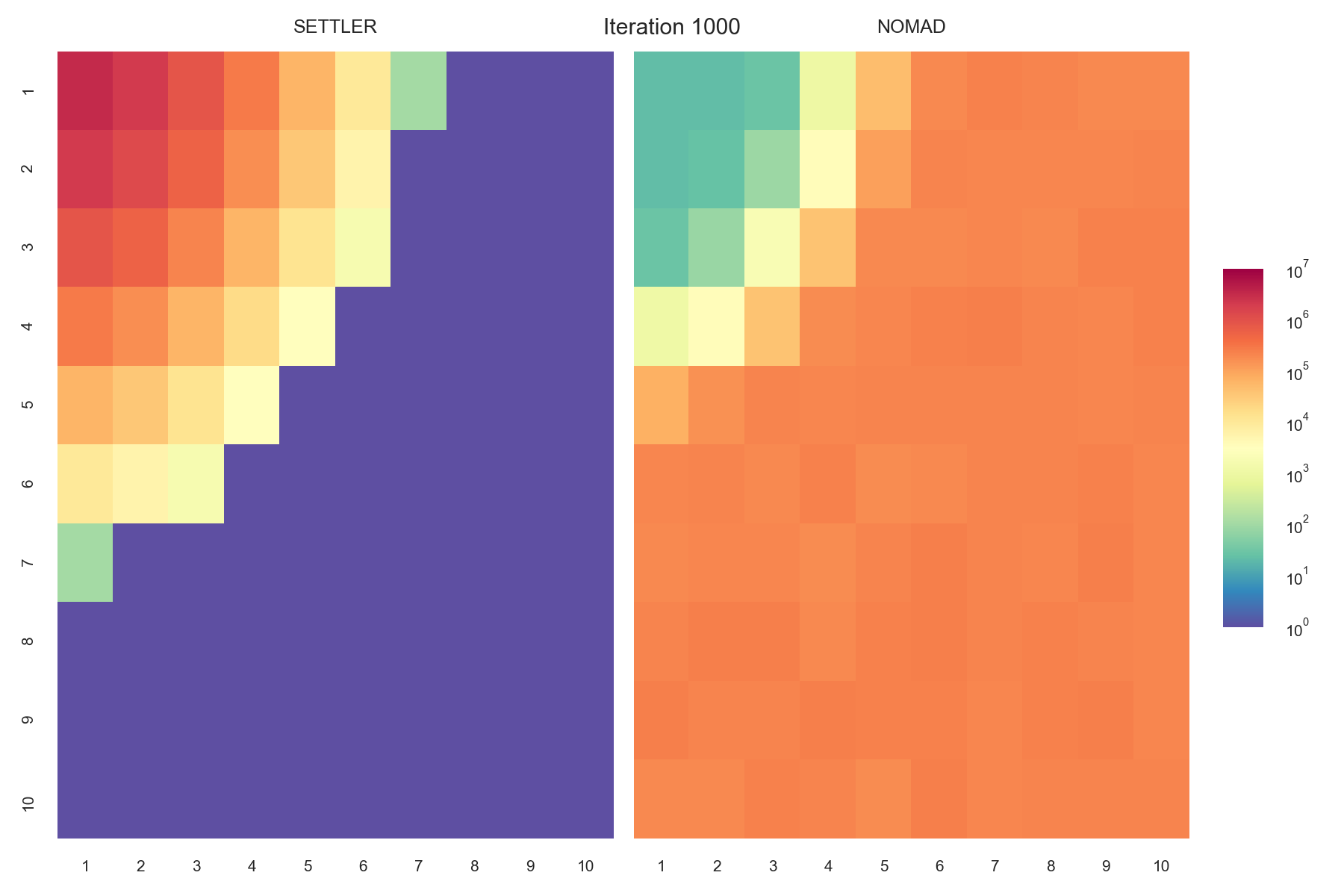

Supplement: Supplementary file 1 [file biology-10-01019-s001.zip › Spatio-temporal dynamics heatmaps/chempenoff_extremelyscarce_lindeath_period1000/1000.png]

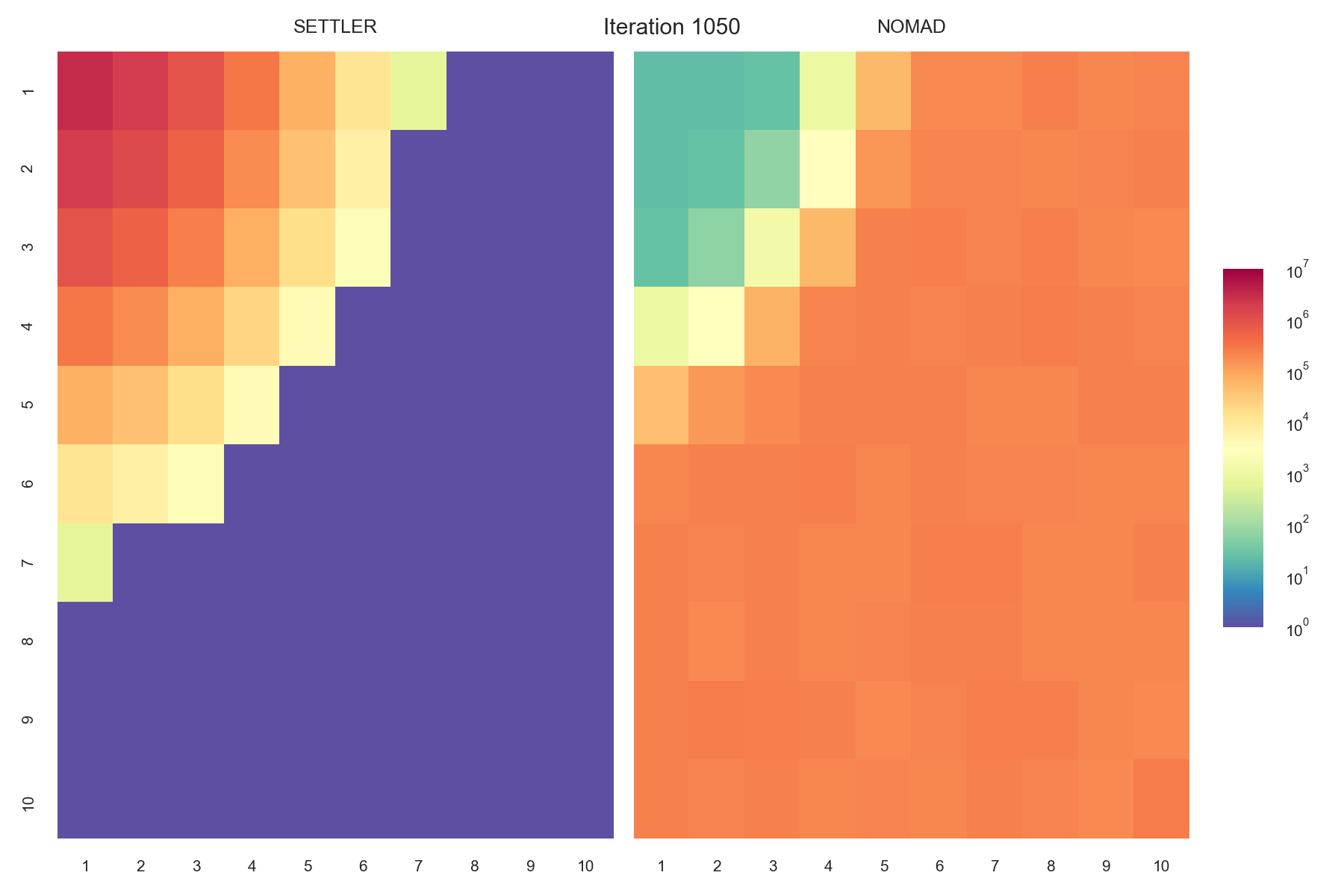

Supplement: Supplementary file 1 [file biology-10-01019-s001.zip › Spatio-temporal dynamics heatmaps/chempenoff_extremelyscarce_lindeath_period1000/1050.png]

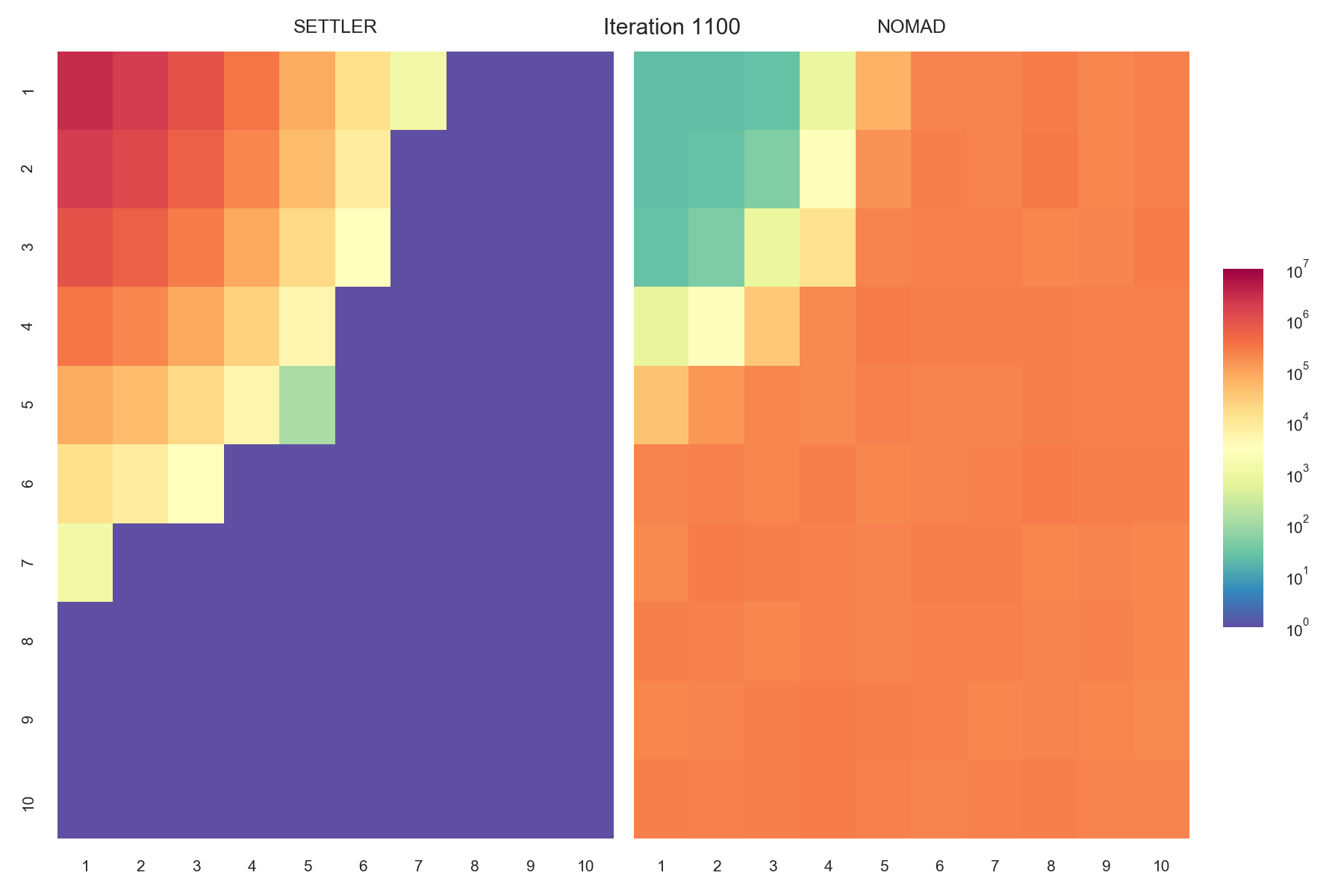

Supplement: Supplementary file 1 [file biology-10-01019-s001.zip › Spatio-temporal dynamics heatmaps/chempenoff_extremelyscarce_lindeath_period1000/1100.png]

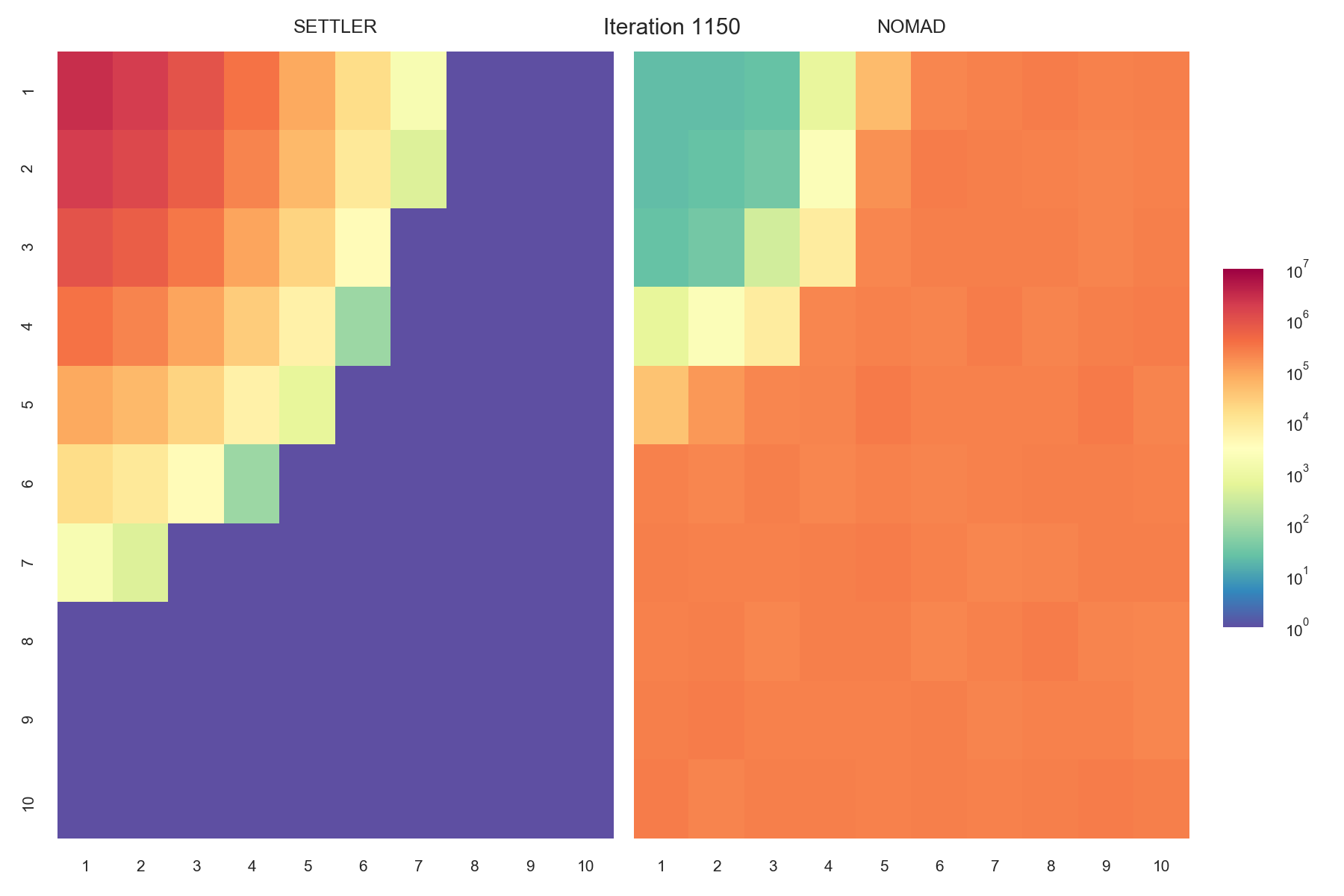

Supplement: Supplementary file 1 [file biology-10-01019-s001.zip › Spatio-temporal dynamics heatmaps/chempenoff_extremelyscarce_lindeath_period1000/1150.png]

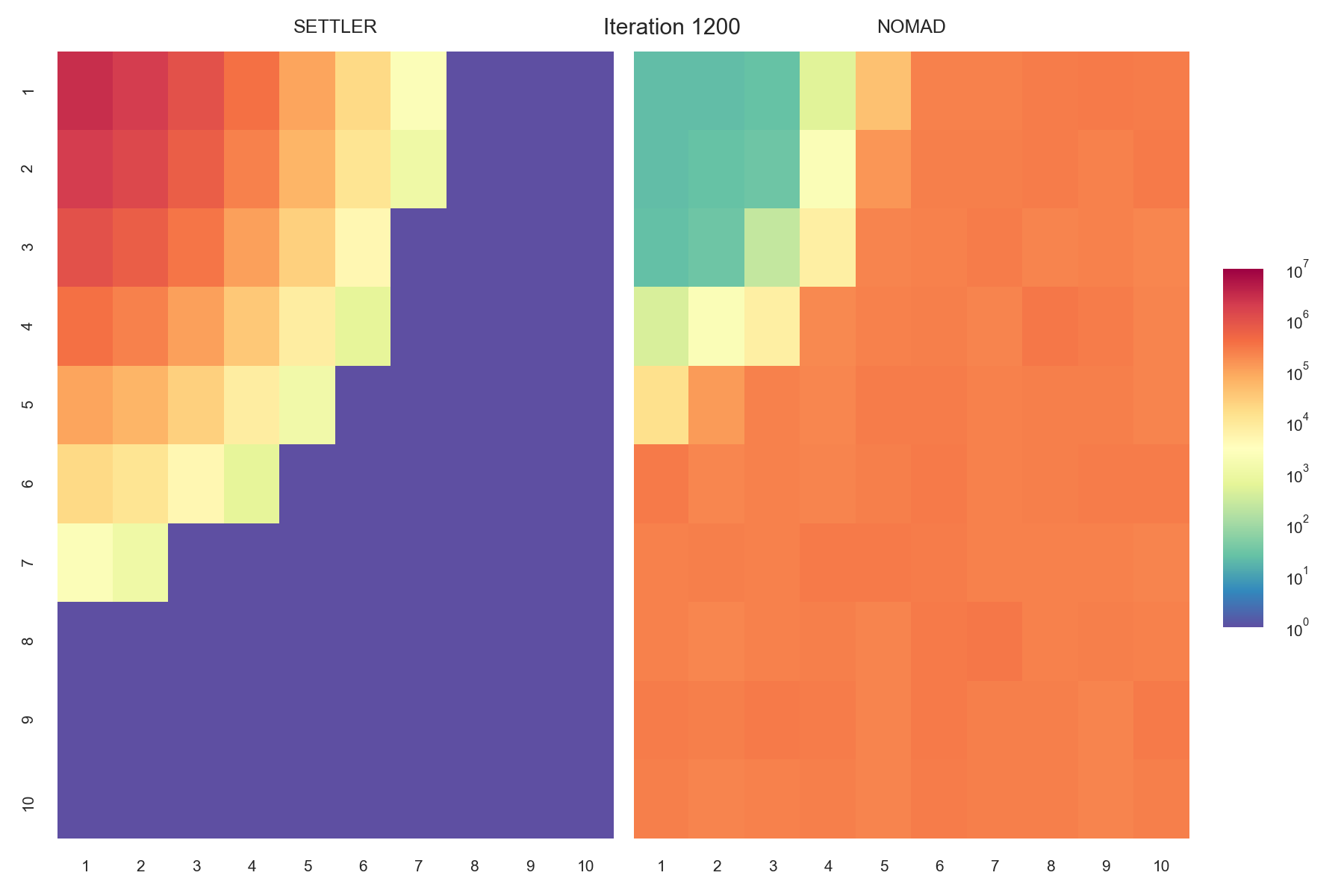

Supplement: Supplementary file 1 [file biology-10-01019-s001.zip › Spatio-temporal dynamics heatmaps/chempenoff_extremelyscarce_lindeath_period1000/1200.png]

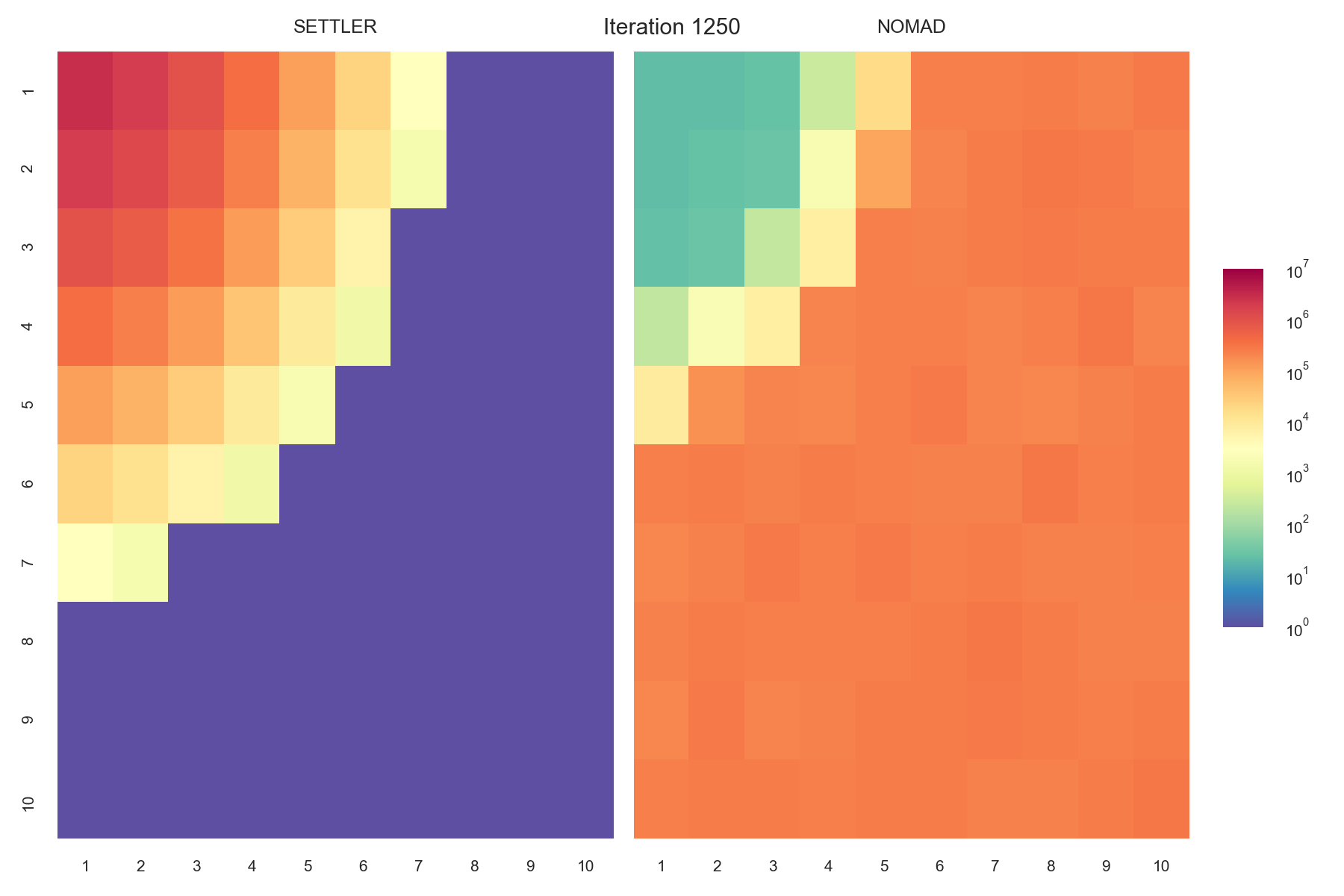

Supplement: Supplementary file 1 [file biology-10-01019-s001.zip › Spatio-temporal dynamics heatmaps/chempenoff_extremelyscarce_lindeath_period1000/1250.png]

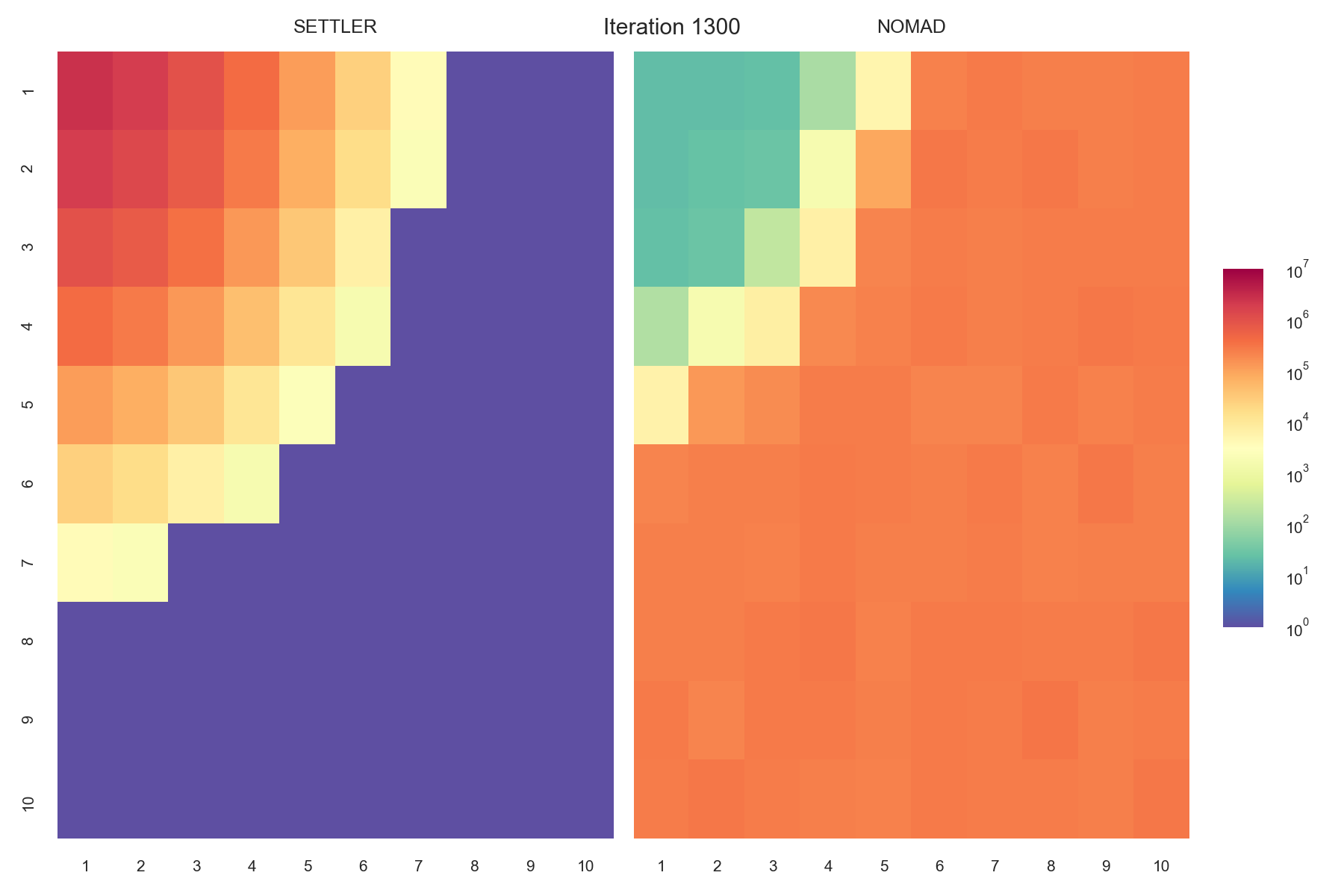

Supplement: Supplementary file 1 [file biology-10-01019-s001.zip › Spatio-temporal dynamics heatmaps/chempenoff_extremelyscarce_lindeath_period1000/1300.png]

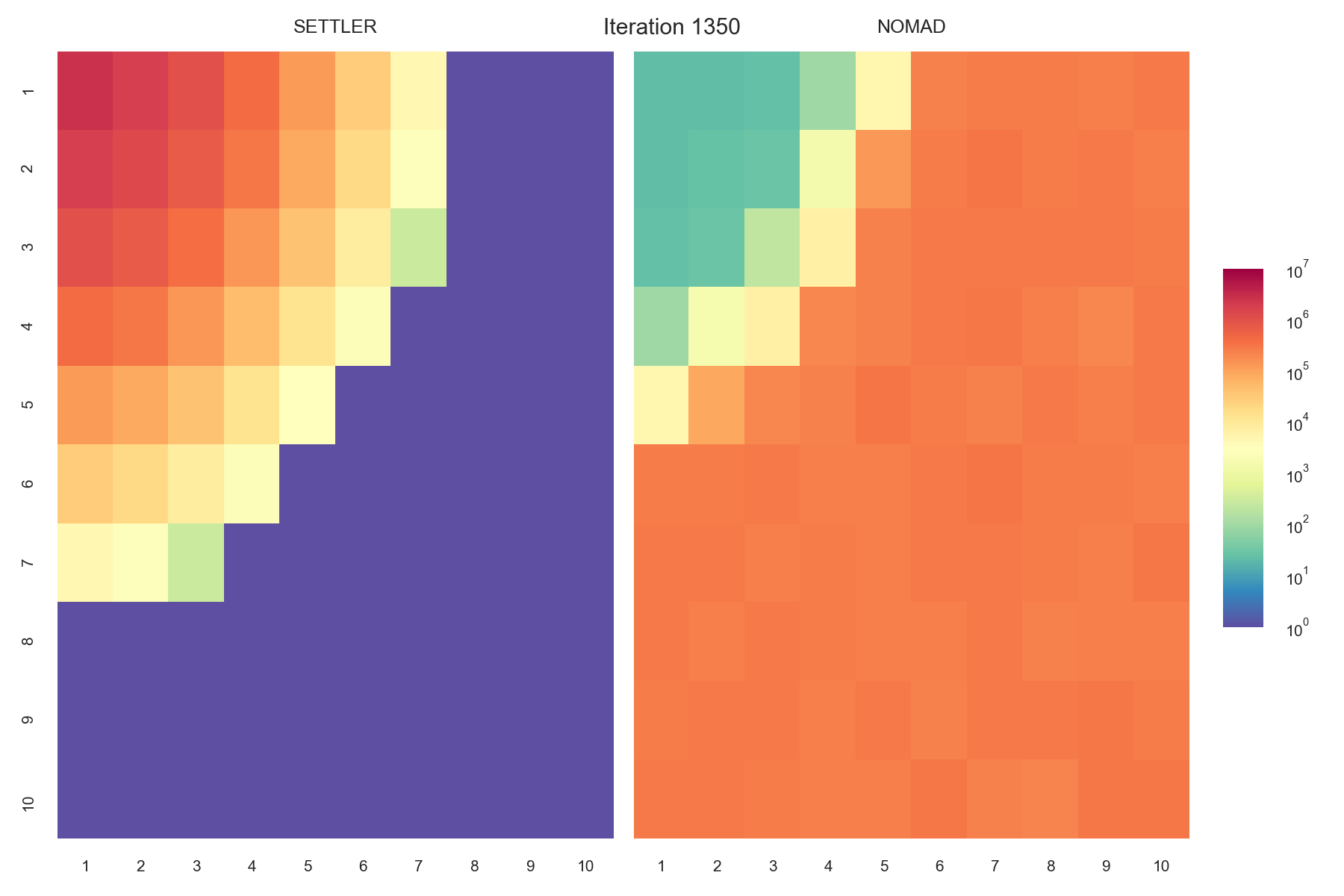

Supplement: Supplementary file 1 [file biology-10-01019-s001.zip › Spatio-temporal dynamics heatmaps/chempenoff_extremelyscarce_lindeath_period1000/1350.png]

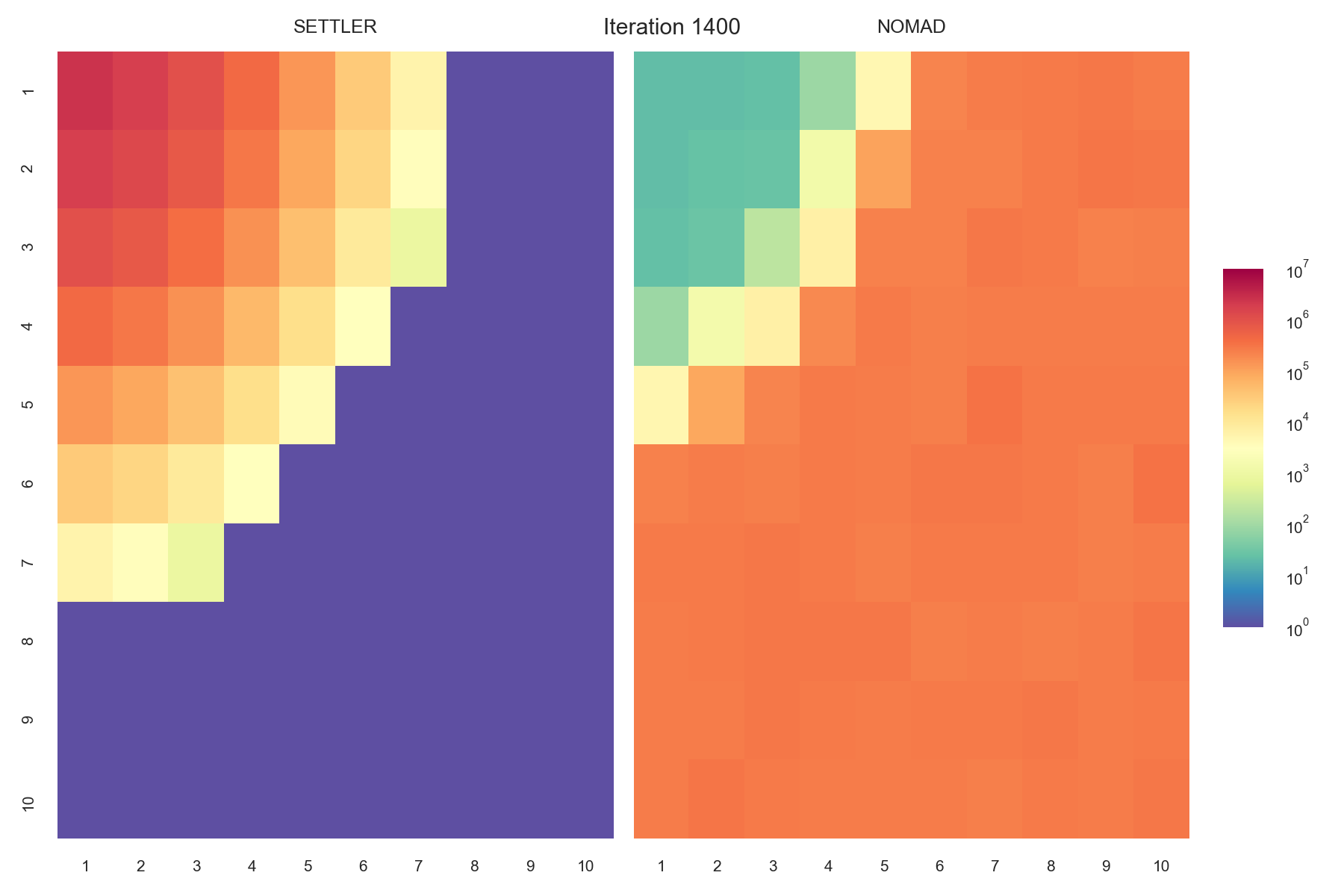

Supplement: Supplementary file 1 [file biology-10-01019-s001.zip › Spatio-temporal dynamics heatmaps/chempenoff_extremelyscarce_lindeath_period1000/1400.png]

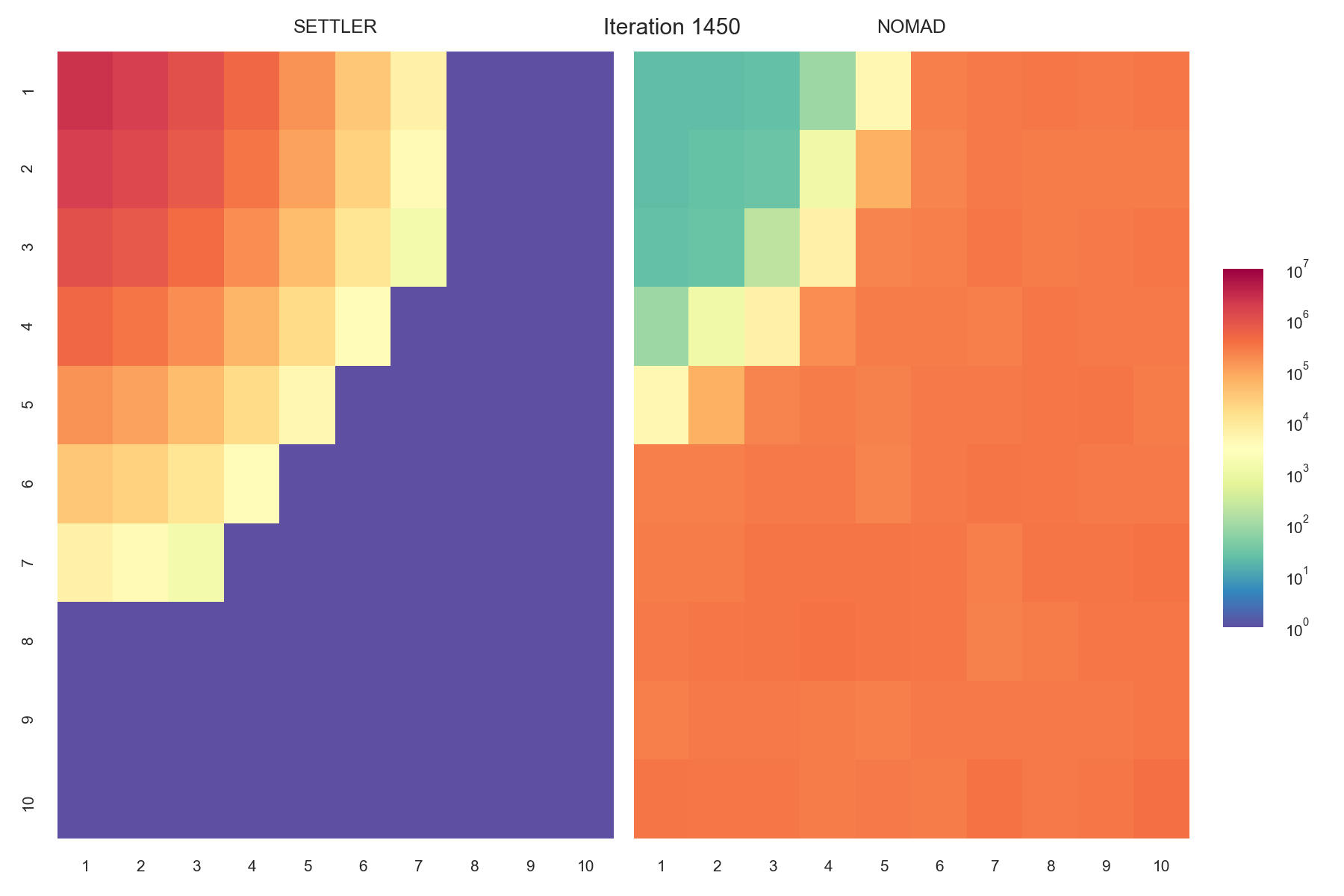

Supplement: Supplementary file 1 [file biology-10-01019-s001.zip › Spatio-temporal dynamics heatmaps/chempenoff_extremelyscarce_lindeath_period1000/1450.png]

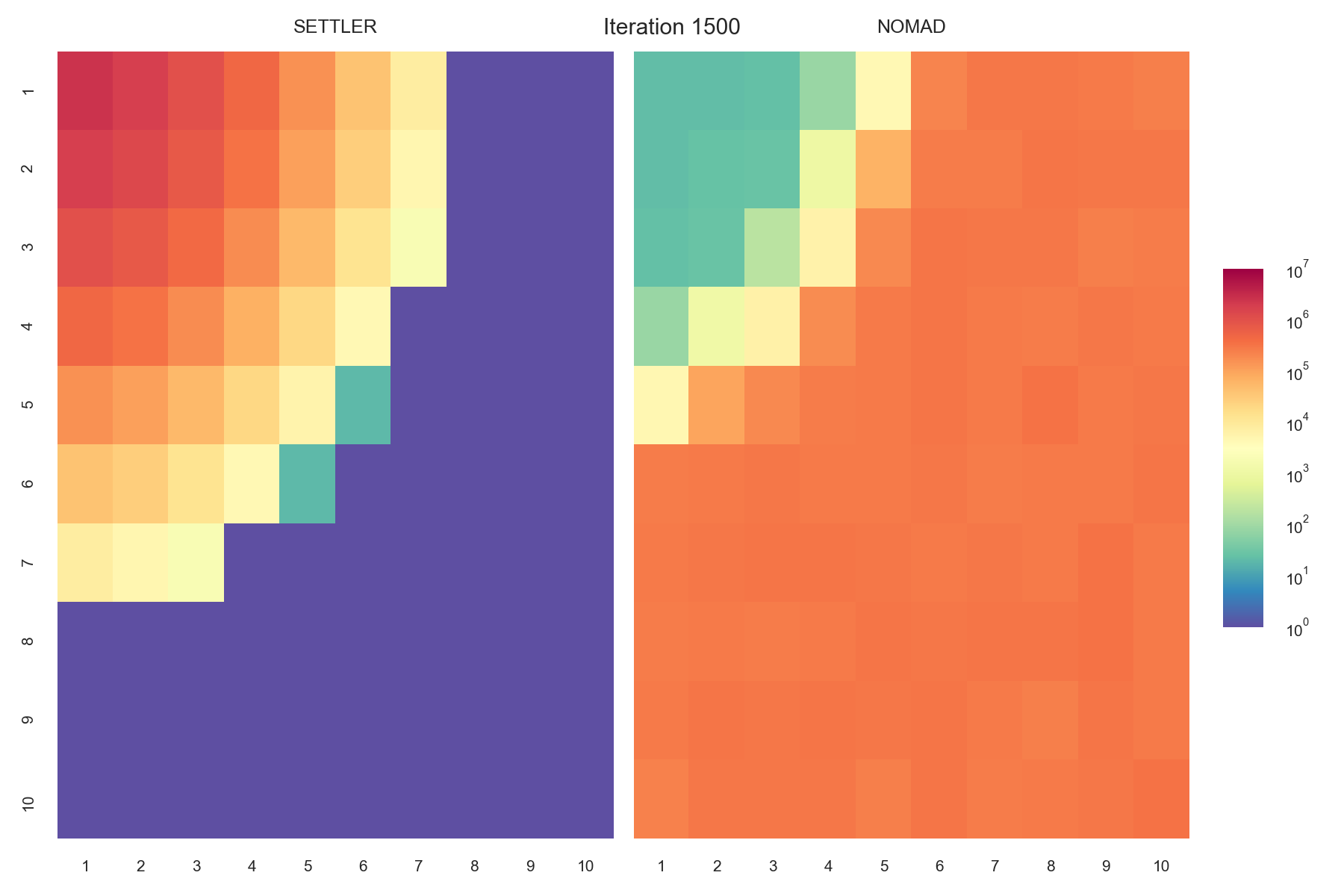

Supplement: Supplementary file 1 [file biology-10-01019-s001.zip › Spatio-temporal dynamics heatmaps/chempenoff_extremelyscarce_lindeath_period1000/1500.png]

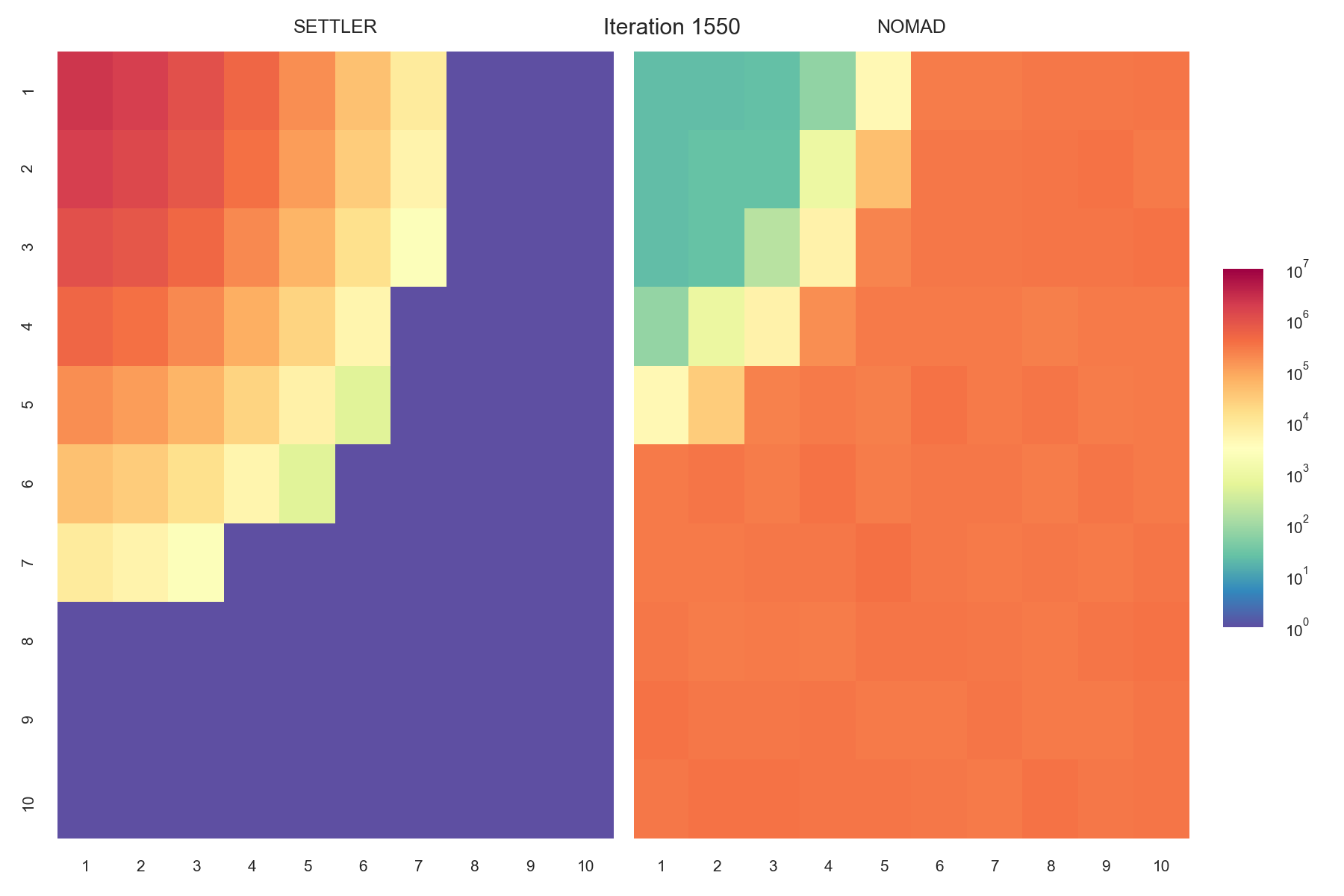

Supplement: Supplementary file 1 [file biology-10-01019-s001.zip › Spatio-temporal dynamics heatmaps/chempenoff_extremelyscarce_lindeath_period1000/1550.png]

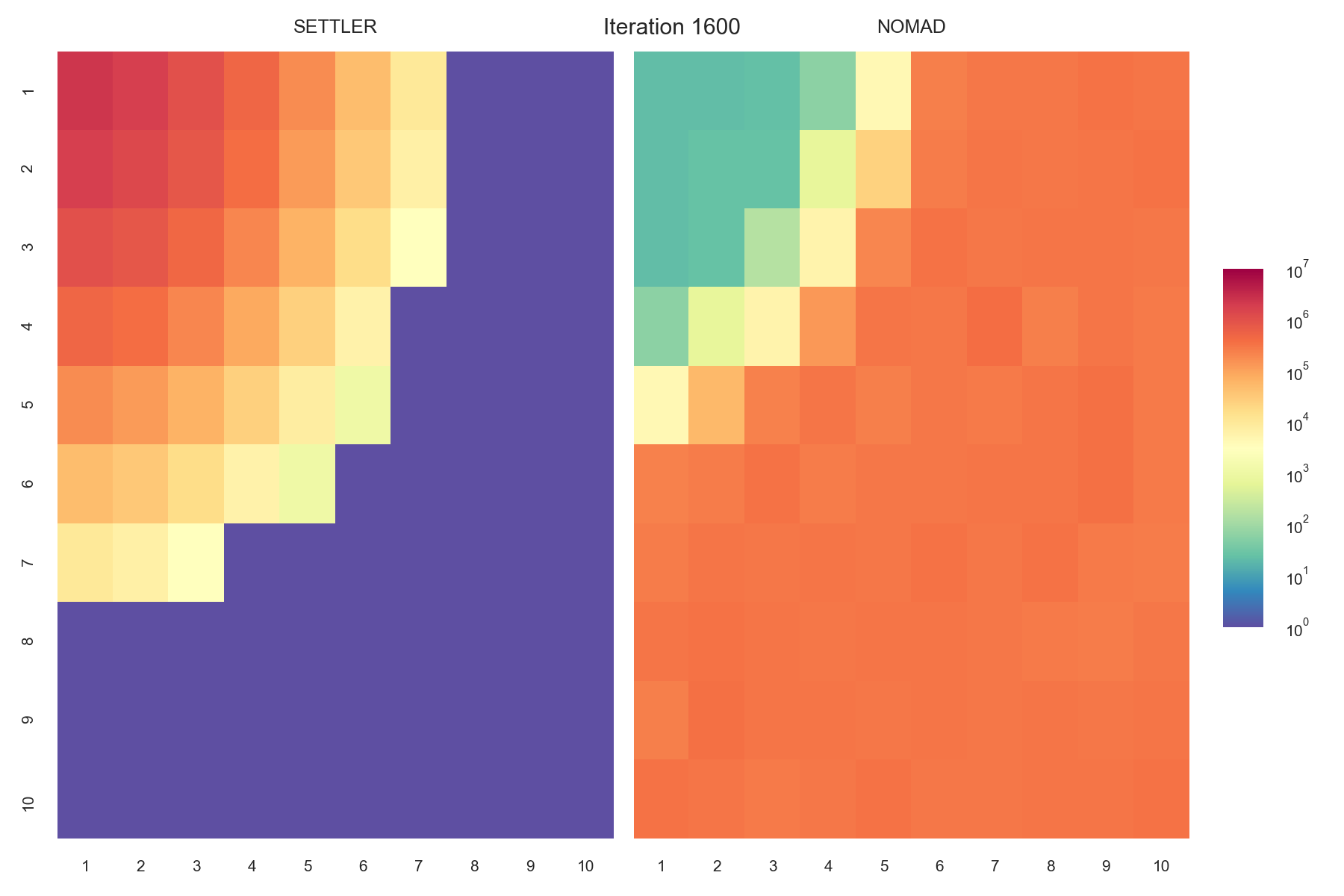

Supplement: Supplementary file 1 [file biology-10-01019-s001.zip › Spatio-temporal dynamics heatmaps/chempenoff_extremelyscarce_lindeath_period1000/1600.png]

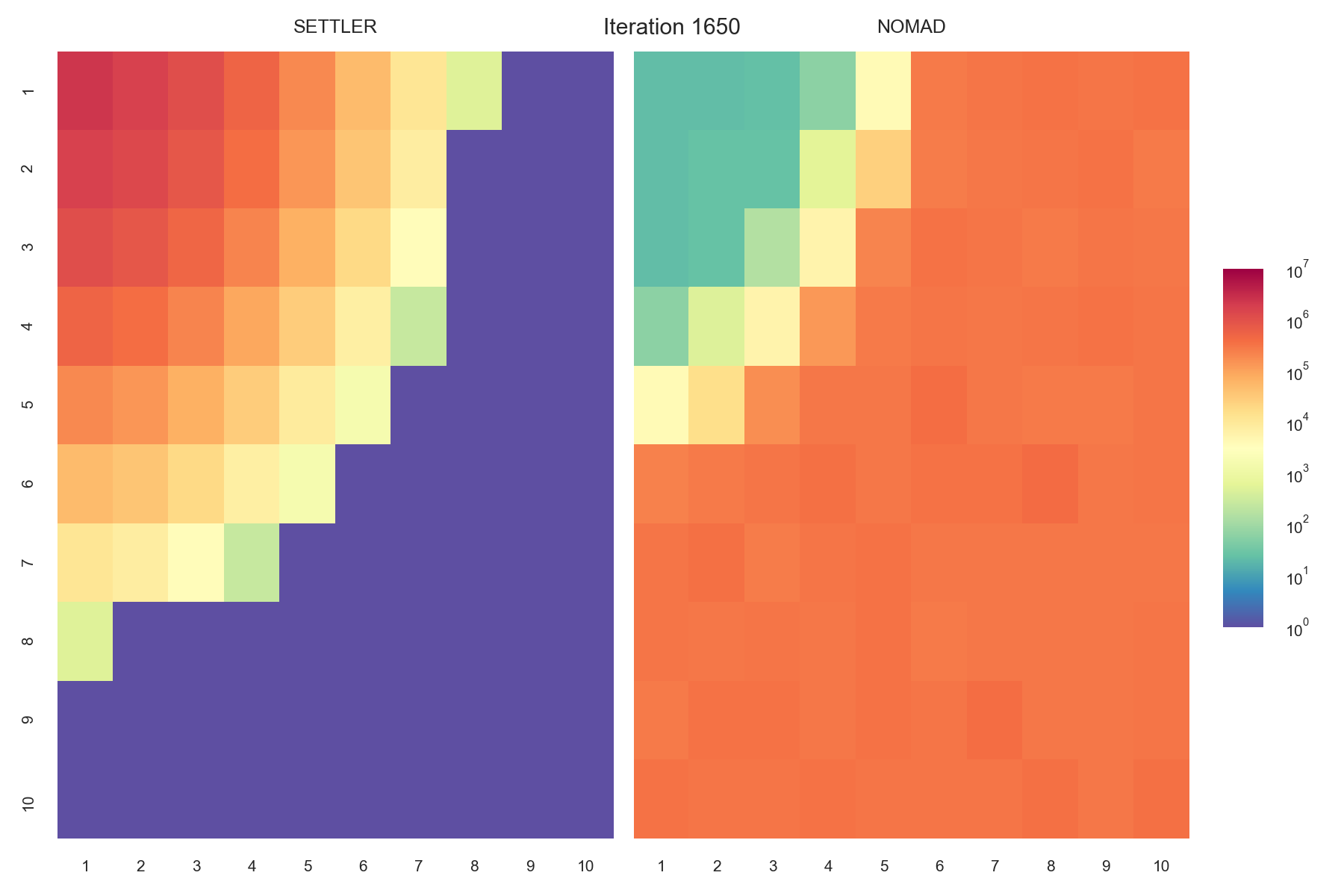

Supplement: Supplementary file 1 [file biology-10-01019-s001.zip › Spatio-temporal dynamics heatmaps/chempenoff_extremelyscarce_lindeath_period1000/1650.png]

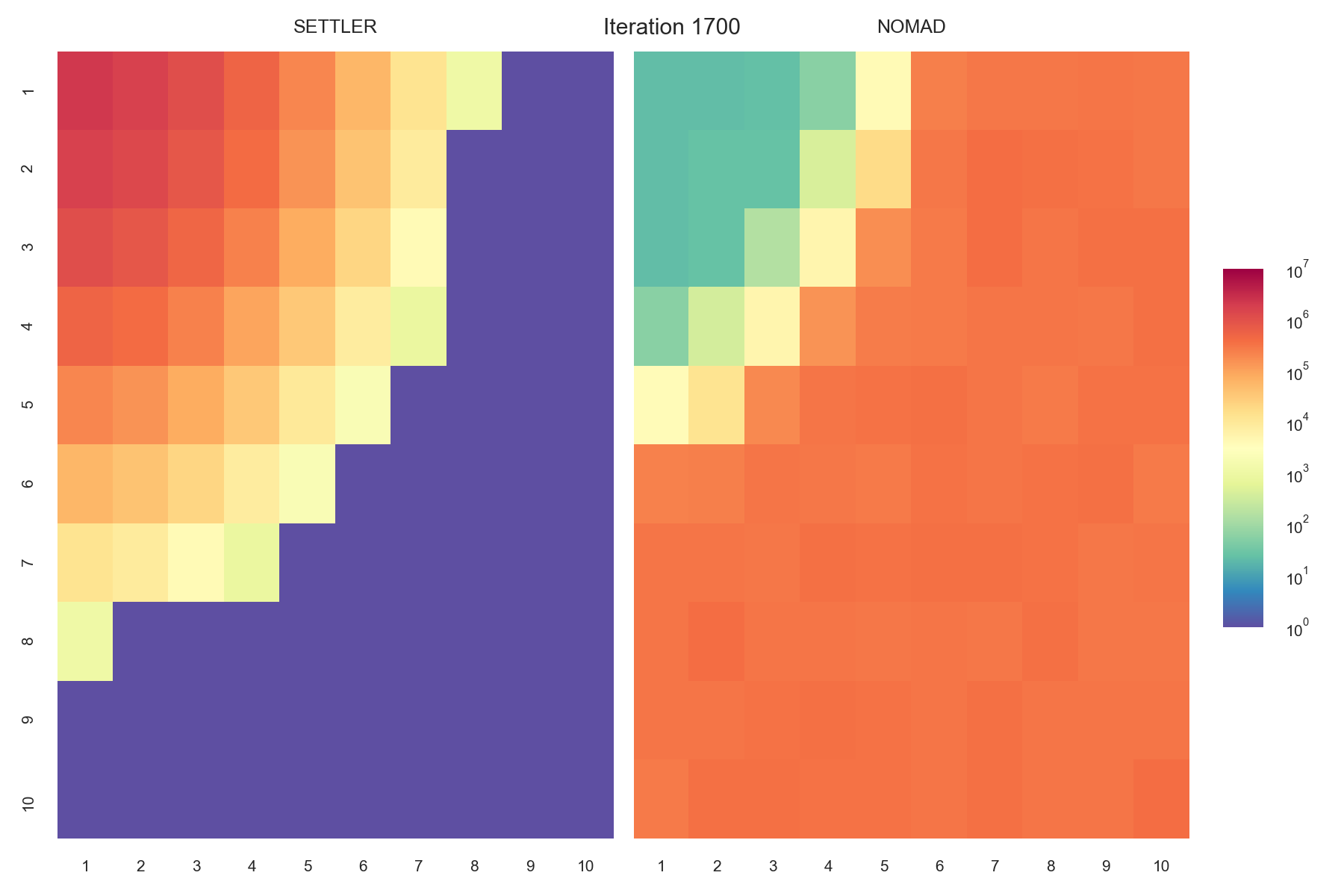

Supplement: Supplementary file 1 [file biology-10-01019-s001.zip › Spatio-temporal dynamics heatmaps/chempenoff_extremelyscarce_lindeath_period1000/1700.png]

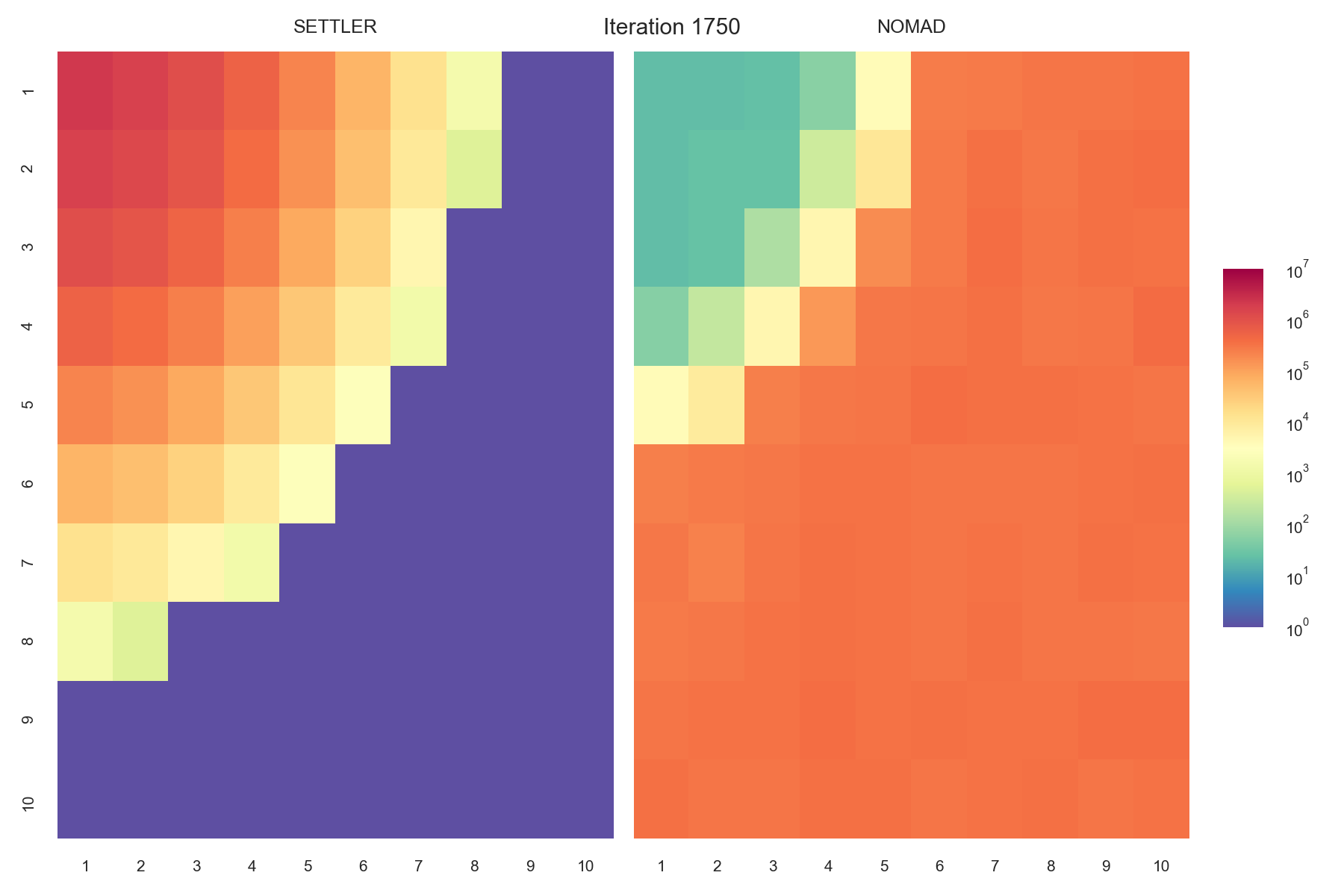

Supplement: Supplementary file 1 [file biology-10-01019-s001.zip › Spatio-temporal dynamics heatmaps/chempenoff_extremelyscarce_lindeath_period1000/1750.png]

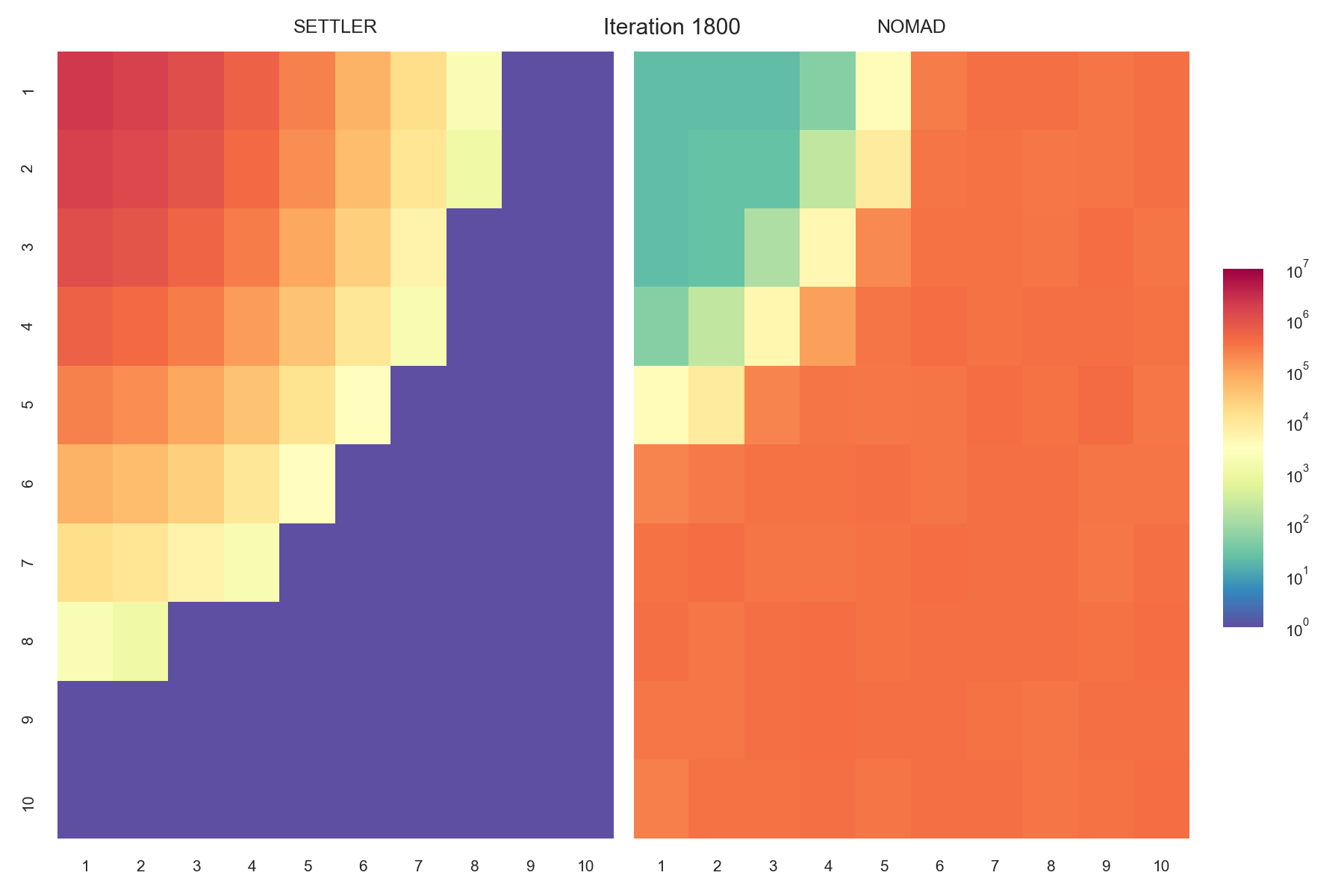

Supplement: Supplementary file 1 [file biology-10-01019-s001.zip › Spatio-temporal dynamics heatmaps/chempenoff_extremelyscarce_lindeath_period1000/1800.png]

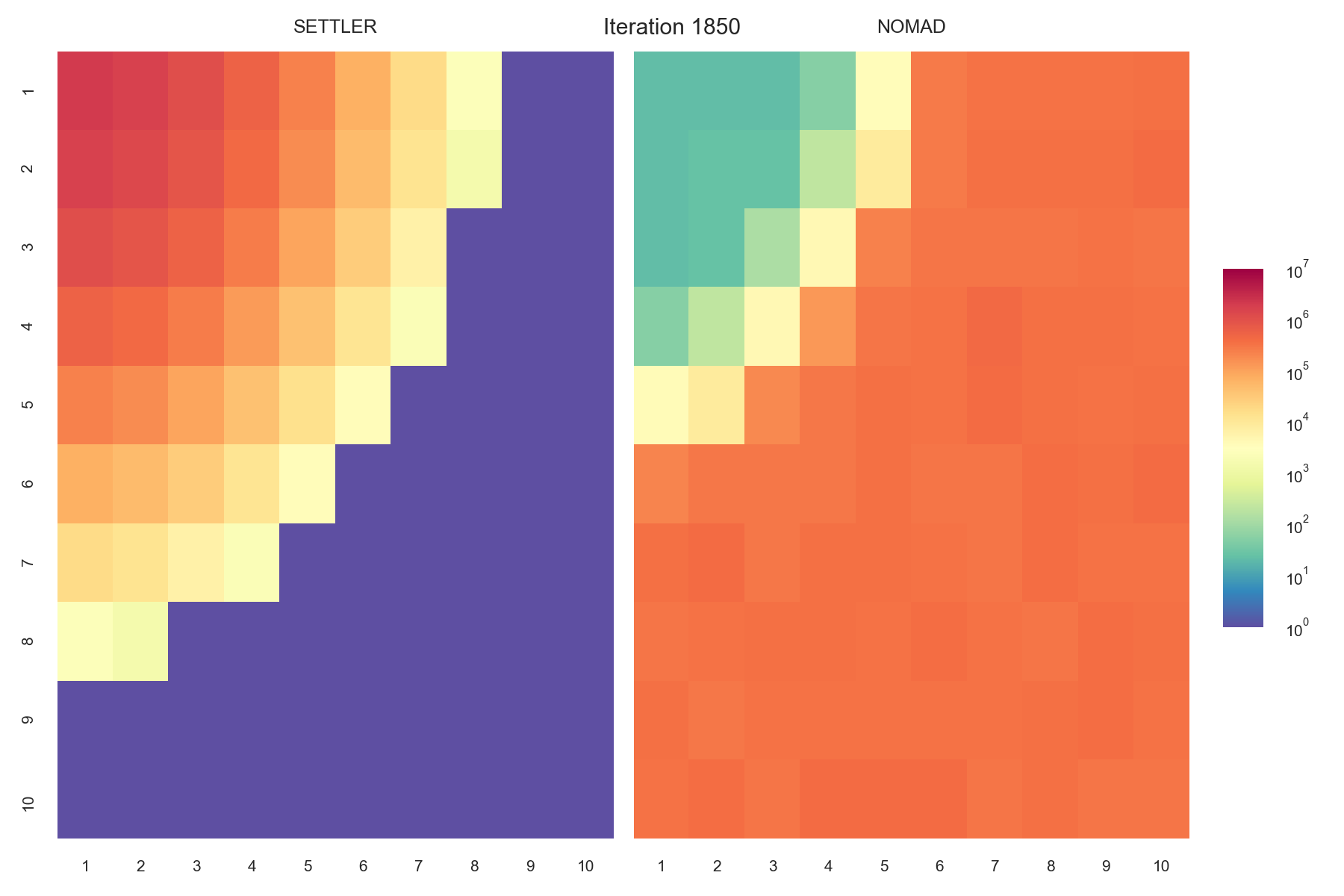

Supplement: Supplementary file 1 [file biology-10-01019-s001.zip › Spatio-temporal dynamics heatmaps/chempenoff_extremelyscarce_lindeath_period1000/1850.png]

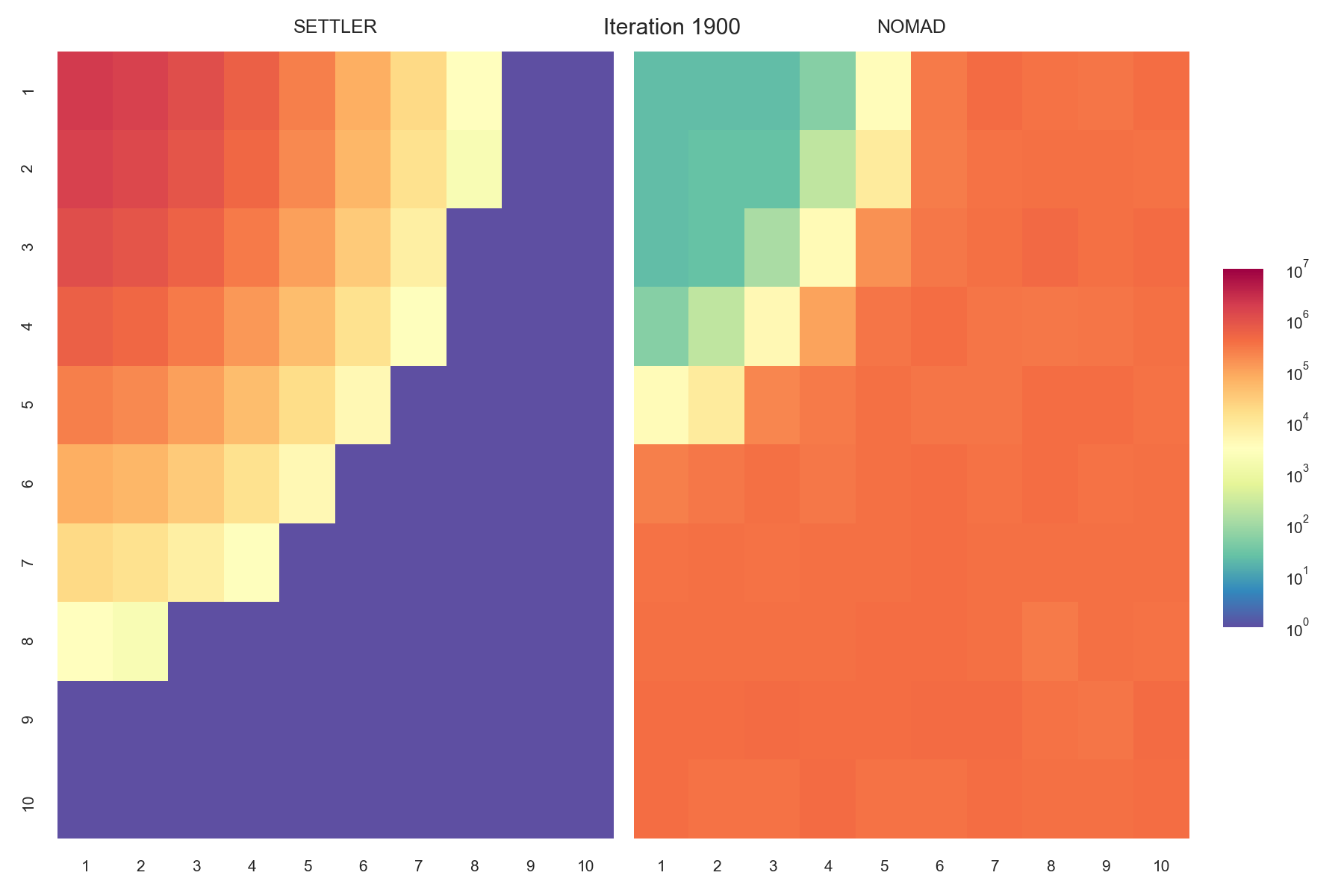

Supplement: Supplementary file 1 [file biology-10-01019-s001.zip › Spatio-temporal dynamics heatmaps/chempenoff_extremelyscarce_lindeath_period1000/1900.png]

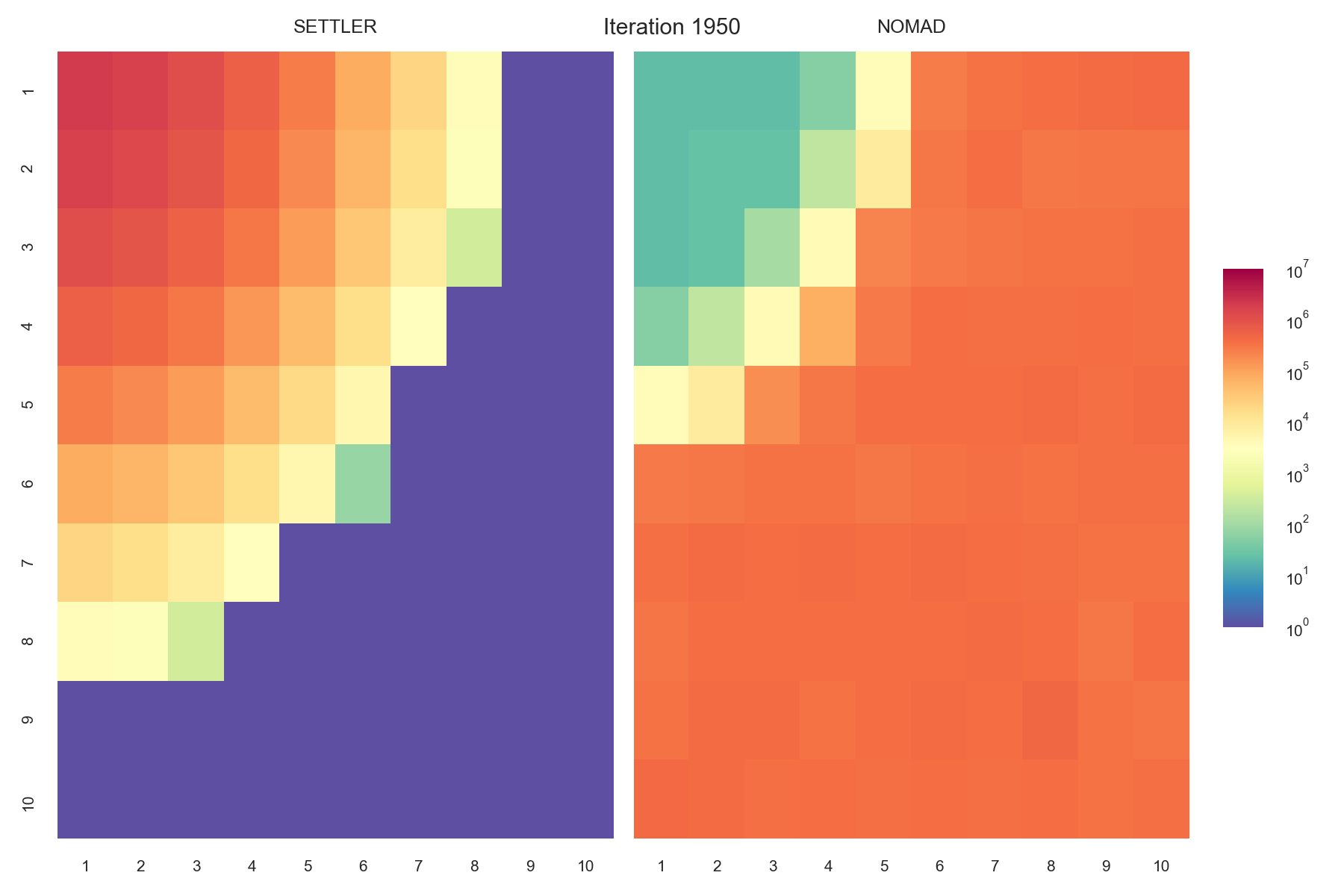

Supplement: Supplementary file 1 [file biology-10-01019-s001.zip › Spatio-temporal dynamics heatmaps/chempenoff_extremelyscarce_lindeath_period1000/1950.png]

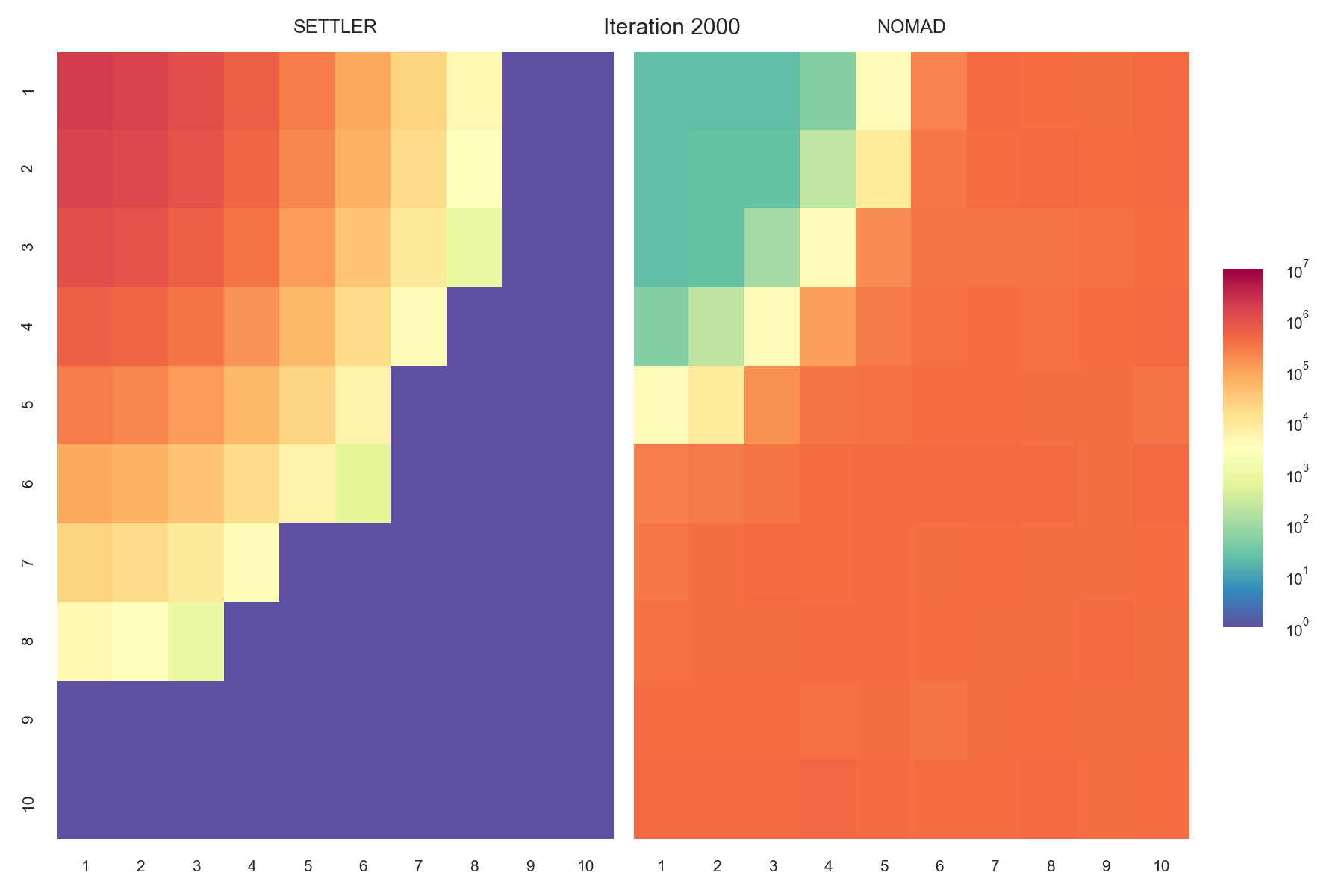

Supplement: Supplementary file 1 [file biology-10-01019-s001.zip › Spatio-temporal dynamics heatmaps/chempenoff_extremelyscarce_lindeath_period1000/2000.png]

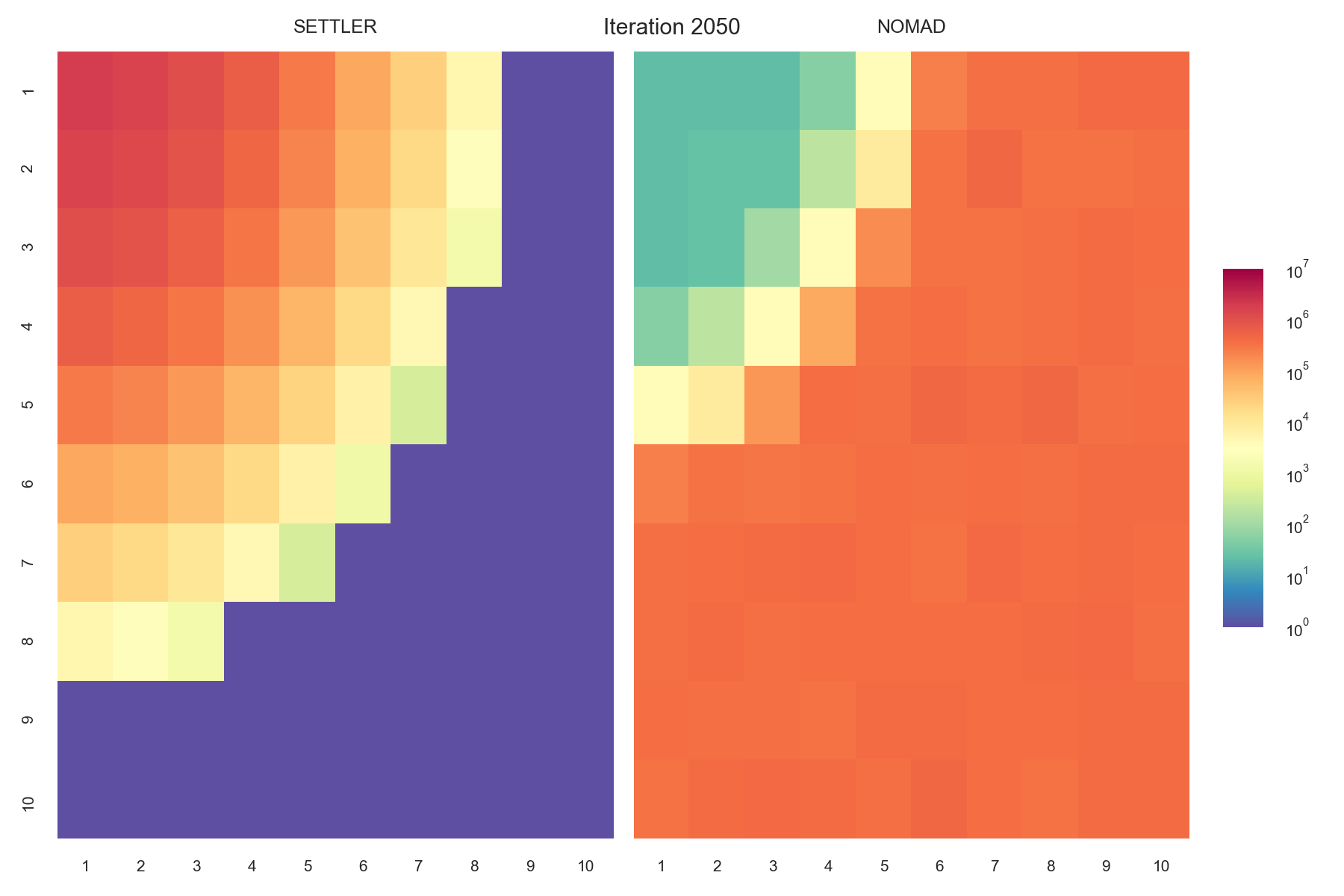

Supplement: Supplementary file 1 [file biology-10-01019-s001.zip › Spatio-temporal dynamics heatmaps/chempenoff_extremelyscarce_lindeath_period1000/2050.png]

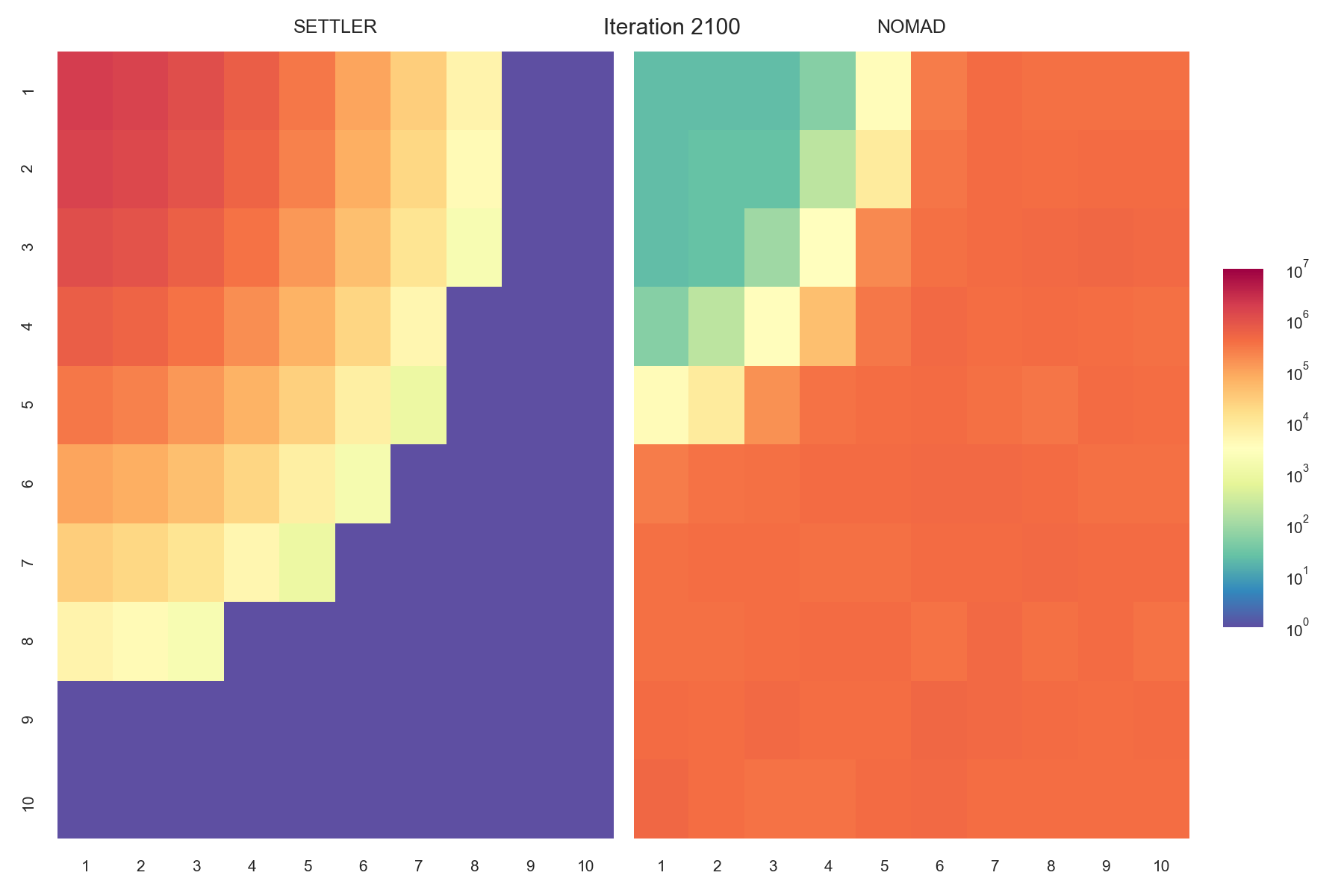

Supplement: Supplementary file 1 [file biology-10-01019-s001.zip › Spatio-temporal dynamics heatmaps/chempenoff_extremelyscarce_lindeath_period1000/2100.png]

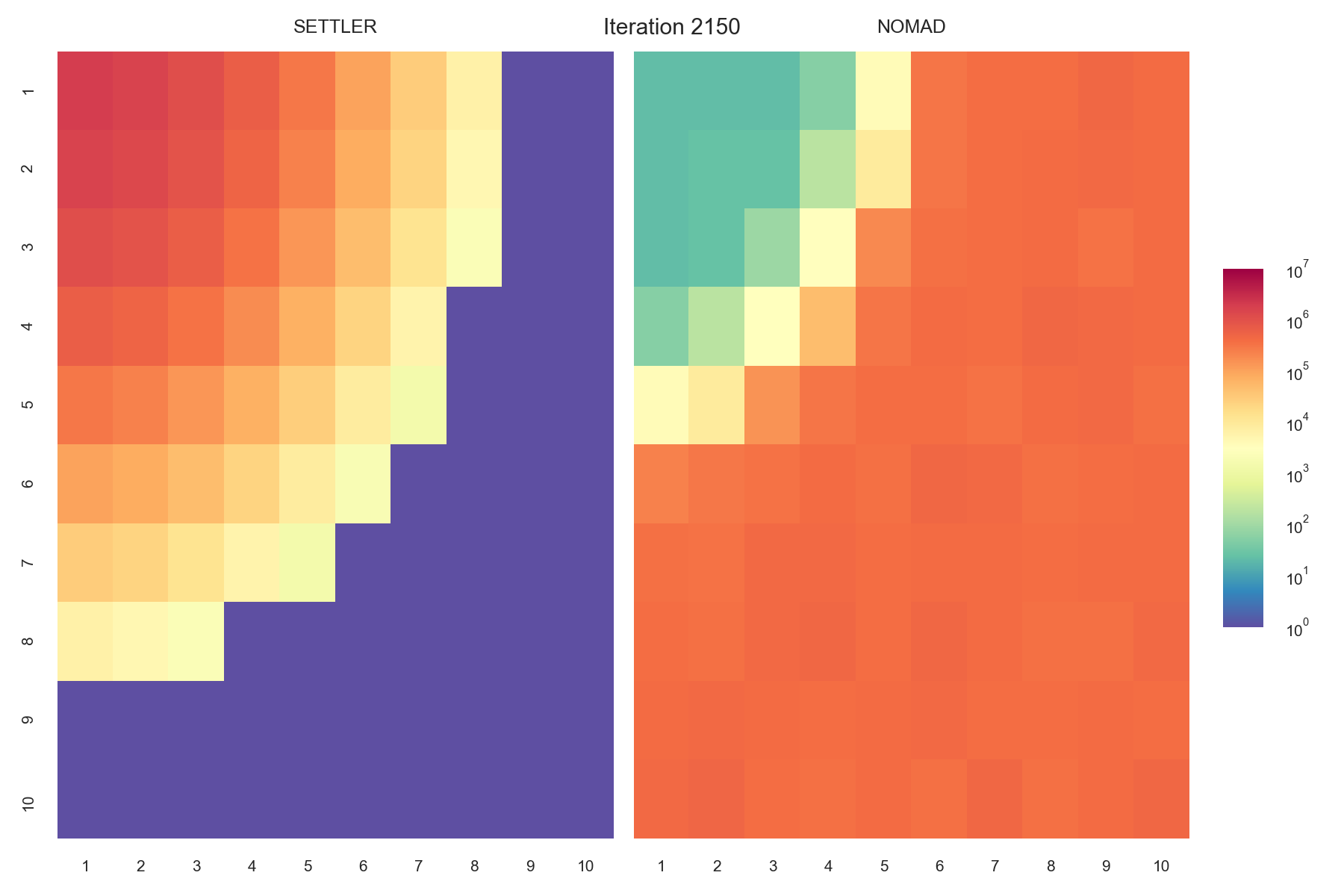

Supplement: Supplementary file 1 [file biology-10-01019-s001.zip › Spatio-temporal dynamics heatmaps/chempenoff_extremelyscarce_lindeath_period1000/2150.png]

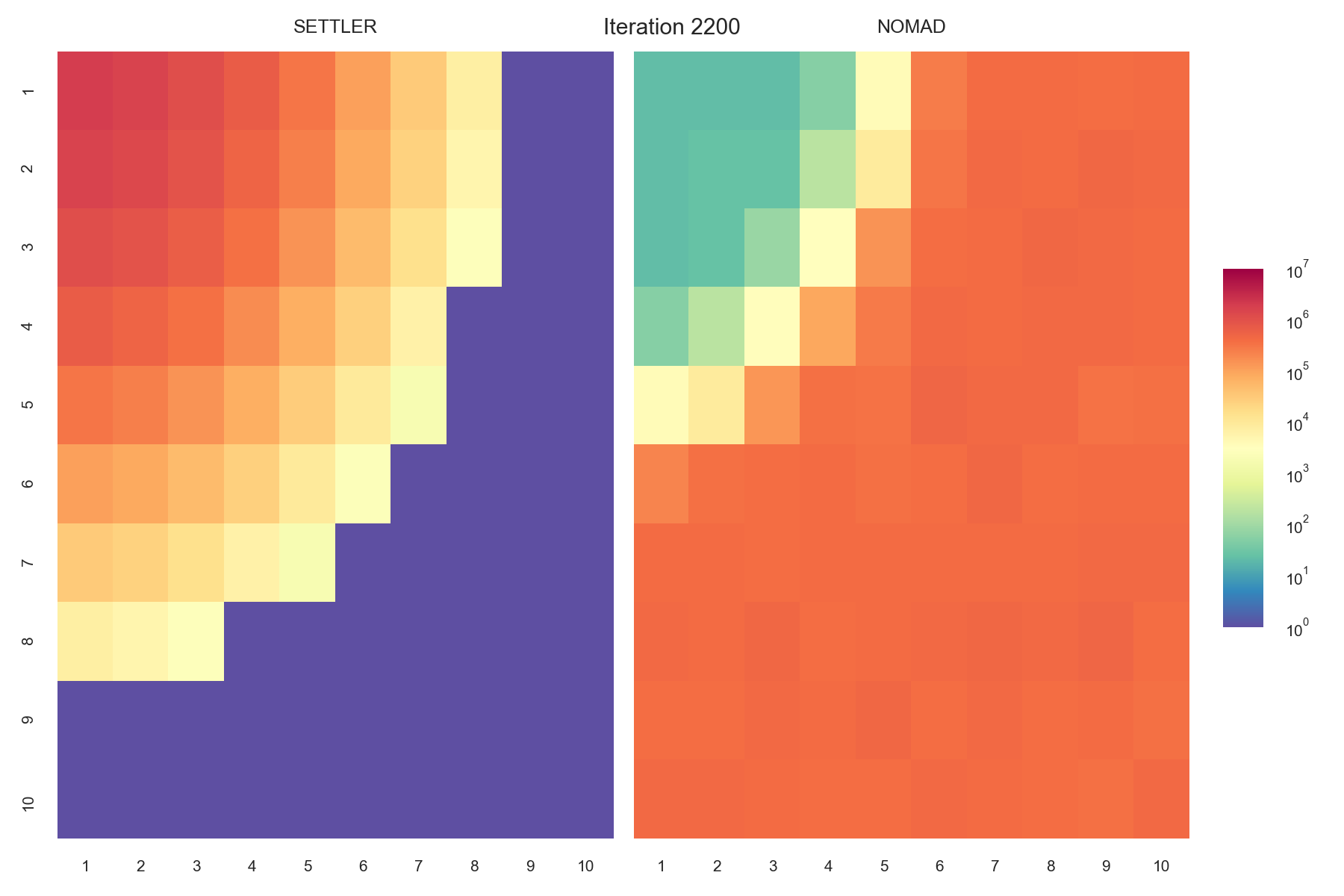

Supplement: Supplementary file 1 [file biology-10-01019-s001.zip › Spatio-temporal dynamics heatmaps/chempenoff_extremelyscarce_lindeath_period1000/2200.png]

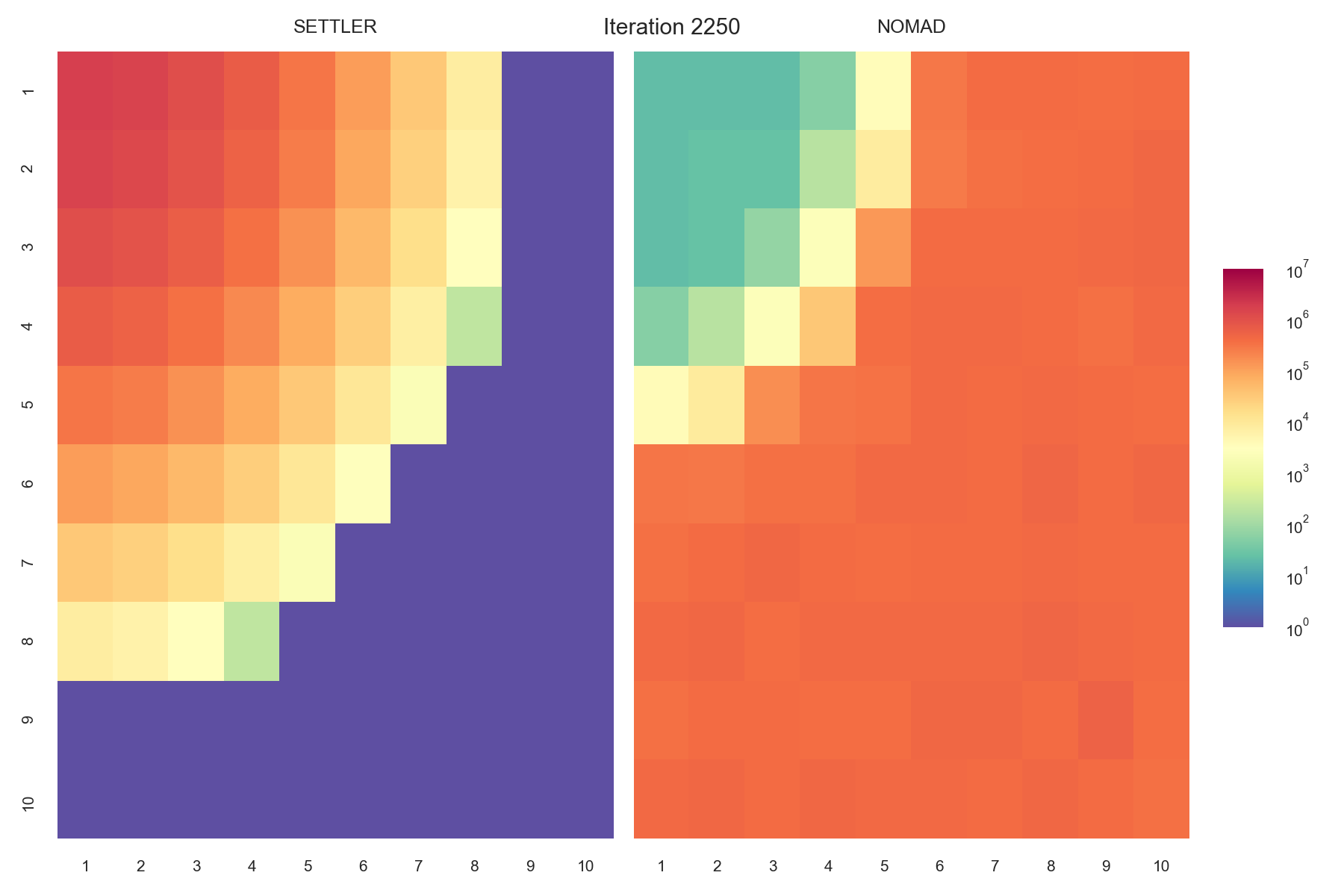

Supplement: Supplementary file 1 [file biology-10-01019-s001.zip › Spatio-temporal dynamics heatmaps/chempenoff_extremelyscarce_lindeath_period1000/2250.png]

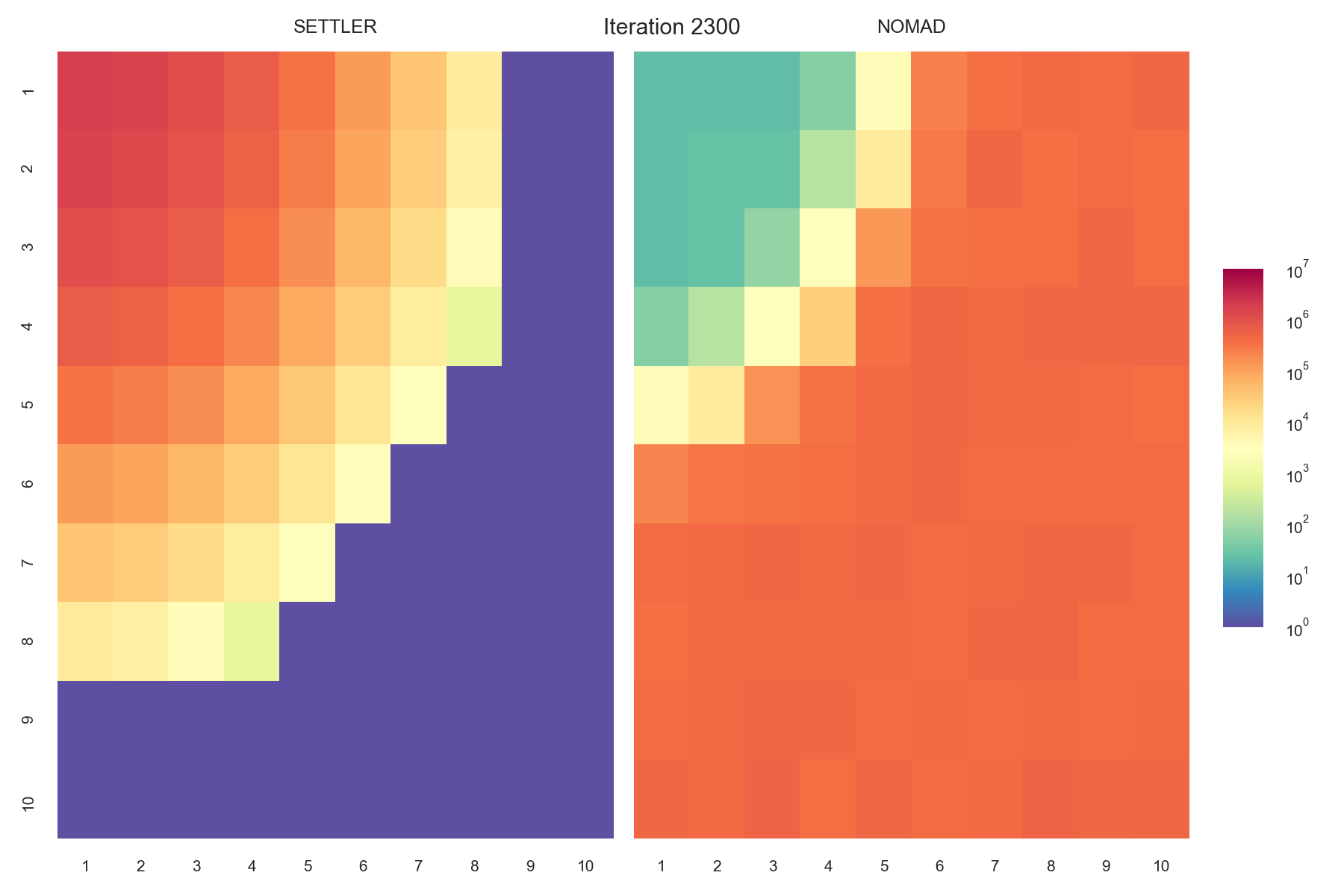

Supplement: Supplementary file 1 [file biology-10-01019-s001.zip › Spatio-temporal dynamics heatmaps/chempenoff_extremelyscarce_lindeath_period1000/2300.png]

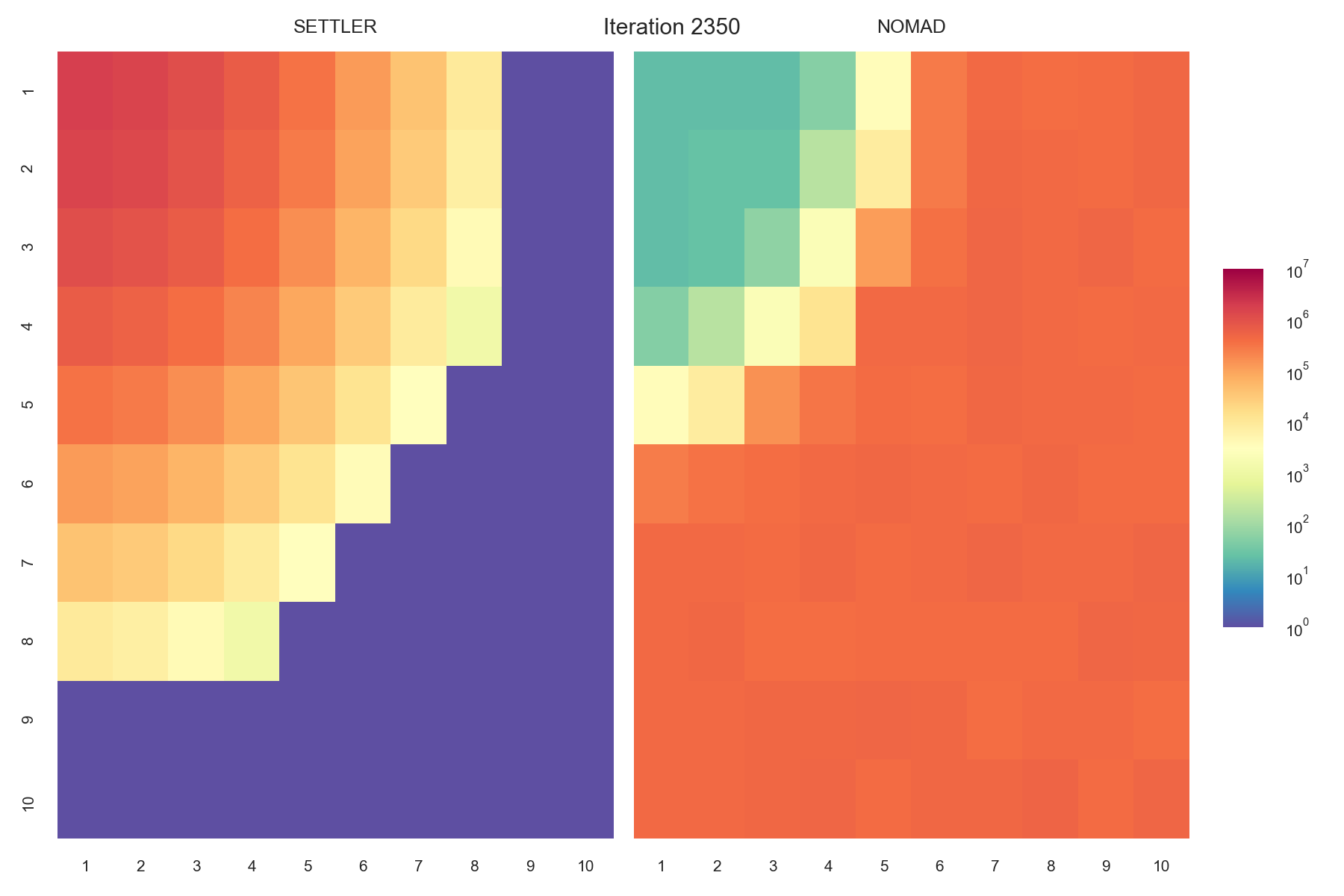

Supplement: Supplementary file 1 [file biology-10-01019-s001.zip › Spatio-temporal dynamics heatmaps/chempenoff_extremelyscarce_lindeath_period1000/2350.png]

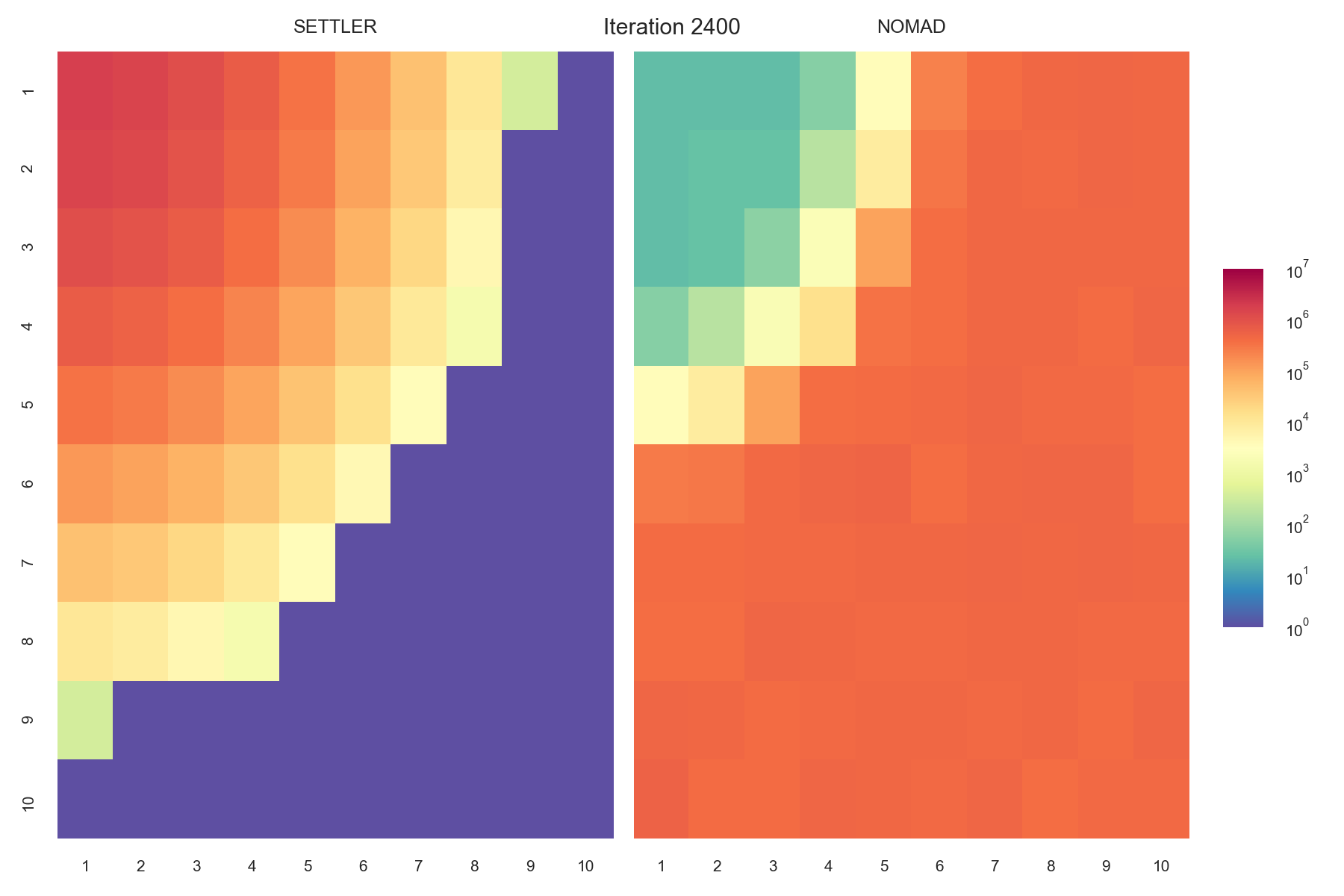

Supplement: Supplementary file 1 [file biology-10-01019-s001.zip › Spatio-temporal dynamics heatmaps/chempenoff_extremelyscarce_lindeath_period1000/2400.png]

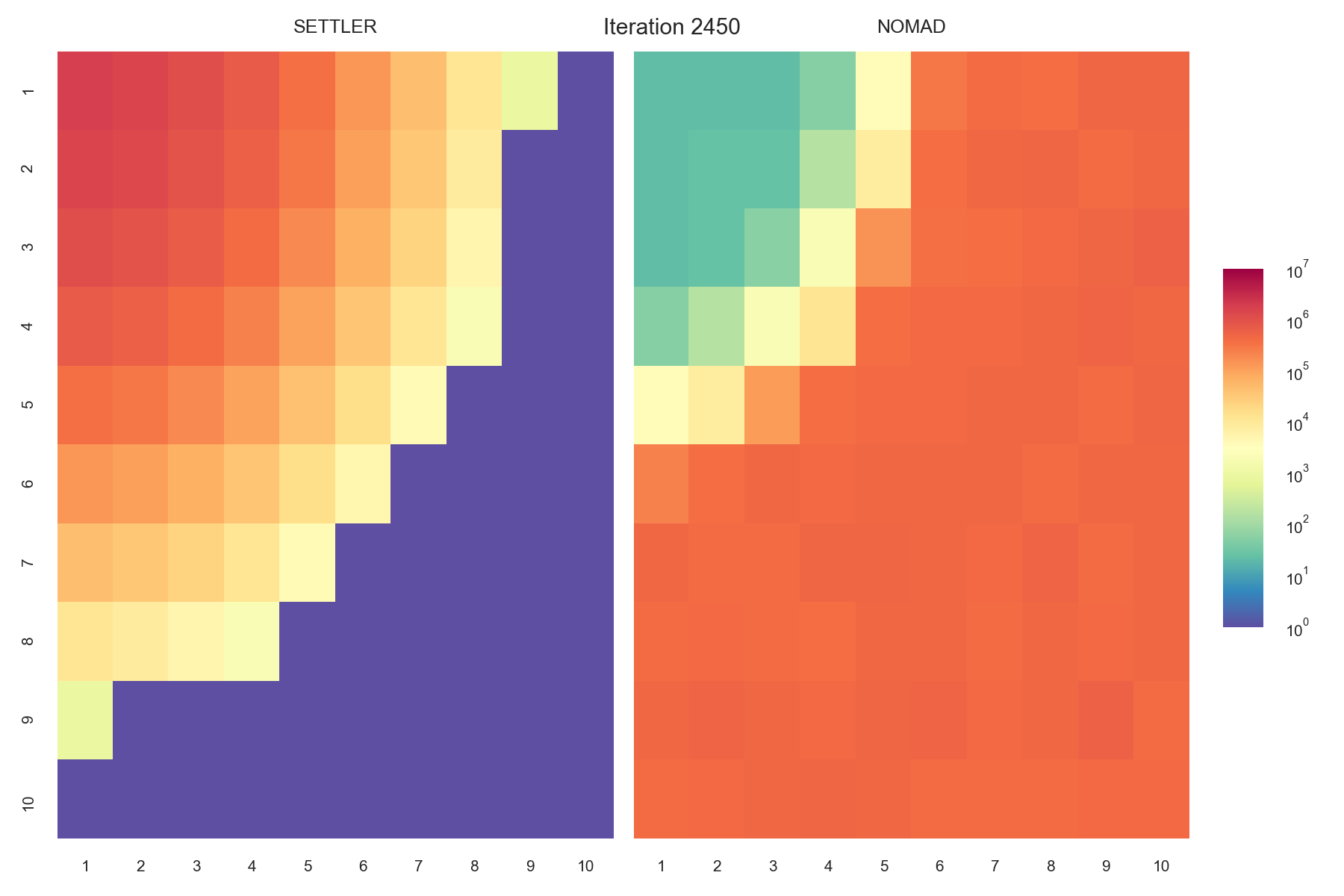

Supplement: Supplementary file 1 [file biology-10-01019-s001.zip › Spatio-temporal dynamics heatmaps/chempenoff_extremelyscarce_lindeath_period1000/2450.png]

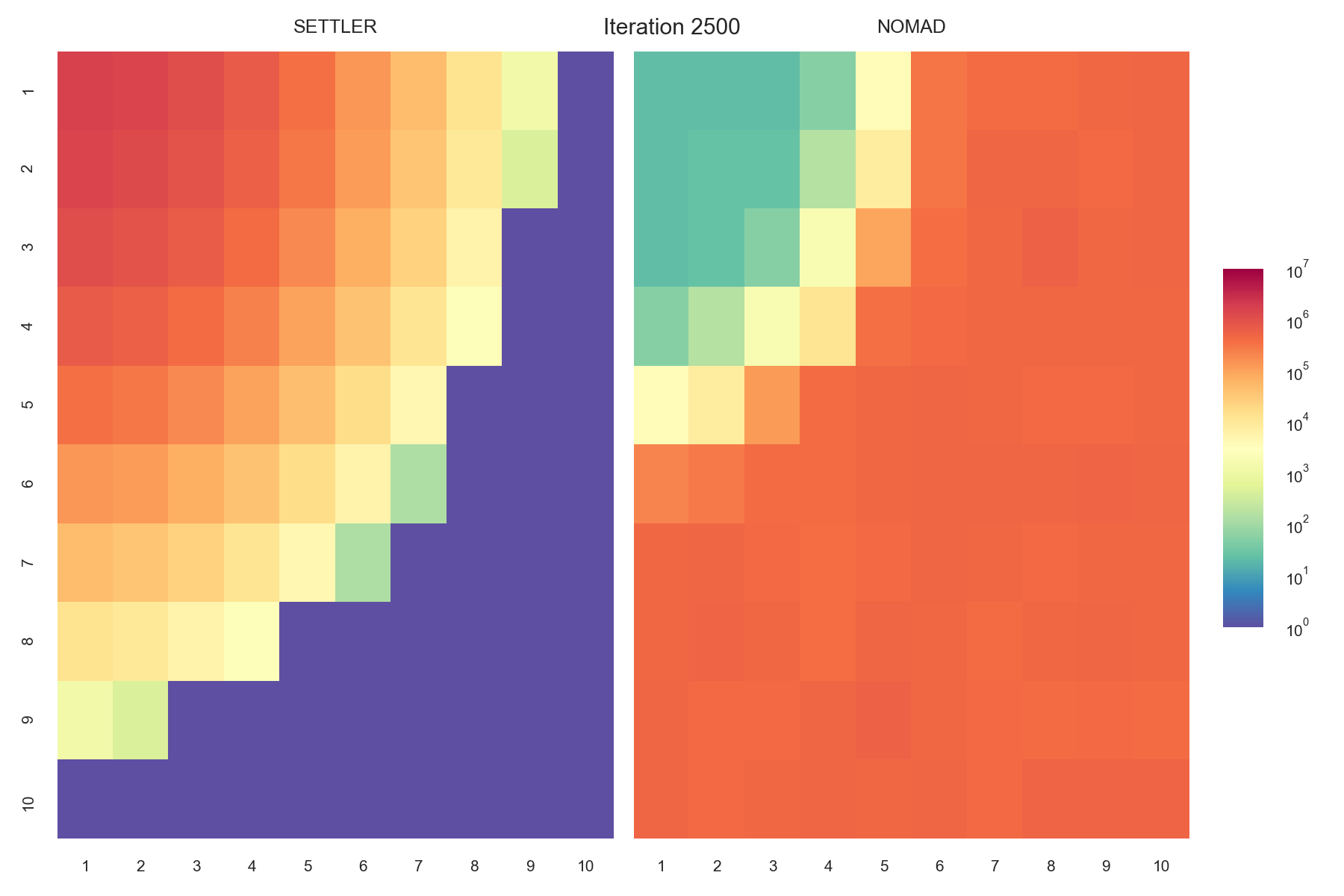

Supplement: Supplementary file 1 [file biology-10-01019-s001.zip › Spatio-temporal dynamics heatmaps/chempenoff_extremelyscarce_lindeath_period1000/2500.png]

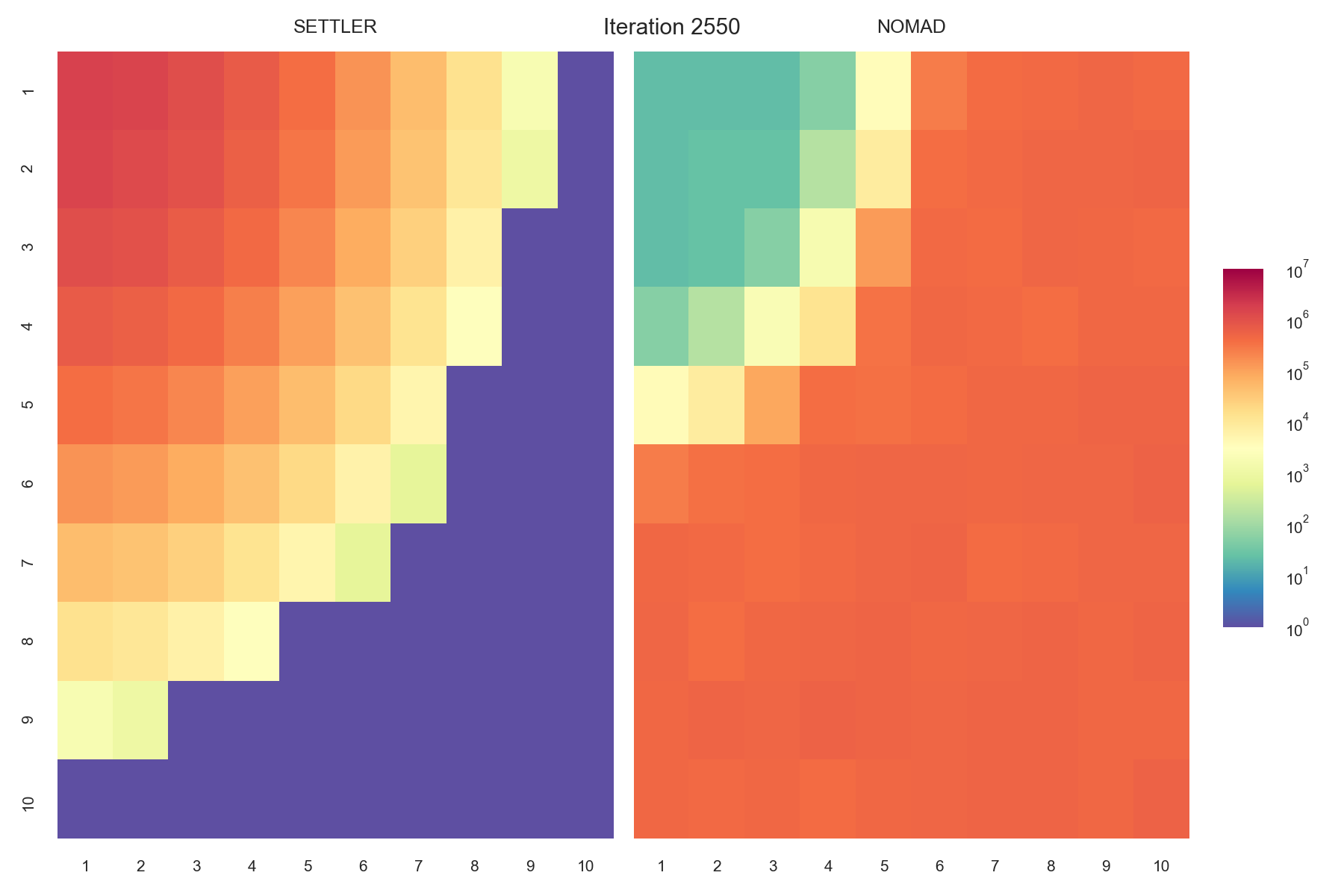

Supplement: Supplementary file 1 [file biology-10-01019-s001.zip › Spatio-temporal dynamics heatmaps/chempenoff_extremelyscarce_lindeath_period1000/2550.png]

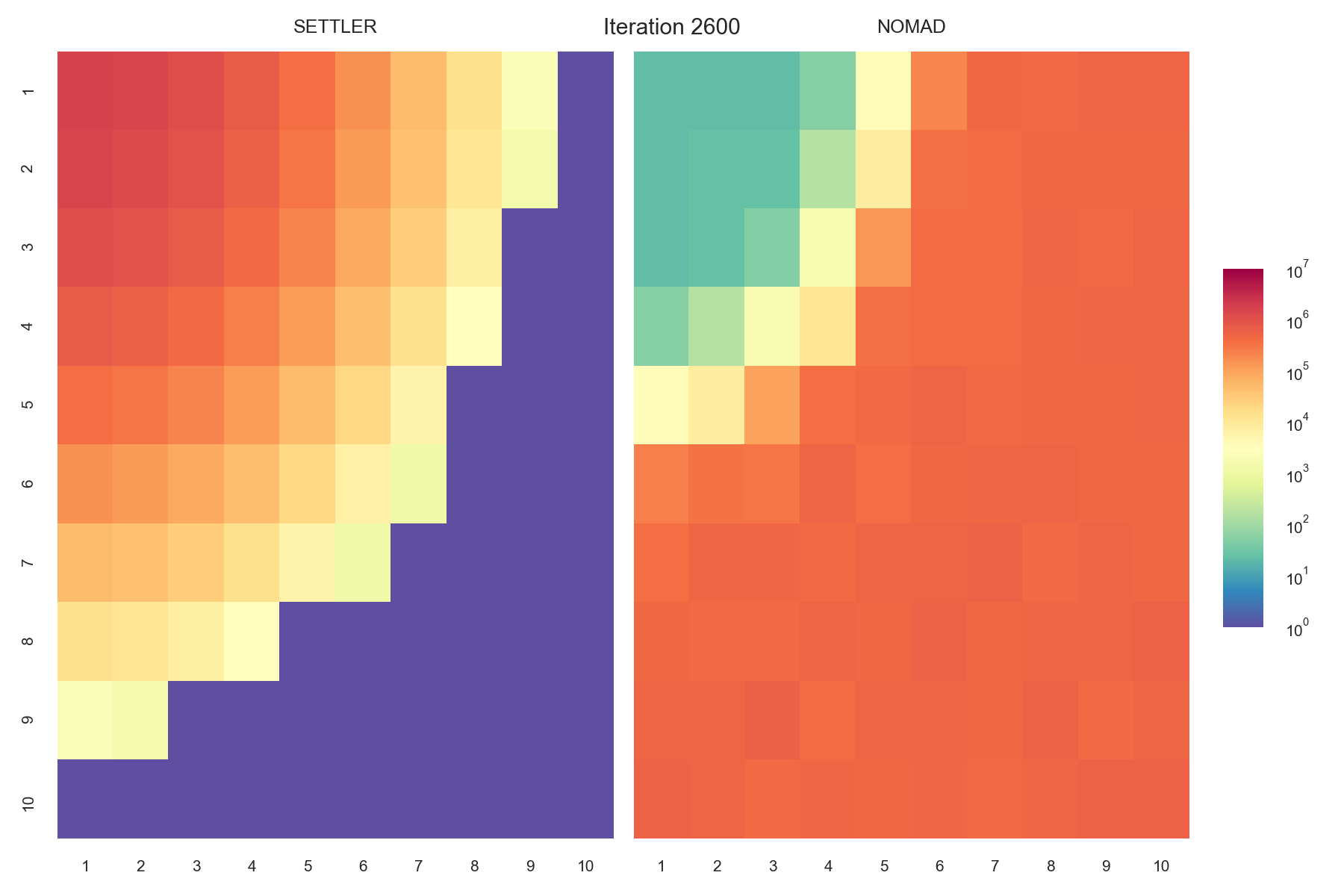

Supplement: Supplementary file 1 [file biology-10-01019-s001.zip › Spatio-temporal dynamics heatmaps/chempenoff_extremelyscarce_lindeath_period1000/2600.png]

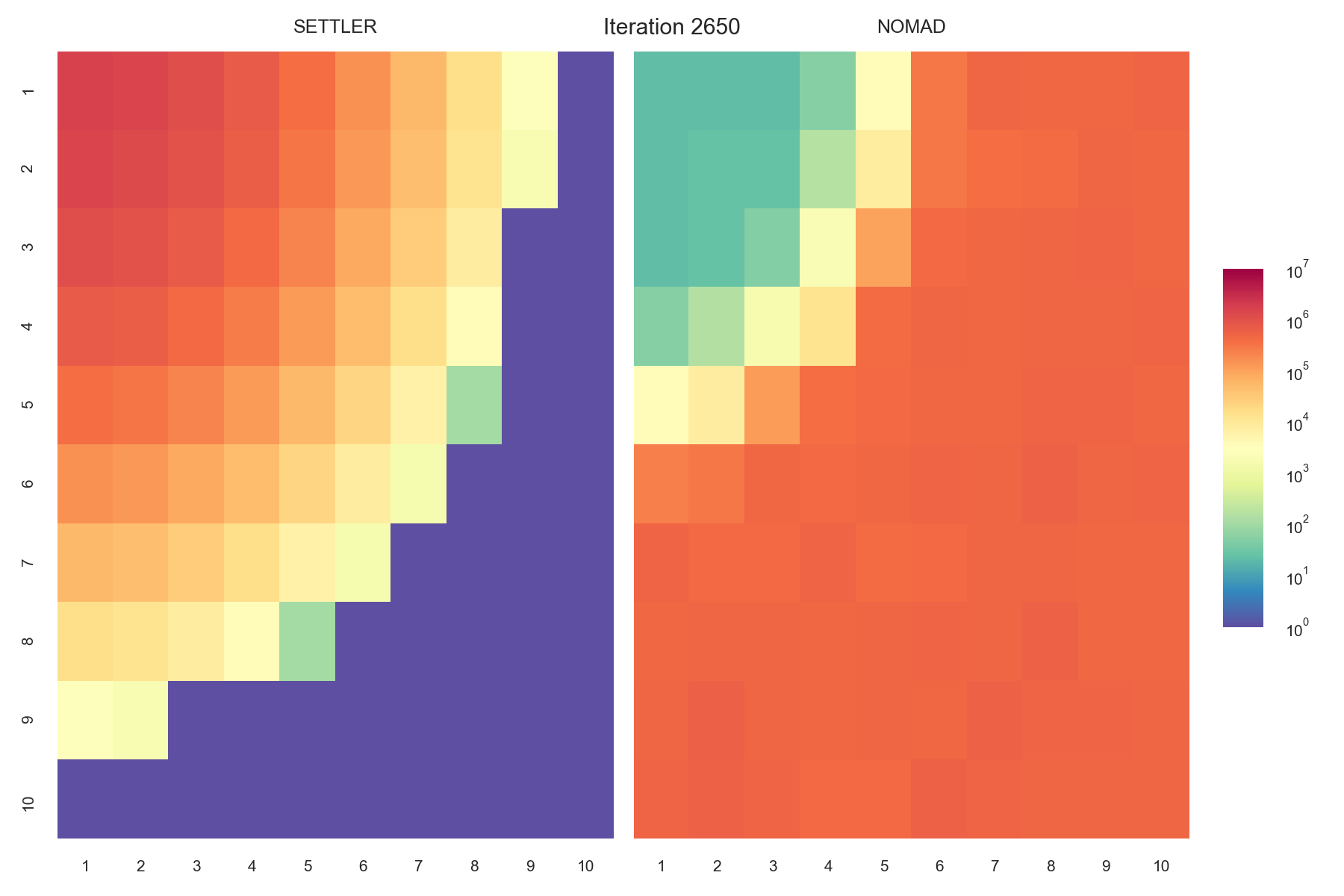

Supplement: Supplementary file 1 [file biology-10-01019-s001.zip › Spatio-temporal dynamics heatmaps/chempenoff_extremelyscarce_lindeath_period1000/2650.png]

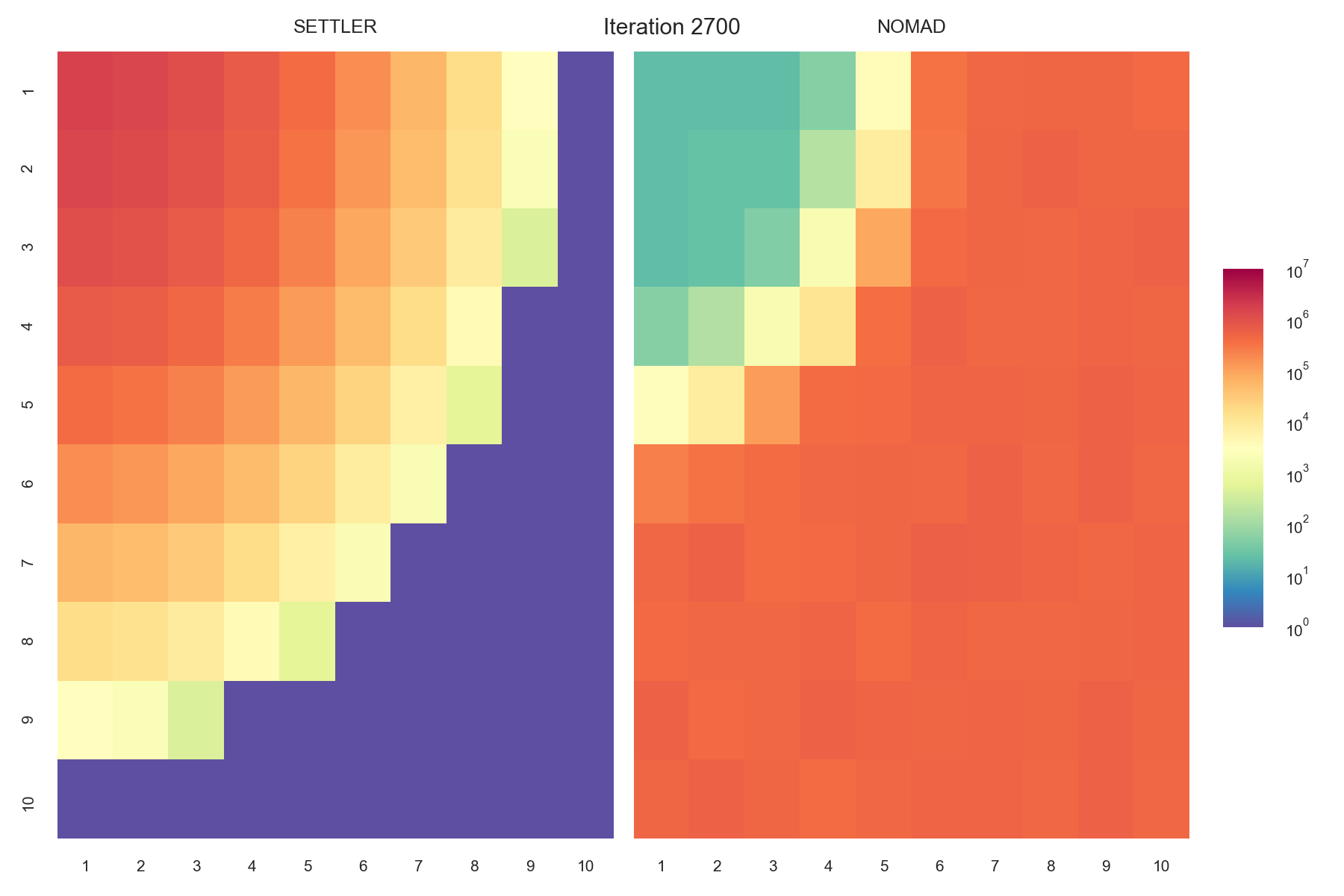

Supplement: Supplementary file 1 [file biology-10-01019-s001.zip › Spatio-temporal dynamics heatmaps/chempenoff_extremelyscarce_lindeath_period1000/2700.png]

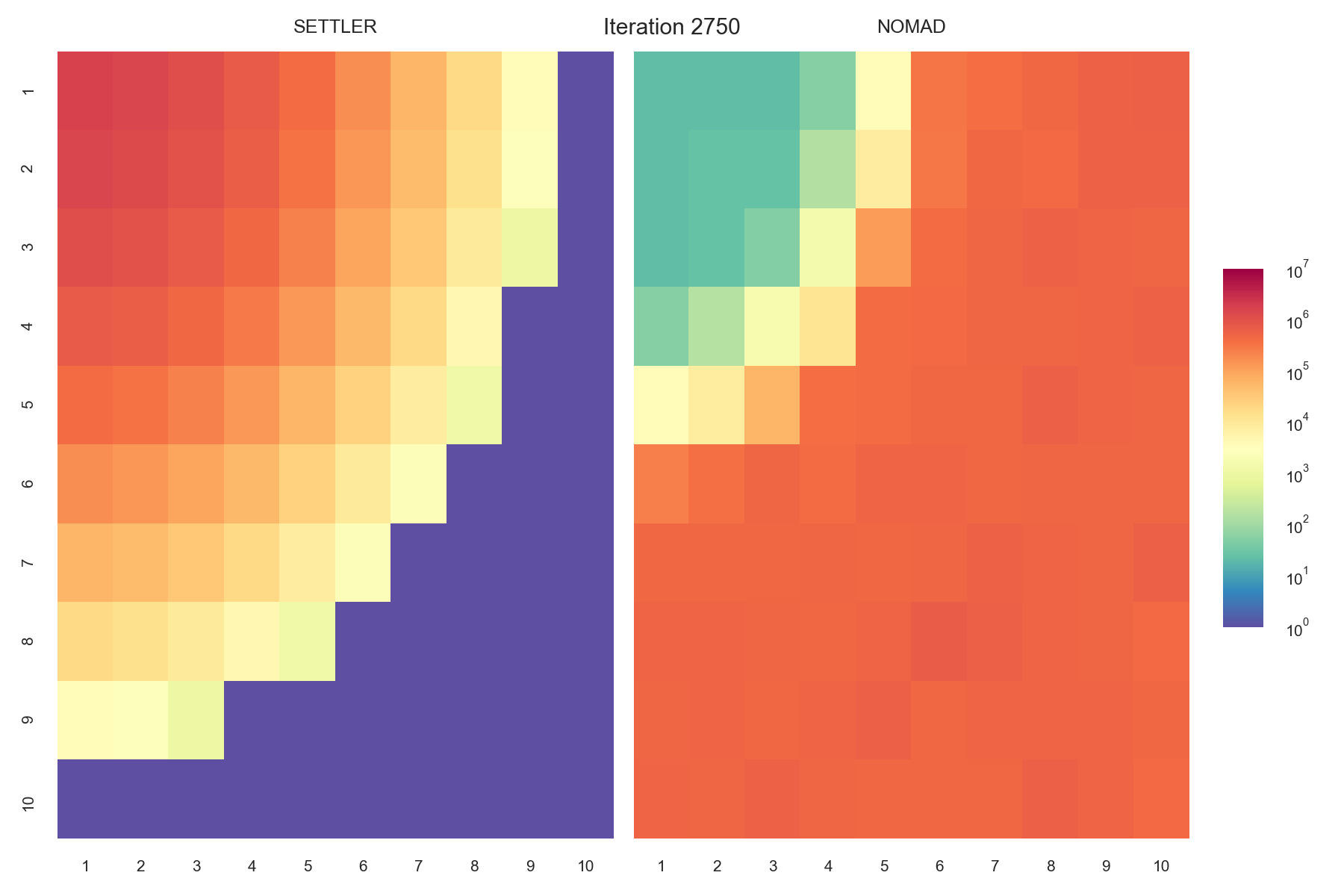

Supplement: Supplementary file 1 [file biology-10-01019-s001.zip › Spatio-temporal dynamics heatmaps/chempenoff_extremelyscarce_lindeath_period1000/2750.png]

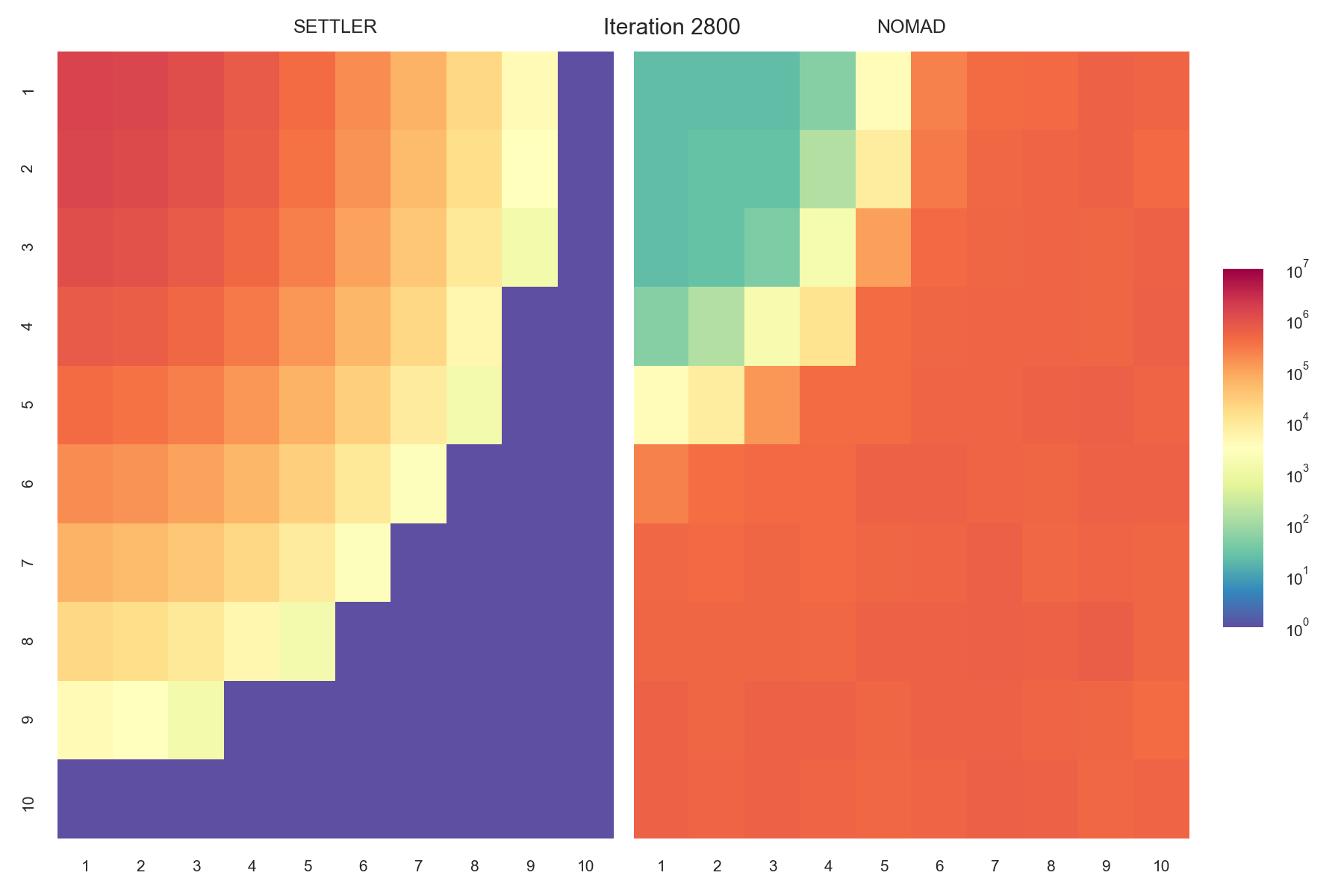

Supplement: Supplementary file 1 [file biology-10-01019-s001.zip › Spatio-temporal dynamics heatmaps/chempenoff_extremelyscarce_lindeath_period1000/2800.png]

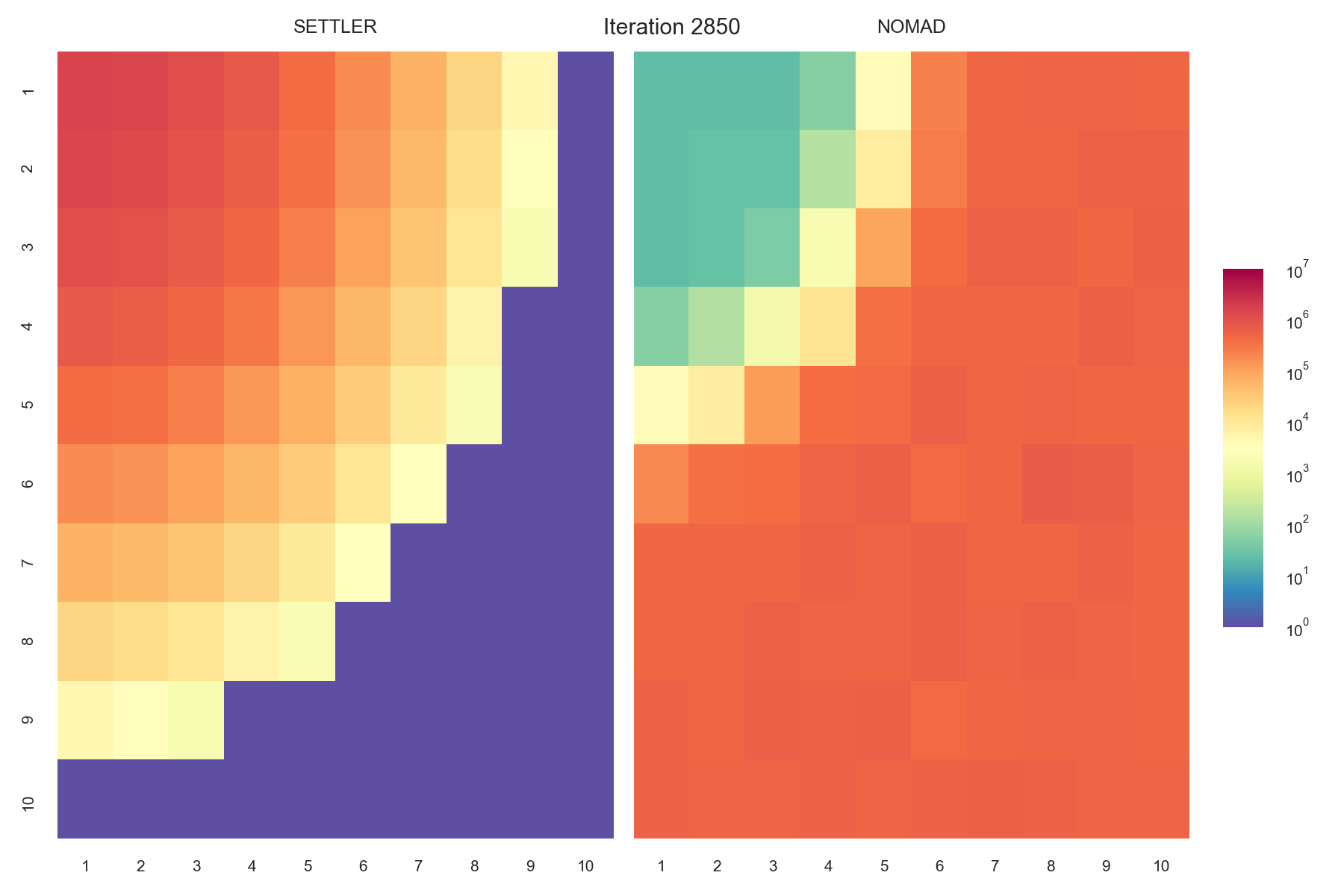

Supplement: Supplementary file 1 [file biology-10-01019-s001.zip › Spatio-temporal dynamics heatmaps/chempenoff_extremelyscarce_lindeath_period1000/2850.png]

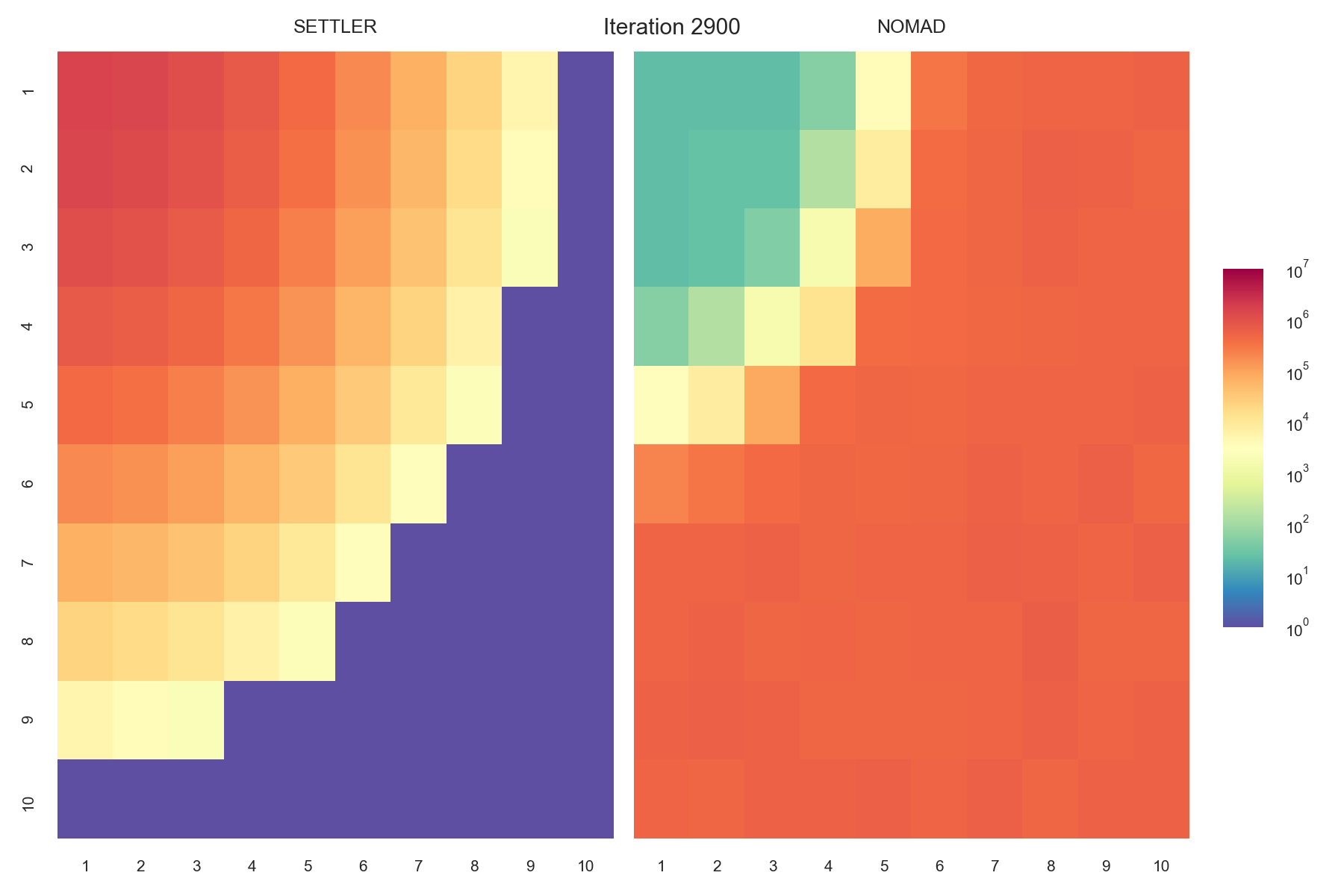

Supplement: Supplementary file 1 [file biology-10-01019-s001.zip › Spatio-temporal dynamics heatmaps/chempenoff_extremelyscarce_lindeath_period1000/2900.png]

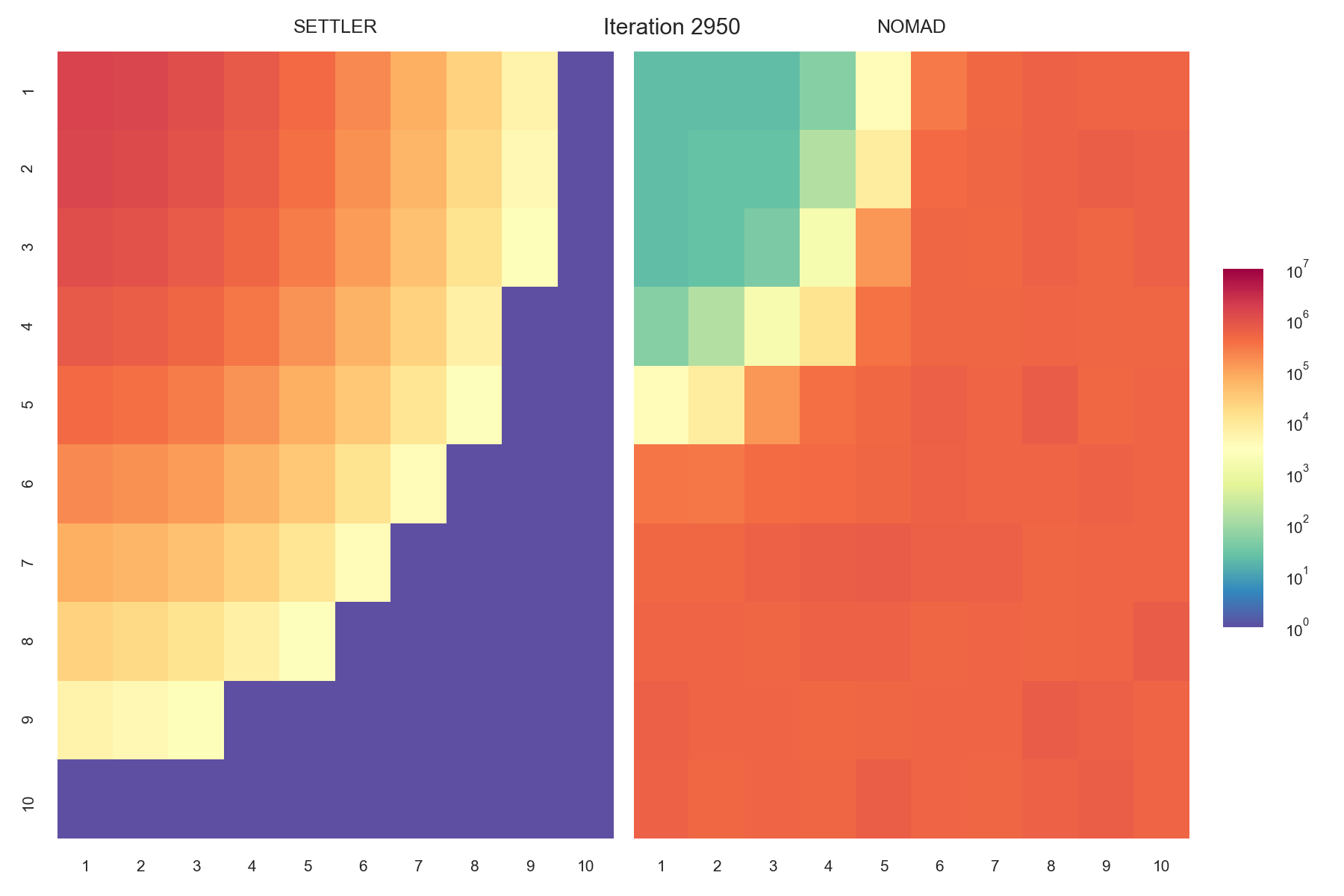

Supplement: Supplementary file 1 [file biology-10-01019-s001.zip › Spatio-temporal dynamics heatmaps/chempenoff_extremelyscarce_lindeath_period1000/2950.png]

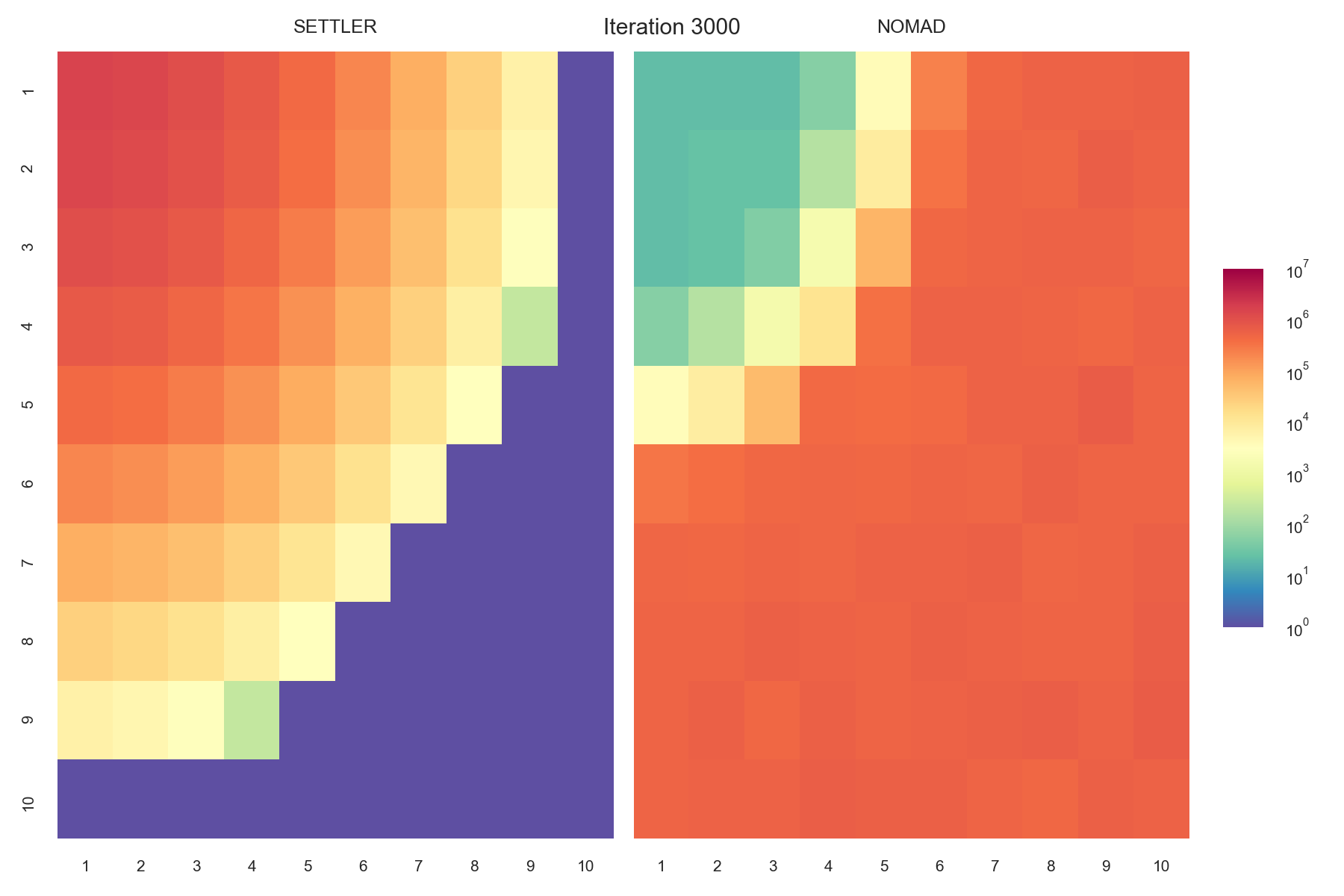

Supplement: Supplementary file 1 [file biology-10-01019-s001.zip › Spatio-temporal dynamics heatmaps/chempenoff_extremelyscarce_lindeath_period1000/3000.png]

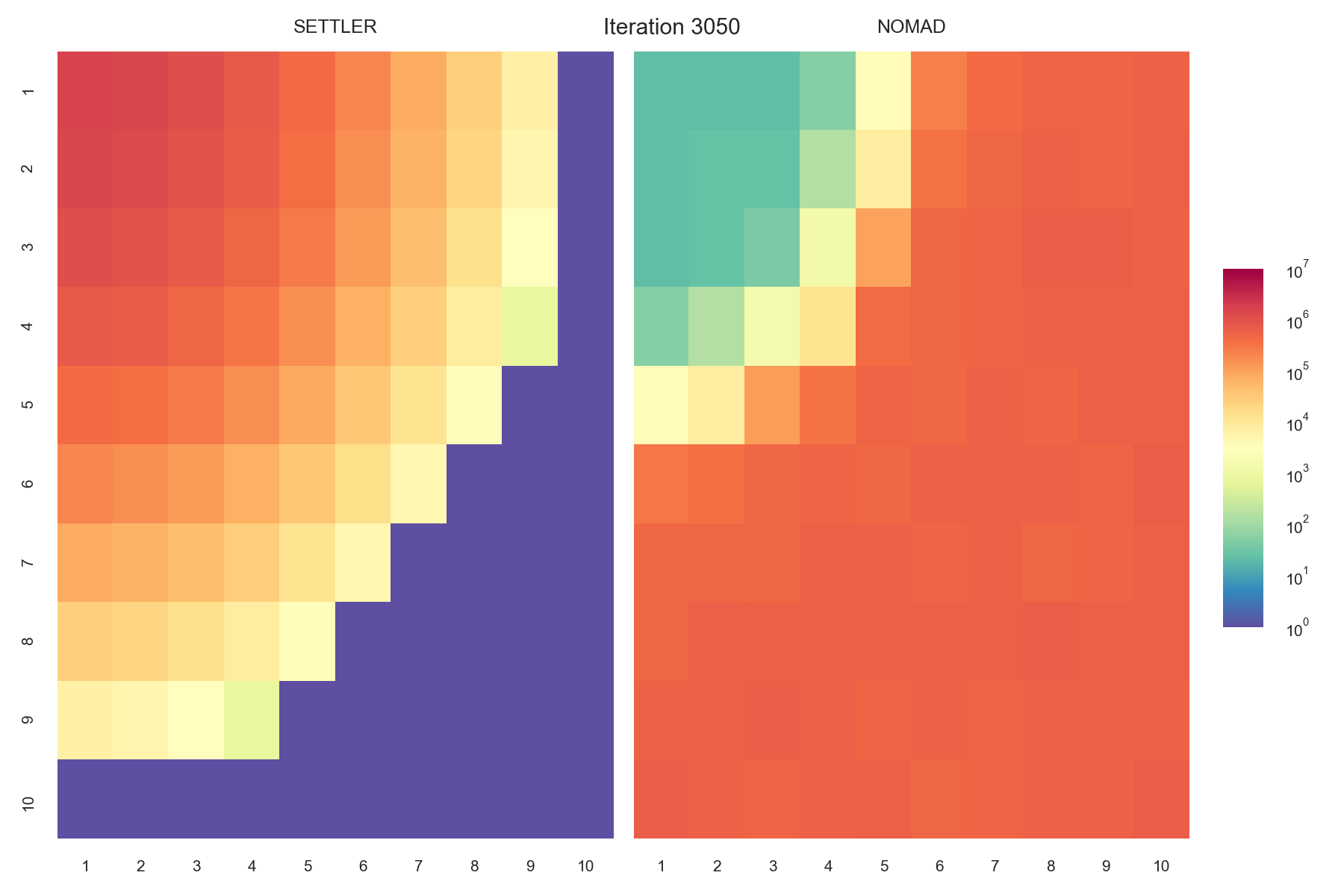

Supplement: Supplementary file 1 [file biology-10-01019-s001.zip › Spatio-temporal dynamics heatmaps/chempenoff_extremelyscarce_lindeath_period1000/3050.png]

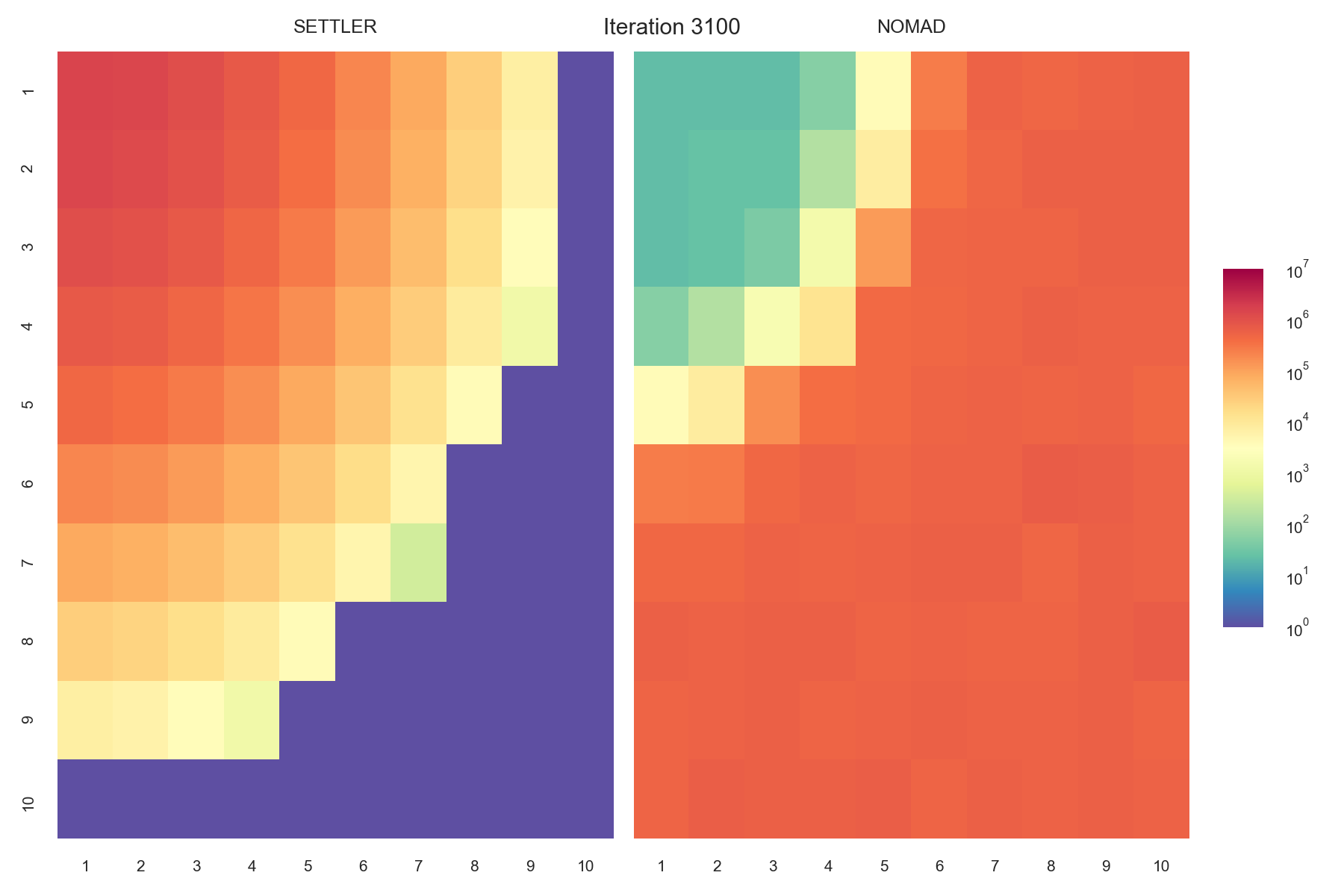

Supplement: Supplementary file 1 [file biology-10-01019-s001.zip › Spatio-temporal dynamics heatmaps/chempenoff_extremelyscarce_lindeath_period1000/3100.png]

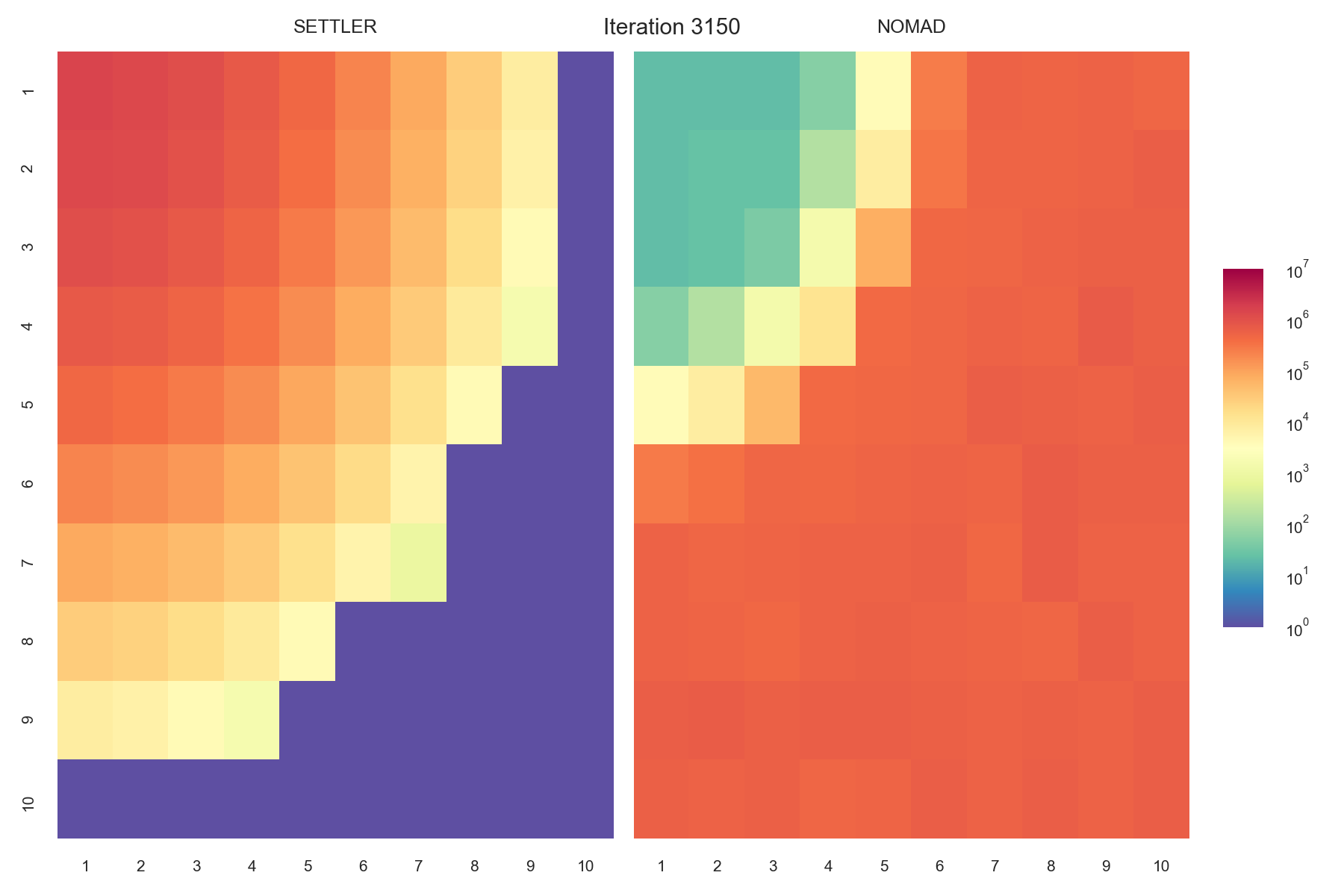

Supplement: Supplementary file 1 [file biology-10-01019-s001.zip › Spatio-temporal dynamics heatmaps/chempenoff_extremelyscarce_lindeath_period1000/3150.png]

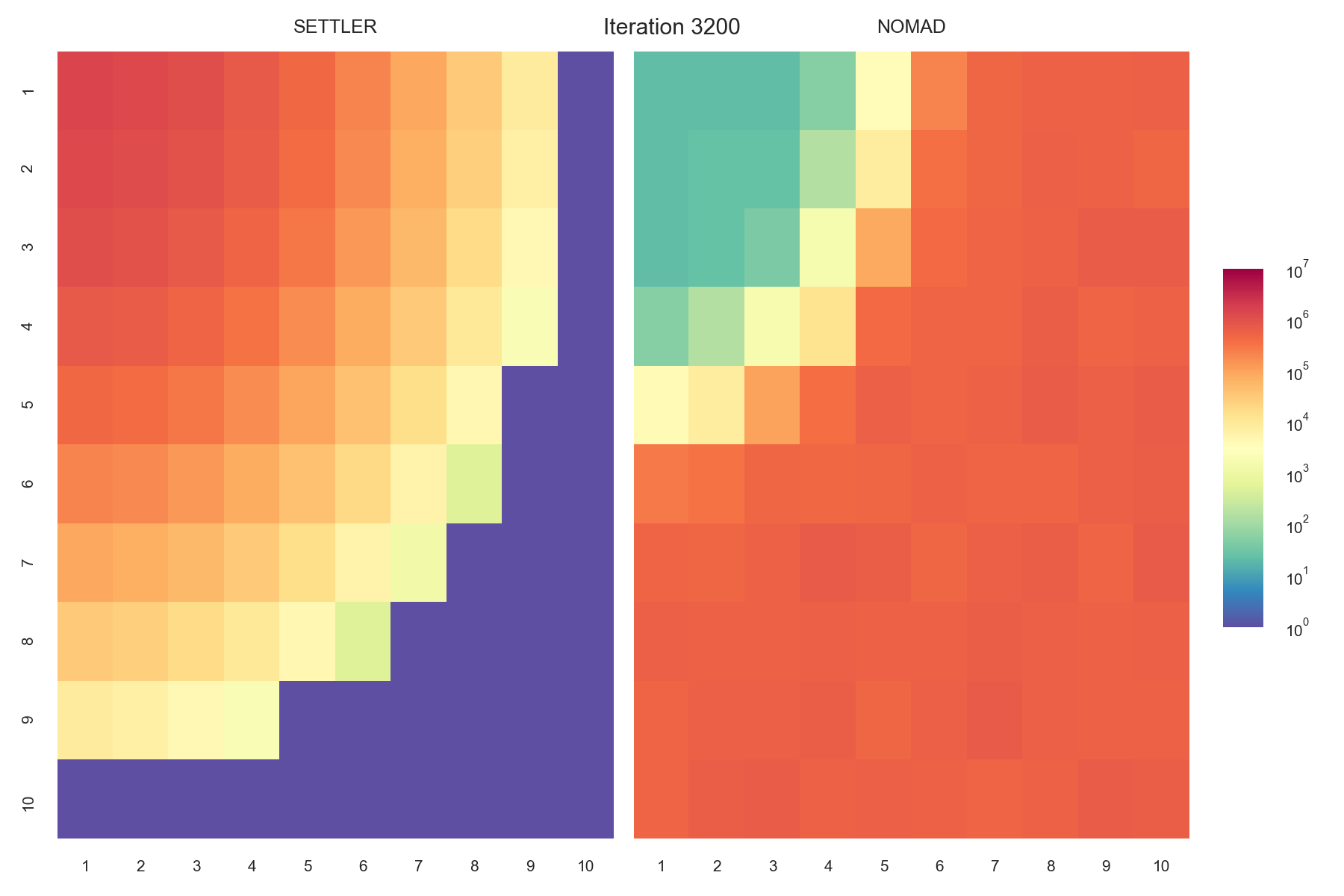

Supplement: Supplementary file 1 [file biology-10-01019-s001.zip › Spatio-temporal dynamics heatmaps/chempenoff_extremelyscarce_lindeath_period1000/3200.png]

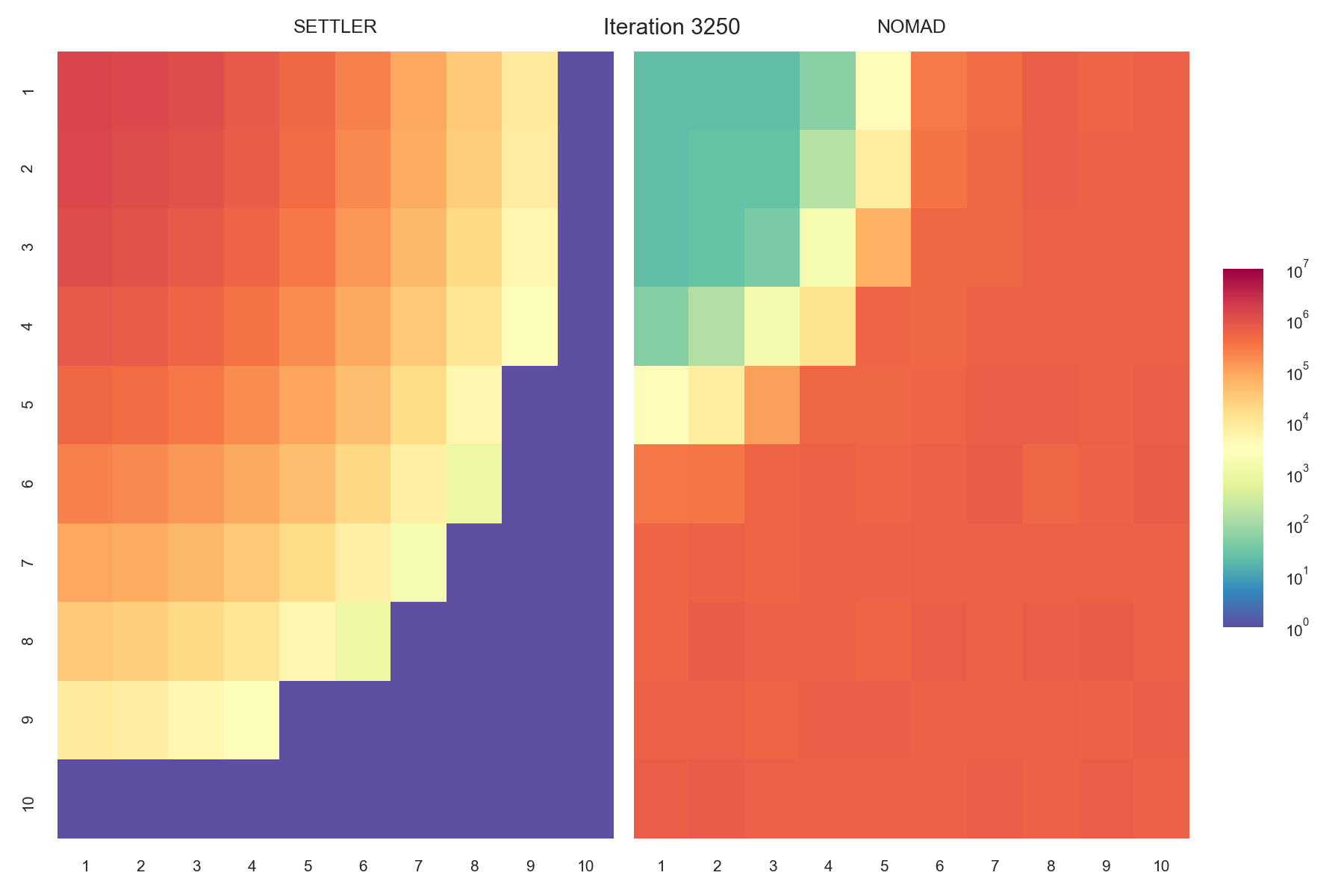

Supplement: Supplementary file 1 [file biology-10-01019-s001.zip › Spatio-temporal dynamics heatmaps/chempenoff_extremelyscarce_lindeath_period1000/3250.png]

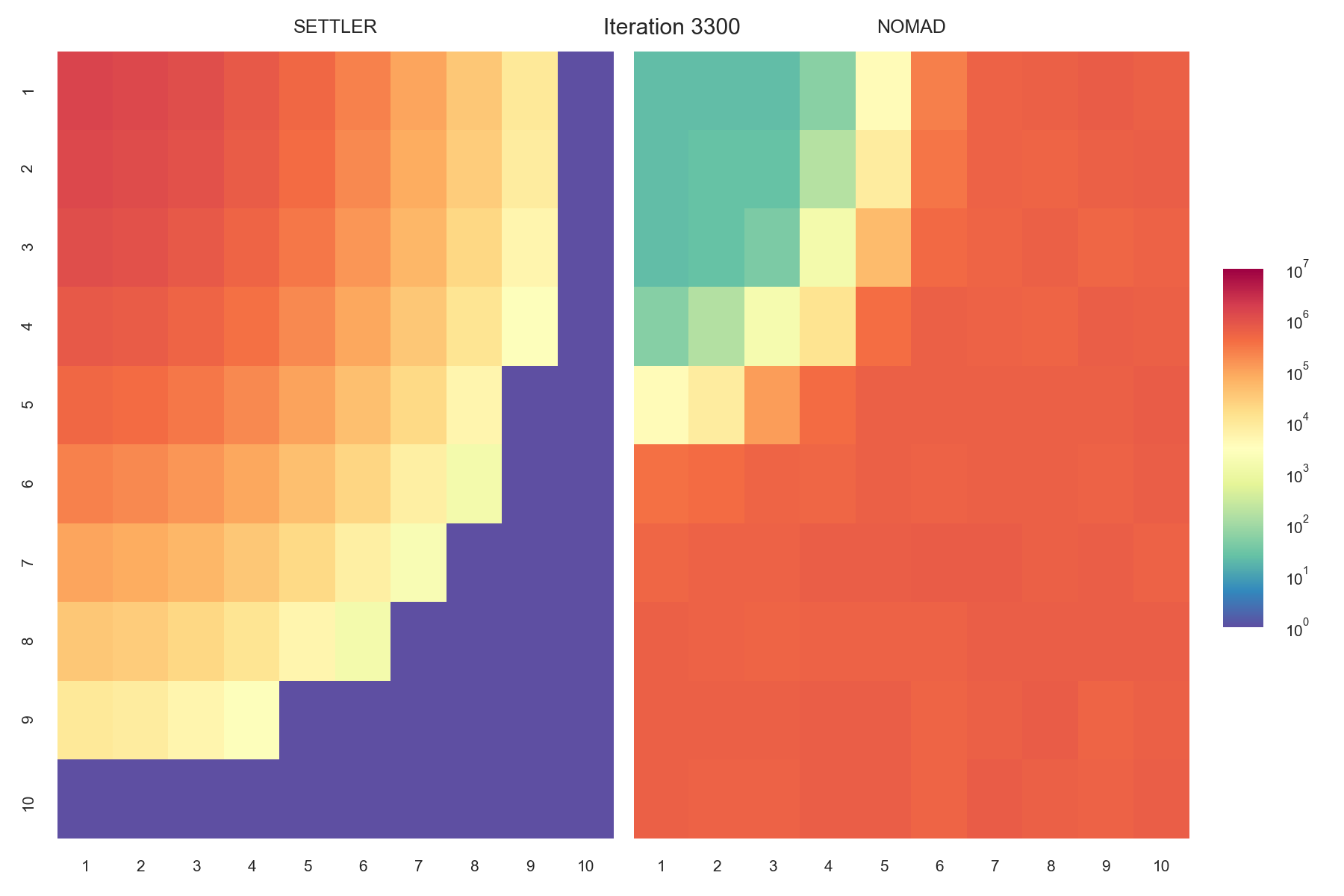

Supplement: Supplementary file 1 [file biology-10-01019-s001.zip › Spatio-temporal dynamics heatmaps/chempenoff_extremelyscarce_lindeath_period1000/3300.png]

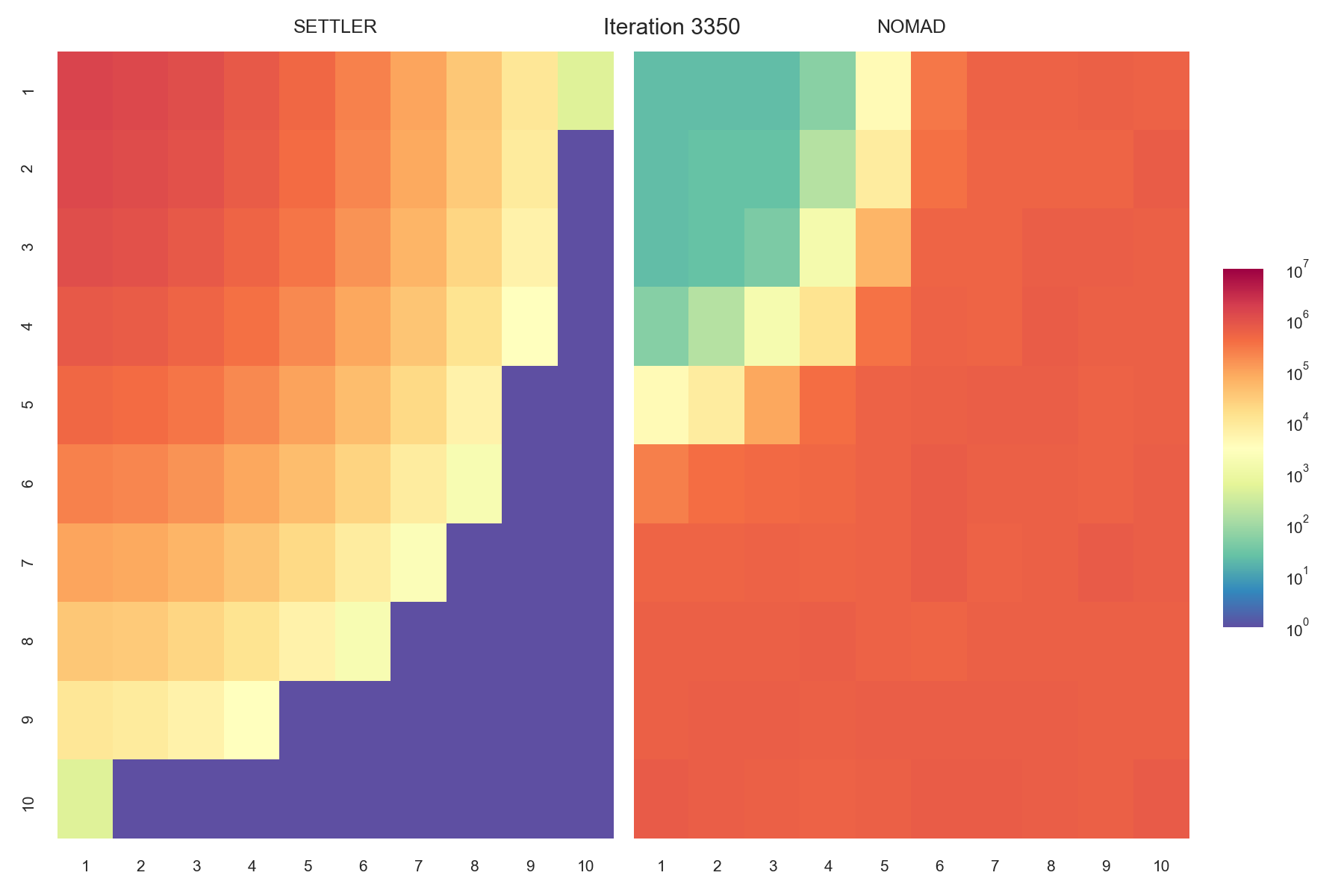

Supplement: Supplementary file 1 [file biology-10-01019-s001.zip › Spatio-temporal dynamics heatmaps/chempenoff_extremelyscarce_lindeath_period1000/3350.png]

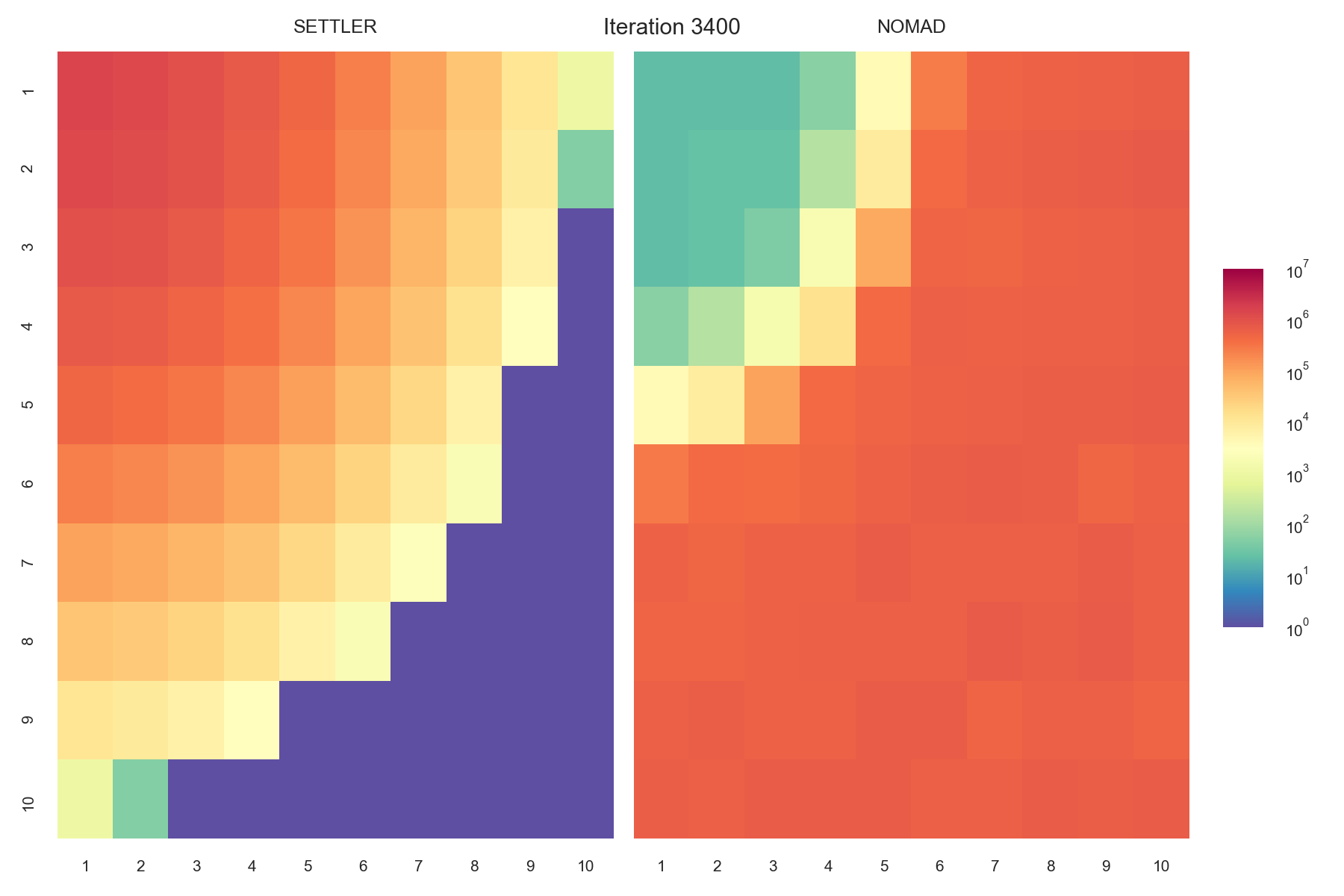

Supplement: Supplementary file 1 [file biology-10-01019-s001.zip › Spatio-temporal dynamics heatmaps/chempenoff_extremelyscarce_lindeath_period1000/3400.png]

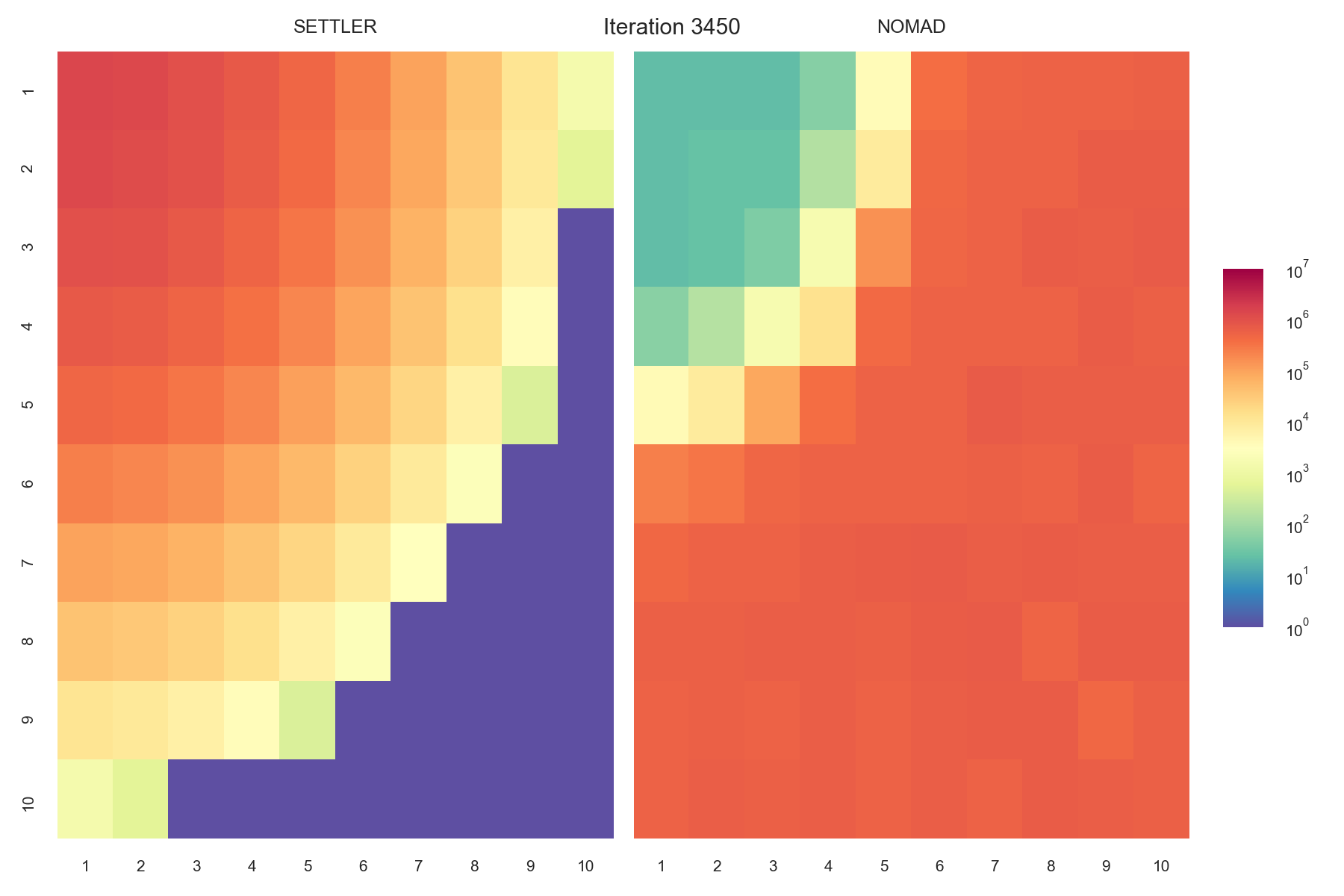

Supplement: Supplementary file 1 [file biology-10-01019-s001.zip › Spatio-temporal dynamics heatmaps/chempenoff_extremelyscarce_lindeath_period1000/3450.png]

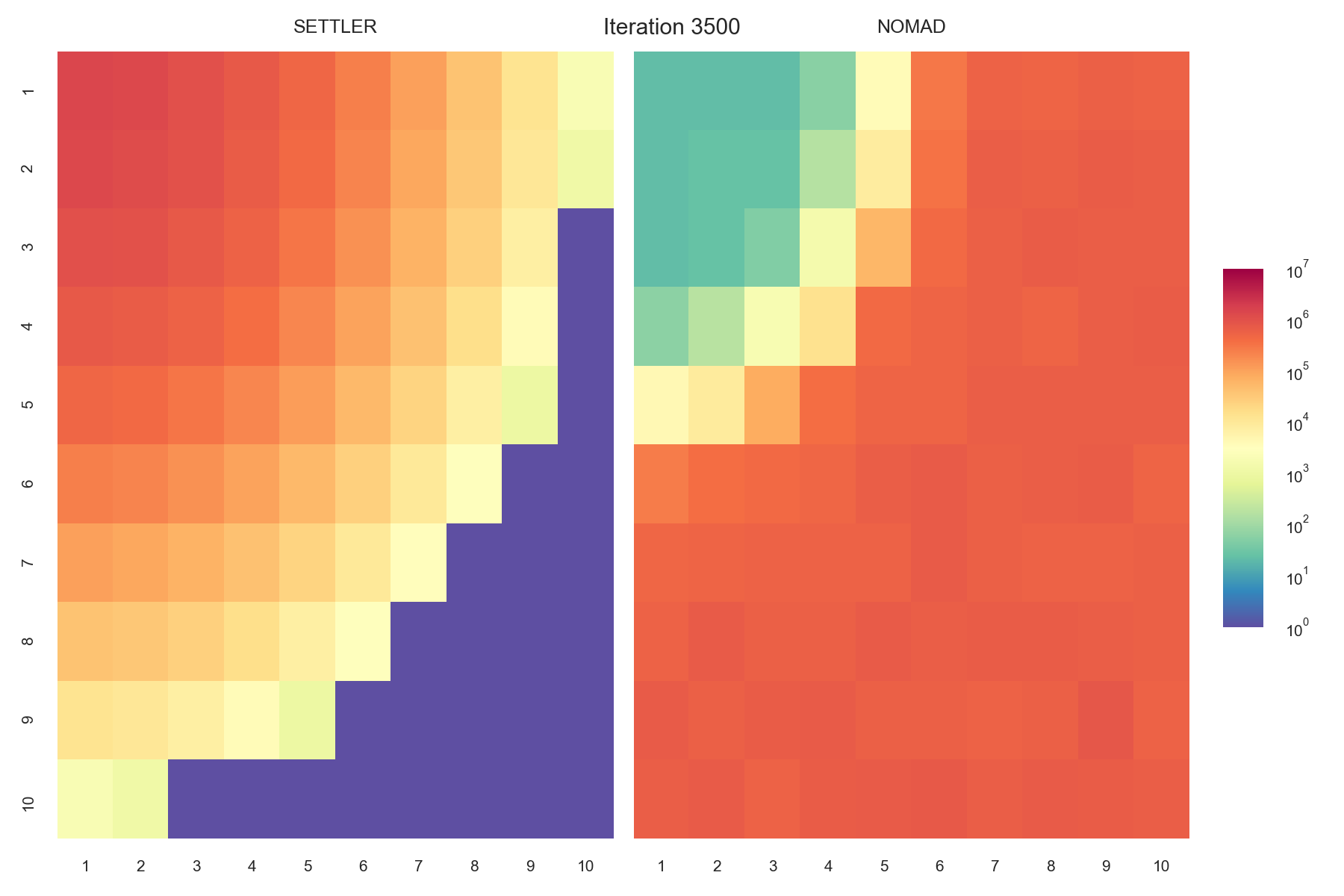

Supplement: Supplementary file 1 [file biology-10-01019-s001.zip › Spatio-temporal dynamics heatmaps/chempenoff_extremelyscarce_lindeath_period1000/3500.png]

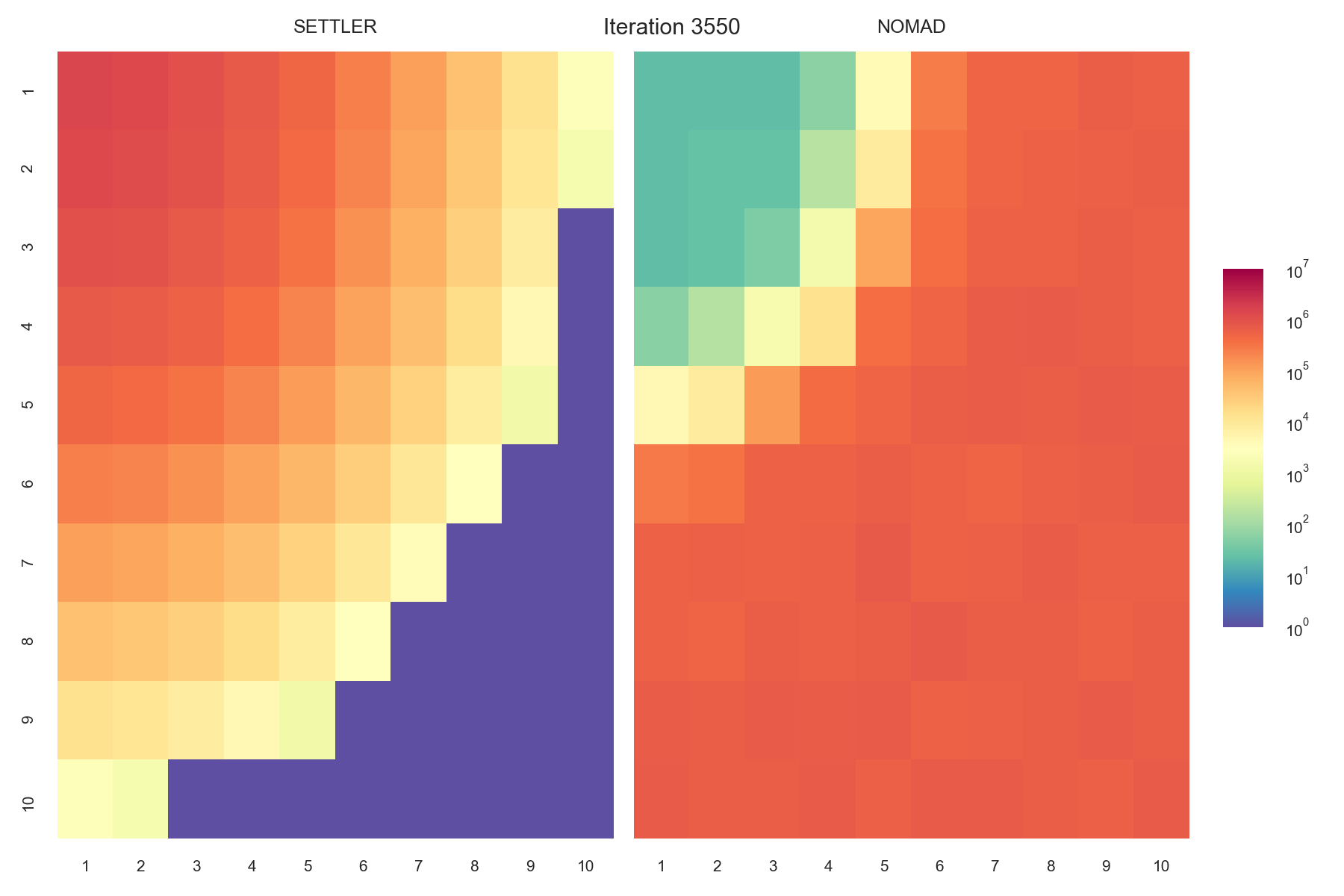

Supplement: Supplementary file 1 [file biology-10-01019-s001.zip › Spatio-temporal dynamics heatmaps/chempenoff_extremelyscarce_lindeath_period1000/3550.png]

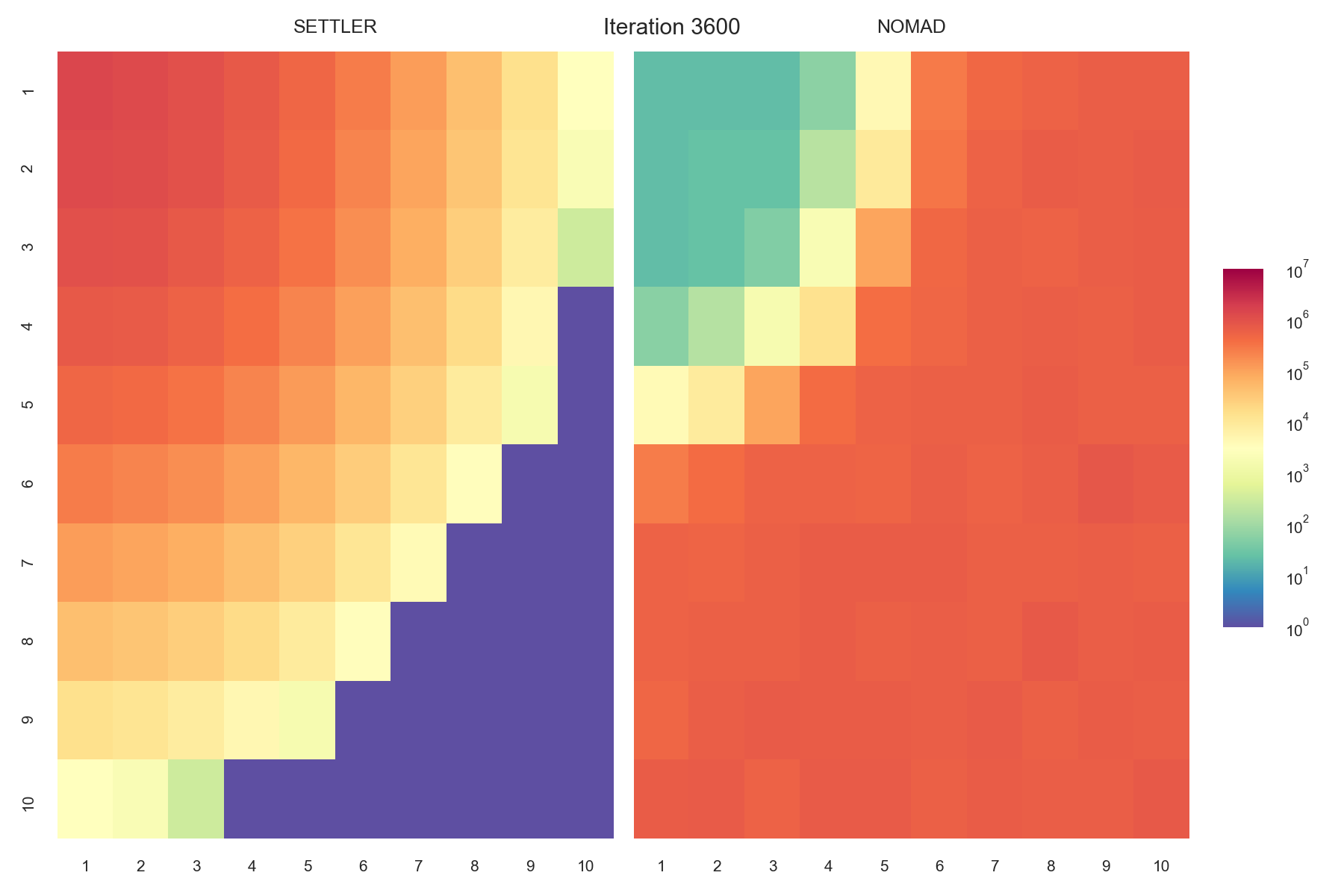

Supplement: Supplementary file 1 [file biology-10-01019-s001.zip › Spatio-temporal dynamics heatmaps/chempenoff_extremelyscarce_lindeath_period1000/3600.png]

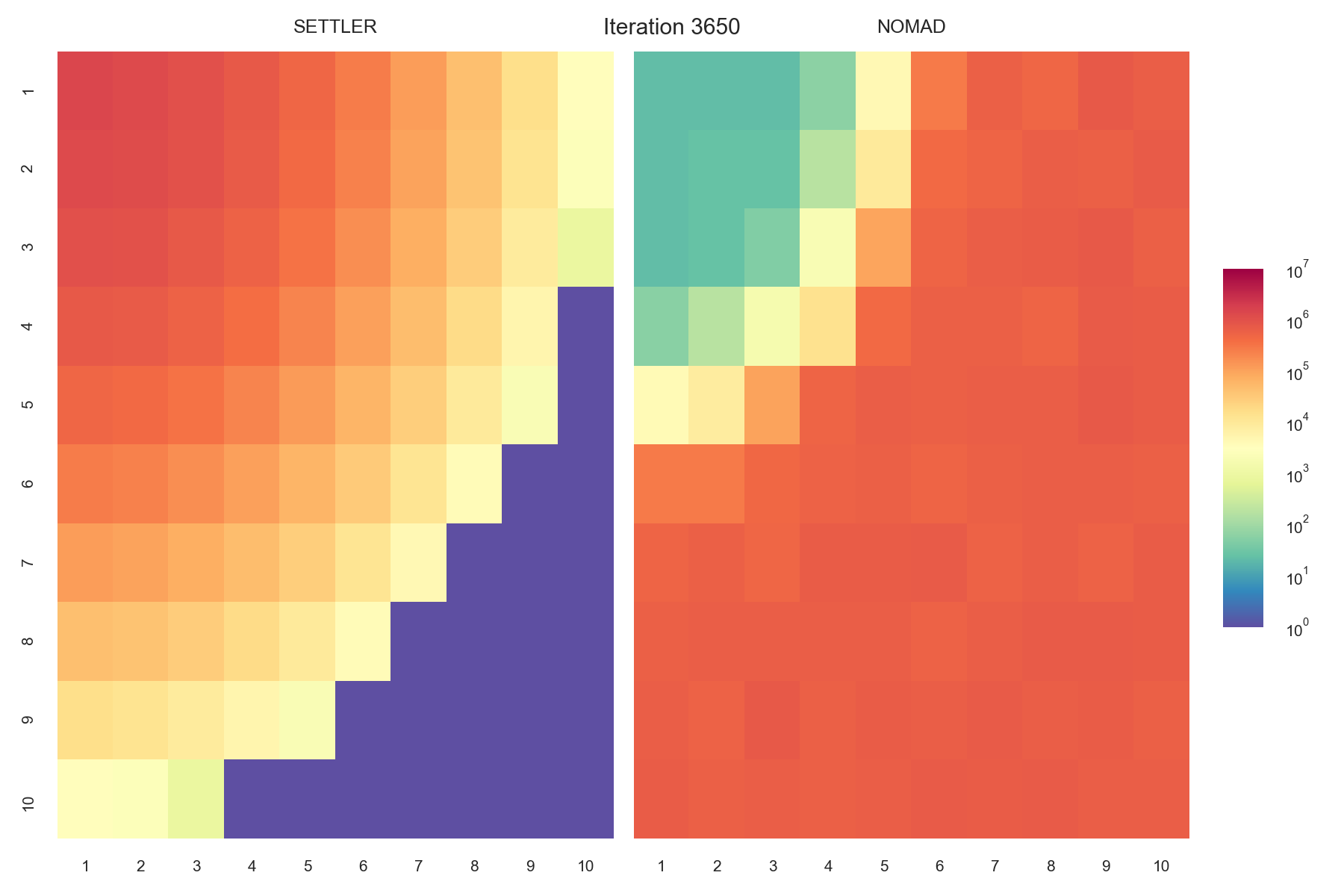

Supplement: Supplementary file 1 [file biology-10-01019-s001.zip › Spatio-temporal dynamics heatmaps/chempenoff_extremelyscarce_lindeath_period1000/3650.png]

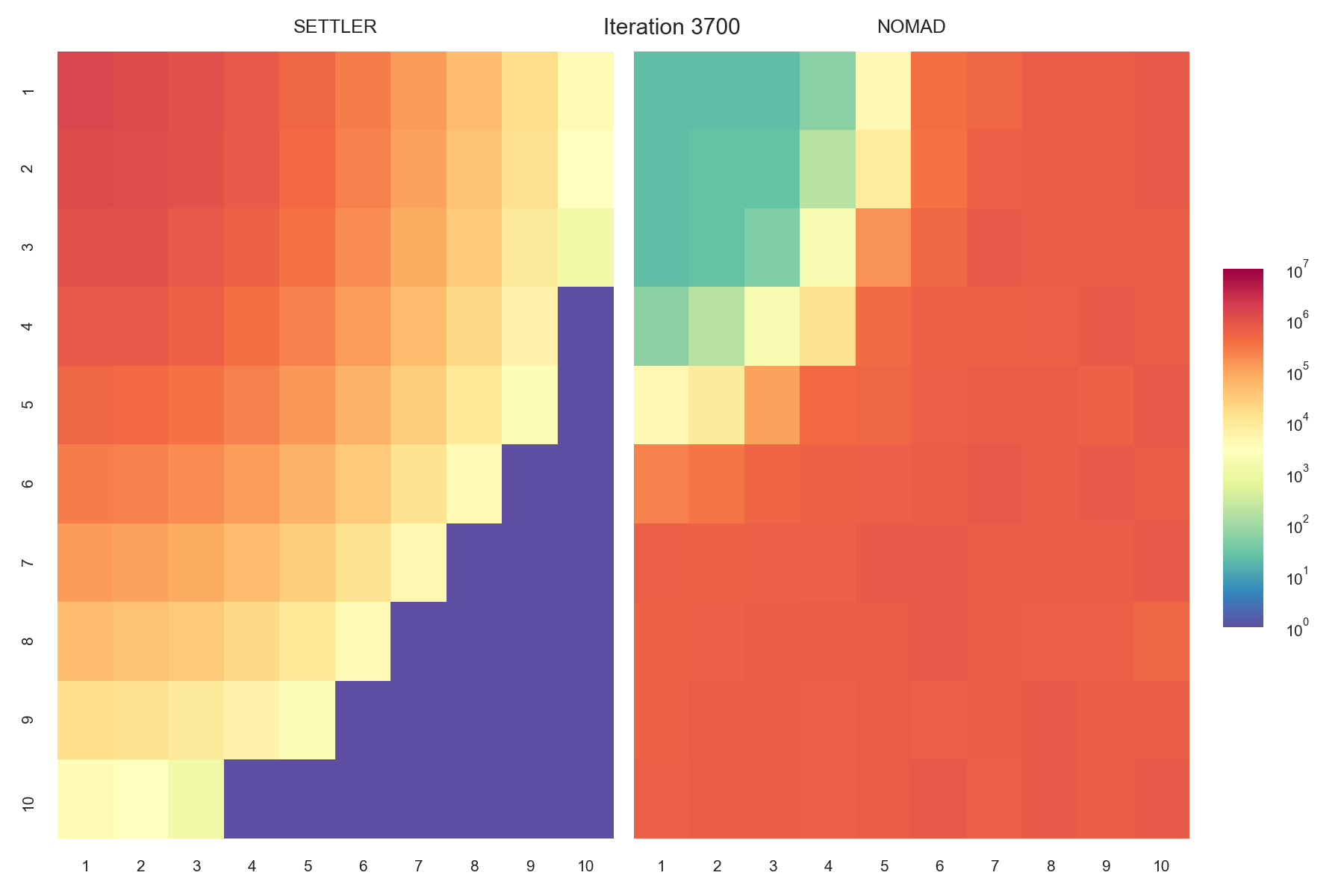

Supplement: Supplementary file 1 [file biology-10-01019-s001.zip › Spatio-temporal dynamics heatmaps/chempenoff_extremelyscarce_lindeath_period1000/3700.png]

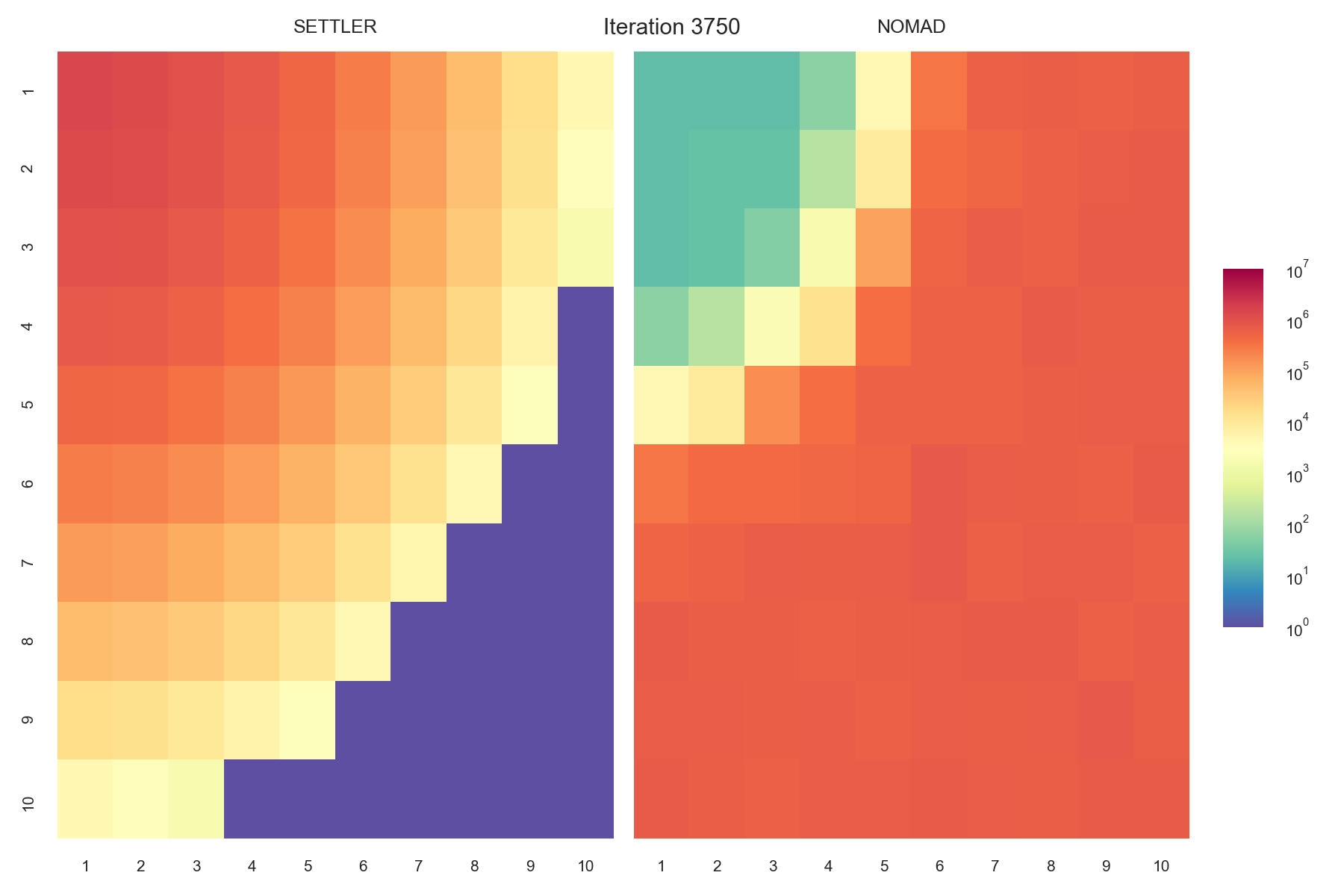

Supplement: Supplementary file 1 [file biology-10-01019-s001.zip › Spatio-temporal dynamics heatmaps/chempenoff_extremelyscarce_lindeath_period1000/3750.png]

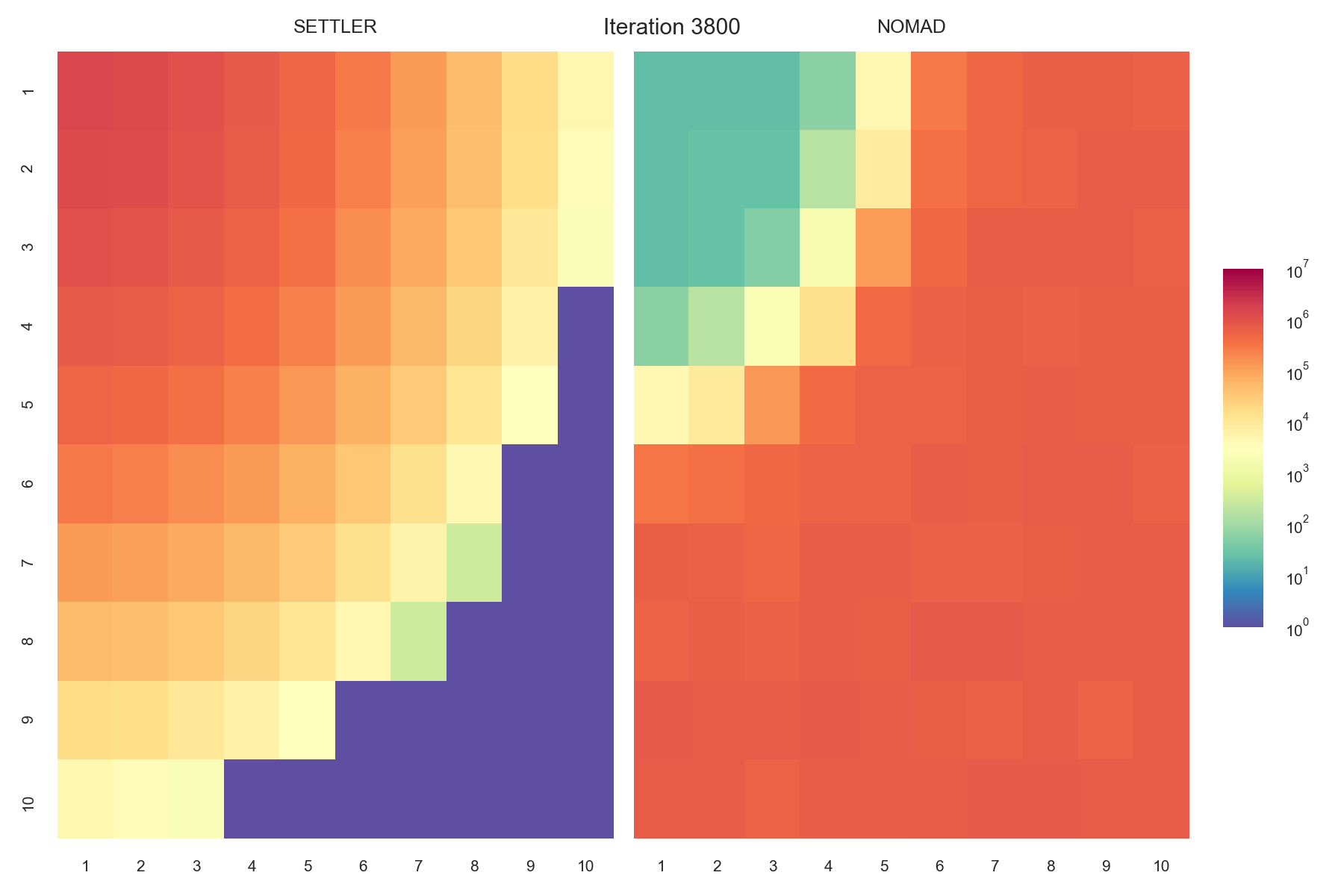

Supplement: Supplementary file 1 [file biology-10-01019-s001.zip › Spatio-temporal dynamics heatmaps/chempenoff_extremelyscarce_lindeath_period1000/3800.png]

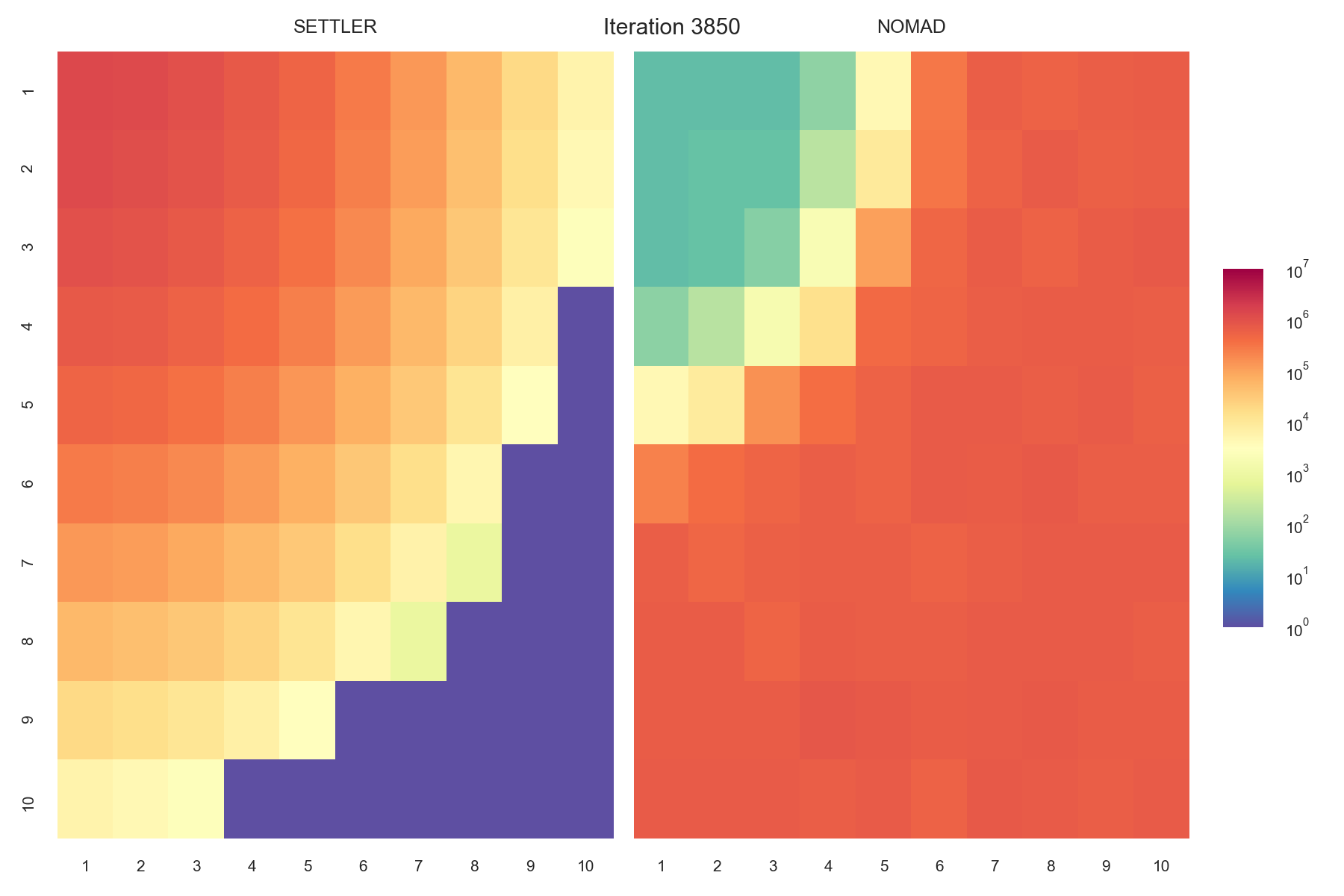

Supplement: Supplementary file 1 [file biology-10-01019-s001.zip › Spatio-temporal dynamics heatmaps/chempenoff_extremelyscarce_lindeath_period1000/3850.png]

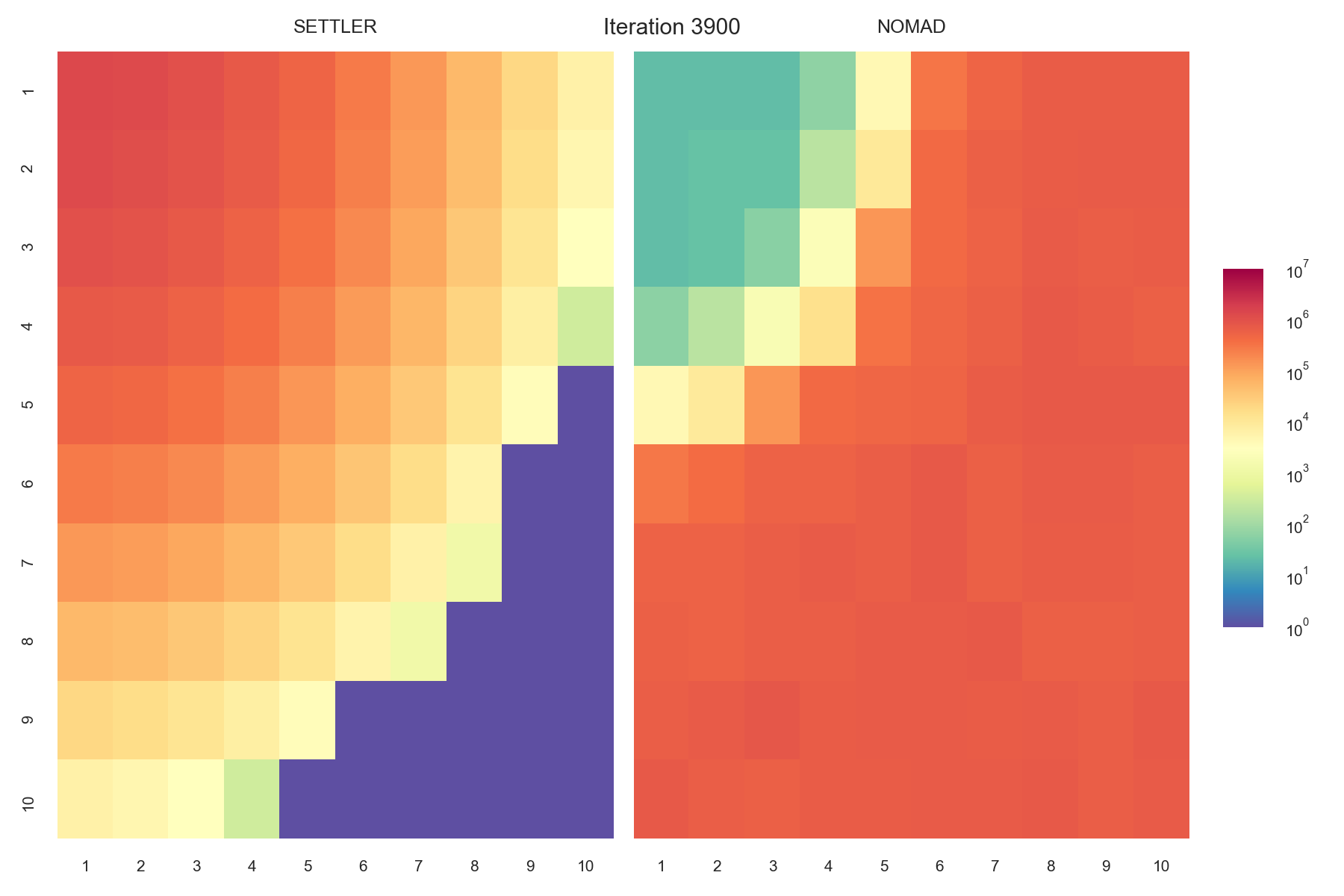

Supplement: Supplementary file 1 [file biology-10-01019-s001.zip › Spatio-temporal dynamics heatmaps/chempenoff_extremelyscarce_lindeath_period1000/3900.png]

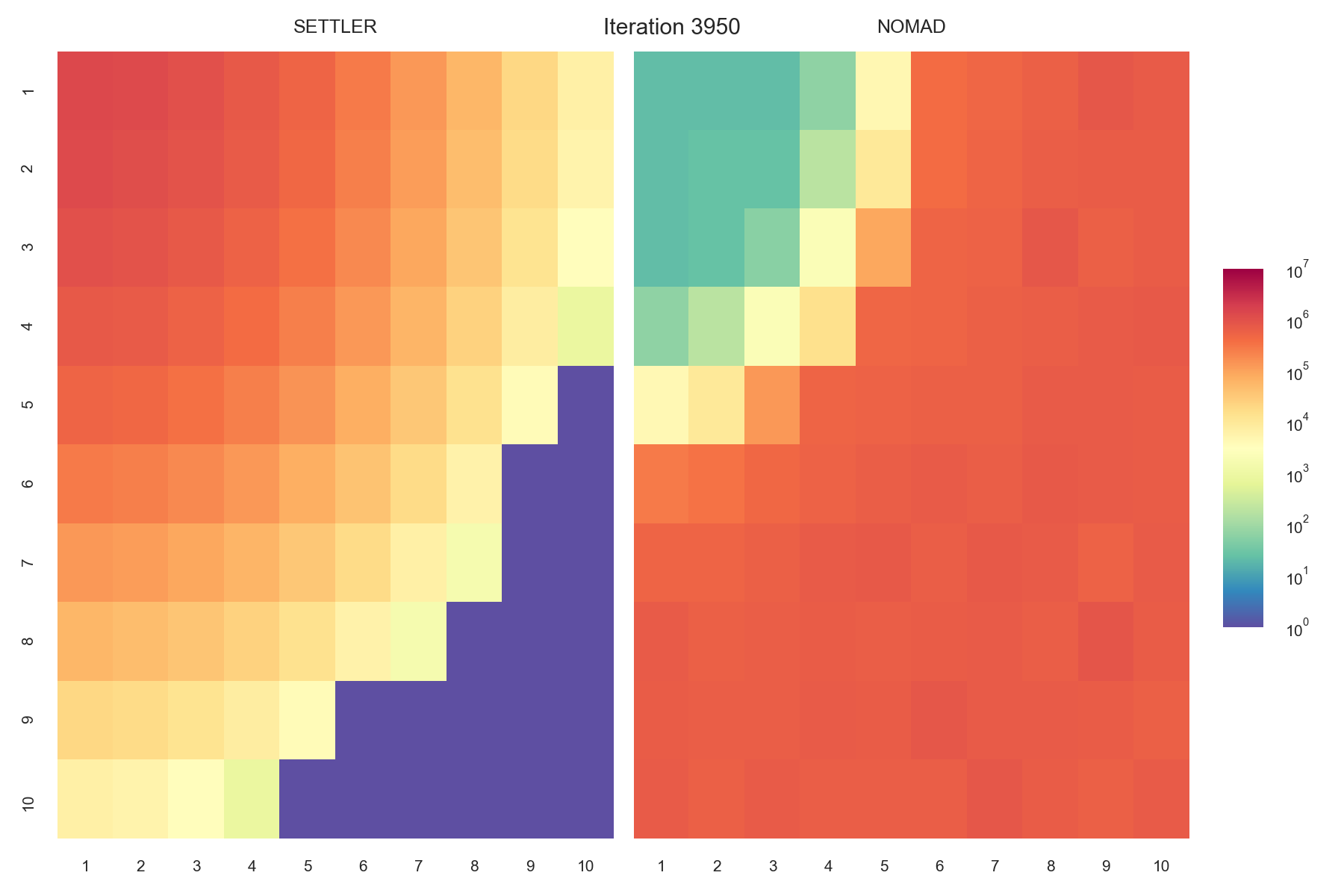

Supplement: Supplementary file 1 [file biology-10-01019-s001.zip › Spatio-temporal dynamics heatmaps/chempenoff_extremelyscarce_lindeath_period1000/3950.png]

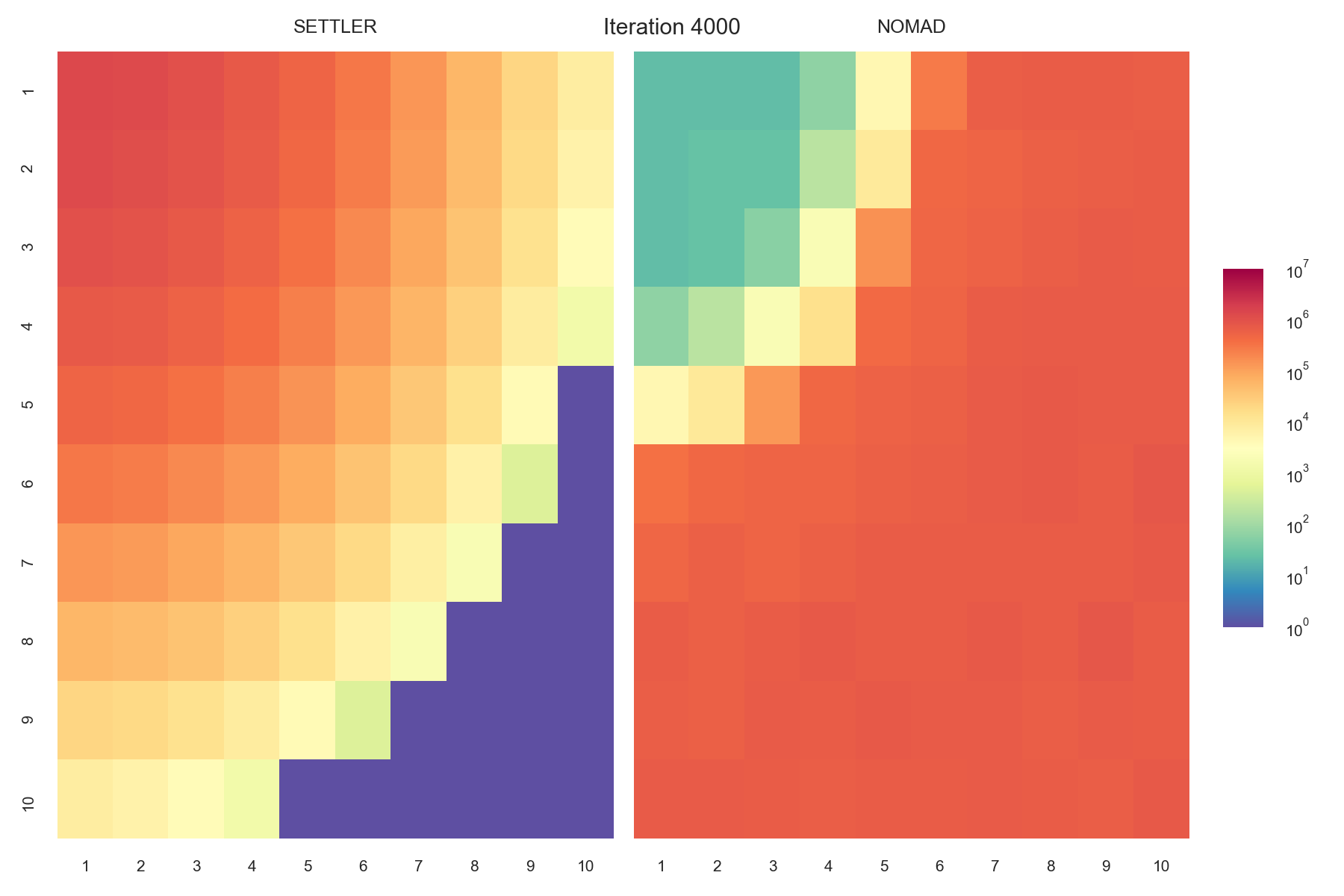

Supplement: Supplementary file 1 [file biology-10-01019-s001.zip › Spatio-temporal dynamics heatmaps/chempenoff_extremelyscarce_lindeath_period1000/4000.png]

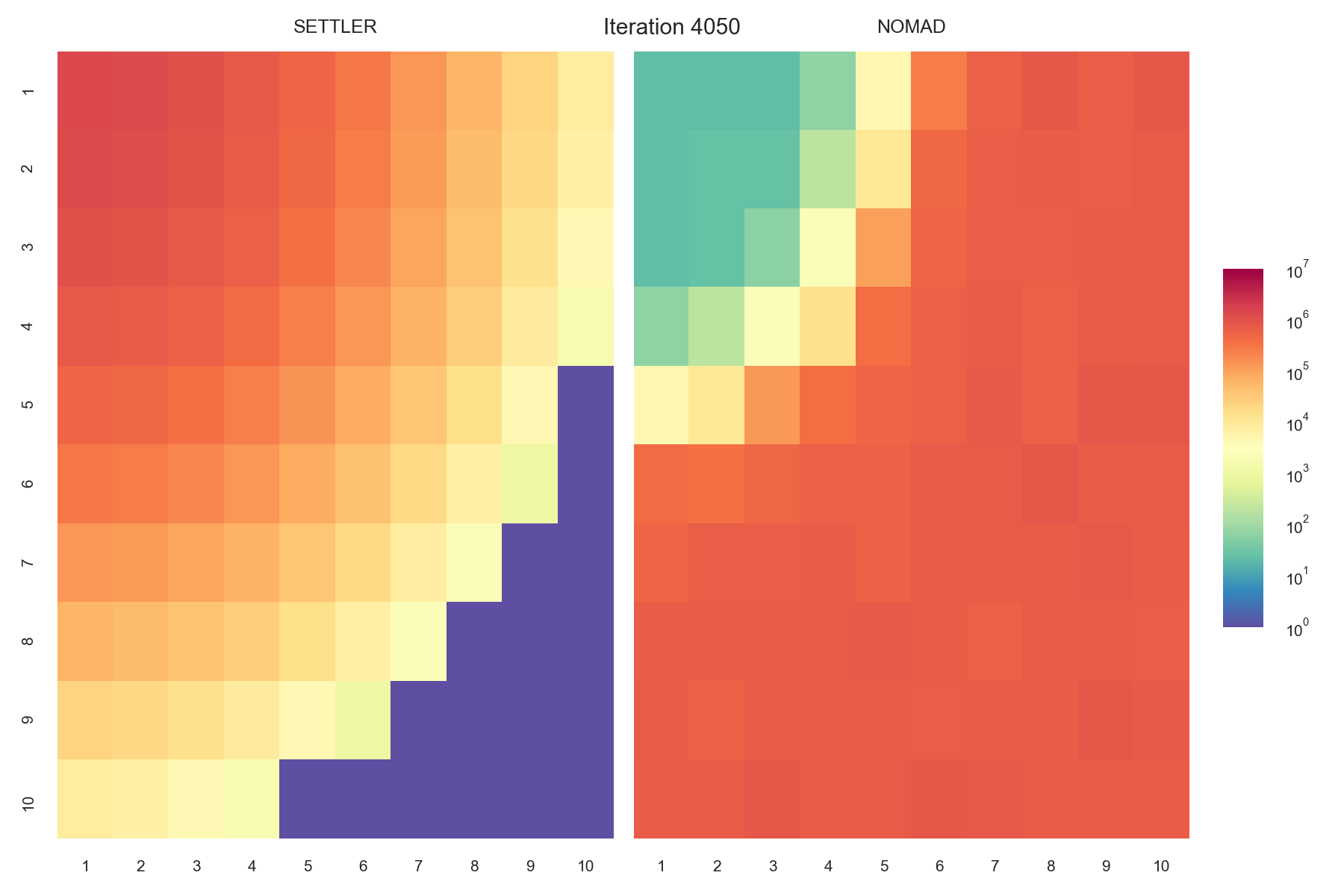

Supplement: Supplementary file 1 [file biology-10-01019-s001.zip › Spatio-temporal dynamics heatmaps/chempenoff_extremelyscarce_lindeath_period1000/4050.png]

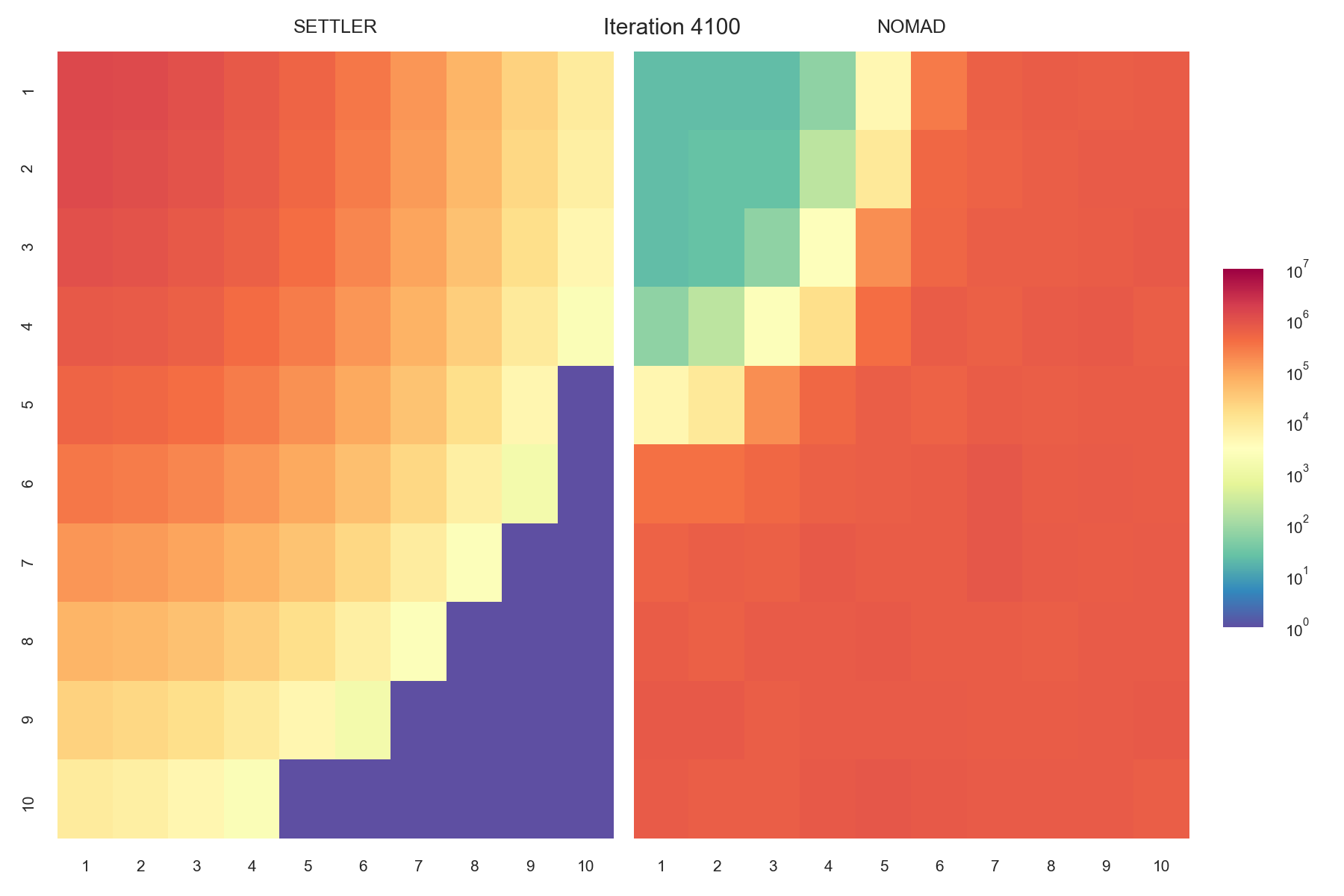

Supplement: Supplementary file 1 [file biology-10-01019-s001.zip › Spatio-temporal dynamics heatmaps/chempenoff_extremelyscarce_lindeath_period1000/4100.png]

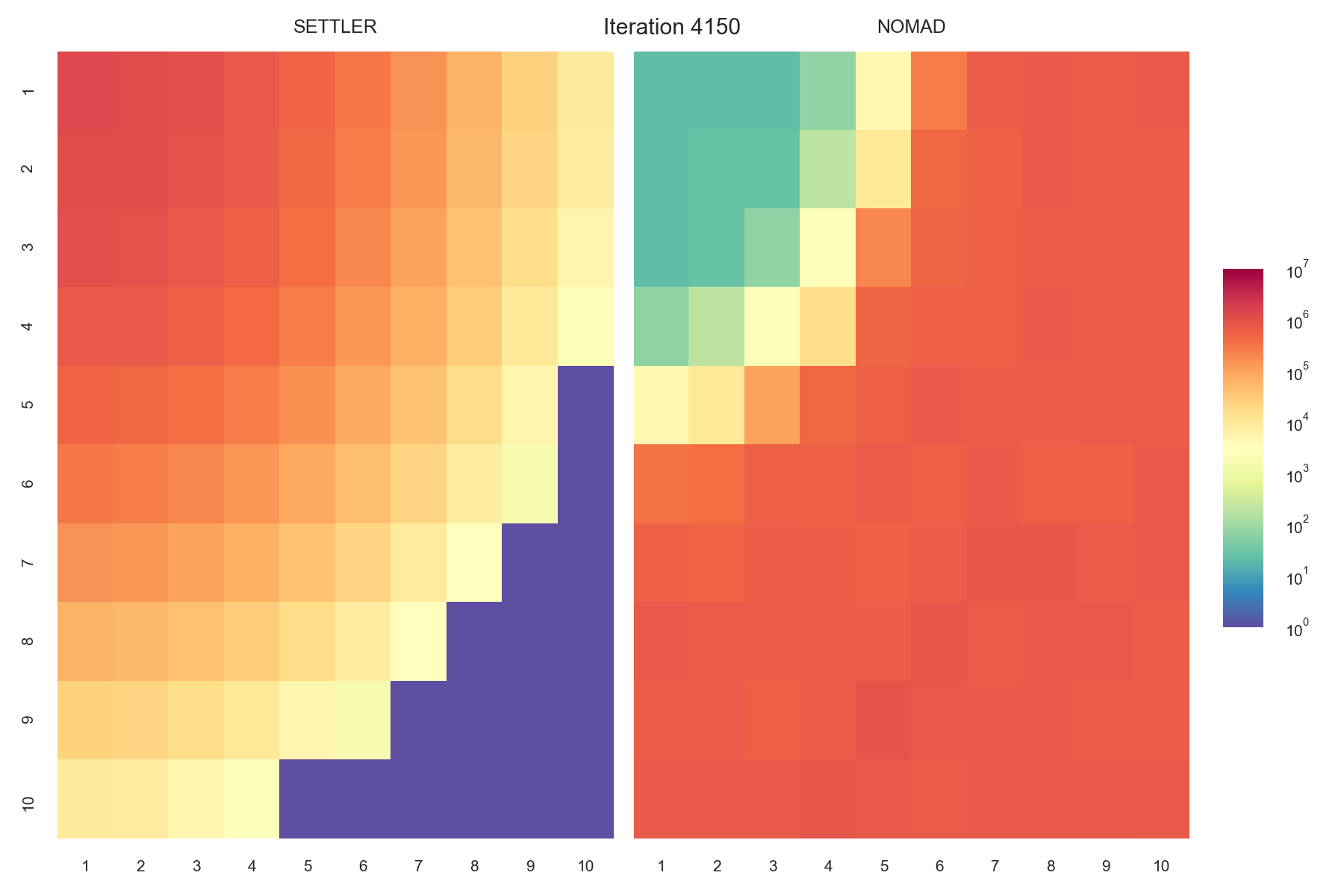

Supplement: Supplementary file 1 [file biology-10-01019-s001.zip › Spatio-temporal dynamics heatmaps/chempenoff_extremelyscarce_lindeath_period1000/4150.png]

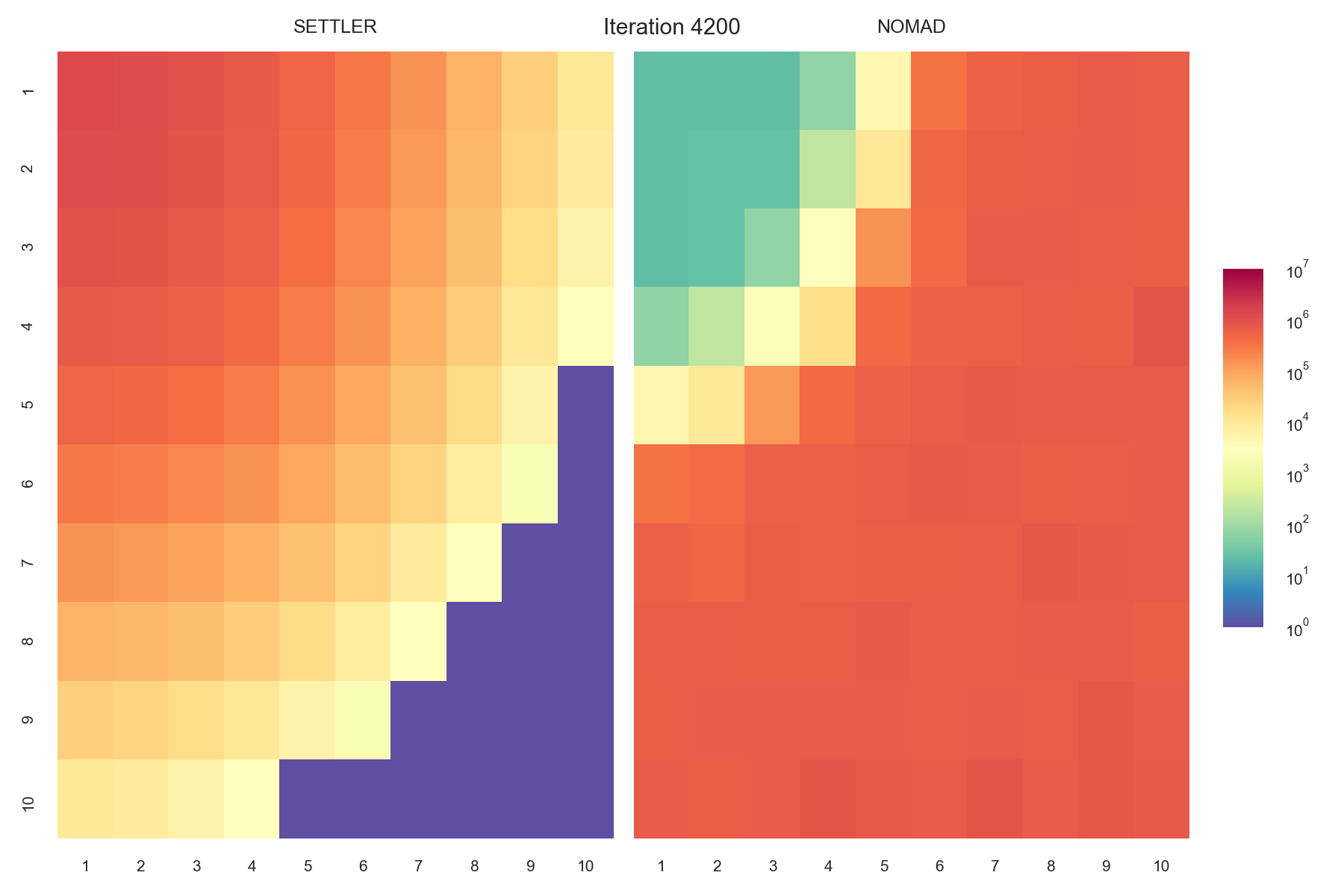

Supplement: Supplementary file 1 [file biology-10-01019-s001.zip › Spatio-temporal dynamics heatmaps/chempenoff_extremelyscarce_lindeath_period1000/4200.png]

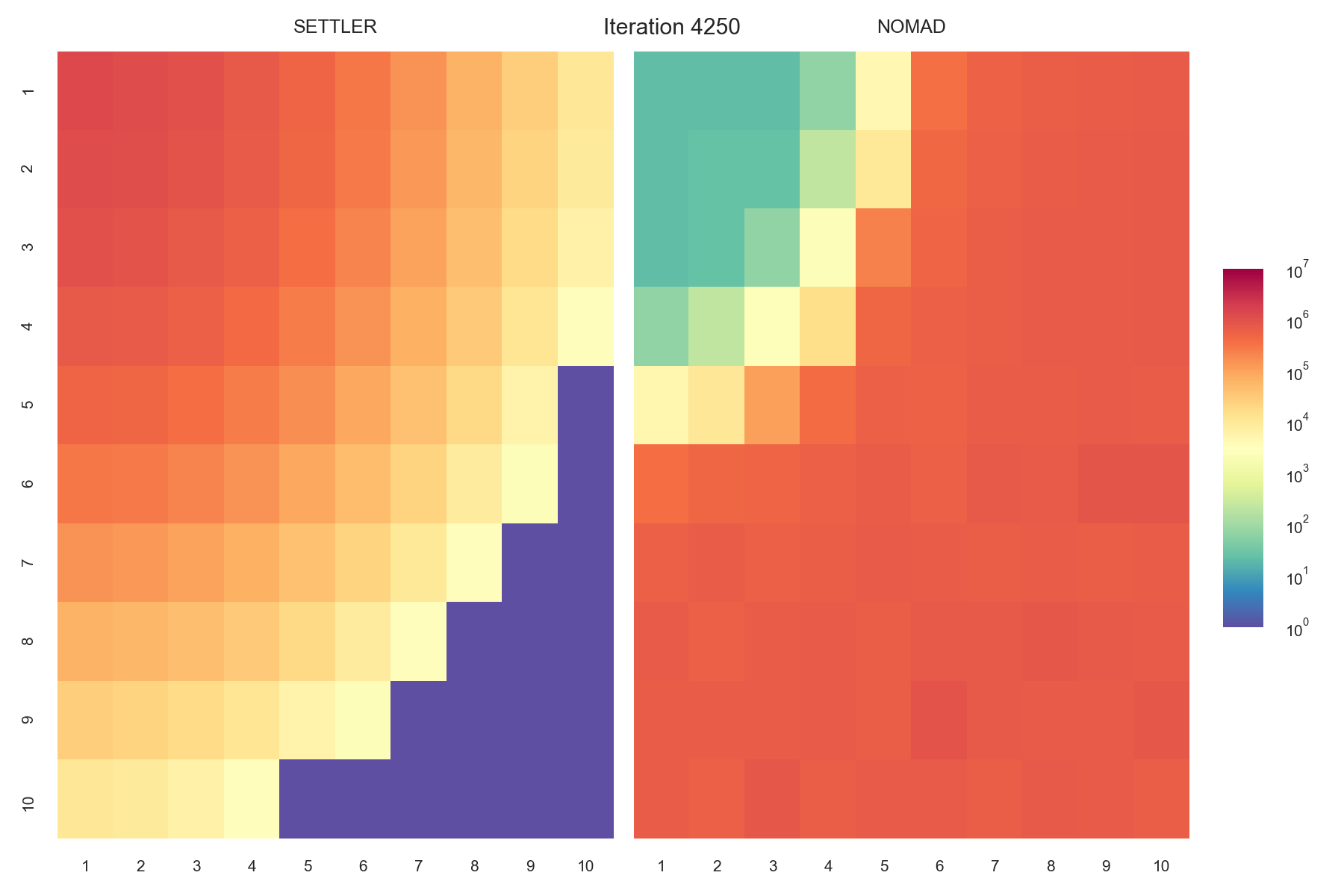

Supplement: Supplementary file 1 [file biology-10-01019-s001.zip › Spatio-temporal dynamics heatmaps/chempenoff_extremelyscarce_lindeath_period1000/4250.png]

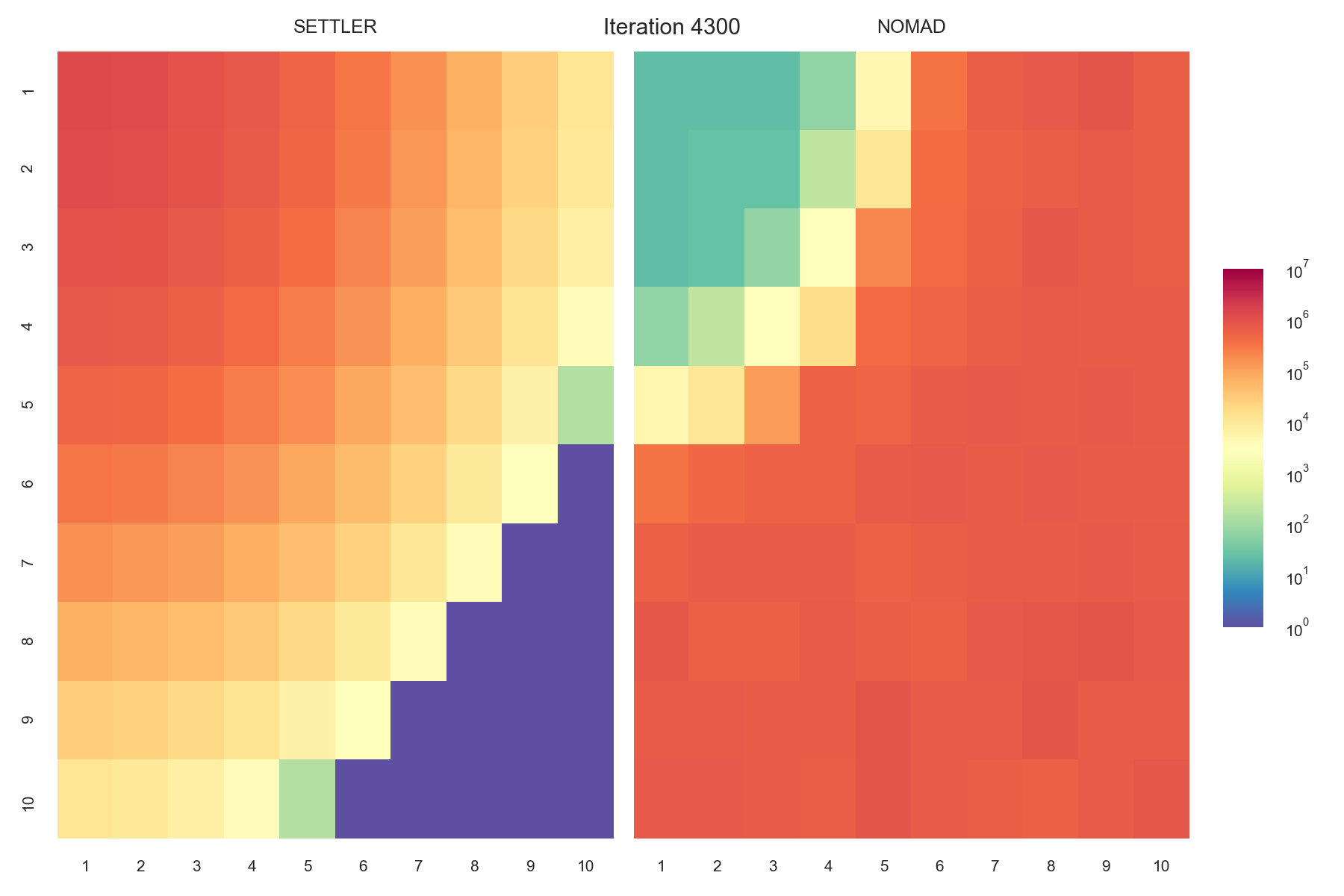

Supplement: Supplementary file 1 [file biology-10-01019-s001.zip › Spatio-temporal dynamics heatmaps/chempenoff_extremelyscarce_lindeath_period1000/4300.png]

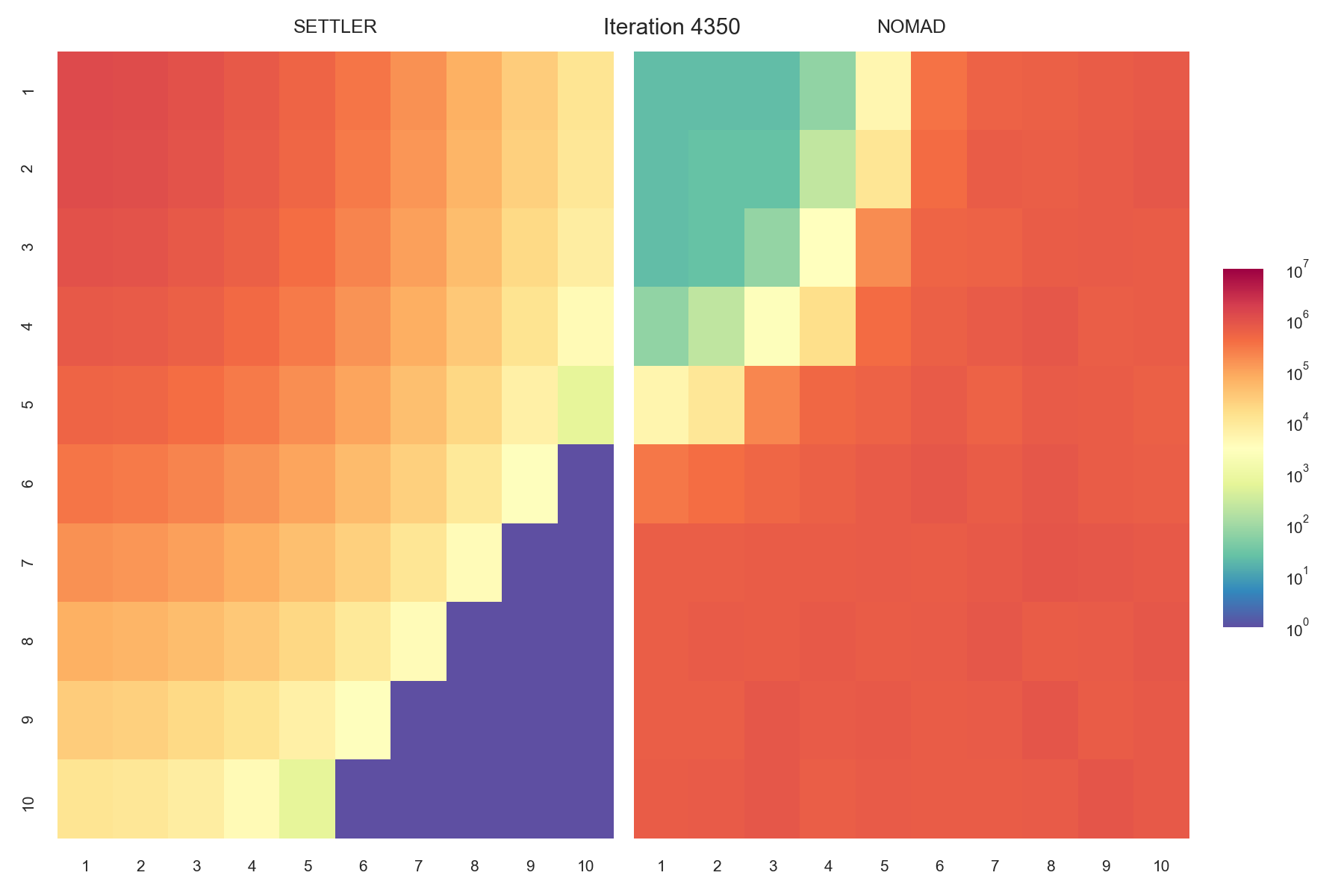

Supplement: Supplementary file 1 [file biology-10-01019-s001.zip › Spatio-temporal dynamics heatmaps/chempenoff_extremelyscarce_lindeath_period1000/4350.png]

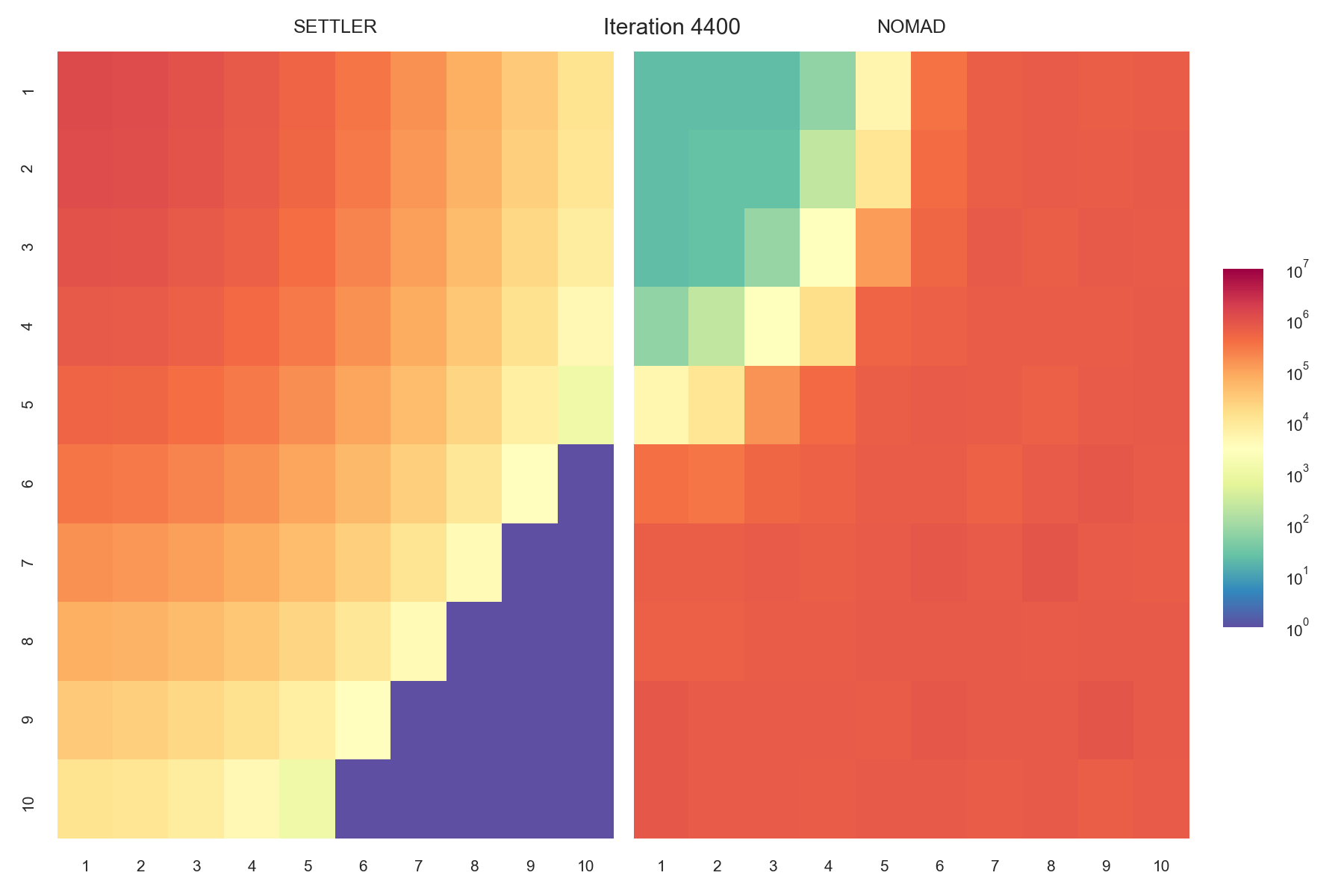

Supplement: Supplementary file 1 [file biology-10-01019-s001.zip › Spatio-temporal dynamics heatmaps/chempenoff_extremelyscarce_lindeath_period1000/4400.png]

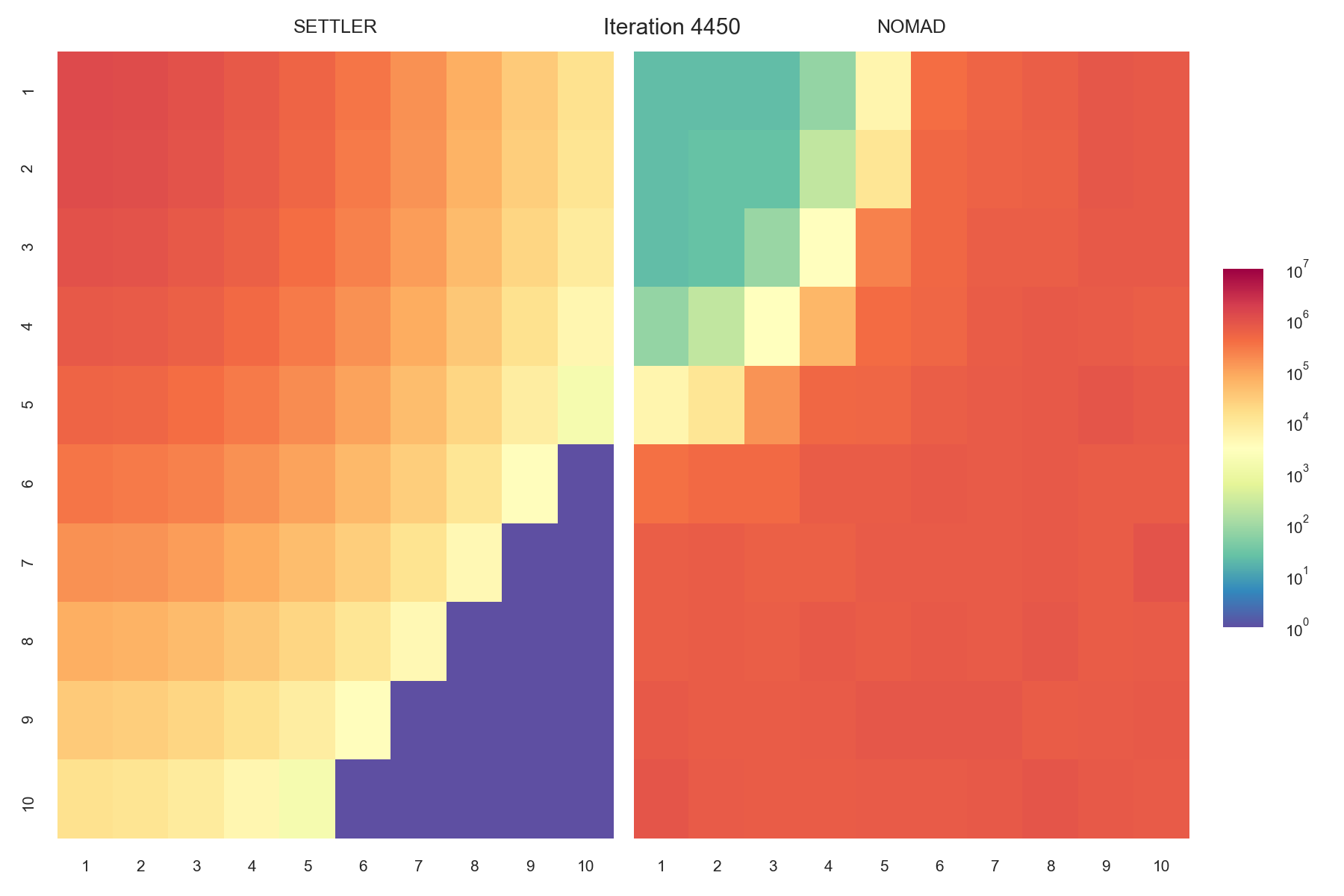

Supplement: Supplementary file 1 [file biology-10-01019-s001.zip › Spatio-temporal dynamics heatmaps/chempenoff_extremelyscarce_lindeath_period1000/4450.png]

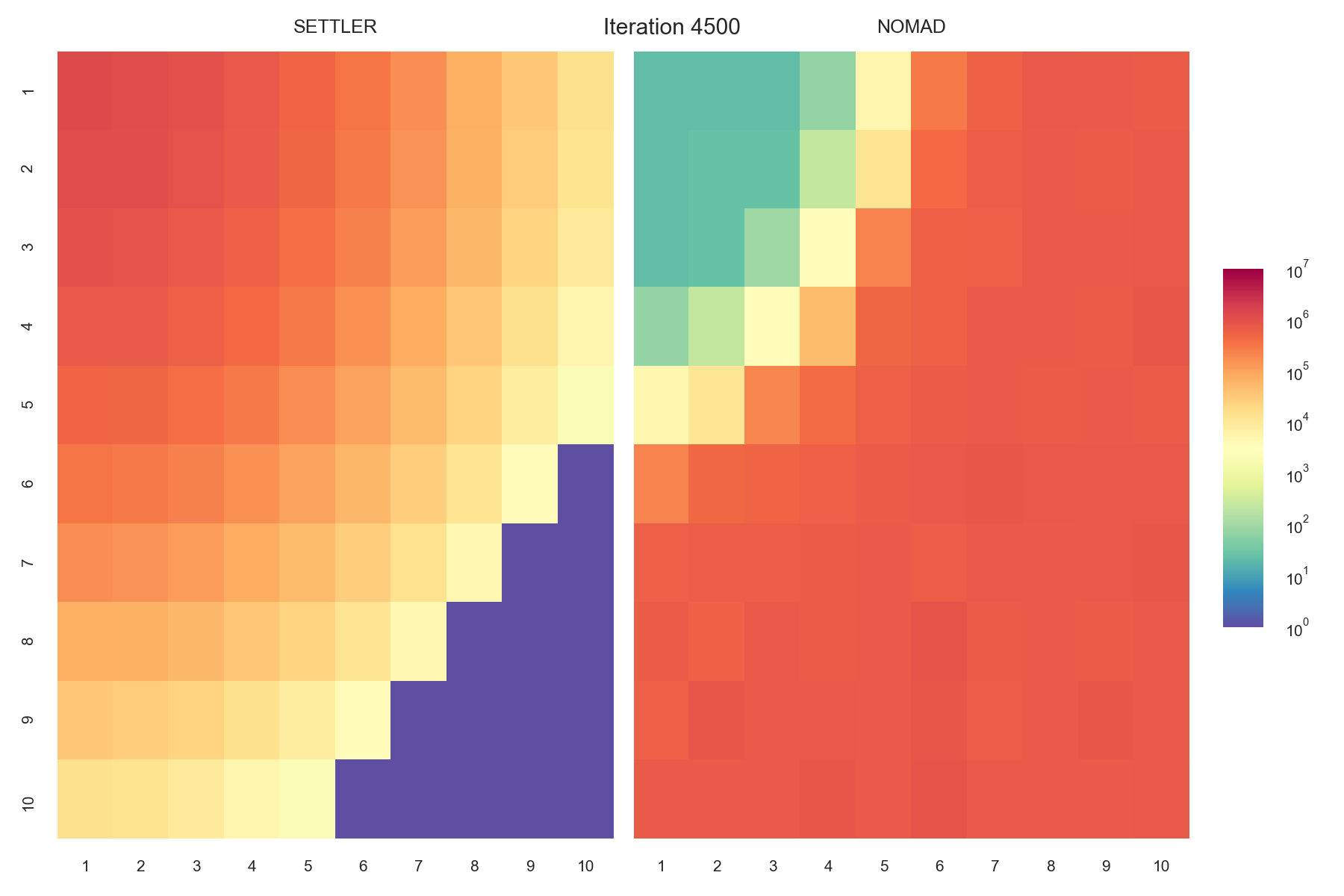

Supplement: Supplementary file 1 [file biology-10-01019-s001.zip › Spatio-temporal dynamics heatmaps/chempenoff_extremelyscarce_lindeath_period1000/4500.png]

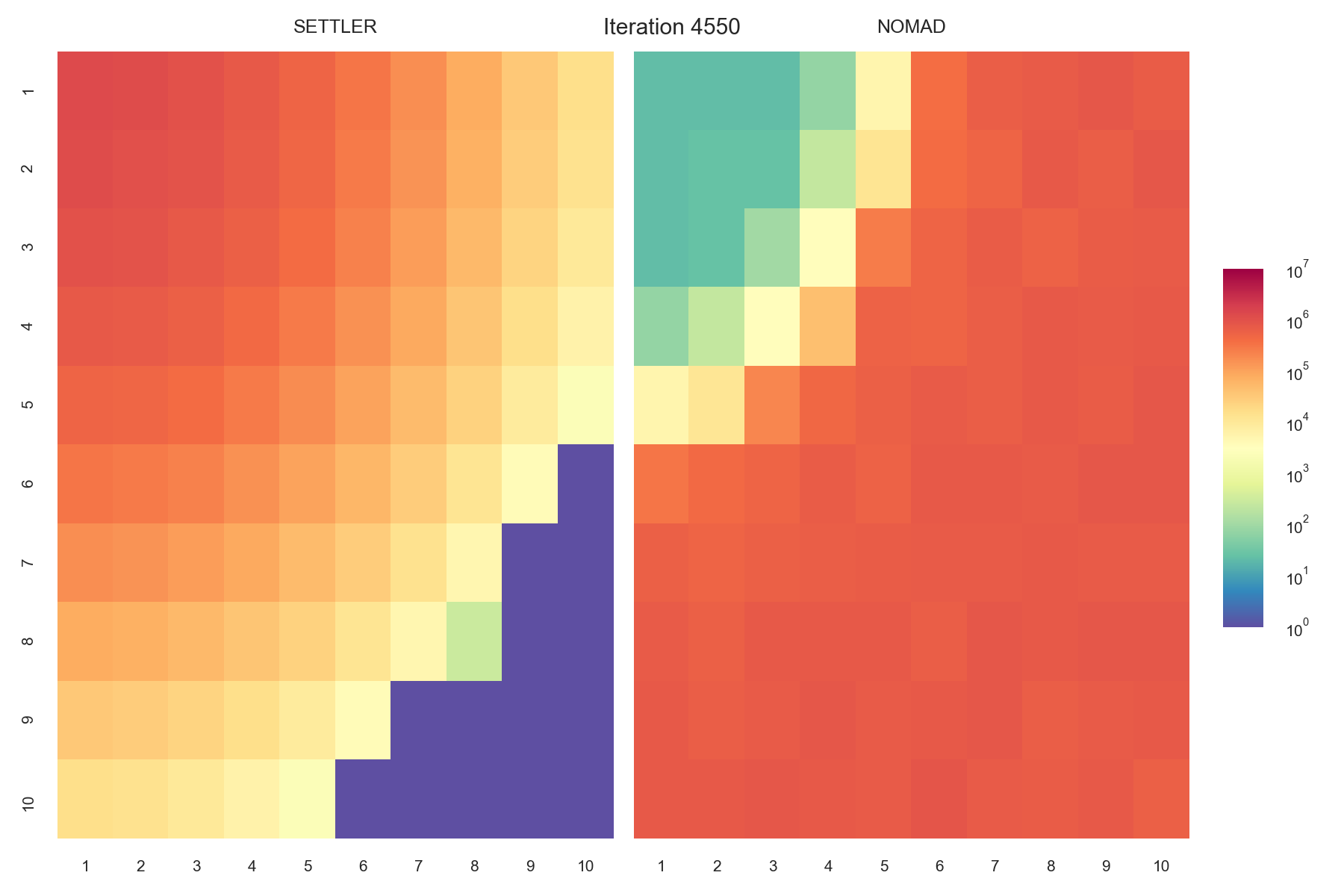

Supplement: Supplementary file 1 [file biology-10-01019-s001.zip › Spatio-temporal dynamics heatmaps/chempenoff_extremelyscarce_lindeath_period1000/4550.png]

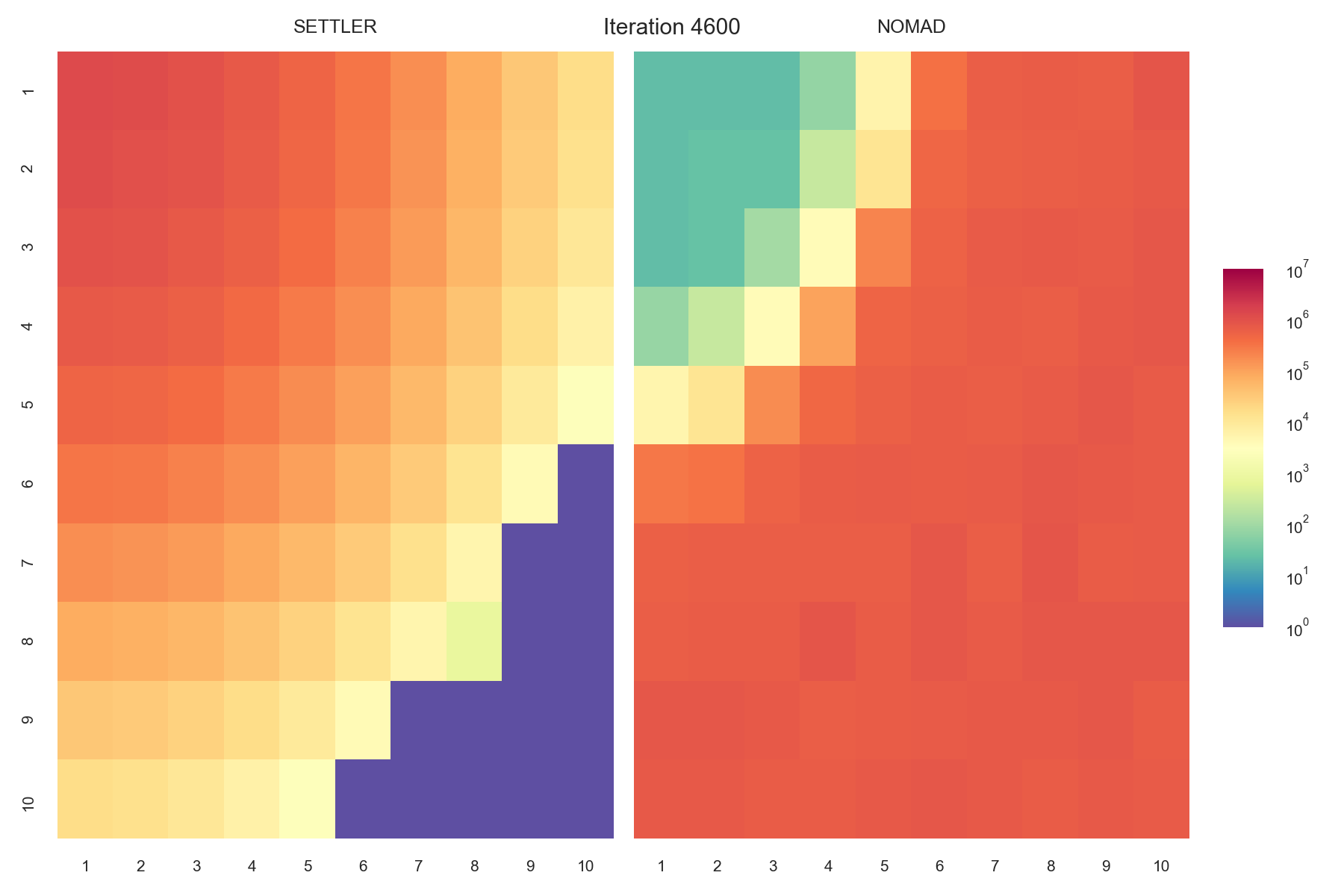

Supplement: Supplementary file 1 [file biology-10-01019-s001.zip › Spatio-temporal dynamics heatmaps/chempenoff_extremelyscarce_lindeath_period1000/4600.png]

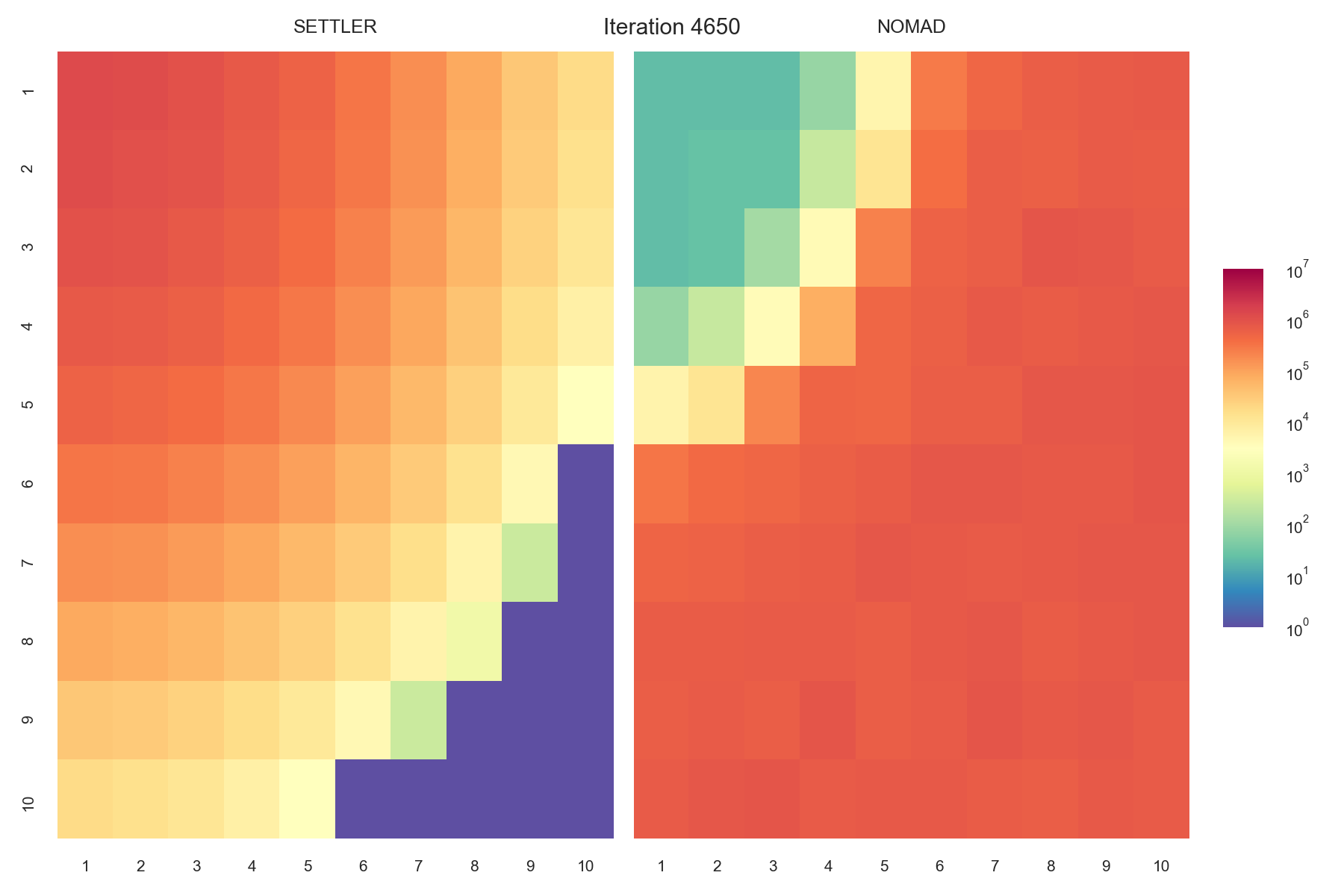

Supplement: Supplementary file 1 [file biology-10-01019-s001.zip › Spatio-temporal dynamics heatmaps/chempenoff_extremelyscarce_lindeath_period1000/4650.png]

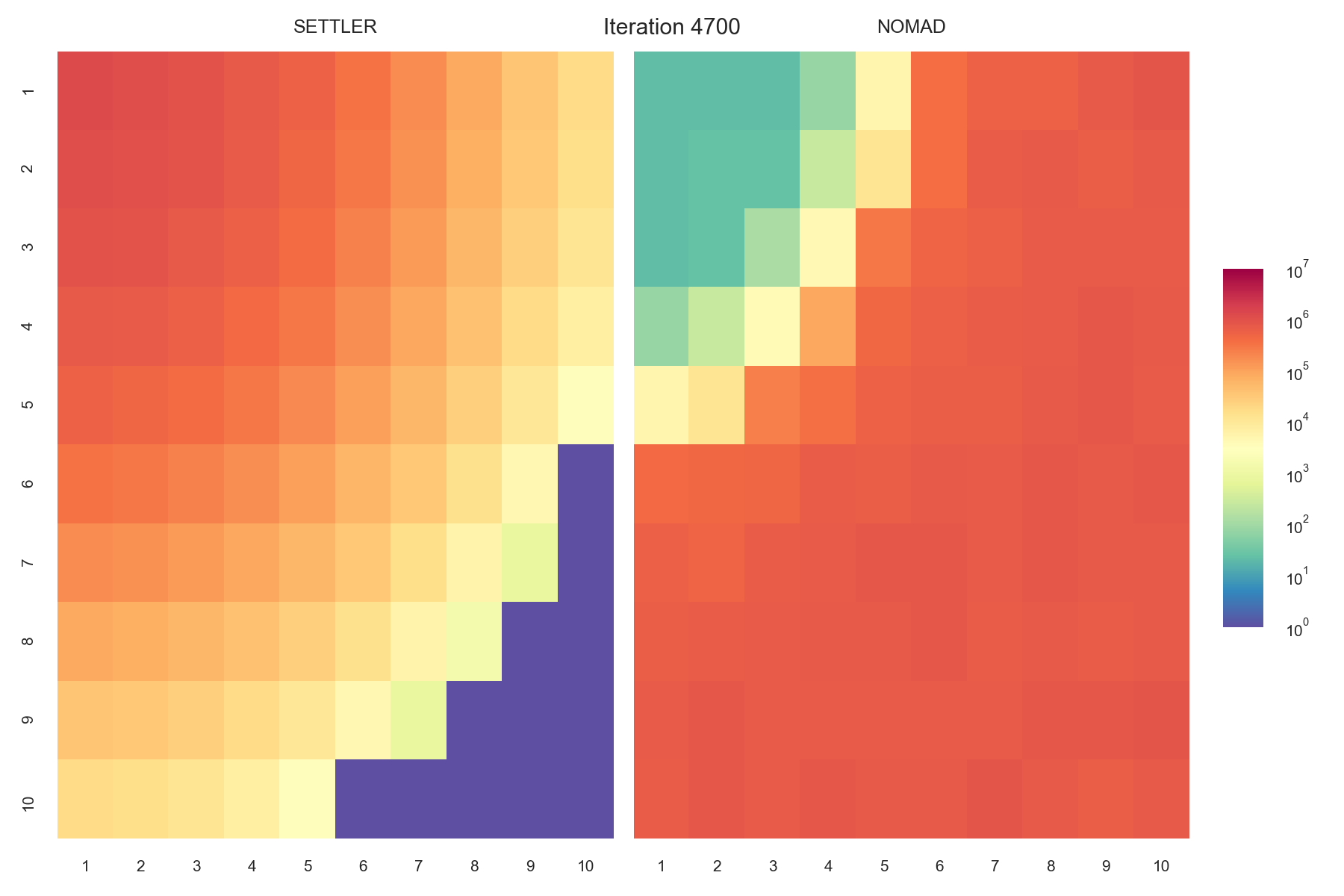

Supplement: Supplementary file 1 [file biology-10-01019-s001.zip › Spatio-temporal dynamics heatmaps/chempenoff_extremelyscarce_lindeath_period1000/4700.png]

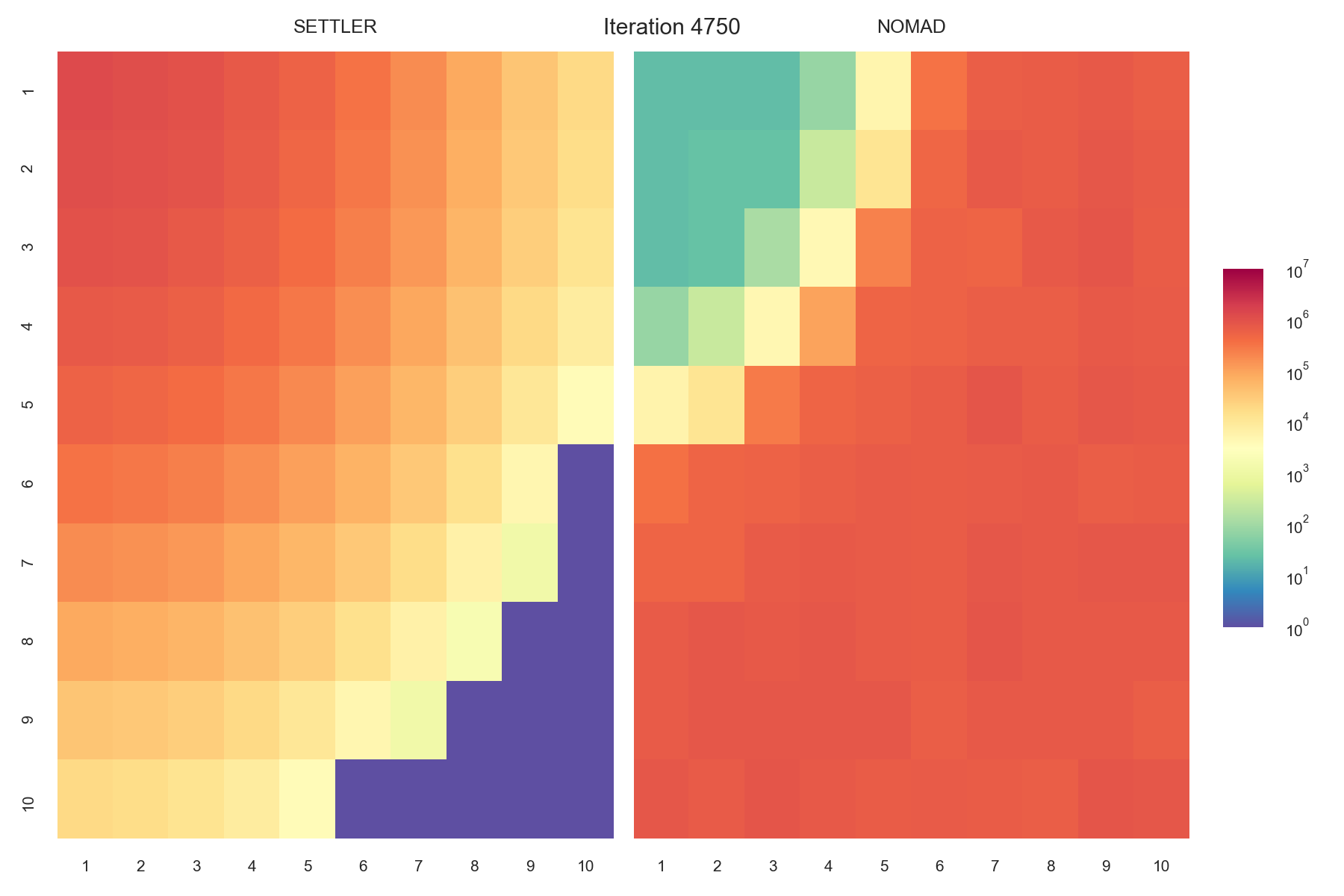

Supplement: Supplementary file 1 [file biology-10-01019-s001.zip › Spatio-temporal dynamics heatmaps/chempenoff_extremelyscarce_lindeath_period1000/4750.png]

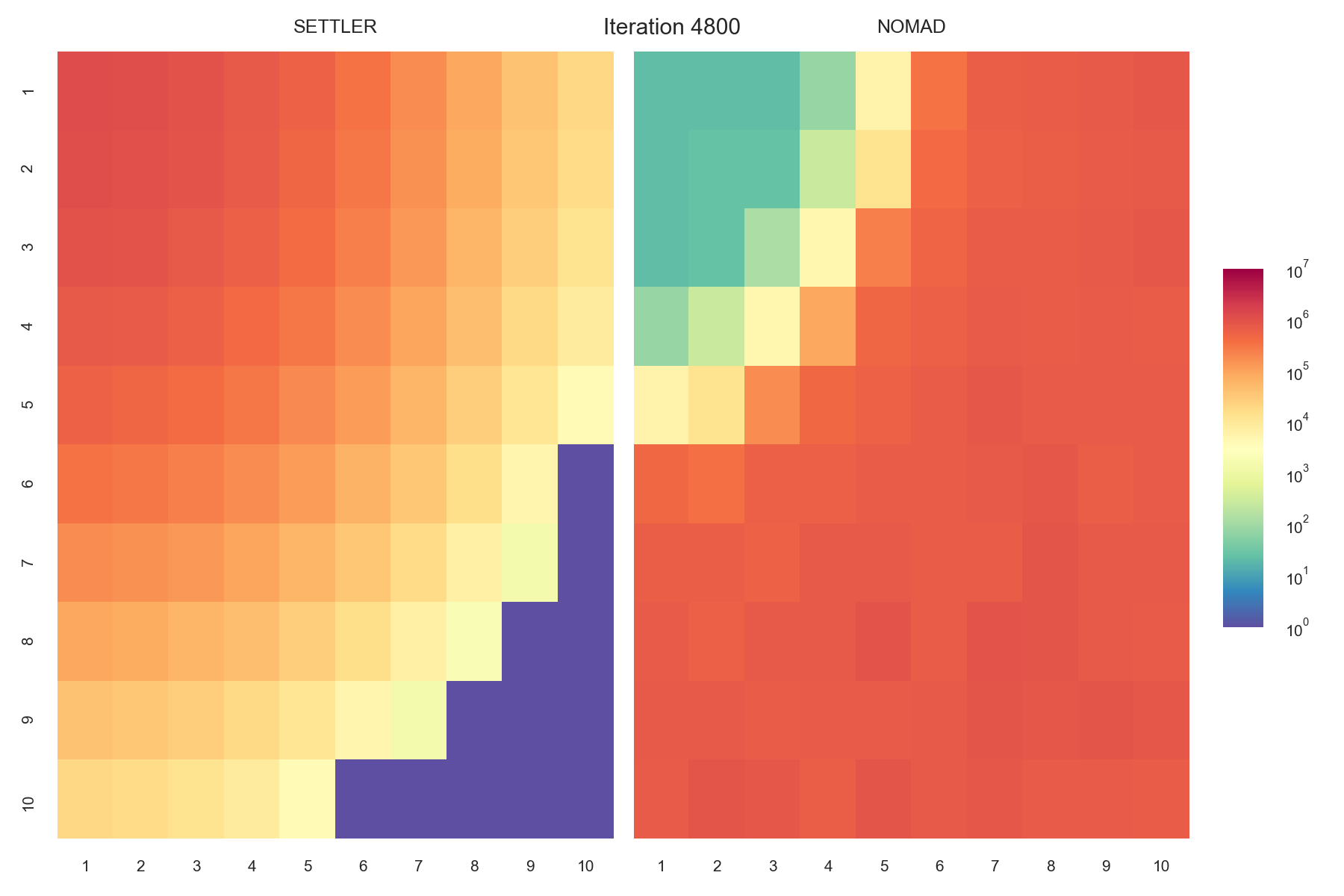

Supplement: Supplementary file 1 [file biology-10-01019-s001.zip › Spatio-temporal dynamics heatmaps/chempenoff_extremelyscarce_lindeath_period1000/4800.png]

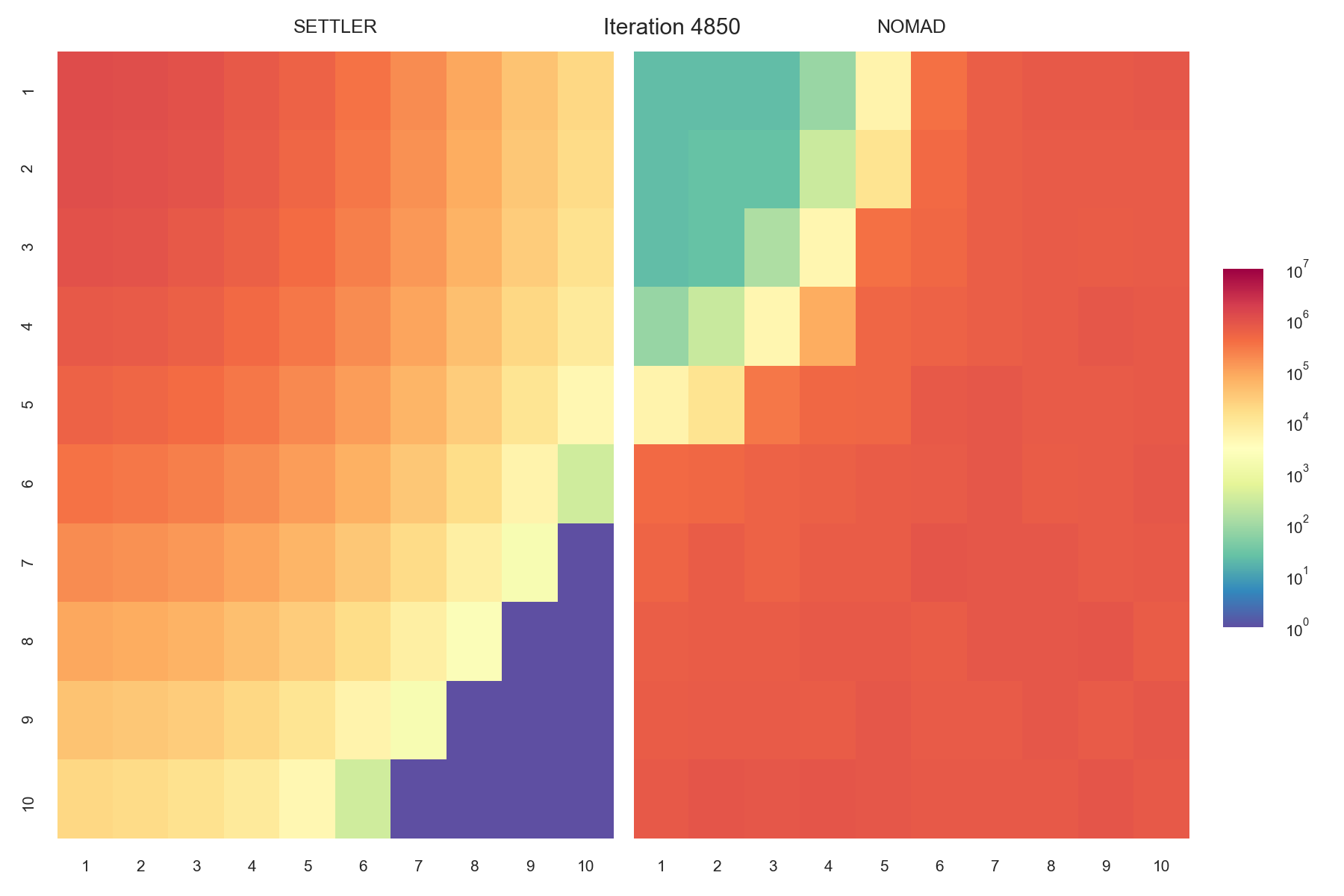

Supplement: Supplementary file 1 [file biology-10-01019-s001.zip › Spatio-temporal dynamics heatmaps/chempenoff_extremelyscarce_lindeath_period1000/4850.png]

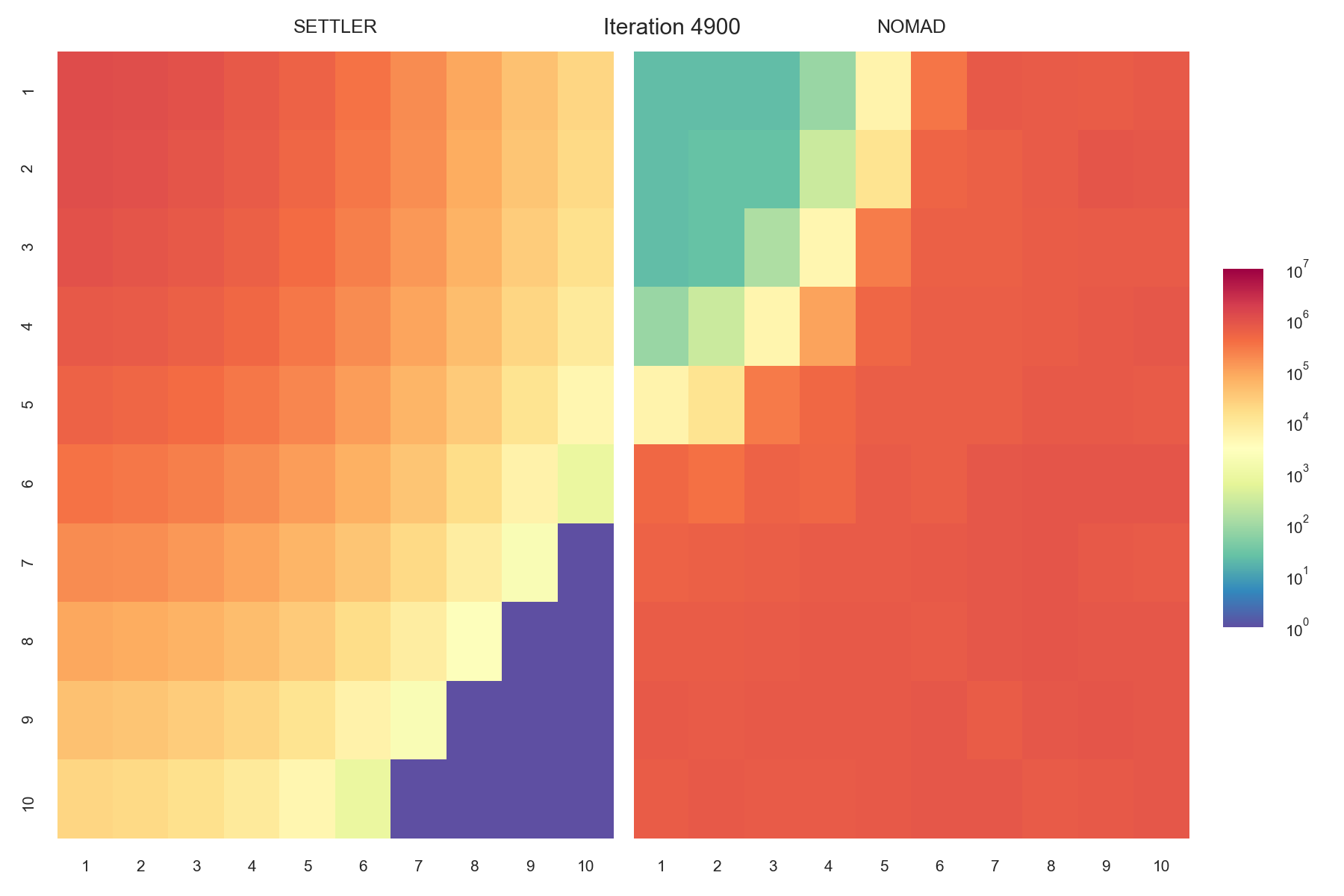

Supplement: Supplementary file 1 [file biology-10-01019-s001.zip › Spatio-temporal dynamics heatmaps/chempenoff_extremelyscarce_lindeath_period1000/4900.png]
