# Supplementary material for: Leave or Stay: Simulating Motility and Fitness of Microorganisms in Dynamic Aquatic Ecosystems
Source: Biology (Basel). 2021 Oct 9;10(10):1019. doi: 10.3390/biology10101019 (PMC8533222; doi:10.3390/biology10101019)
Supplement: Supplementary file 1 [file biology-10-01019-s001.zip › Table S1.Settler Nomad model state variables.pdf]

Table S1. Settler-Nomad model state variables.

**Variable name**	**Description**	**Unit**
cons_ns_settler(i,j)	Consumed nonspecific substrate molecules per Settler cell (energy reserve of a cell) in the node (i,j)	Number of molecules
cons_ns_nomad(i,j)	Consumed nonspecific substrate molecules per Nomad cell (energy reserve of a cell) in the node (i,j)	Number of molecules
P_settler(i,j)	Population abundance of Settler in the node (i,j)	Number of cells
P_nomad(i,j)	Population abundance of Nomad in the node (i,j)	Number of cells
NS_conc(i,j)	Nonspecific substrate concentration in the node (i,j)	M
