# Supplementary material for: Leave or Stay: Simulating Motility and Fitness of Microorganisms in Dynamic Aquatic Ecosystems
Source: Biology (Basel). 2021 Oct 9;10(10):1019. doi: 10.3390/biology10101019 (PMC8533222; doi:10.3390/biology10101019)
Supplement: Supplementary file 1 [file biology-10-01019-s001.zip › Table S2.All parameters table.pdf]

Table S2. All the parameters of the Settler-Nomad model. All population parameters are the same for Settler and Nomad unless otherwise stated.

**Parameter name**	**Description**	**Value**	**Unit**
	Environmental parameters		
V_{total}	Environmental (habitat) volume (liters)	0.6	l
NCB	Nutrient concentration in a batch	10^{-10}-0.1	M
z	Batch period	1-1000	generations
k_d *(D)*	Diffusion rate parameter (Diffusion coefficient)	0.001 ($1.698\cdot10^{-6}$)	$\frac{g\cdot m^2}{mol\cdot s}\left(\frac{cm^2}{s}\right)$
	Population parameters		
k_{death}	Death rate of a population (for quadratic mortality term)	10^{-12}	1/cell
k_{death}	Death rate of a population (for linear mortality term)	10^{-12}	dimensionless
$V_{consumed}$	Average environmental volume infiltrated through a cell	$5.4\cdot10^{-12}$	liters/generation
k_{free}	Portion of "free"-floating (plankton) cells	1	dimensionless
m	The fraction of actively moving cells in a population	10 (for Nomad) / 0 (for Settler)	%
M	The maximal amount of nutrient molecules that can be consumed per cell per generation	10^7	number of molecules / cell / generation
x	Basic migratory costs	0-10	dimensionless
h	Degree of nonlinearity between the migratory costs function and basic migration costs	4	dimensionless
C(x)	Migratory costs function	0-0.9999	dimensionless
k_{pass}	Passive migration rate	0.001	dimensionless
K	Parameter of the growth function determining the growth range	5	dimensionless
B	Parameter of the growth function controlling the value of basal amount of growth	0.8	dimensionless
N	Genetically determined nutrient utilization efficacy	1	dimensionless
R	Costs for reproduction	0.7992	dimensionless
k_{degrad}	Nutrient degradation rate	0.1998	dimensionless
